# A three-dimensional stereotaxic atlas of the gray short-tailed opossum (*Monodelphis domestica*) brain

**DOI:** 10.1007/s00429-017-1540-x

**Published:** 2017-12-06

**Authors:** Piotr Majka, Natalia Chlodzinska, Krzysztof Turlejski, Tomasz Banasik, Ruzanna L. Djavadian, Władysław P. Węglarz, Daniel K. Wójcik

**Affiliations:** 1 Laboratory of Neuroinformatics, Department of Neurophysiology, Nencki Institute of Experimental Biology of Polish Academy of Sciences, 3 Pasteur Street, 02-093 Warsaw, Poland; 2 Laboratory of Neurobiology of Development and Evolution, Nencki Institute of Experimental Biology of Polish Academy of Sciences, 3 Pasteur Street, 02-093 Warsaw, Poland; 3 Department of Biology and Environmental Science, Cardinal Stefan Wyszynski University, 1/3 Woycicki Street, 01-938 Warsaw, Poland; 4 H. Niewodniczański Institute of Nuclear Physics of Polish Academy of Sciences, Radzikowskiego 152, 31-342 Kraków, Poland; 5 Department of Molecular and Cellular Neurobiology, Nencki Institute of Experimental Biology of Polish Academy of Sciences, 3 Pasteur Street, 02-093 Warsaw, Poland

**Keywords:** Monodelphis opossum, Brain atlas, MRI, Neuroanatomy, Digital atlas, Image registration, Nissl staining, Brain template, 3D visualization

## Abstract

The gray short-tailed opossum (*Monodelphis domestica*) is a small marsupial gaining recognition as a laboratory animal in biomedical research. Despite numerous studies on opossum neuroanatomy, a consistent and comprehensive neuroanatomical reference for this species is still missing. Here we present the first three-dimensional, multimodal atlas of the *Monodelphis* opossum brain. It is based on four complementary imaging modalities: high resolution ex vivo magnetic resonance images, micro-computed tomography scans of the cranium, images of the face of the cutting block, and series of sections stained with the Nissl method and for myelinated fibers. Individual imaging modalities were reconstructed into a three-dimensional form and then registered to the MR image by means of affine and deformable registration routines. Based on a superimposition of the 3D images, 113 anatomical structures were demarcated and the volumes of individual regions were measured. The stereotaxic coordinate system was defined using a set of cranial landmarks: interaural line, bregma, and lambda, which allows for easy expression of any location within the brain with respect to the skull. The atlas is released under the Creative Commons license and available through various digital atlasing web services.

**Electronic supplementary material:**

The online version of this article (10.1007/s00429-017-1540-x) contains supplementary material, which is available to authorized users.

## Introduction

To tackle the complexity of the vertebrate central nervous system and unveil the origins of its pathology and disease, various animal models are used in neuroscience. One of them is the gray short-tailed opossum (*Monodelphis domestica*) recently gaining recognition in biomedical research. It is a small (80–150 g) marsupial native to xeric regions of South America (Macrini 2004; Smith 2008). Laboratory animals are progeny of seven wild specimens that were first bred in 1978 (VandeBerg and Robinson 1997); this stock is named the laboratory or *Monodelphis* opossum.

The laboratory opossum has several characteristics making this species more useful in certain studies than the commonly used small eutherians, e.g. mice or rats. It is a non-seasonal breeder, with ovulation provoked by the presence of male (Macrini 2004). Numerous litters (4–14 pups) are born after very short (14 days) pregnancy, at an extremely early stage of development, equivalent to that found on the fetal day 11 in mice or the 6$\text {th}$ gestational week in human embryo (Saunders et al. 1989). For the first three postnatal weeks pups are permanently attached to mother’s teats, which facilitates experimental manipulations. Similar experiments on mice require cesarean section, resulting in substantially larger mortality of fetuses, stress and post-operation condition in mothers.

Therefore, the *Monodelphis* opossum is frequently used in developmental research (e.g., Puzzolo and Mallamaci 2010; Seelke et al. 2013; Bartkowska et al. 2013, 2014) as well as in comparative and evolutionary studies (Cheung et al. 2010; Dooley et al. 2013; Kaas 2004). The cytoarchitecture of its brain has been explored extensively, for instance, in the neocortex (e.g., Wong and Kaas 2009; Seelke et al. 2014) or in the olfactory bulb (Jia and Halpern 2004; Martinez-Marcos and Halpern 2006). Cytoarchitectonic study of the thalamus was performed by Olkowicz et al. (2008), while a partial description of putamen, caudate nucleus, and globus pallidus was provided by Domaradzka-Pytel et al. (2007). In addition, the *Monodelphis* opossum is also frequently used in biomedical studies, especially on melanoma (VandeBerg and Robinson 1997; Wang et al. 2009; Nair et al. 2014) or spinal cord injury treatment (Wheaton et al. 2015). Moreover, after sequencing its genome by Mikkelsen et al. (2007), the value of the opossum as a laboratory animal has increased noticeably.

Despite almost forty years of using the *Monodelphis* opossum in research, a comprehensive neuroanatomical reference for its brain is not yet available. Although its skull has been previously described (Rowe et al. 2005), no spatial relation between the cranium and the brain structures has been established, which is a practical limitation of the experimental procedures. Moreover, no high resolution structural MR imaging of the opossum brain has been reported. Therefore, there is a need for a standard reference tool, i.e. an atlas of the adult brain to better compare the results of different anatomical studies and to deepen the understanding of the brain structure and its function.

Brain atlases of various kinds of animals are among the most important neuroscientific tools. Their purpose is to organize brain-related data in a common reference framework. Brain atlases are created in various forms depending on their goals, and many are released as printed publications (Lanciego and Vázquez 2012; Paxinos et al. 2012; Ding et al. 2016; Radtke-Schuller et al. 2016). However, with the recent advancement in brain imaging (e.g., Axer et al. 2011; Osten and Margrie 2013) and computations (e.g., Lebenberg et al. 2010; Schubert et al. 2016), the atlases in printed form are being complemented with digital and three-dimensional counterparts (Amunts et al. 2013; Papp et al. 2014; Dai et al. 2017). Such tools offer flexibility which helps to address modern conceptual, computational, and analytical challenges. By combining imaging data from multiple techniques such as histology, MR, gene expression, connectivity data (Johnson et al. 2010; Oh et al. 2014; Zakiewicz et al. 2015) they have emerged as frameworks for data integration and exploration (Boline 2008; Sunkin et al. 2013).

In this spirit, we propose an atlas of the adult *Monodelphis* opossum brain. Combination of four complementary imaging modalities constituting the template made it possible to demarcate 113 anatomical structures. Additionally, its three-dimensional form allows for virtual cuts through the brain in arbitrary planes and for conducting various morphometric studies. The open nature of the atlas enables further development and expansion by researchers.

## Materials and methods

### Animals

The experiments were performed on two, one year-old (i.e. fully adult and not senile) males of the *Monodelphis domestica*, bred in the Nencki Institute Animal Care Facility (Warsaw, Poland). All procedures were approved by the Local Ethics Committee in Warsaw and they comply with the requirements of the Directive 2010/63/UE of the European Parlament and Council, as well as with its implementation into the Polish law. Body weights of the animals were 145.7 and 129.8 g. Both animals were overdosed with pentobarbital (100 mg/kg) via intraperitoneal injection and, when they ceased breathing, perfused transcardially first with saline (0.9% NaCl) followed by 4% paraformaldehyde (PFA) in phosphate buffer, pH 7.4. The opossums received a contrasting agent (ProHance, Bracco Diagnostics Inc.) with the PFA infusion (1:20, *v*:*v*). Subsequently, their heads were separated and cleaned of skin and muscles. Lower jaws were removed, eardrums perforated, and zygomatic arches were trimmed. Each specimen was placed in a 50 ml centrifuge tube filled with the solution of 4% PFA and ProHance. One was post-fixed in this solution in a temperature of 4$\,^\circ$C and underwent magnetic resonance imaging twice: 48 h and 1 month after the perfusion. The second specimen was post-fixed for one week and then underwent micro-computed tomography (micro-CT) imaging.

### Magnetic resonance imaging

The magnetic resonance imaging was performed with a 9.4 T Bruker Biospec®90/20 MRI System (Bruker BioSpin MRI GmbH, Ettlingen), equipped with B-GA 12S gradient coils (116 mm inner diameter and 675 mT/m maximum gradient strength) and 35 mm inner diameter birdcage RF coil. The imaging was conducted on the heavier of the specimens with the brain left in the cranium resulting in the 48 h ex-vivo scan. A T1/T2* weighted 3D FLASH sequence with scan parameters optimized for the contrast between gray and white matter was used, i.e. repetition/echo time (TR/TE): 9/4.5 ms, flip angle: 20$^{\circ }$, field of view (FOV): 19 $\times$ 25.6 $\times$ 22 mm (mediolateral, rostrocaudal and dorsoventral, respectively), image matrix (MTX): 380 $\times$ 512 $\times$ 440, number of repetitions (NEX): 35. The sequence took approximately 16 h to complete. Afterwards, the sample was stored in the fixative for additional 28 days and the imaging was repeated with the same settings, yielding the 30-days ex-vivo scan.

### Cryosectioning and staining

**Fig. 1:**
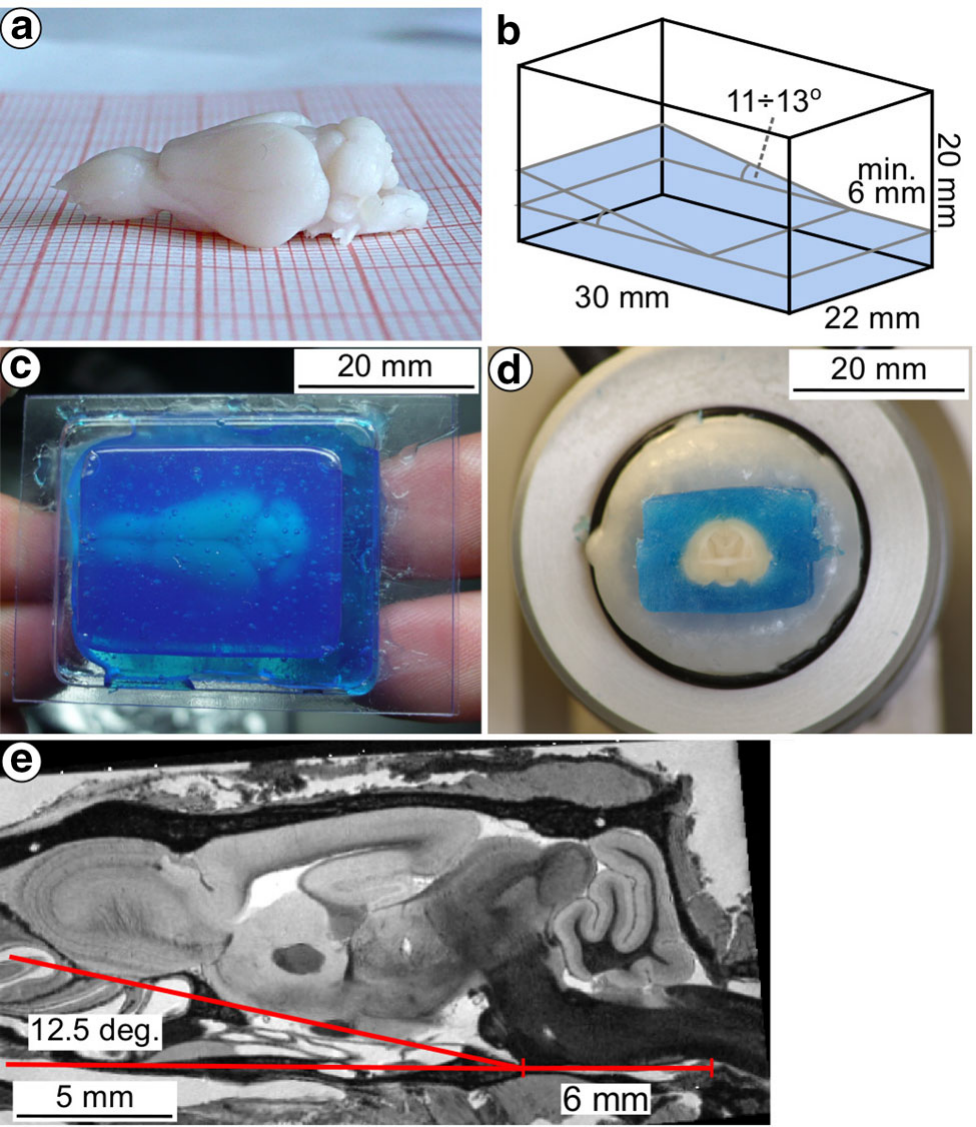
Consecutive steps of preparing and cryosectioning the specimen. **a** Fixed brain extracted from cranium; **b** Cryomold with the base formed of congealed colored Cryogel; **c** brain embedded in the Cryogel in the Cryomold; **d** example blockface photography (no. 228 out of 528); **e** parasagittal cross-section (1 mm lateral from the midsagittal plane) through the 30 days MR image illustrating the rationale behind the stereotactic orientation and the shape of the cryomold. The angle of inclination of the base of the brain (marked with the red line) is the same as in the cryomold; the ventral surfaces of the pons and the brainstem are oriented horizontally and extend for at least 6 mm

After the MR imaging, the brain was extracted from the cranium (Fig. 1a), weighted (1.009 g), and cryoprotected by soaking for 3 days in sucrose solutions of increasing concentrations (10, 20 and 30%; 24 h each). Afterwards, the brain was snap-frozen in $-\,70\,^{\circ }$C isopentane and placed in a Leica CM1850 cryostat at $-\,20\,^{\circ }$C. In order to preserve the coronal plane of the sectioning, a plastic Cryomold^®^ Standard, $25 \times 20 \times 5$ mm mold was used (Fig. 1b). The Cryomold was filled with a Cryogel (Instumedics Inc.), stained with 0.2% blue ink to approx. 1/3 of its height, placed on a wedge angled at $12 \pm 1^{\circ }$ and left in the cryostat until it started to freeze. At this inclination, the ventral surfaces of the pons and the brainstem were oriented horizontally (Fig. 1e, and Fig. S6 in supplementary materials), and the bregma and lambda cranial landmarks lay on the same horizontal level as well (see Sect. “Stereotaxic coordinate system”). Once the Cryogel was mildly frozen, the brain was placed on its surface aligned with the mold. After about 5 min the Cryomold was further filled with colored Cryogel to a level of 5–6 mm above the top of the brain (Fig. 1c) and placed in a refrigerator ($-\,20\,^{\circ }$C) for 24 h. Afterwards, it was cut into 40 $\upmu$m thick coronal sections at a $-\,20\,^{\circ }$C temperature.

Before cutting each section, an image of the face of the cutting block (a blockface image, Fig. 1d) was taken with a digital camera (Canon 60D with a Sigma 70–300 mm telephoto lens) mounted on a tripod in front of the cryostat. A total of 528 blockface photos were captured with a resolution of 16.9 $\upmu$m/pixel.

The cut sections were collected into two alternating series. To limit slice distortion, before cutting each section, a piece of Adhesive Tape Windows (Leica Microsystems Inc.; Nissanov et al. 2001) was taped to the cutting block face. Each section was individually transferred to a gelatinized slide with the tape attached. After drying for 1 h on a Slide Warmer plate (40$\,^{\circ }$C), the Tape Windows were peeled off the slides. Then the sections were stained directly on the glass slides. The first series of 264 sections was stained with the standard Nissl method, and the second with the Gallyas (1971) stain for myelinated fibers. No section was lost in the process of cutting and staining, however, a few were distorted (see Sect. “Data preprocessing” below). Finally, the stained sections were digitized using an Epson Perfection V700 flatbed scanner with a resolution of 3200 DPI (approx. 5.8 $\upmu$m/pixel).

### Micro-computed tomography imaging

Micro-CT imaging of the second opossum’s cranium was conducted on an X-Tek (Nikon) Benchtop CT160Xi microtomograph equipped with a 3 mega pixel Varian PaxScan 2520V flat panel detector with a pixel area of $127 \times 127$ $\upmu$m$^2$ (array size of $1916 \times 1536$ pixels). The sample within the centrifuge tube was placed in the microtomograph’s chamber and 1428 projections were captured (0.25$^{\circ }$ of rotation between each projection). The exposure time was set to 1 s and a single frame was averaged from 2 exposures in order to increase signal to noise ratio. The imaging sequence took 134 minutes and resulted in a 3D image of 41 $\upmu$m/voxel isotropic resolution.

### Computational environment

The computations required to create the atlas involved an array of open source software for biomedical image processing and visualization. The reconstruction of three-dimensional brain images based on the series of sections was carried out using the Possum framework (http://github.com/pmajka/poSSum, Majka and Wójcik 2016) which enables one to perform versatile reconstruction tasks without the need for low-level implementation. To conduct 3D to 3D image registration, we used the Advanced Normalization Tools (ANTS) software suite (http://picsl.upenn.edu/software/ants/, Avants et al. 2011a). The ANTS allows one to compute affine transformations and deformable mappings using the Symmetric Normalisation algorithm (SyN, Avants et al. 2008) driven by one or more similarity terms. In addition, the InsightToolkit (ITK, Schroeder 2005, http://www.itk.org/), Convert3d (2006b and ImageMagick (http://www.imagemagick.org/) software suites were used to accomplish auxiliary image processing tasks, such as conversion between formats, various kinds of filtering, resampling, cropping, etc. Three-dimensional visualizations were created using the Visualization Toolkit system (Schroeder et al. 2006, http://www.vtk.org/). The calculations were performed under an Ubuntu 12.04 operating system deployed on a dual Intel^®^ Xeon^®^ E5520 CPU (16 $\times$ 2.27 GHz logical processors) workstation equipped with a 48 GB of memory.

### Data preprocessing

Before the three-dimensional reconstruction and coregistration of the imaging data from multiple modalities, several preprocessing steps were conducted.

The first stage was to create brain tissue masks (i.e. to erase all elements of the MR images which did not depict brain tissue, such as muscles, cranium, large blood vessels or cranial nerves, etc.). For that purpose, Atropos Markov random field segmentation (Avants et al. 2011b) was applied which provided four, KMeans-initialized, classes of tissue labels which were manually refined into a brain mask using an open source ITK-SNAP 2.4 program (https://itksnap.org, Yushkevich et al. 2006b).

Afterwards, the digitized histology sections were subjected to quality control to identify those not suitable for 3D reconstruction due to tears, folds, excessive distortion or shrinkage, etc. Images of such sections were replaced with those of their immediate undistorted neighbors. In the Nissl-stained series 24 out of 264 sections were replaced (9.1%), in the myelin-stained sections—40 out of 264 (15%). Subsequently, brain tissue masks in images of individual stained sections were created. The red color channel of the images was extracted and smoothed by a median filter with $5 \times 5$ pixel kernel, thresholded at 94.8% of the maximum image intensity and the largest component was extracted. Finally, the slices’ masks were corrected manually. Moreover, for the Nissl-stained sections, a slice-to-slice staining intensity correction was performed based on the weighted histogram matching approach proposed by Li et al. (2009).

In order to create a coherent three-dimensional image of the unstained brain before sectioning, consecutive blockface images were stacked and then aligned to compensate for the jitter of the position of the cryostat head. The alignment was performed using the naive propagation-based approach (p. 327, Kiessling 2011) implemented in the Possum software. Section no. 250 was used as a reference and rigid transformations between consecutive images were serially propagated towards either end of the stack. Finally, the stack was downsampled and 3D unsharp mask filter (Gonzalez and Woods 2007) was applied to increase the tissue contrast. This resulted in a 480 $\times$ 527 $\times$ 350 voxels (mediolateral, rostrocaudal and dorsoventral, respectively) RGB image with an isotropic resolution of 40 $\upmu$m/voxel.

### Three-dimensional reconstruction and coregistration

**Fig. 2:**
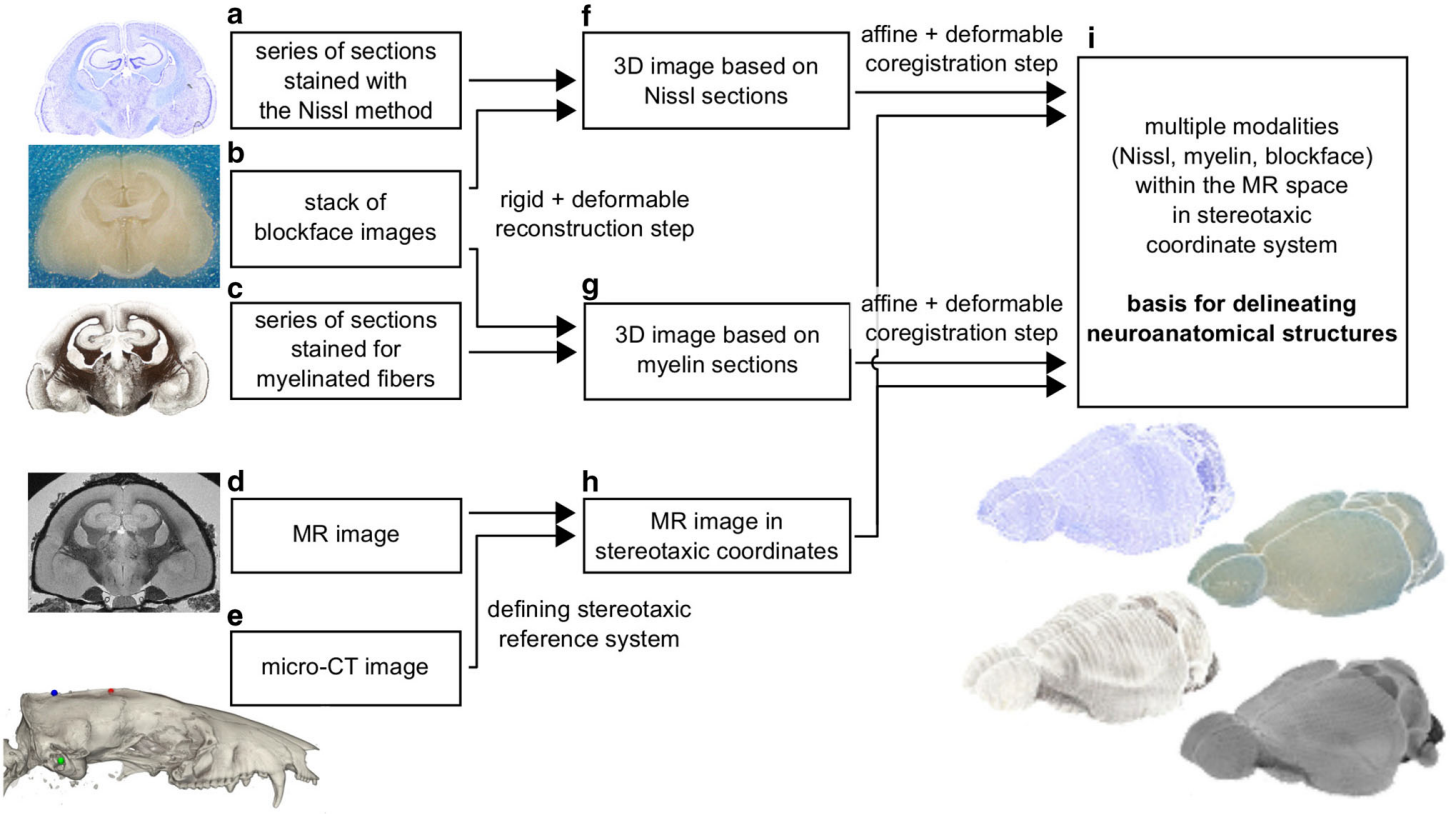
Diagram of the data integration workflow. The blocks **a**–**i** represent individual datasets while the arrows indicate direction of the data flow. The inscriptions next to the arrows describe consecutive processing steps

The reconstruction of the three-dimensional brain images from the serial sections and further coregistration with the MR imaging data was accomplished according to the scheme presented in Fig. 2. The input for the workflow comprised the acquired data, namely: the series of sections stained with the Nissl method (Fig. 2a) and for myelinated fibres (Fig. 2c), the 3D blockface image (Fig. 2b), the MR image (Fig. 2d), and the 3D skull image obtained via the micro-CT imaging (Fig. 2e). The integration was initiated by a reconstruction of the 3D brain image from the serial sections (Fig. 2f, g), performed in two steps: rigid and deformable. Afterwards, the MR image was aligned to the micro-CT scan (Fig. 2h). Subsequently, the reconstructed 3D histological images (Fig. 2f, g) and the 3D blockface image were affinely aligned to the reference MR image. Next, the 3D histological reconstructions were additionally registered to the MR image using deformable transformations, forming the final template (Fig. 2i) which was ultimately used as a basis for delineation of the brain structures.

In order to perform the affine reconstruction step, we utilized a modified coarse-to-fine method proposed by Yushkevich et al. (2006a), implemented in the Possum software. This approach relies on calculating and then combining two series of rigid transformations: *coarse* and *fine*. It is known to, both, preserve faithful anatomical shape of the reconstructed 3D brain image by mitigating the Z-shift, also called the *banana effect* (see e.g., Malandain et al. 2004), and to provide coherence between consecutive sections. The coarse-scale alignment involved the blockface image stack which is a spatially consistent 3D image of the brain just before sectioning. Since every stained section corresponds to a specific blockface image, to recover the anatomical shape of the brain, images of individual stained sections were aligned to their blockface counterparts yielding a series of coarse-scale transformations.

The fine-scale reconstruction was performed by sequentially aligning the sections starting with those located centrally in the image stack (sections no. 160 and 150 for the Nissl- and the myelin-stained sections, respectively) towards either end of the stack. Red color channels of both image stacks were extracted and smoothed with a 5 $\times$ 5 pixels median filter and the registration was performed with the cross-correlation (CC) as an image similarity metric. This resulted in a series of fine-scale rigid transformations.

Finally, the transformations obtained in both steps were merged by combining the high-frequency component of the fine-scale transformation with the coarse-scale alignment. This was done by Gaussian smoothing ($\sigma =$ 5 sections) of individual parameters of the fine-scale transformation (translation and rotation angle) across the *z* (stack) dimension, filtering them out, and combining with the parameters of the coarse-scale registration. The image stack was then transformed accordingly and passed to the deformable reconstruction routine.

The approach to the nonlinear refinement of the 3D histological image stems from an assumption that the change of the shape of the brain structure takes place in larger spatial scales than the section thickness itself, and thus the neighboring images are similar to one another in a formal sense (Adler et al. 2014; Gaffling et al. 2014). Therefore, the elementary stage of this refinement relies on warping a given section image towards an average image of its nearest neighbors sections in either direction. Such warping is performed for all images in the stack which constitutes a single iteration of the procedure. Many iterations have to be applied in order to obtain the final reconstruction. Registration parameters (similarity metric, amount of deformation, regularization, etc.) depend on a particular iteration.

The method was applied to both stacks of images (Nissl and myelin), 264 sections each. In the initial step of compensating highly deformed sections, various ANTS software parameters values were used: The gradient step for the SyN transformation varied from 0.15 to 0.5. The cross-correlation image similarity metric kernel size was either 4 or 16. A Gaussian regularization term with a sigma of 3 voxels for the similarity gradient, and 1 voxel for the displacement field was used. Then, 18 iterations for the Nissl-stained sections, and 17 iterations for the myelin-stained sections, were carried out with parameters detailed in Tables S1 and S2, respectively. Eventually, the computed displacement fields were applied to the corresponding sections yielding 3D brain images which, in the following step, were coregistered with the MR image.

The purpose of the last step was to bring individual 3D reconstruction into a common spatial reference frame of the MR image. First, an affine coregistration was performed. It relied on aligning the masks of the MR image and the mask of the blockface stack using the summed squared distance (SSD) as a similarity metric. Since the 3D blockface image was used as the reference for the reconstruction of the 3D brain images based on the serials sections, the computed transformation is also applicable to these reconstructions.

Following the affine alignment, a deformable coregistration of histological reconstructions towards the MR image was carried out. Three similarity terms (three pairs of fixed/moving images) were used to drive the coregistration: (1) a pair of intensity (grayscale) images, (2) a pair of binary masks of the brain tissue, and (3) segmentations of the images being coregistered. The moving intensity images were the red image channels for the histological reconstructions and the grayscale MR image was used as the fixed image. The binary brain masks of MR and histological reconstructions constituted the second set of images. The last set comprised the segmentations of the MR image and histological reconstructions. Their role was to provide an expert knowledge on corresponding brain regions to increase the coregistration accuracy. Ten structures were delineated in all three images for that purpose: (1) olfactory bulb white matter; (2) external plexiform layer of the olfactory bulb; (3) granular cell layer of the olfactory bulb; (4) cerebral cortex (isocortex and piriform cortex combined); (5) dentate gyrus of the hippocampal formation; (6, 7) left and right sides of the brainstem; (8) periaqueductal white matter; (9) hippocampus; (10) cerebellum. Two additional structures were delineated only in the Nissl-stained and MR images: (11) layer II of the piriform cortex; (12) anterior commisure and collateral fibres. Note that these delineations were established for the sole purpose of improving the coregistration and were independent from the neuroanatomical delineation described further in this article.

Following preparation of the images, ten registration steps for each histological reconstruction were carried out. The cross-correlation image similarity metric kernel size was set to 5 voxels, regularization of the displacement field kernel radius varied from 0.1 to 0.05 mm, no smoothing of the velocity field was used, and the ANTS point set estimation (PSE) metric was used with the default settings. Detailed specification of the parameters applied in individual coregistration steps may be found in Table S3. Finally, the calculated deformation fields were applied to the corresponding images bringing the Nissl- and myelin-based 3D brain images into register with the MR image.

### Evaluation of the coregistration accuracy

To assess the accuracy of the affine and the deformable coregistration, 100 landmarks were selected uniformly over the whole brain within each of the coregistered 3D images (Nissl-stain reconstruction, myelin-stain reconstruction, and MR image). The corresponding points were located manually on each image after the affine coregistration. Discrepancy between homologous landmarks was measured by calculating Euclidean distance between corresponding points yielding a discrepancy measure of the affine coregistration. Then, the landmarks’ locations were transformed using the deformation fields computed during the deformable coregistration process and the discrepancies were calculated again.

### Stereotaxic coordinate system

The stereotaxic coordinate system is based on the cranial landmarks identified on the micro-CT scan of the skull as the MR modality did not provide a satisfactory basis (see Fig. S6 in supplementary materials). The cranial sutures in the opossum skull are less pronounced than in rodents and they are additionally obscured by the gristles and sinews. Moreover, the MR protocol, fine-tuned to highlight the brain itself, yielded insufficient signal to noise ratio in the skull, while the micro-CT scan offered an excellent contrast. Therefore, using such an image, even from a different individual, significantly facilitated establishing the stereotaxic reference system.

The two chosen cranial landmarks were the bregma and the lambda. The bregma was defined as the intersection point of the sagittal and coronal sutures, the lambda—as the connection point of the sagittal and lambdoid sutures. Moreover, the course of the interaural line was defined as an additional reference. Afterwards, the micro-CT image was reoriented so the lambda and bregma points lay in the same horizontal and medial planes. Finally, distances between the established landmarks were measured.

In order to align the brain with the skull, a mask of the braincase was drawn on the micro-CT image. Then, the mask of the brain, from MR image, was registered to the mask of the braincase using a rigid transformation. Since the micro-CT scan of the skull was acquired from a different specimen than the brain images, and the braincase is slightly larger than the brain itself, minor manual adjustments were required to precisely match the midplane and assure flat orientation of the base of the brainstem.

### Delineation of the anatomical structures

The process of delineation of the anatomical structures within the opossum brain was conducted using the ITK-SNAP software. Both MR images (48 h and 30 days post-fixation) were displayed in grayscale, while the Nissl- and the myelin-based reconstructions were viewed in RGB mode. All neuroanatomical delineations were performed manually in all three cardinal planes. Since the 30 days MR image had the least amount of imperfections and higher contrast, it was chosen as a reference modality. Therefore, the brain structures were outlined by superimposing the 48 h MR image, reconstructions of the Nissl- and the myelin-stained sections, onto the 30 days MR image.

To ensure a proper identification of individual regions (structures, nuclei, cell layers, or fibres) we used the cyto- and the myeloarchitecture to determine the borders and the location with respect to other regions. A region was delineated when its outline was distinguishable, in its majority, on at least one of the modalities. When, occasionally, the borders were partially indiscernible due to a lack of microscopic details, the physical sections were inspected directly under a Nikon Eclipse 90i microscope (4 $\times$ or 10 $\times$ Plan Fluor objective) to precisely resolve the boundaries. Sporadically, high-resolution microphotographs were taken for future reference (see Fig. S7 in supplementary materials). We refrained from delineating individual nuclei which could be subdivided only arbitrarily or regions with gradual transitions to its neighboring structures.

The other criterion for identification was the spatial location with respect to other structures. In phylogenetically old parts of the brain (midbrain, hindbrain, pons, medula oblongata, etc.), where individual structures are arranged similarly to their homologues in rodents, the identification was guided by atlases of, both, rodents (Paxinos and Watson 2007; Paxinos and Franklin 2008; Swanson 2004), and marsupials (Oswaldo-Cruz and Rocha-Miranda 1968; Ashwell 2010). In regions, where the marsupial brain diverges from rodent’s, we used the atlas of Oswaldo-Cruz and Rocha-Miranda (1968) as well as Olkowicz et al. (2008) and Ashwell (2010) to establish the relative location of the structures.

As there was no brain atlas of *Monodelphis* opossum, the employed nomenclature, in its majority, was based on the recent editions of the atlases of the rodent brain, in particular Paxinos and Watson (2007), Paxinos and Franklin (2008) and Swanson (2004). The nomenclature and partitions of the atlas of *Didelphis marsupialis* opossum brain (Oswaldo-Cruz and Rocha-Miranda 1968), although concerning the closest relative of the *Monodelphis*, were taken with reservation, as this atlas is outdated in some parts.

## Results

### Reconstruction and coregistration

**Fig. 3:**
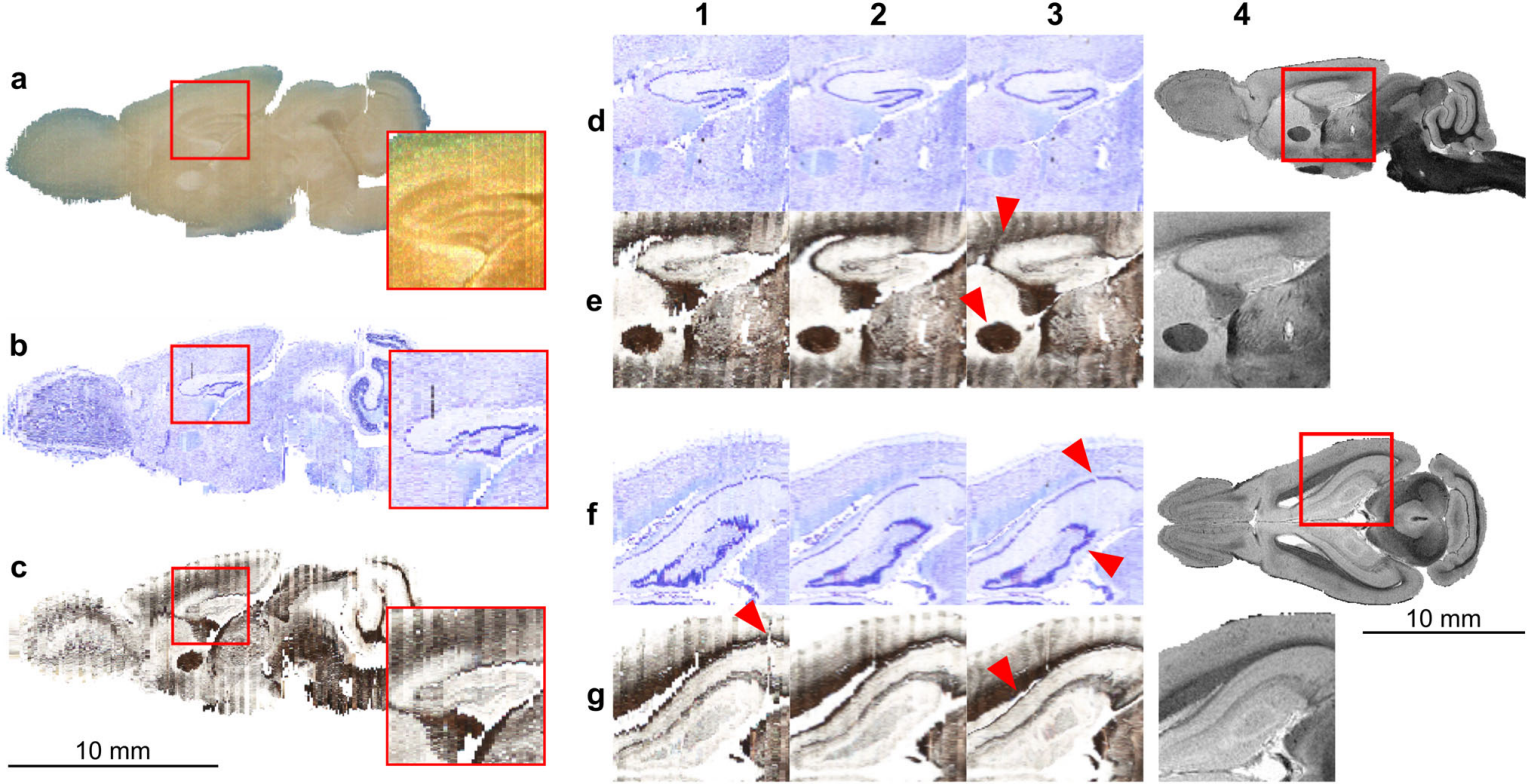
Consecutive steps of the 3D reconstruction process. **a**–**c** Comparison of the results of the rigid reconstruction step, sagittal-cross sections through the 3D image. Note that the brain was cryosectioned in the coronal plane. **a** Blockface image stack; **b** Nissl- and **c** myelin modality. Red rectangles indicate the area of the dentate gyrus magnified in the insets. Rows** d**–**g** show closeups of the sagittal (rows** d** and** e**) and horizontal (rows** f** and** g**) cuts through the reconstructions at various stages. Columns 1–3 depict the reconstruction after (1) rigid reconstruction; (2) deformable reconstruction; (3) deformable registration to the reference MR image (shown to the right). Rows **d** and **f** present reconstruction based on the Nissl-stained sections while rows **e** and **g** are based on the myelin-stained sections. For the description of the features indicated by the red triangles in the panels** d**–**g** see Sect. “Reconstruction and coregistration”

Figure 3 summarizes the consecutive steps of the reconstruction and the coregistration of the histological sections to the MR image. The rigid reconstruction (Fig. 3a–c and column 1) transformed the stacks of the Nissl- and myelin-stained sections so they match the blockface image stack and therefore recovered the overall, anatomical shape of the brain. In addition, this step allowed us to recreate even thin cell layers, e.g. individual cell layers in the olfactory bulb or in the dentate gyrus (insets on the Fig. 3a–c). However, the resulting reconstructions contained a lot of crisp edges and suffered from section-specific artefacts (e.g. red triangle in Fig. 3, 1g). Subsequent deformable reconstruction (Fig. 3, column 2) eliminated the majority of section-specific deformations leading to smooth, continuous reconstructions of the 3D histological images, significantly improving their visual quality. This is especially noticeable when inspecting thin cell layers (e.g. dentate gyrus, cortical layers) and the overall brain outline (Fig. 3; column 2d–g). The ultimate deformable coregistration (Fig. 3, column 3) compensated for the distortions due to the cutting and staining processes, e.g. excessive shrinkage, in particular close to the ventricles (compare Fig. 3, columns 2g and 3g), and further improved similarity to the reference MR image (Fig. 3, column 4). Additional examples of the reconstructions during consecutive processing steps are available in Figures S1 to S4 in the supplementary materials.

### Evaluation of the registration accuracy

**Fig. 4:**
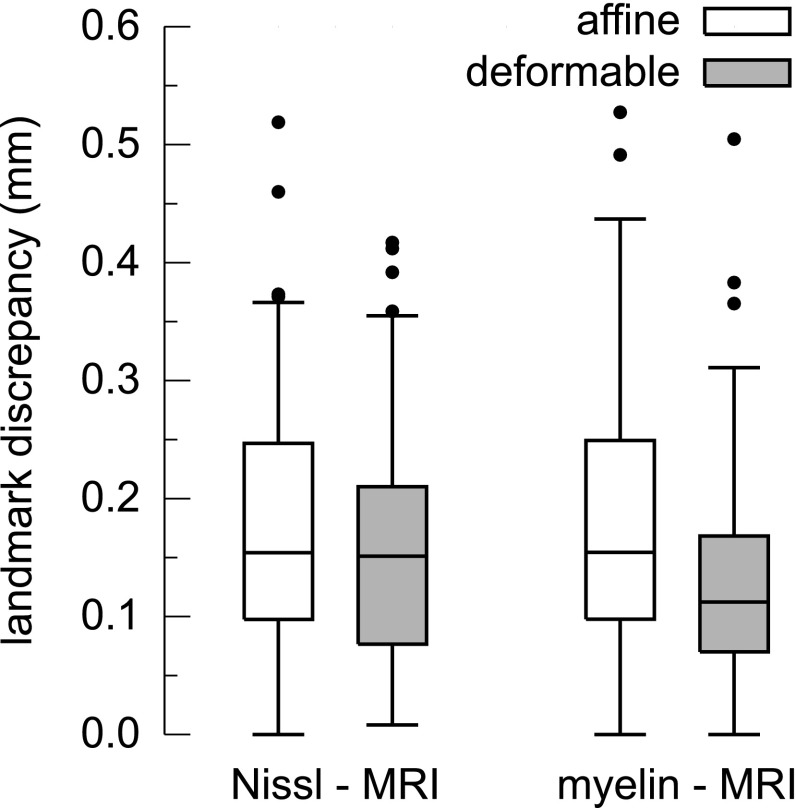
Comparison of the distances between landmarks before (white bars) and after (gray bars) deformable coregistration step. Box and whisker plots showing order statistics (5% percentile, lower quartile, median, upper quartile and 95% percentile) of the distribution of the distances between corresponding landmarks

Comparison of the distances between the landmarks is presented in Fig. 4. For the Nissl to MR affine coregistration, the discrepancy of 75% landmarks was less than 0.25 mm (5 times the voxel size), for 95% less than 0.36 mm (7.2 times the voxel size). The median value was 0.15 mm (3 times the voxel size). After the deformable registration, the discrepancy was slightly lower: the median remained at the same level, however, the upper quartile decreased to 0.21 mm and the 95th centile decreased to 0.35 mm.

For the myelin-stained volume, the discrepancy between the landmarks after the affine coregistration was comparable with that of the Nissl-stain. The median value was 0.16 mm (3 times the voxel size), the third quartile was 0.25 mm and the 95th centile—0.4 mm. After the deformable registration step, the median of discrepancy distribution lowered noticeably to 0.11 mm (2.2 times the voxel size), 75% values were below 0.17 mm and the 95th centile decreased to 0.31 mm (6.2 times the voxel size).

### Delineation of the neuroanatomical structures

**Fig. 5:**
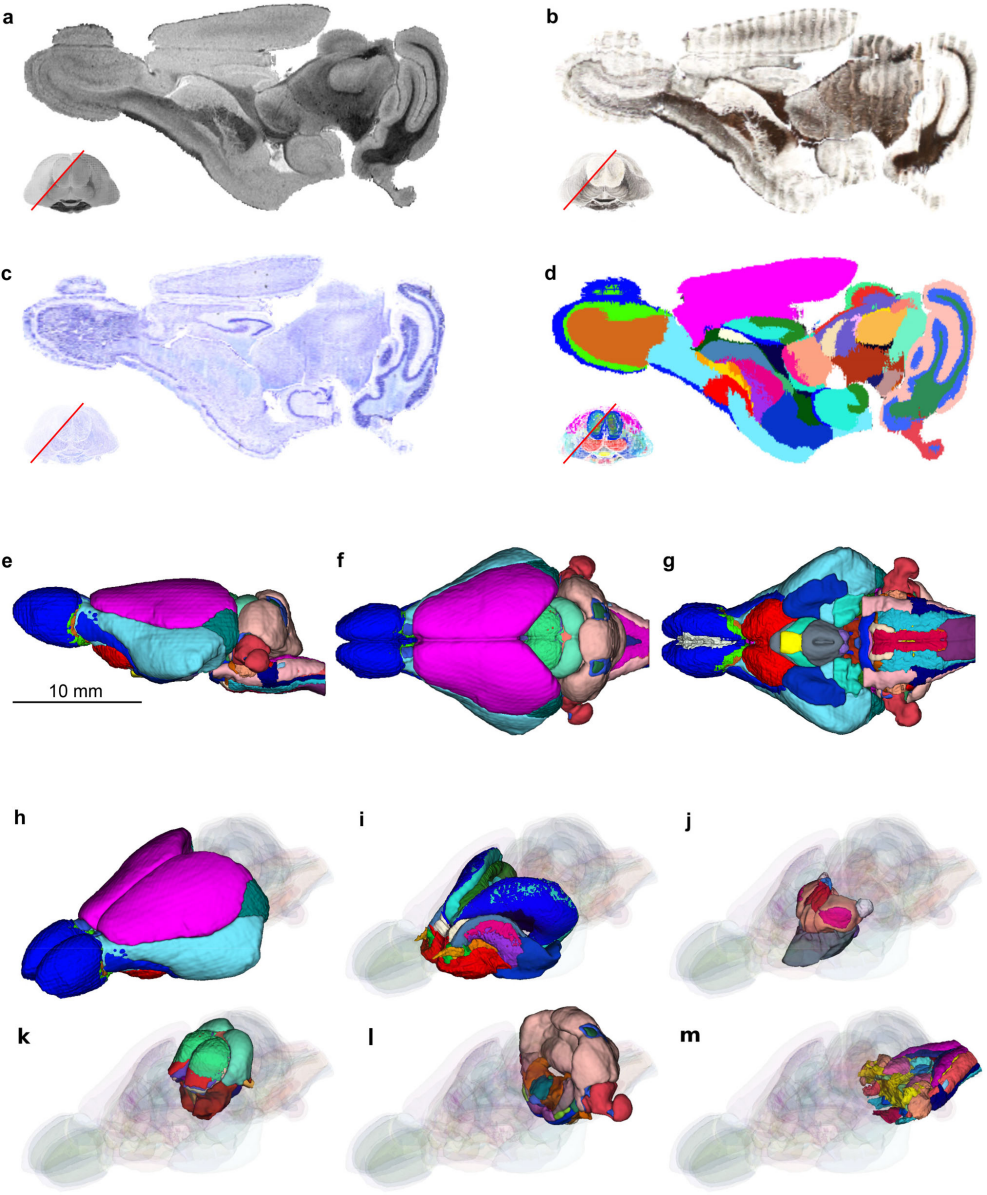
Oblique cross-section of the brain template (**a**–**d**) and 3D visualisations of the delineated brain structures (**e**–**m**). **a** 30 day MR image, **b** myelin modality, **c** Nissl modality and **d** segmentation into the brain regions. 3D visualisations of the structures in **e** sagittal, **f** dorsal and **g** ventral projections. Regions of the: **h**, **i** forebrain, **j** diencephalon, **k** midbrain, **l** hindbrain and **m** medulla oblongata. The full list of the demarcated structures is shown in Fig. 6

**Fig. 6:**
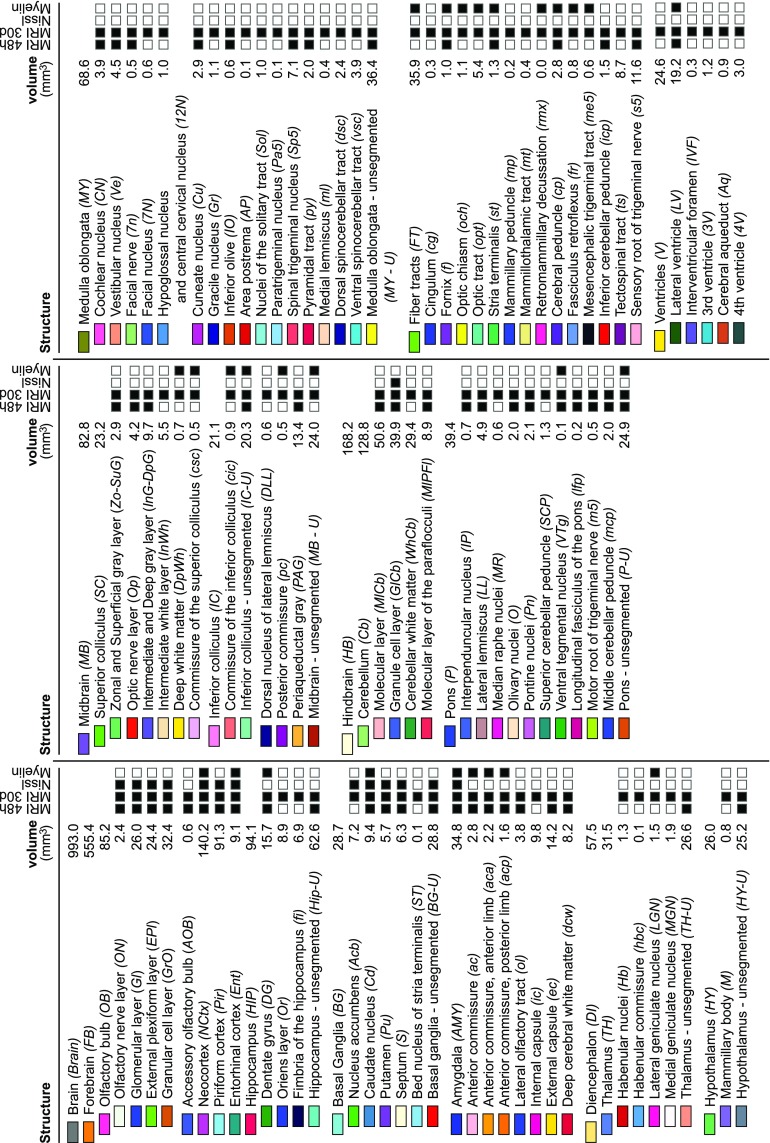
A hierarchical list of the delineated structures with abbreviations and their volumes within the opossum brain. Black squares depict imaging modalities used in the delineation or identification of specific structures whereas blank squares indicate modalities which were not utilised in the segmentation of a particular region

Based on the MR images and the three-dimensional reconstructions we demarcated 113 neuroanatomical structures within the opossum brain (Fig. 5) and measured their volumes (Fig. 6). The majority of the delineations was based on the MR image of the brain after 30 days of post-fixation, chosen as the reference modality for the atlas while other modalities superimposed onto this image aided in resolving ambiguities between regions.

The different times of post-fixation (48 h and 30 days) resulted in different highlighting of various brain structures. In the 48 h fixed brain MR image the outer brain parts were particularly well differentiated. Granule cells layers in the olfactory bulb (*GrO*) and cerebellum (*GlCb*) were hypointense, while other layers of these structures appeared hyperintense. Differences in the observed MR signal intensity and colours of the stained brain tissue were used to draw boundaries between the granule and other cell layers in these structures. The thick monolayers of cells in the piriform cortex (*Pir*), the hippocampus (*HIP*) and the dentate gyrus (*DG*) were visible as bright lines on otherwise homogeneously, myelinated brain regions of low MR signal intensity. Thick axon bundles, like anterior commissure (*ac*) with the anterior (*aca*) and posterior (*acp*) parts, internal capsule (*ic*), sensory root of the trigeminal nerve (*s5*) or the optic tract (*opt*) were noticeably darker in comparison with light gray appearance of the surrounding regions. Other brain structures were less outstanding in the 48 h fixed MR image, nevertheless, combined with the 30 days MR image, it provided information helpful in localisation of several brain structures (see Fig. 5).

The myelin-based reconstruction was used frequently in conjunction with the MR image of the brain fixed for 30 days to identify myelinated axons in brain structures (Fig. 6). The regions stained dark brown indicate a higher proportion of myelinated axons (e.g. anterior limb of the anterior commissure—*aca*, optic tract—*opt*), while the light brown areas represent structures with smaller concentration of the myelinated fibers (e.g. anterior commissure—*ac*, external capsule—*ec*). On the other hand, the areas with minimal content of myelinated axons lacked the brown tint (e.g. caudate nucleus—*Cd*, dentate gyrus—*DG*).

The Nissl-stained sections reflected different density of cell bodies. This allowed for identification and outlining of several nuclei, such as the nucleus accumbens (*Acb*), septum (*S*), caudate nucleus (*Cd*), putamen (*Pu*), and cellular layers in certain structures, e.g. the olfactory bulb (glomerular layer—*Gl*, external plexiform layer—*Epl*, granular cell layer—*GrO*), and others (see Fig. 6).

### Stereotaxic reference system

**Fig. 7:**
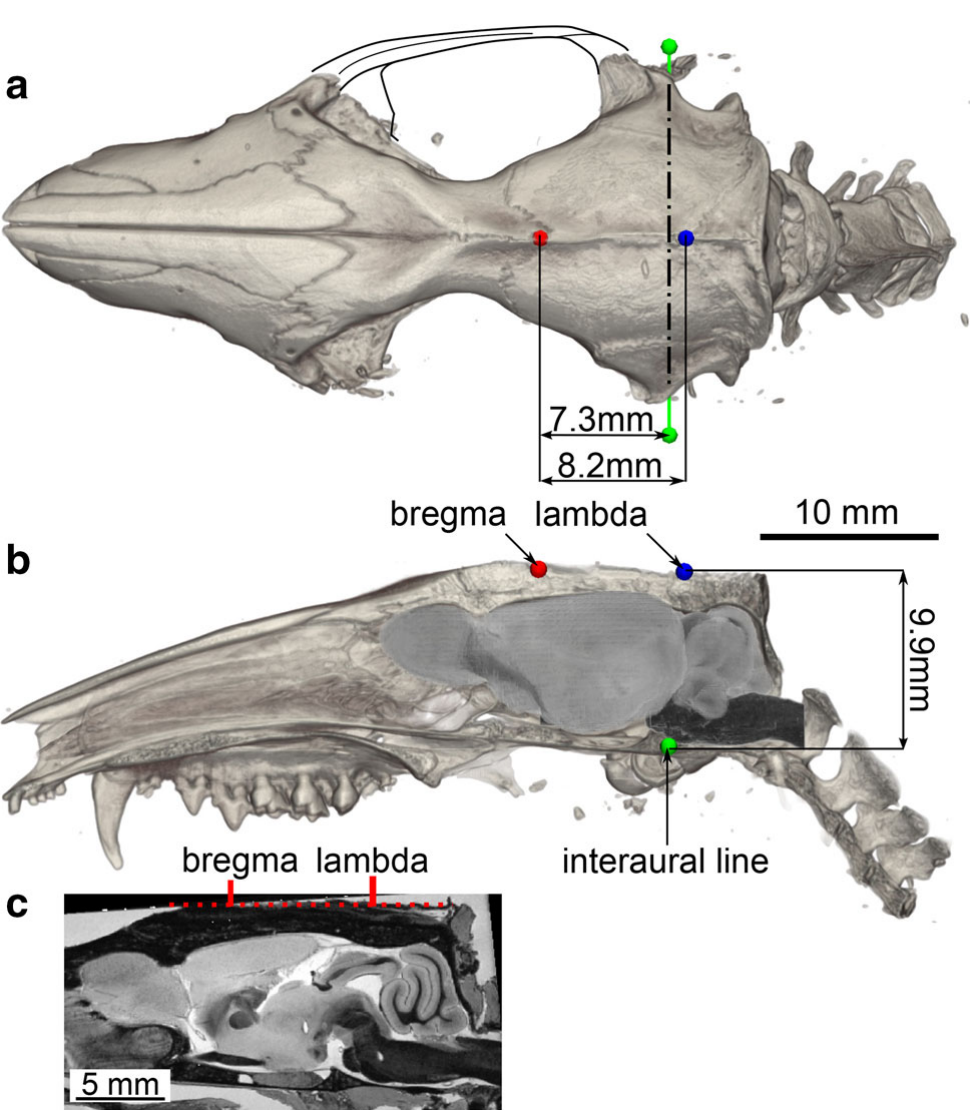
Stereotaxic coordinate system based on the micro-CT imaging of the skull. The color points denote the established landmarks: the bregma (red), the lambda (blue) and the course of the interaural line (green). **a** Horizontal projection; the right zygomatic arch, trimmed during preparative procedures, was drawn manually; **b** sagittal projection; the skull was cut along the midsagittal plane and the MR image aligned to the braincase is shown; **c** midsagittal cross-section through the MR image. The arrows point at the cranial landmarks while the dotted line indicates that both landmarks lie in the same horizontal plane. See Fig. S6 in supplementary materials for an extended version of the figure

Figure 7shows the opossum skull obtained with the micro-CT imaging along with the established landmarks: the bregma, the lambda, and the course of the interaural line. Introduction of stereotaxic alignment system similar to that used in rodents is not recommended: the angled shape of the opossum’s external ear canals (Fig. 7a and Fig. S5D in supplementary materials), thin bones of the base of the skull, delicate nasal bones, and narrow dental diastema, significantly reduce reliability of this approach. In our method, when we incline the brain by 11$^{\circ }$ –13$^{\circ }$ lifting up the rostral part, we bring both landmarks to the same level (Fig. 7b, c). Then, the bregma point is located above the prefrontal cortex, while the lambda is above the border between the superior and the inferior colliculi. The interaural line passes anterior to the brainstem, between the pineal gland and the pontine nuclei. The distance between bregma and lambda is 8.2 mm while the horizontal distance from bregma to the projection of interaural line is 7.3 mm anteriorly, and lambda is placed additional 0.9 mm posteriorly. The horizontal plane which includes bregma and lambda is located 9.9 mm dorsally to the interaural line.

Within the established reference frame, the investigated brain spans 14.4 mm along the perpendicular (left–right) axis. It has a total length of 22.3 mm along the longitudinal axis, from the beginning of the olfactory bulb to the posterior-most fragment of the cerebellum, and it extends 8.8 mm in the dorso-ventral direction from 1.0 to 9.8 mm ventrally from lambda.

**Fig. 8:**
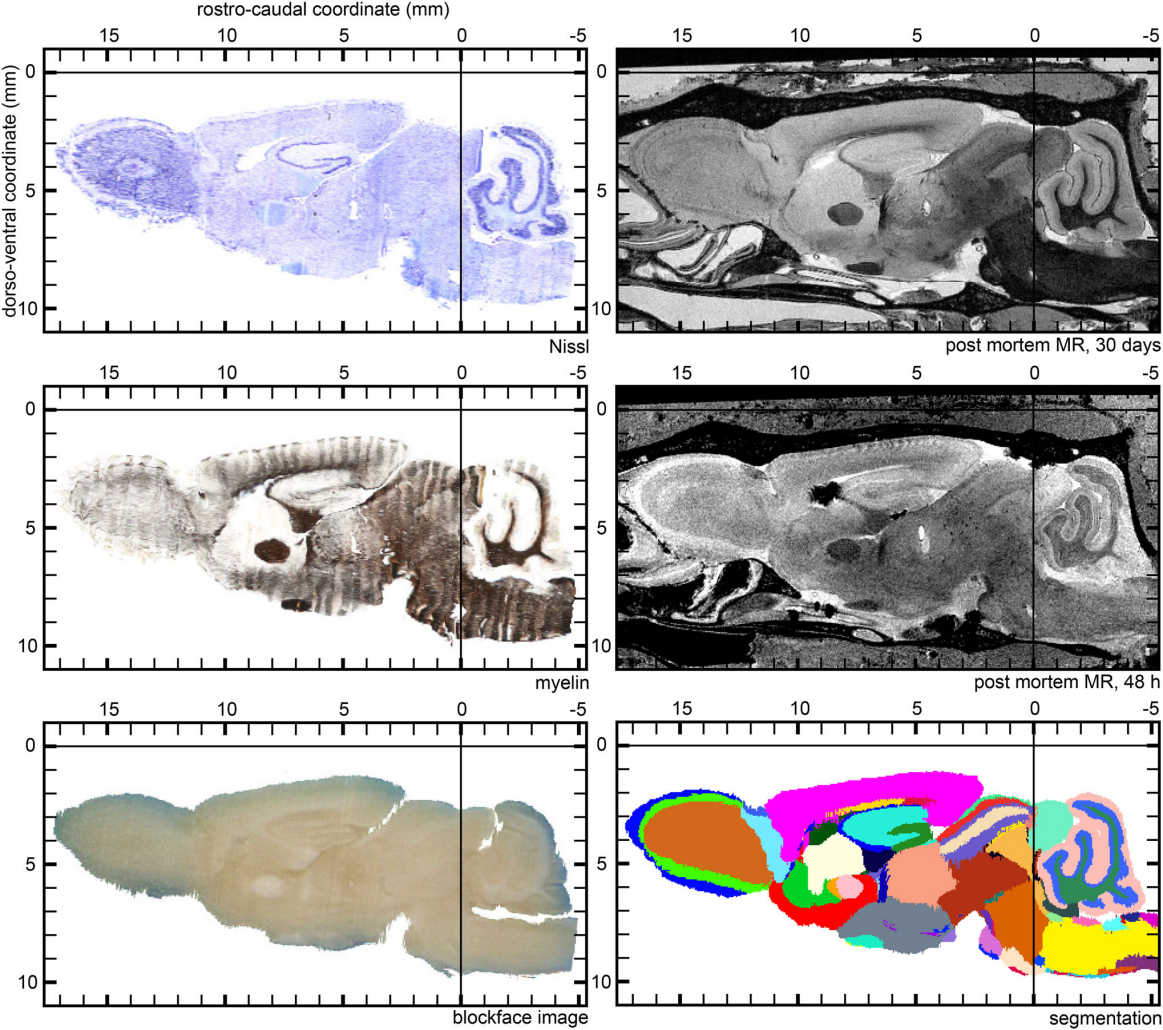
Example sagittal cross-sections (1.0 mm lateral from the medial plane) illustrating the atlas. Individual imaging modalities coregistered to a common stereotaxic reference frame: 3D reconstruction based on Nissl- and myelin-stained sections, 3D blockface image, MR images and the segmentation of the brain into individual anatomical structures. Full list of delineated regions is shown in Fig. 6. The complete atlas in a form of printable series of cross-sections and in a fully three-dimensional form is provided as supplementary materials

Upon establishing the stereotaxic coordinate system, imaging data from all modalities can be expressed within this common reference frame and the atlas can be browsed and visualized in three dimensions (Fig. 5). For instance, oblique cross-sections (Fig. 5a–e) could be obtained using open source software packages such as 3D Slicer (https://www.slicer.org/), Paraview (https://www.paraview.org/) or ITK-SNAP (https://itksnap.org). In addition, a series of digital cross sections can be generated and presented in a more traditional way, similar to the atlases in printed form (Fig. 8 and supplementary materials).

## Discussion

### Experimental and computational considerations

During the creation of this atlas we followed currently the best experimental and computational practices (e.g., Amunts et al. 2013). For the purpose of the MR imaging, the brain was perfused with the contrasting agent to improve the anatomical details, left in the cranium to avoid distortions caused by its extraction from the skull, and scanned twice to increase the data diversity. Additionally, collecting the blockface images helped to facilitate subsequent 3D reconstruction and coregistration process by splitting it into two independent steps (Fig. 2, also Uberti et al. 2009).

Despite the care taken during the experimental handling, the specimen suffered from a few artifacts. The left paraflocullus was lost during the extraction of the brain from the cranium. Also, artifacts passing obliquely through the hindbrain and the brain stem occurred as a result of penetration with a sharp pin. Finally, in the myelin modality, the staining intensity on the dorsal- and ventral-most parts of consecutive triplets of sections alternates (*chattering* on Figs. 5b and 8) due to mass staining of the sections using the Galyas protocol.

An emphasis was put on the quality of the coregistration between individual modalities. Rigorous procedures for the data integration made it possible to virtually eliminate distortions related to global shrinkage due to fixation and cryoprotection. By utilizing deformable 3D reconstruction routines we mitigated non-uniform, section-specific distortions inherent to the cryosectioning and staining processes (Dauguet et al. 2007) and frequently reported as serious limitations of the atlases in printed form (Lanciego and Vázquez 2012). This allowed us to precisely match the histological data with the MR images and more accurately express the location of the histological sections in the stereotaxic coordinates.

The proposed experimental and computational pipeline uses open software, is of a general applicability, and could be used to create 3D brain atlases of other laboratory animals.

### Coregistration accuracy

To quantitatively characterize the coregistration accuracy, we calculated the distances between corresponding landmarks established in the histological modalities and in the reference MR image. The colocalization error between the Nissl-based 3D reconstruction and the MR image is 0.15 mm (median) and 0.36 mm (95${\text {th}}$ centile). In case of the myelin modality the respective values are: 0.11 and 0.31 mm. To put these values into perspective, Yang et al. (2012) registered 3D reconstructions based on a Nissl-stained mice sections to 30 $\upmu$m/voxel MR images and estimated the average accuracy at a level of 0.27 ± 0.14 mm (which corresponds to 9.0 ± 4.5 voxels).

It has to be noted that the provided values describe both, the coregistration error itself and the uncertainty in defining individual landmarks. The procedure could be improved by placing the landmarks multiple times and by many raters which would result in more precise estimates (Sergejeva et al. 2015). Nonetheless, the concordance between the modalities obtained in this study is considerably higher than that of typical printed 2D atlases.

### Delineation and nomenclature of brain structures

In both MR images (48 h and 30 days post-fixation), the gray matter appeared hyperintense, while the regions containing the white matter had low signal intensity. However, in each scan, the highlighting of brain structures was somewhat different due to the different time of contrasting agent penetration and absorption in the tissue (see Fig. 8) which is well described in the literature, for instance by Kim et al. (2009). While all four modalities were used as a basis for delineation of the anatomical structures (Fig. 6), the 30 days MR image was chosen as the spatial reference due to excellent differentiation of the white and gray matter and the least amount of distortions or imaging artefacts. Additionally, closer examination of the physical sections under a microscope, whenever the detail of lower-resolution images was insufficient, helped to achieve a more reliable segmentation.

We consider the parcellation provided in this atlas an important, first attempt of a systematic delineation of the adult opossum brain. Earlier publications exploring the cytoarchitecture of specific brain regions, due to their nature, rarely provide complete delineations of entire structures (Olkowicz et al. 2008; Wong and Kaas 2009). Usually, only the center is indicated, sometimes accompanied by an approximate border. In the present work we attempted to completely delineate entire structures as consistent three-dimensional objects. A consequence of this approach is the varying level of detail of subdivisions in different structures, as only those unambiguously distinguishable with cyto- and myelo-architectonic imaging data of moderate resolution were outlined. This is, for instance, why the neocortex was delineated as a single region. While, in principle, more series of sections stained for other features than cyto- and myeloarchitecture could be collected from the same brain, this is not a preferable option in our case as it compromises the 3D nature of the reconstruction from serial sections. This happens because each additional series increases the spacing between two sections stained with the same method. In this situation, a natural, and already ongoing, continuation of the presented study, is to draw the fine neuroanatomical subdivisions based on histological and immunohistochemical stainings acquired from additional specimens.

The nomenclature of the brain structures has a long tradition and it is still evolving. While there are ongoing works to develop a consensus neuroanatomical terminology and delineation criteria, frequently they differ not only between species (Swanson 2015) but also within (Van De Werd and Uylings 2014). In the present work we followed the nomenclature and delineation criteria proposed in the widely recognized atlases of Paxinos and Watson (2007), Swanson (2004) and Dong (2008).

### Anatomical variability

A limitation of the present atlas is the available experimental basis of two male specimen, with only one brain fully imaged and delineated. This precludes, in particular, an assessment of anatomical variability. Such evaluation is further impeded by the scarcity of research in this domain. We found only two studies, Karlen and Krubitzer (2006) and Evans et al. (2010), investigating the variability of body mass. However, only the former, based on a mixed group of males and females of various age, focuses on the brain mass and volume.

In the Karlen and Krubitzer study, 11 female and 11 male opossums, 6–32 months old, (13.9 ± 8) months (mean ± SD), with a weight between 66 and 143 g, (97.6 ± 26) g, were examined. The one year old individual from which we obtained the brain had a body mass of 145.7 g, considerably heavier than the average. On the other hand, the cranium was acquired from an animal with a weight of 129.8 g which is closer to the reported average.

In terms of the brain, Karlen and Krubitzer report the average mass of (828.0 ± 67.6) $\upmu$g and the average volume of (768 ± 53) mm$^3$. The brain we used had a mass of 1 009 $\upmu$g and a volume (excluding the ventricular system) of 974.7 mm$^3$. Unfortunately, Karlen and Krubitzer do not provide a table with measurements for individual animals which prohibits more detailed analyses. Nonetheless, their results may indicate how to apply the present template to a new individual.

To fully utilize our atlas, the anatomical variability in opossum should be further studied using MR and micro-CT imaging. This would make it possible to depict quantitative information on variability within examined groups using approaches such as the Mean Deformation Template (Kovacević et al. 2005), and thus to assess quantitative differences between shapes of the brain structures and the cranium.

### Stereotaxic reference system

The stereotaxic coordinate system is based on non-destructive micro-CT imaging of the skull and allows one to express any location within the brain in relation to the cranial landmarks. The main rationale behind this approach were difficulties with identifying these points in the MR images. The protocol was tuned to differentiate between the gray and the white matter which resulted in an insufficient image contrast in other regions. Conversely, on the micro-CT scan, the landmarks are clearly distinguishable and, once located, their location could be transferred to the MR modality.

The coronal plane acquired by inclining the brain and aligning the bregma and lambda horizontally is relatively easy to determine in a live animal and reproducible between individuals (Fig. S6, supplementary materials). This approach gives effects comparable to the method of positioning of the rat brain used in Swanson (2004), who places it on the dorsal surface of the cortex and supports the cerebellum with a cylinder. Due to the large olfactory bulbs of the opossum, the Swanson’s method is impractical here.

Acquiring the skull from a different animal than the brain inevitably introduces some inaccuracies. Nevertheless, in view of the discussion above and the facts that both our individuals were of the same age and sex, derived from the same stock, and bred in the same conditions, we argue that this is not a significant shortcoming. This is further confirmed by the fitting quality of the brain to the cranium (Fig. 7b).

### Summary

The primary contribution of this study is the first three-dimensional, multimodal, stereotaxic atlas of the adult *Monodelphis domestica* opossum brain. The atlas was obtained by coregistration of imaging data from multiple techniques which allowed us to delineate 113 brain structures.

Thanks to the fully three-dimensional form and the possibility of arbitrary cross-sections, the atlas provides a thorough view of the opossum brain anatomy, which would be impossible to achieve using only cuts in the cardinal planes, e.g., measuring angles and distances across the brain, and performing morphometric studies. It could be used to design atypical, oblique cuts preserving structures or connections between specific brain regions. Moreover, the reference coordinate system makes the atlas more directly applicable in sterotaxic surgeries, lesions, electrophysiological recordings as well as in the analysis of the resulting data. The present template is also the first step towards an atlas of the opossum brain development, allowing for identification of changes in the brain anatomy induced by genetic and epigenetic factors. Furthermore, it will allow precise comparative analysis of the brain of opossum and other marsupial and placental mammals (e.g., Izpisua Belmonte et al. 2015).

## Electronic supplementary material

Below is the link to the electronic supplementary material.

The supplementary pages include an extended description of the procedure for the 3D reconstruction, a detailed illustration of the method of establishing the stereotaxic coordinate system as well as series of cross-sections throughout the three-dimensional images comprising the atlas. The complete, three-dimensional atlas and the unprocessed imaging datasets used to produce the template can be accessed via an online repository (https://osf.io/hbx5p/). Interactive 3D visualizations of the delineated brain structures are available through the 3D Brain Atlas Reconstructor website (Majka et al. 2013, https://3dbars.org). Individual imaging modalities along with the delineations can be also accessed via the ScalableBrainAtlas system (Bakker et al. 2015, https://scalablebrainatlas.incf.org/). (PDF 63175 kb)
