# Supplementary material for: A three-dimensional stereotaxic atlas of the gray short-tailed opossum (Monodelphis domestica) brain
Source: Brain Struct Funct. 2017 Dec 6;223(4):1779–95. doi: 10.1007/s00429-017-1540-x (PMC5884921; doi:10.1007/s00429-017-1540-x)
Supplement: Supplementary file 1 — The supplementary pages include an extended description of the procedure for the 3D reconstruction, a detailed illustration of the method of establishing the stereotaxic coordinate system as well as series of cross-sections throughout the three-dimensional images comprising the atlas. The complete, three-dimensional atlas and the unprocessed imaging datasets used to produce the template can be accessed via an online repository (https://osf.io/hbx5p/). Interactive 3D visualizations of the delineated brain structures are available through the 3D Brain Atlas Reconstructor website (Majka et al. 2013, https://3dbars.org). Individual imaging modalities along with the delineations can be also accessed via the ScalableBrainAtlas system (Bakker et al. 2015, https://scalablebrainatlas.incf.org/). (PDF 63175 kb) [file 429_2017_1540_MOESM1_ESM.pdf]

Supplementary materials for the article:

A three-dimensional stereotaxic atlas of the gray short-tailed opossum (*Monodelphis domestica*) brain

Piotr Majka[1*], Natalia Chlodzinska[2], Krzysztof Turlejski[3],
Tomasz Banasik[4], Ruzanna L. Djavadian[5], Władysław P. Węglarz[4],
Daniel K. Wójcik[1]

(1) Laboratory of Neuroinformatics, Department of Neurophysiology, Nencki Institute of Experimental Biology of Polish Academy of Sciences, 3 Pasteur Street, 02-093 Warsaw, Poland; p.majka@nencki.gov.pl; (2) Laboratory of Neurobiology of Development and Evolution, Nencki Institute of Experimental Biology of Polish Academy of Sciences, 3 Pasteur Street, 02-093 Warsaw, Poland; (3) Department of Biology and Environmental Science, Cardinal Stefan Wyszynski University, 1/3 Woycicki Street, 01-938 Warsaw, Poland; (4) H. Niewodniczański Institute of Nuclear Physics of Polish Academy of Sciences, Radzikowskiego 152, 31-342 Cracow, Poland; (5) Laboratory of Calcium Binding Proteins, Department of Molecular and Cellular Neurobiology, Nencki Institute of Experimental Biology of Polish Academy of Sciences, 3 Pasteur Street, 02-093 Warsaw, Poland

A repository comprising the atlas and the unprocessed imaging datasets used to produce the template is available at:

https://osf.io/hbx5p/

Various visualizations of the atlas are available at:

https://youtu.be/kbXB3Y4DJrk

Selected details of the reconstruction of the histological slices

Detailed description of the coarse-to-fine reconstruction step

The coarse-scale alignment involved the blockface image stack which is a spatially consistent 3D image of the brain just before sectioning. Since every stained section corresponds to a blockface image, to recover the anatomical shape of the brain, images of individual stained sections were aligned to their blockface counterparts yielding a series of coarse transformations.

The fine-scale reconstruction was performed by sequentially aligning sections starting with sections located centrally in the image stack (sections no. 160 and 150 for Nissl- and myelin-stained sections, respectively) towards either end of the stack. Red image channels of both image stacks were extracted and smoothed with 5×5 pixels median filter and the registration was performed with cross correlation as an image similarity metric. This resulted in a series of fine rigid transformations.

Finally, the transformations obtained in both steps were merged by combining the high-frequency component of the fine-scale transformation with the coarse-scale alignment. This was done by Gaussian smoothing ($\sigma = 5$ sections) of individual parameters of the fine-scale transformation (translation and rotation angle) across the z (stack) dimension, filtering them out, and combining with the parameters of the coarse-scale registration. The image stack was then transformed accordingly and passed to the deformable reconstruction routine.

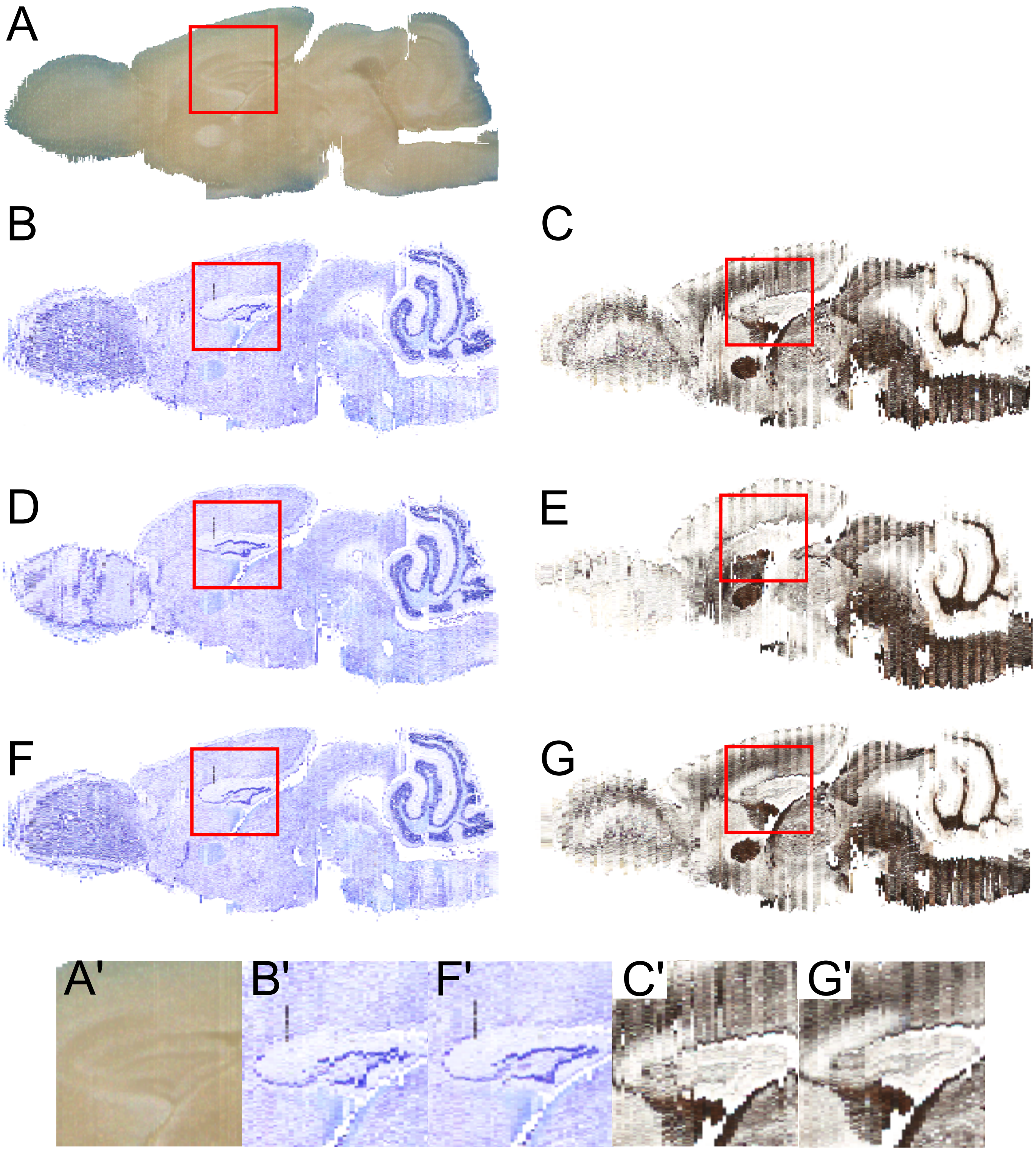

Figure S1: Comparison of the results of the rigid reconstruction of the 3D brain images based on the series of sections stained with the Nissl method and for melinated fibres. Digital saggital cross-sections through the image at various stages of the reconstruction: A) Reference blockface image; B,C) Coarse-stage reconstructions; D,E) Fine-stage reconstructions; F,G) The result of merging the coarse- and fine-stage transformations; A'-G') Magnification of the regions denoted with the red rectangles on the panels A-G.

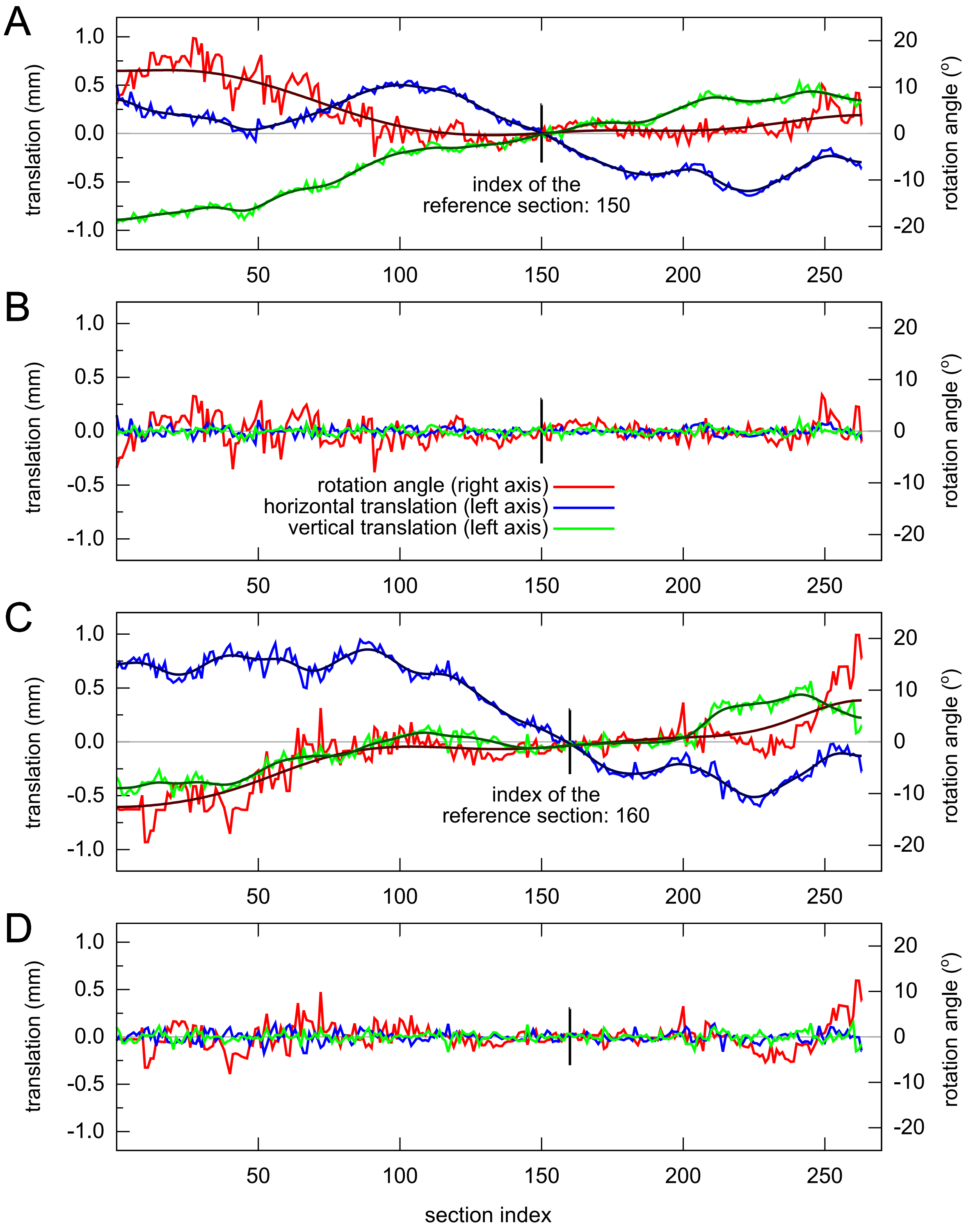

Figure S2: Results of the coarse- and fine-stage tranformations calculated during the rigid reconstruction of the 3D brain images based on series of Nissl- and myelin-stained sections. The abscissas denote indexes of the sections (1-264), the left ordinate axis denotes the translation while the right axis denotes the rotation angle for the given section. The red curves indicate the rotation angle, the blue curves denote the translation in the horizontal direction while the green curves mark translation in the vertical direction.
A) Alignment of the Nissl-stained sections. Bright colors indicate parameters of the fine-scale transformations. Dark shades of red, green and blue denote parameters of the low-frequency component of the fine-scale transformations parameters. **B)** High-frequency component of the fine-scale transformation used to obtain the rigid reconstruction of the Nissl-stained sections. **C-D)** Anologous results for the sections stained for myelinated fibres.

Detailed description of the deformable reconstruction step

The deformable reconstruction process was split into four stages. The output of one stage constituted the input to the next one. First, highly deformed sections which, if unchanged, would cause reconstruction artefacts, were fixed. They were selected manually and corrected by warping towards the closest undistorted neighbor. Registration parameters were chosen individually depending on the type and magnitude of the distortion. The next step was to reconstruct smooth brain outline. The input data were the sections' outlines warped using transformations computed in the previous step and the summed square distance was used as the image similarity metric. The last step was reconstruction of the interior of the brain volume and was divided into two parts – coarse and fine. While this division is not strict, it emphasizes the necessity of using different sets of parameters to gradually increase the reconstruction precision. As the registration in this stage uses intensity images and is intra-modal, cross-correlation is used as a similarity metric.

Table S1: Parameters for deformable reconstruction of brain volume based on Nissl-stained sections.

Number of iterations	Image type	Sections range	ANTS registration parameters
(–)	images	34 selected (*)	CC(16, 0.5), Gauss(3,1),<br>Iterations: 1000x1000x1000x0x0
5	masks	whole stack	MSQ(-, 0.05), Gauss(1,1),<br>Iterations: 1000x1000x1000x0x0
5	images	whole stack	CC(4, 0.05), Gauss(1,1),<br>Iterations: 1000x1000x1000x0x0
6	images	whole stack	CC(4, 0.01), Gauss(1,1),<br>Iterations: 1000x1000x1000x0x0
2	images	1-80 (**)	CC(4, 0.01), Gauss(1,1),<br>Iterations: 1000x1000x1000x0x0

(–) the initial step is not considered as an iteration; (*) Compensation of highly deformed sections was performed on a manually selected set of slices (see text for explanation); (**) Two more iterations were carried out for section constituting the olfactory bulb to improve the shape of this part of the brain.

Table S2: Parameters for deformable reconstruction of brain volume based on myelin-stained slices.

Number of iterations	Image type	Sections range	ANTS registration parameters
(–)	images	17 selected (*)	CC(16, 0.5), Gauss(3,1); CC(4, 0.25), Gauss(3,1) (**), Iterations: 1000x1000x1000x0x0
8	masks	whole stack	MSQ(-, 0.05), Gauss(1,1), Iterations: 1000x1000x1000x0x0
5	images	whole stack	CC(2, 0.01), Gauss(1,1), Iterations: 1000x1000x1000x0x0
4	images	1-80 (***)	CC(2, 0.01), Gauss(1,1), Iterations: 1000x1000x1000x0x0

(–) the initial step is not considered as an iteration; (*) Compensation of highly deformed slices was performed on a manually selected set of slices (see text for explanation); (**) All sets of different parameters used in this stage are presented as different slices were corrected using different parameters; (***) Two more iterations were carried out for slices constituting the olfactory bulb to improve the shape of this part of the brain.

Table S3: Summary of the parameters used during deformable coregistration of the histological volumes towards the MR image.

step no.	w_{CC}	w_{MSQ}	w_{PSE}	fixed / moving image resolution (μm)	ANTS registration parameters
1	0	1	1.0	200	CC(5), SyN(0.35), Gauss(0,0), Iterations: 1000x1000
2-3	1	0	0.5	200	CC(5), SyN(0.25), Gauss(0,0), Iterations: 1000
4-6	1	0	0.5	100	CC(5), SyN(0.25), Gauss(0,0.1), Iterations: 1000
7-8	1	0	0.5	75	CC(5), SyN(0.25), Gauss(0,0.75), Iterations: 1000
9-10	1	0	0.5	50	CC(5), SyN(0.25), Gauss(0,0.05), Iterations: 1000

w_{CC}: weight of the cross-correlation metric; w_{MSQ}: weight of the summed square distances metric; w_{PSE}: weight of the point set estimation metric.

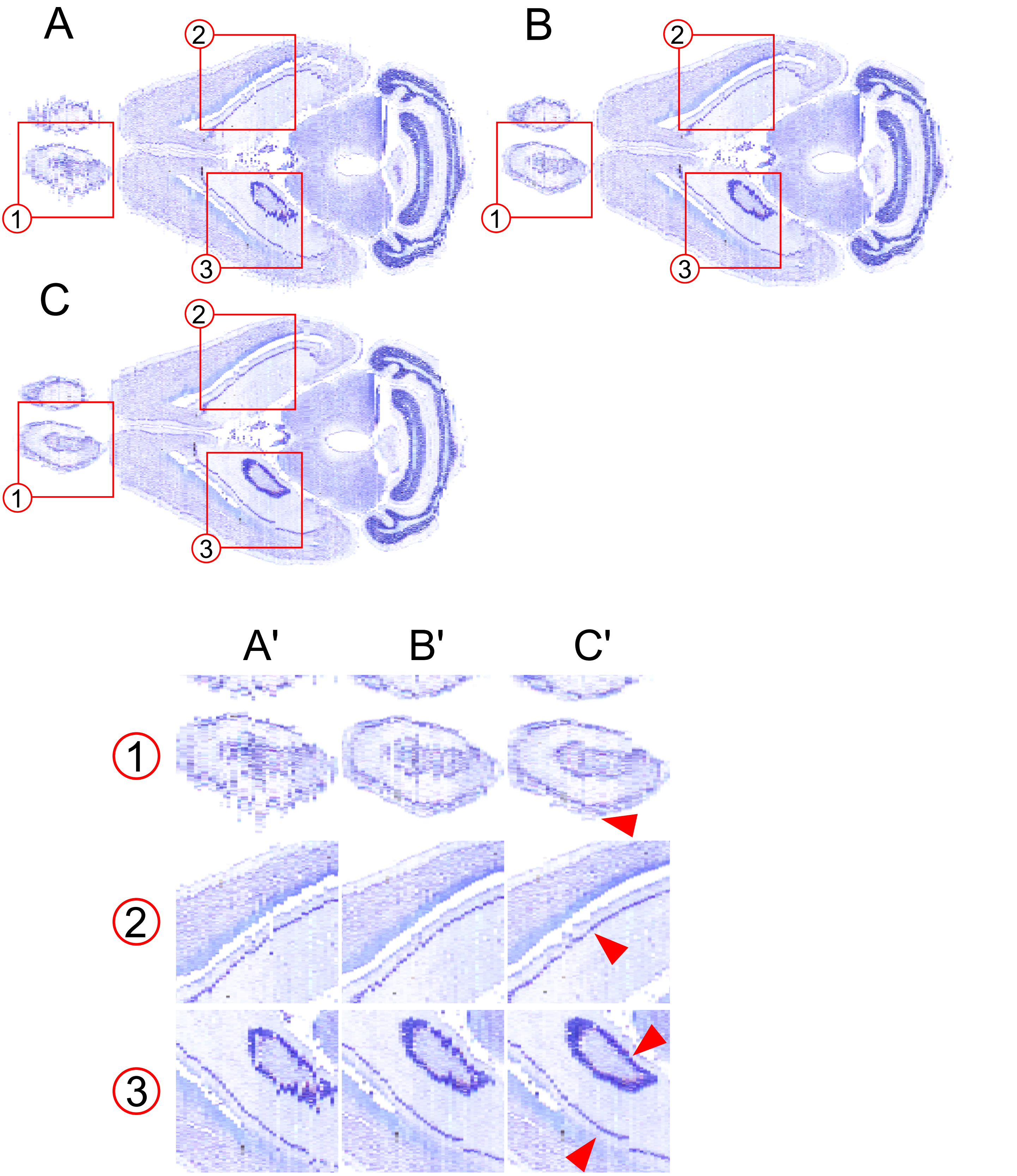

Figure S3: Consecutive steps of the deformable refinement of the 3D brain image based on images of serial coronal sections stained with the Nissl method; Horizontal cross-sections. A) Input rigid reconstruction; B) Reconstruction after alignment of the sections' outlines; C) Final deformable reconstruction. Rows ① to ③ depict fragments denoted with red rectangles on panels A-C. Red trianges indicate fragments of the reconstruction which have improved the most significantly in comparison with the input affine reconstruction.

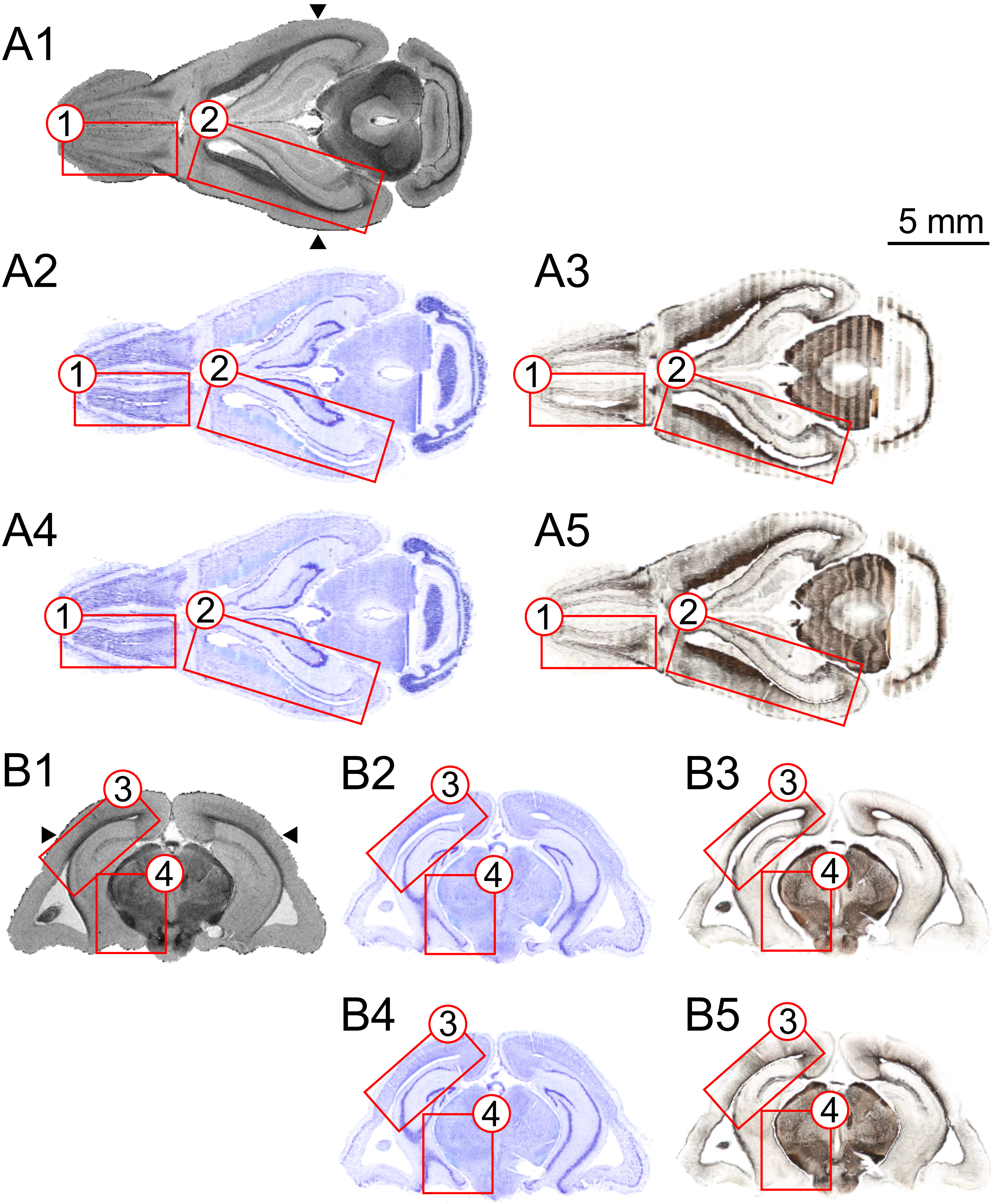

Figure S4: Comparison of the results of the coregistration of the 3D reconstructions based on the Nissl- and myelin-stained sections with the reference MR image (A1, B1) after the affine coregistration (A2, A3, B2, B3) and after the deformable coregistration step (A4, A5, B4, B5). Horizontal (A1-A5) and coronal cuts (B1-B5). After only the affine coregistration step, the distortions specific to the staining technique are noticeable e.g. tissue shrinkage next to ventricles (red rectangles ② and ③). After the deformable reconstruction step these distortions have been significantly mitigated (compare e.g. A3-② vs. A5-② or B3-③ vs. B5-③).

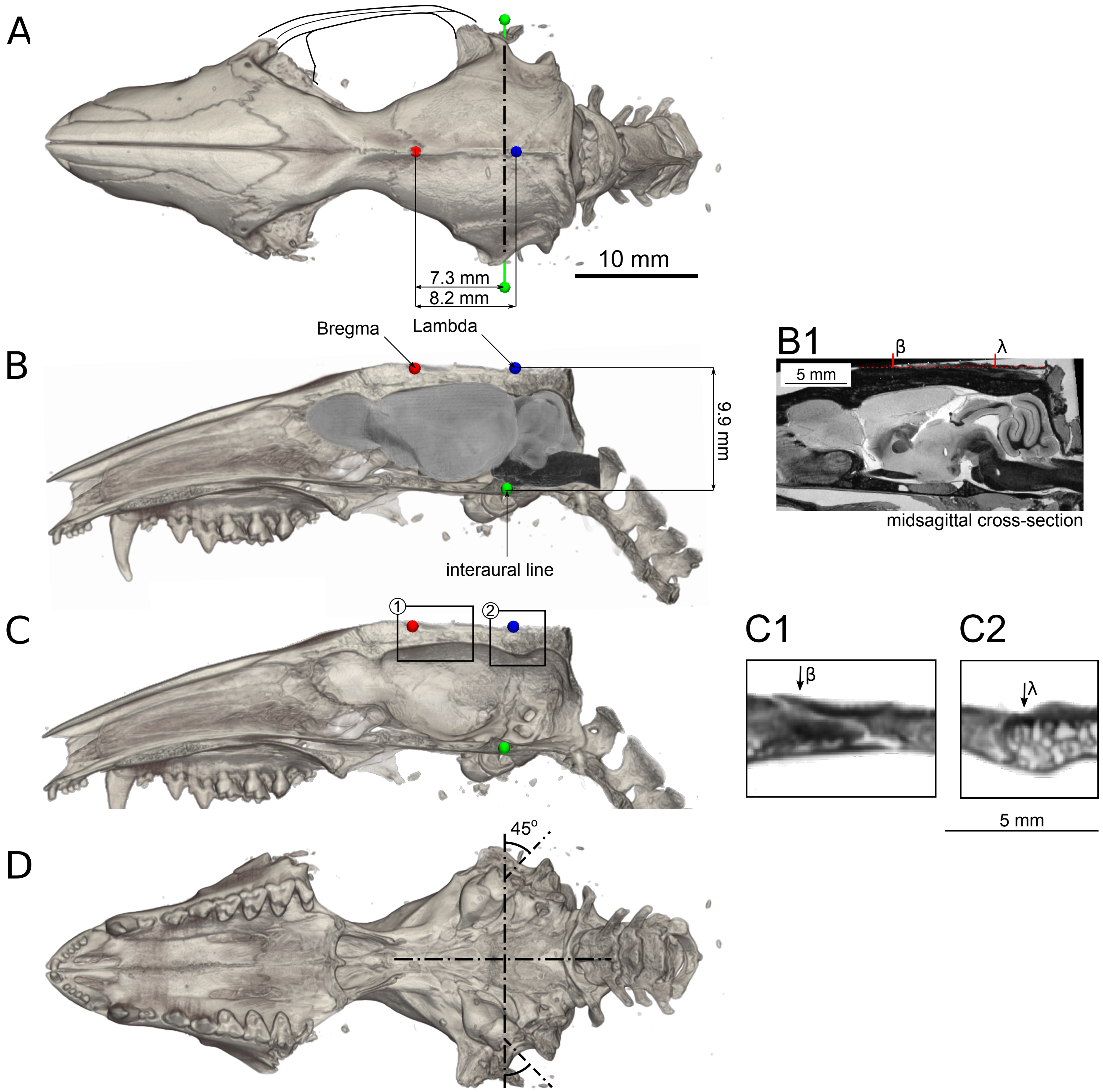

Figure S5: Extended ilustration of the structure of the opossum skull and the landmarks used to establish the stereotaxic coordinate system. A) Horizontal projection; the right zygomatic arch, trimmed during preparative procedures, was drawn manually; B) sagittal projection; the skull was digitally cut along the midsagittal plane and the MR image aligned to the braincase is shown. B1) Midsagittal cross-section through the MR image, the arrows indicate the location of the cranial landmarks, bregma (β) and lambda (λ). The dotted line indicates the level of both landmarks. C) Midsagittal cross-section through the skull whithout the MR image overlay. The braincase is clearly visible in detail. Rectangles ① and ② highlight the neighborhood of the bregma and lambda points magnified on panels C1 and C2, respectively. D) Ventral projection; the entry to the ear canals is angled at approx. 45 deg.

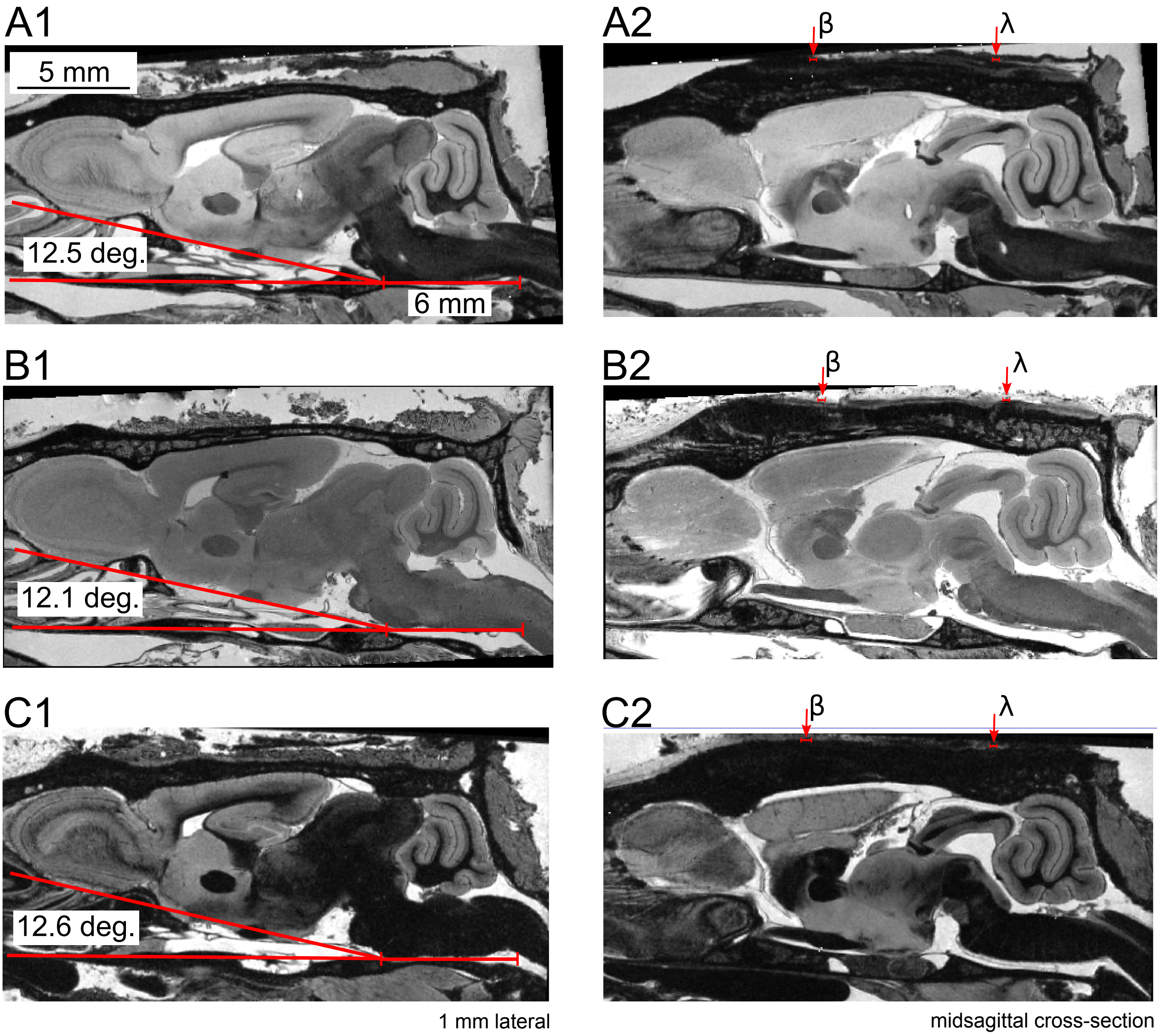

Figure S6: The method for establishing the stereotaxic orientation illustrated with three diferent specimen of one year male opossum imaged using the same MR protocol. The brain is positioned so that the ventral surfaces of the pons and the brainstem are oriented horizontally (the horizontal red lines in the left column). In this orientation, the brain is lifted rostrally by 11-13° and the bregma and lambda are also aligned horizontally. The coronal sectioning plane defined in this way is relatively easy to determine in a live animal for the purpose of stereotaxic interventions. A) The brain of an animal from which the Nissl- and myelin-stained sections were obtained. B and C) Additional specimen not described in the article. A1-C1) Parasagittal cross sections, 1 mm lateral from the midsagittal plane: at this level the flat orientation of the brainstem is clearly visible. A2-C2) Cross-sections through the midsagittal plane. The large sagittal crest is clearly visible dorsal to the brain. The arrows indicate the location of the cranial landmarks, bregma (β) and lambda (λ). The small red bars near the landmarks illustrate the uncertainity of finding these points using only the MR images. Out of the three examples presented in this figure, only in the case B the landmarks can be located unambigously. For comparison, see also Fig. S5, panels C, C1, and C2.

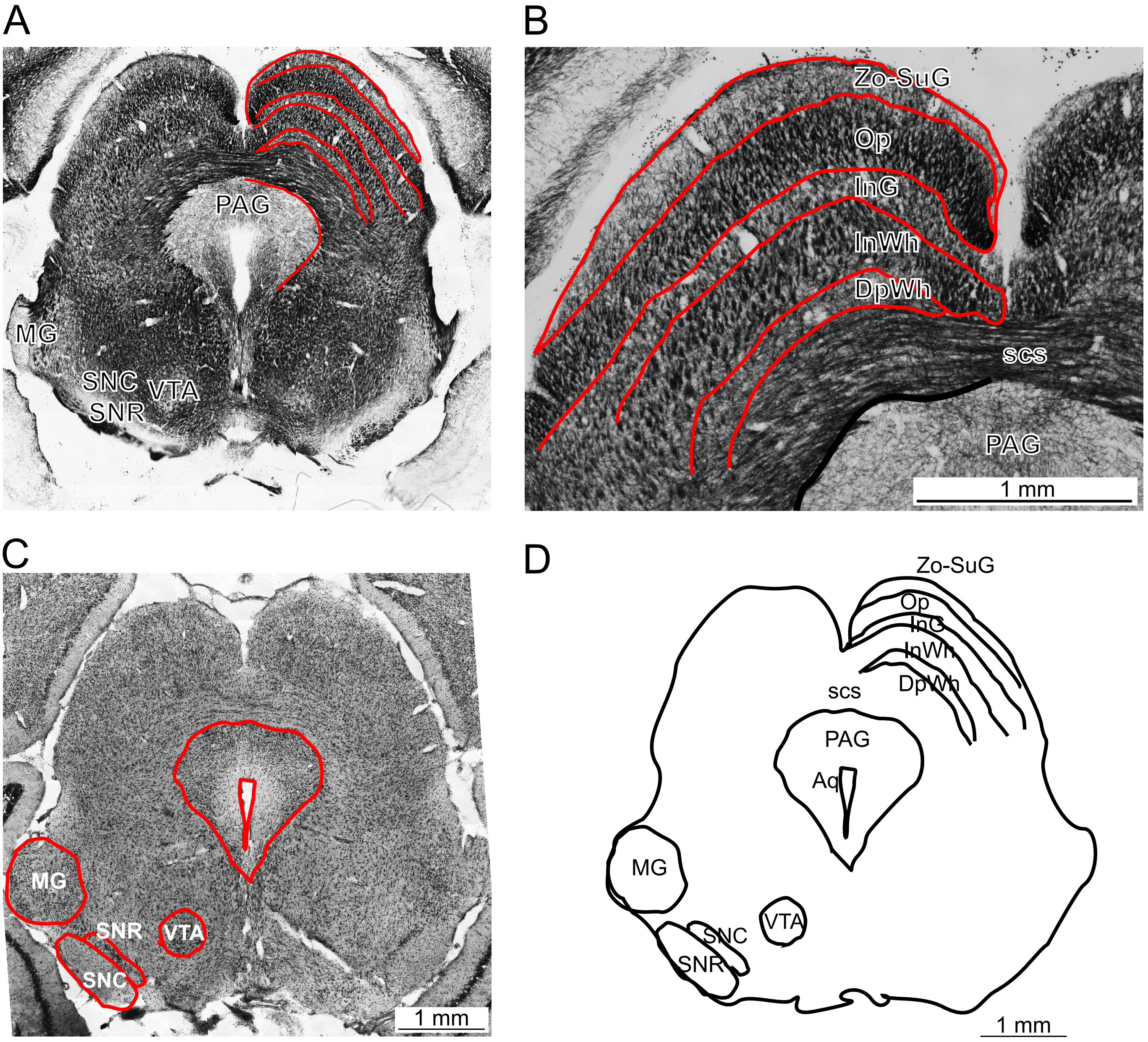

Figure S7: Images of coronal transections through the midbrain on myelin- (A, B) and Nissl-stained (C) sections under a microscope. The same myelin-stained section in low (A) and high (B) magnification. Layers of the superior colliculus are best distinguished by differential intensity of myelin staining.

Coronal cross-section 1
17.5 mm rostral from bregma

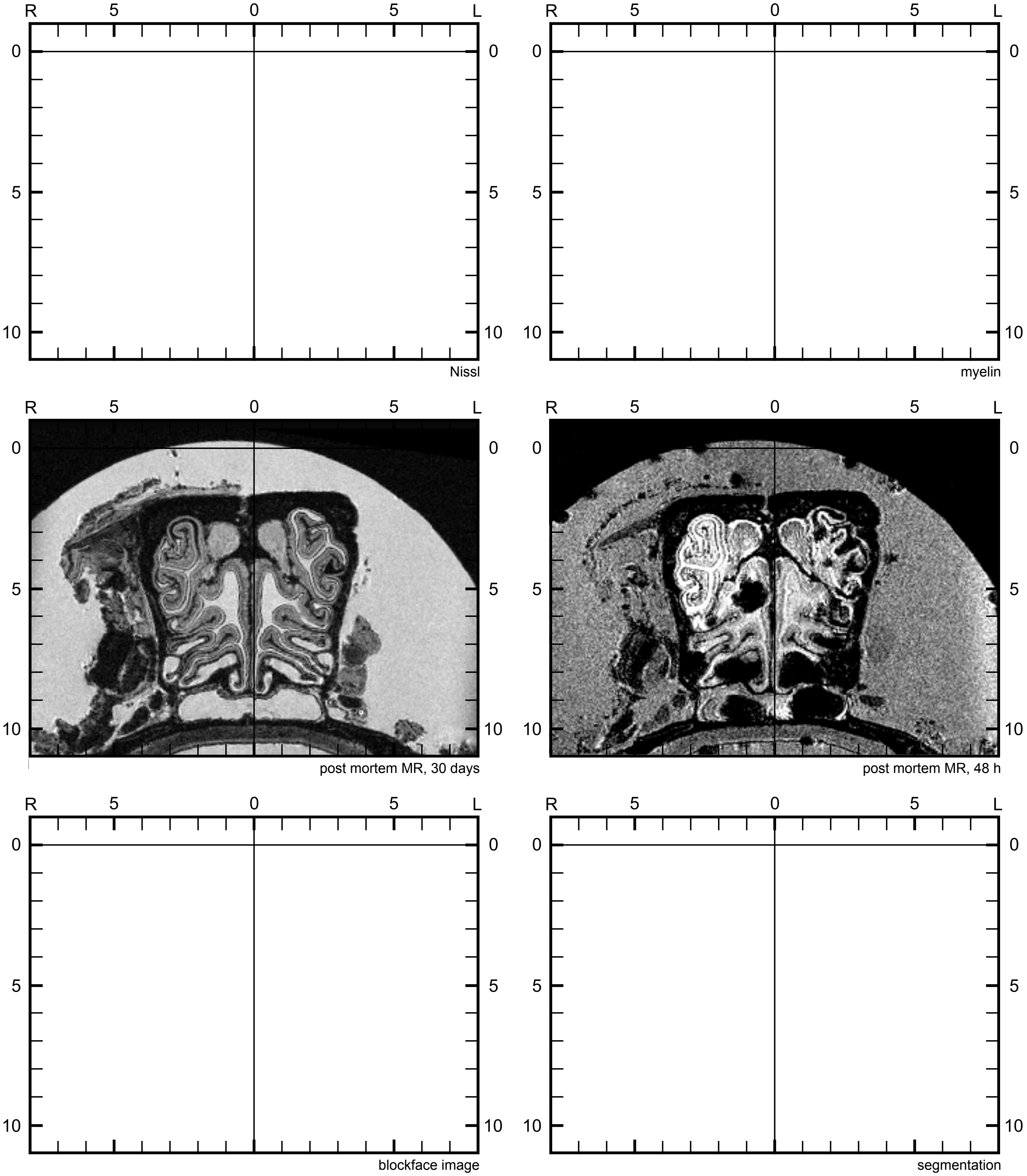

Coronal cross-section 2
17.0 mm rostral from bregma

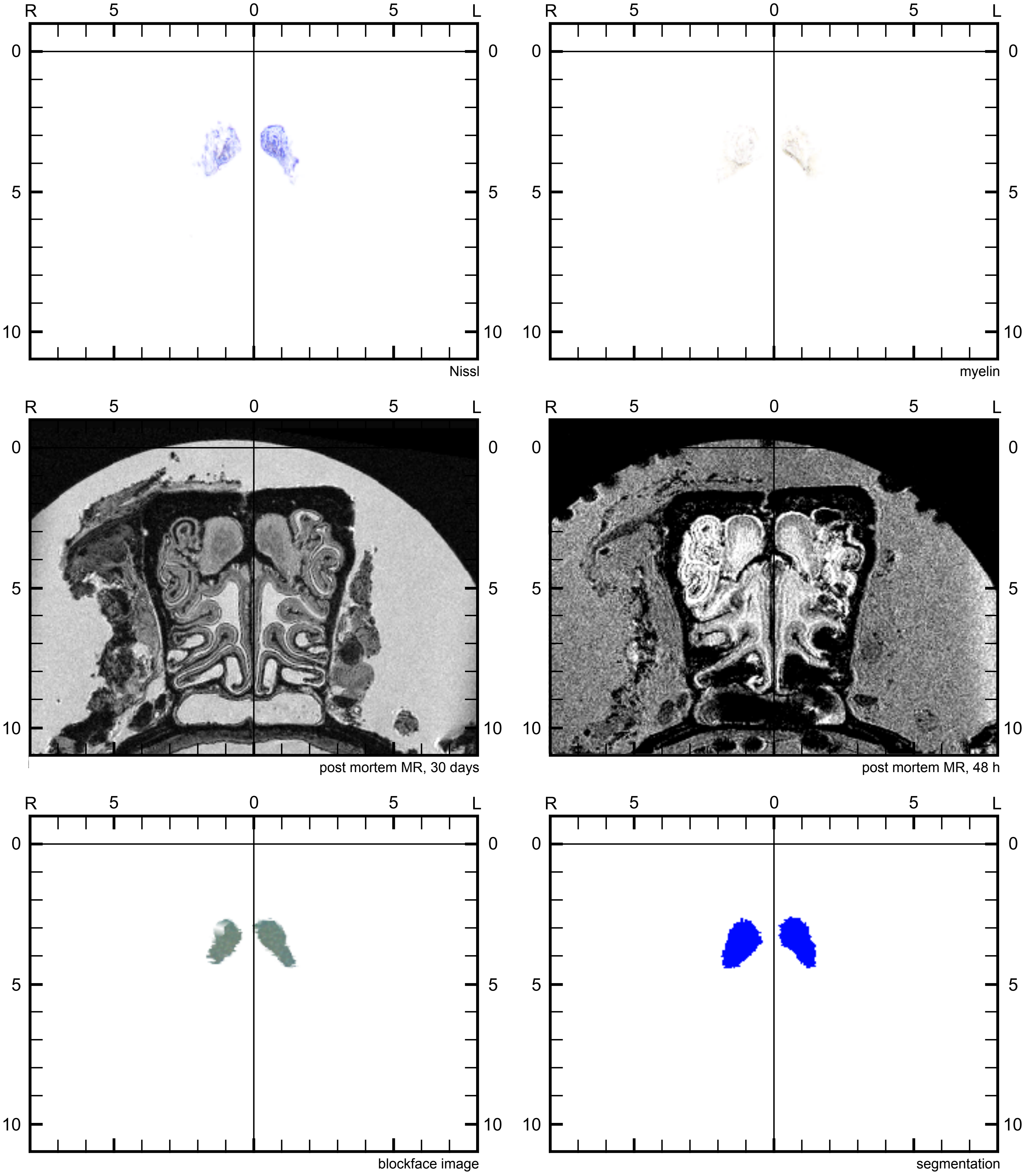

Coronal cross-section 3
16.5 mm rostral from bregma

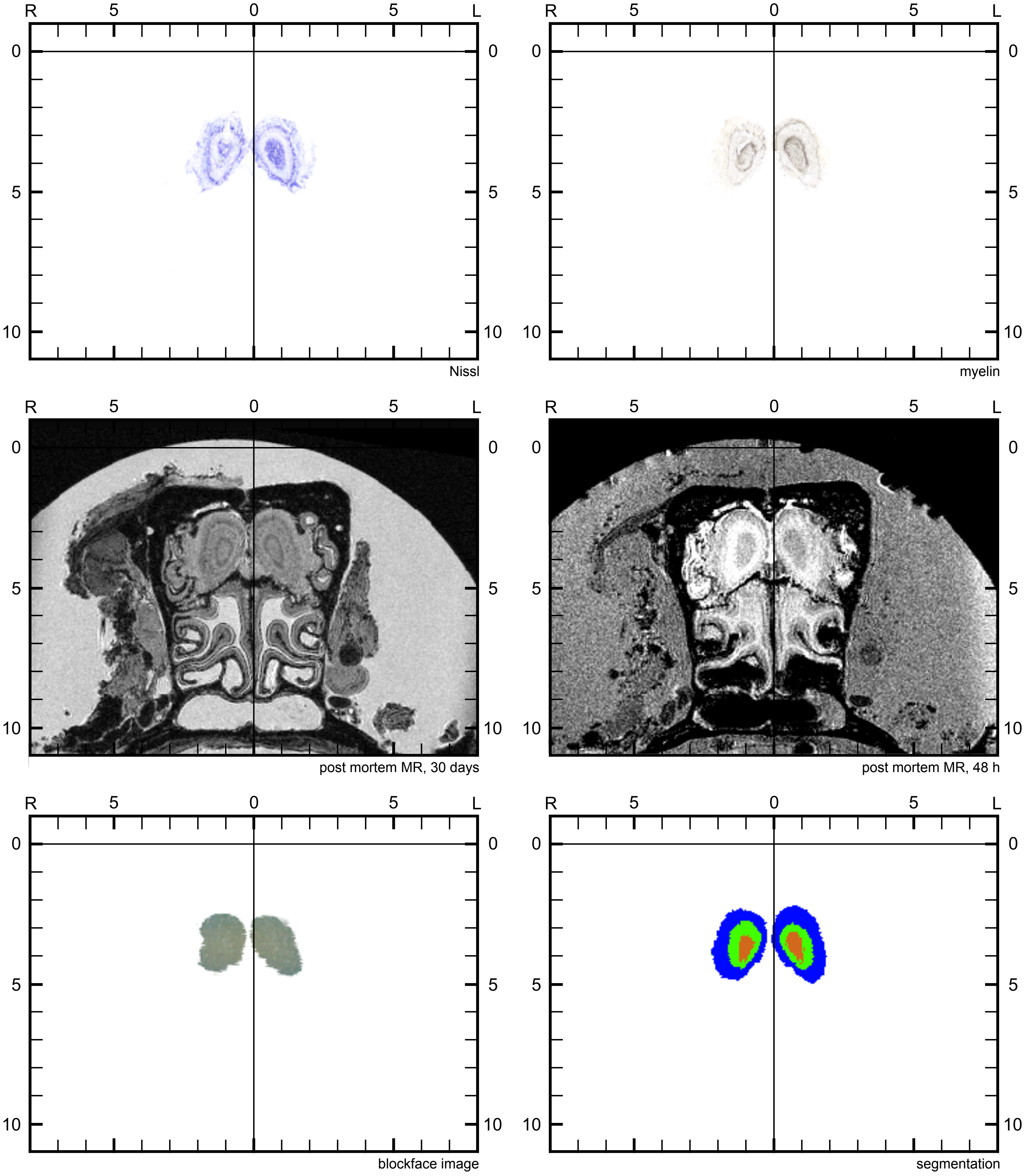

Coronal cross-section 4
16.0 mm rostral from bregma

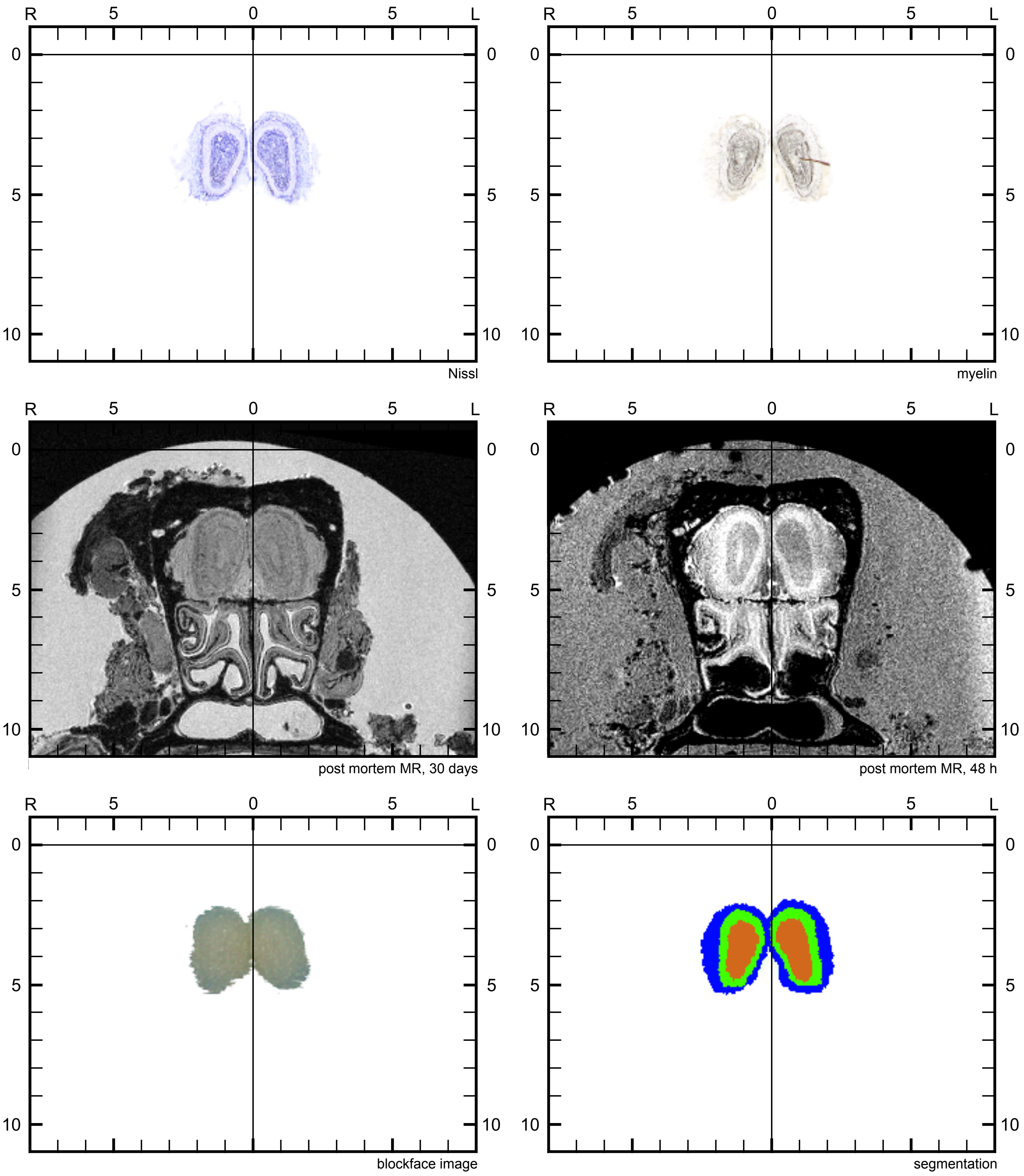

Coronal cross-section 5
15.5 mm rostral from bregma

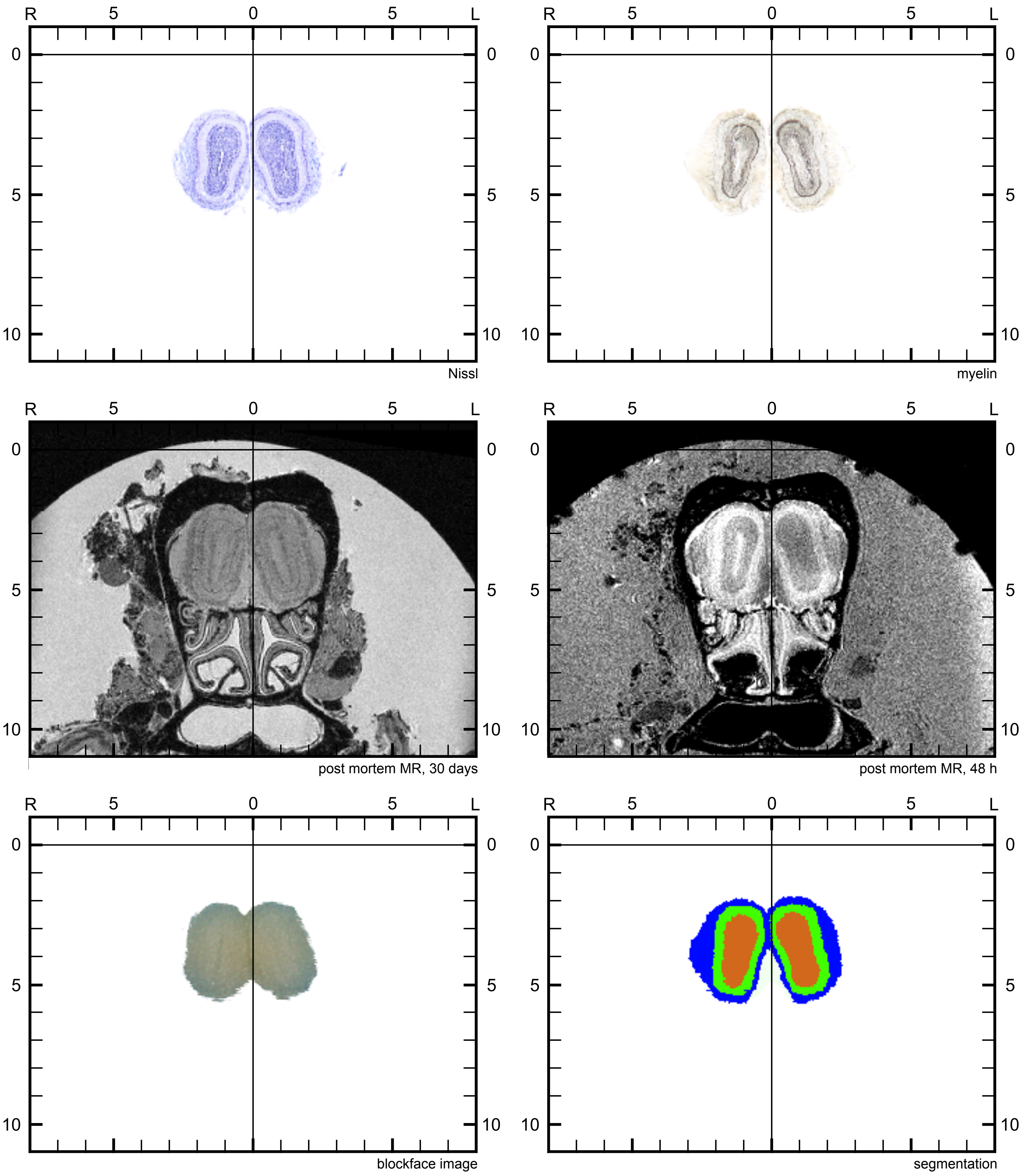

Coronal cross-section 6
15.0 mm rostral from bregma

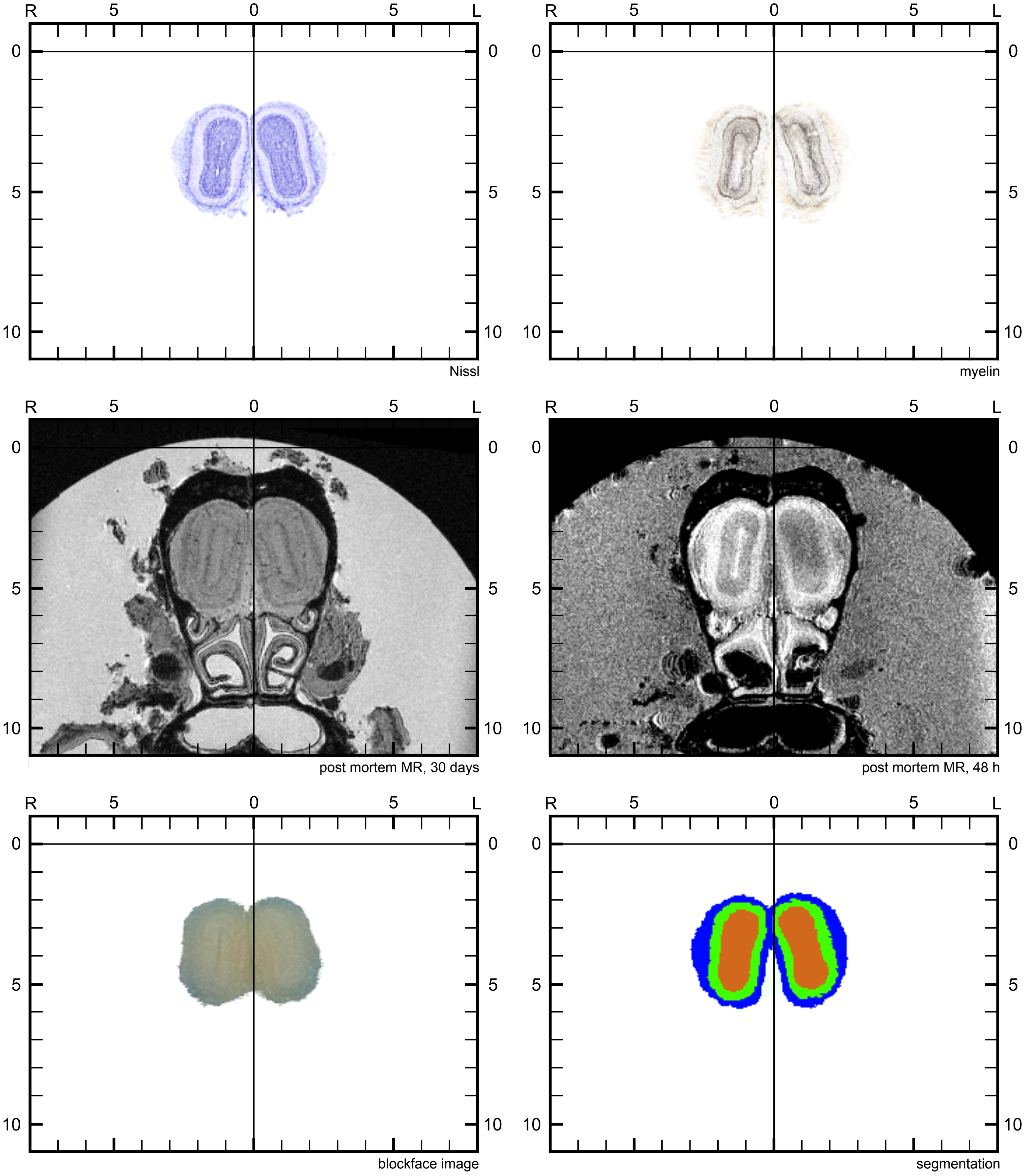

Coronal cross-section 7
14.5 mm rostral from bregma

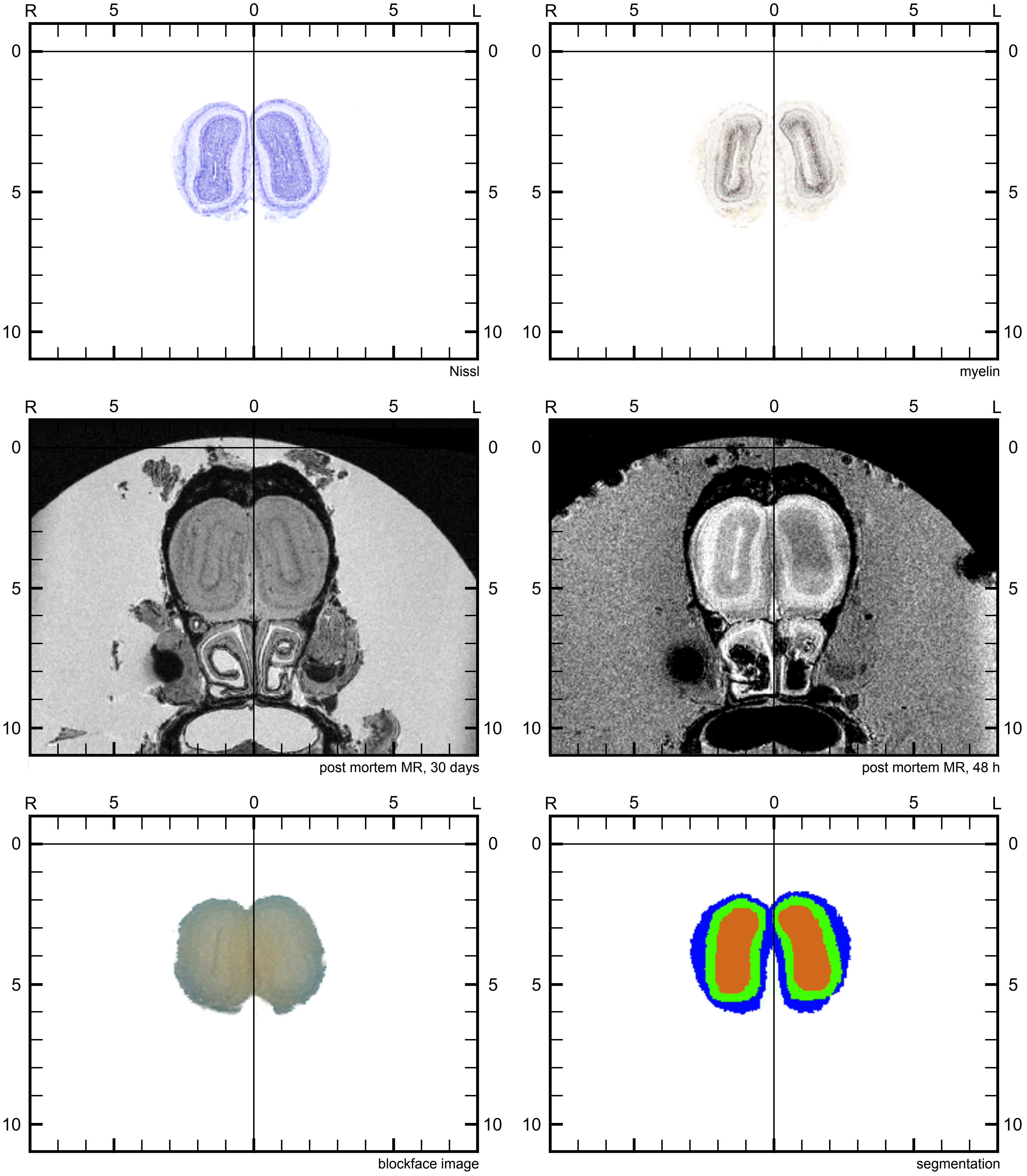

Coronal cross-section 8
14.0 mm rostral from bregma

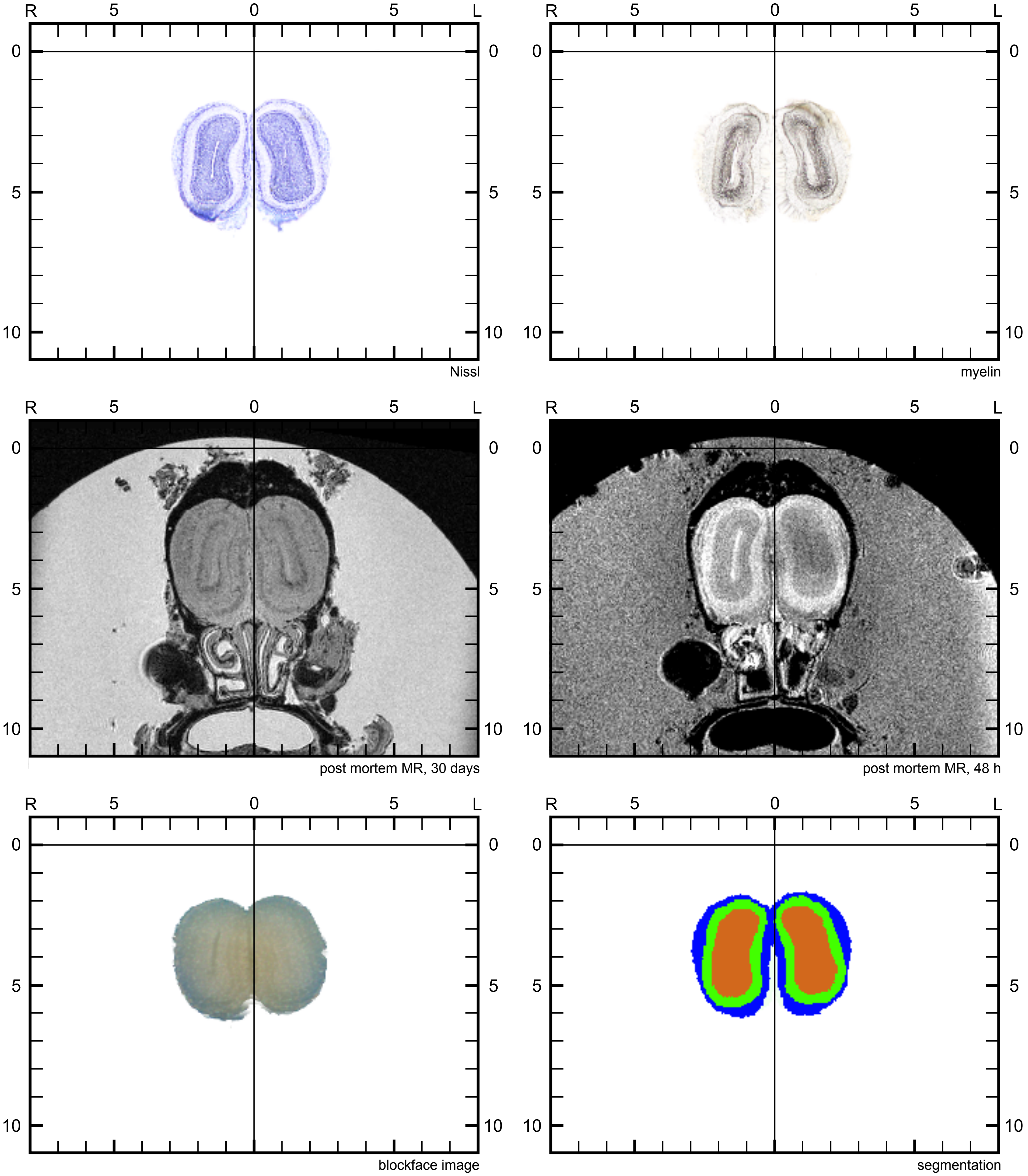

Coronal cross-section 9
13.5 mm rostral from bregma

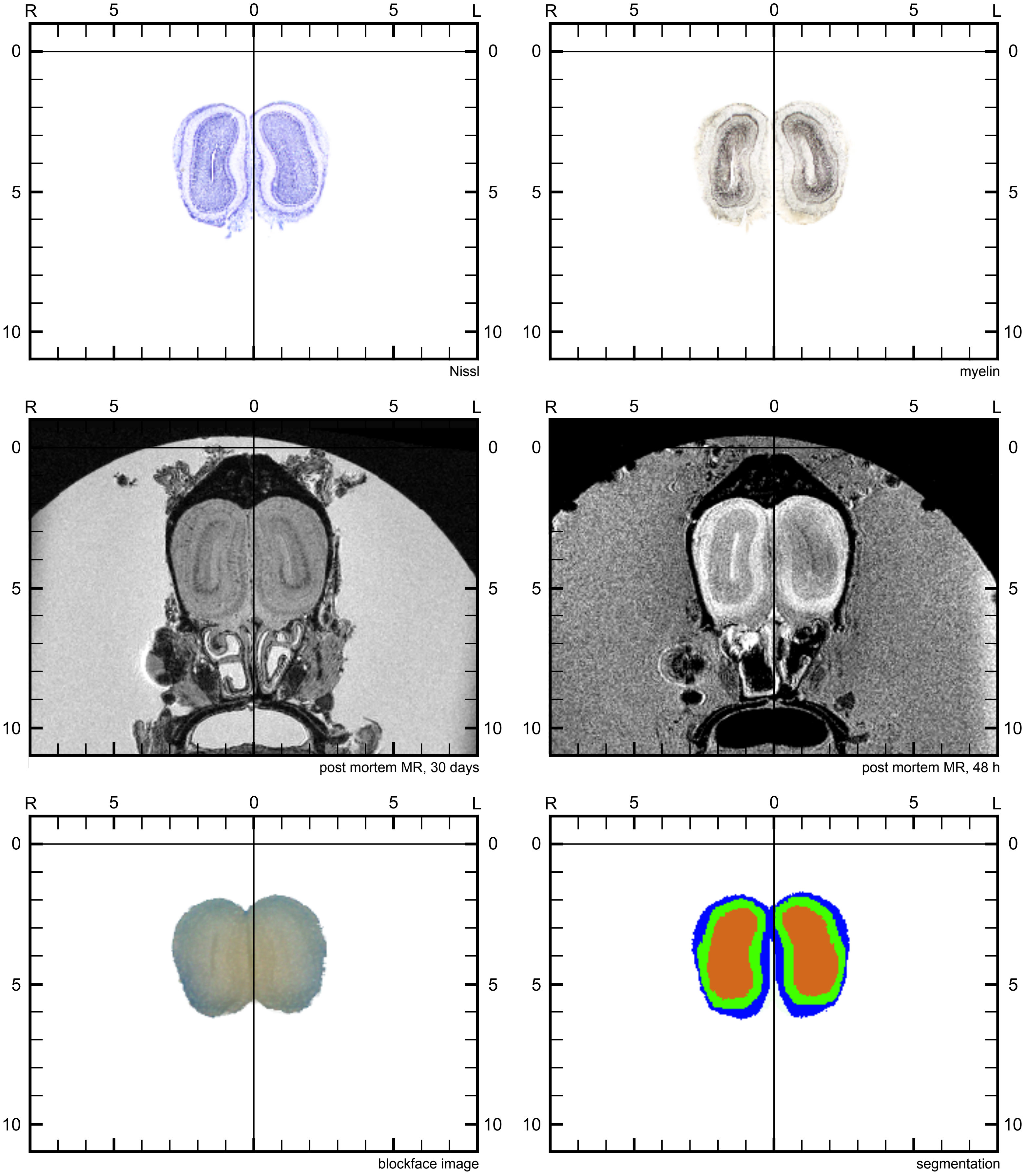

Coronal cross-section 10
13.0 mm rostral from bregma

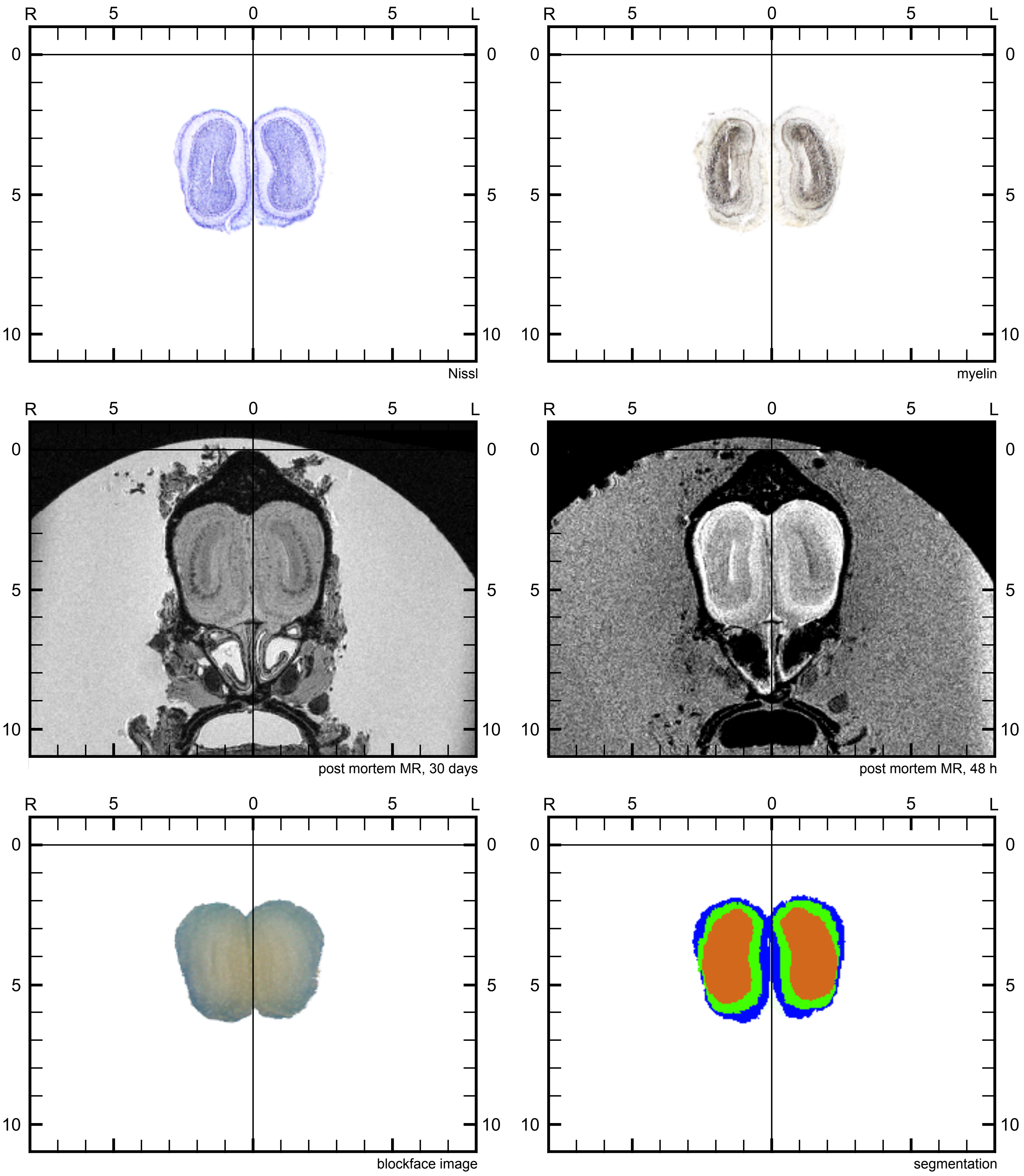

Coronal cross-section 11
12.5 mm rostral from bregma

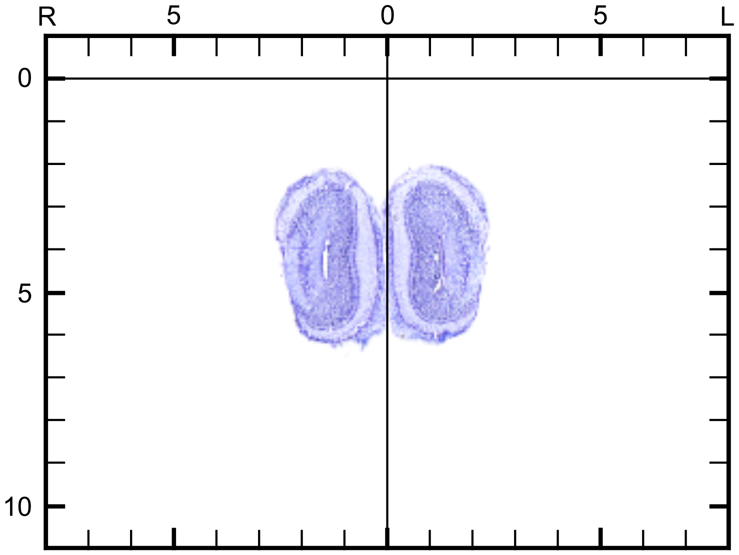

Nissl

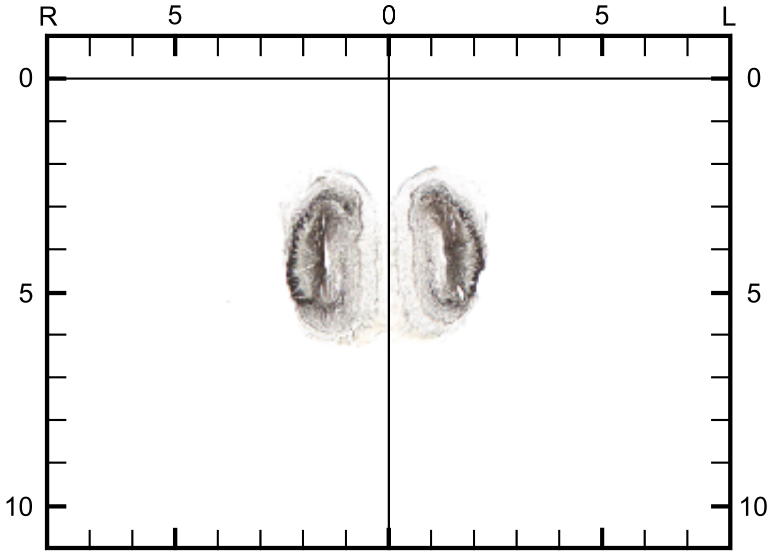

myelin

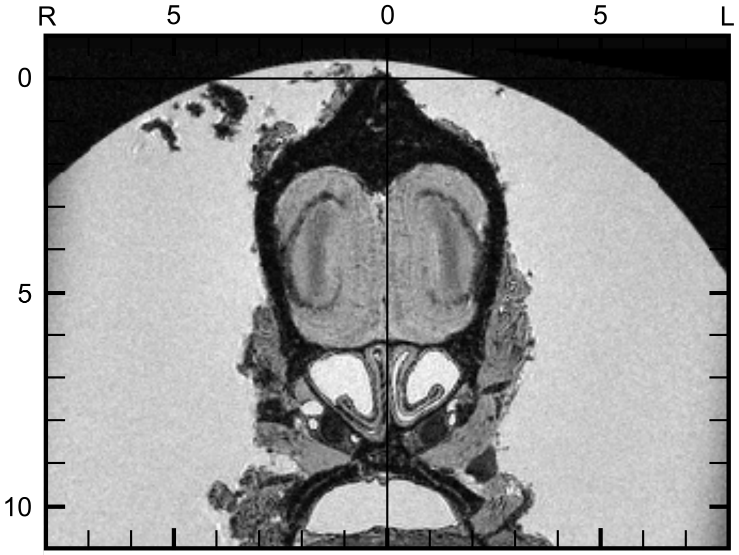

post mortem MR, 30 days

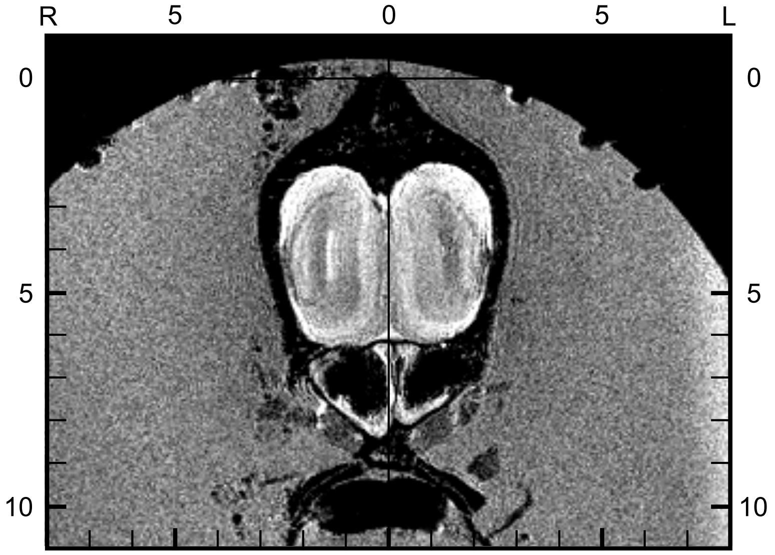

post mortem MR, 48 h

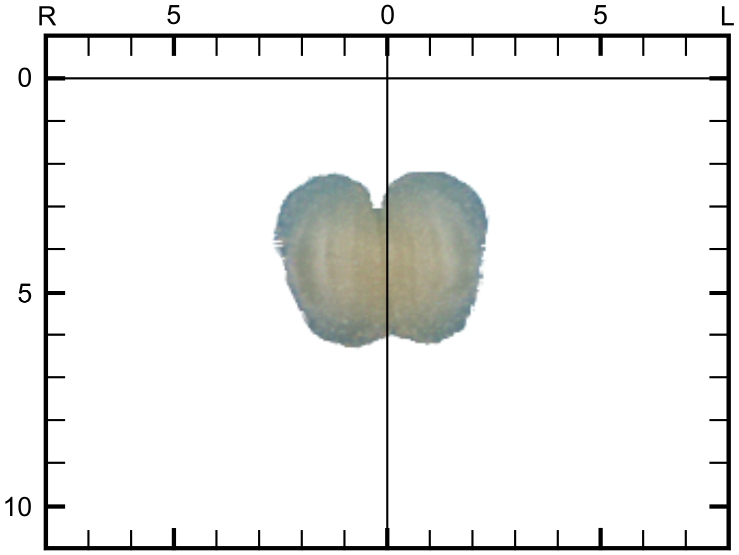

blockface image

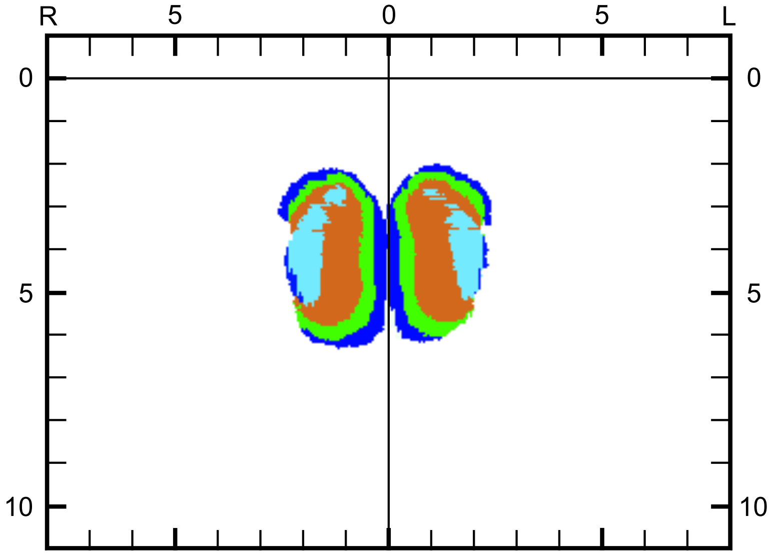

segmentation

Coronal cross-section 12
12.0 mm rostral from bregma

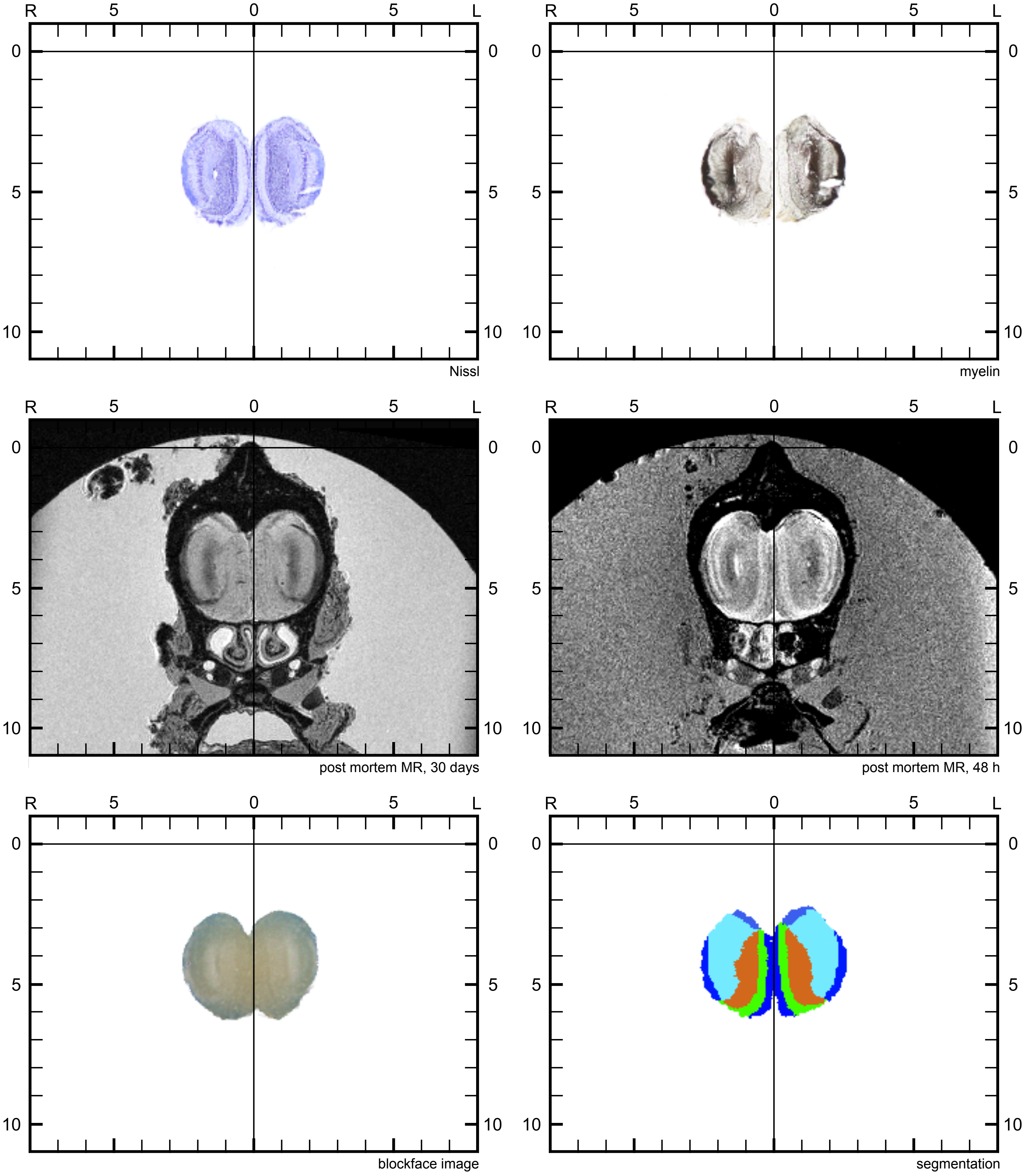

Coronal cross-section 13
11.5 mm rostral from bregma

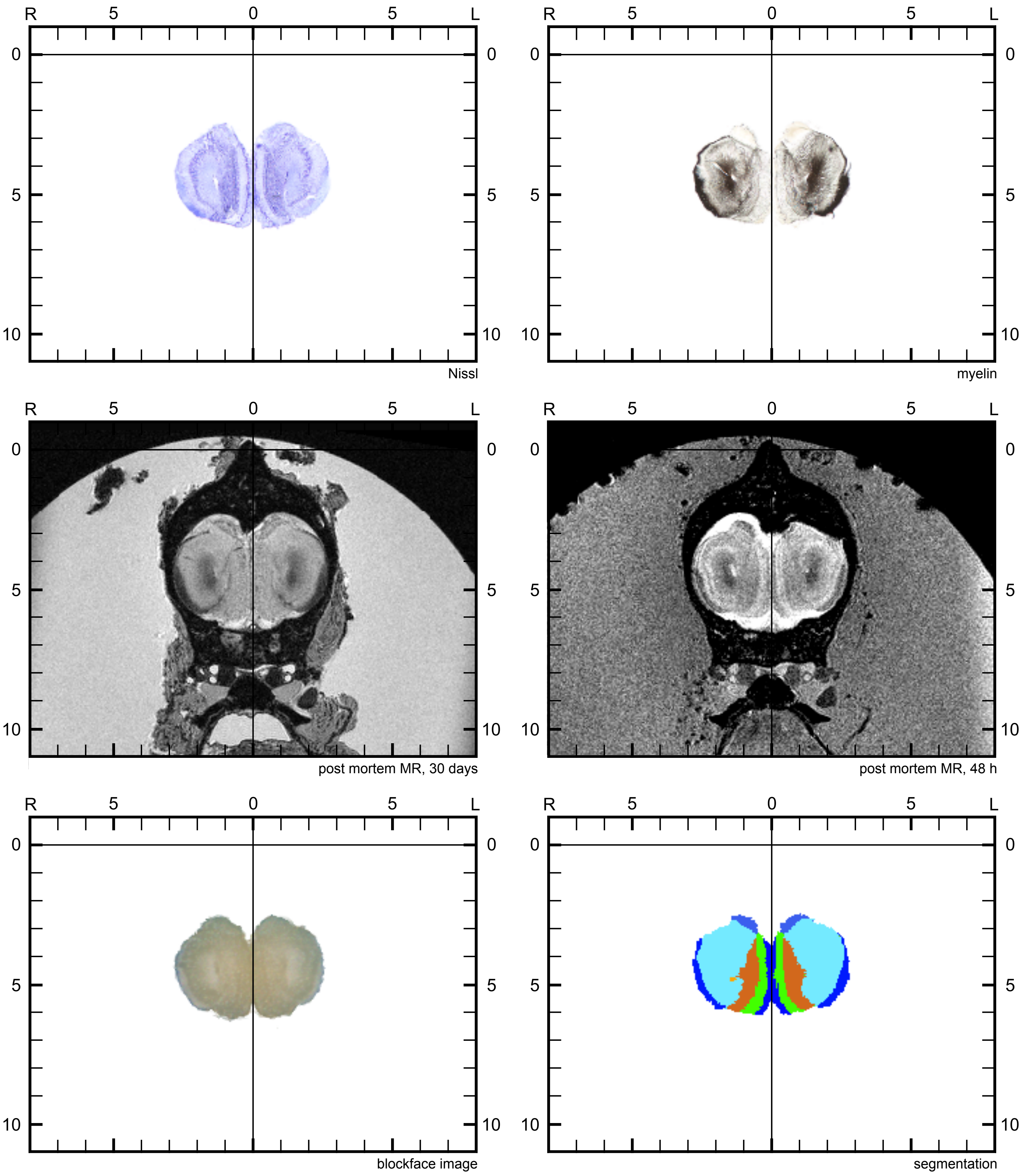

Coronal cross-section 14
11.0 mm rostral from bregma

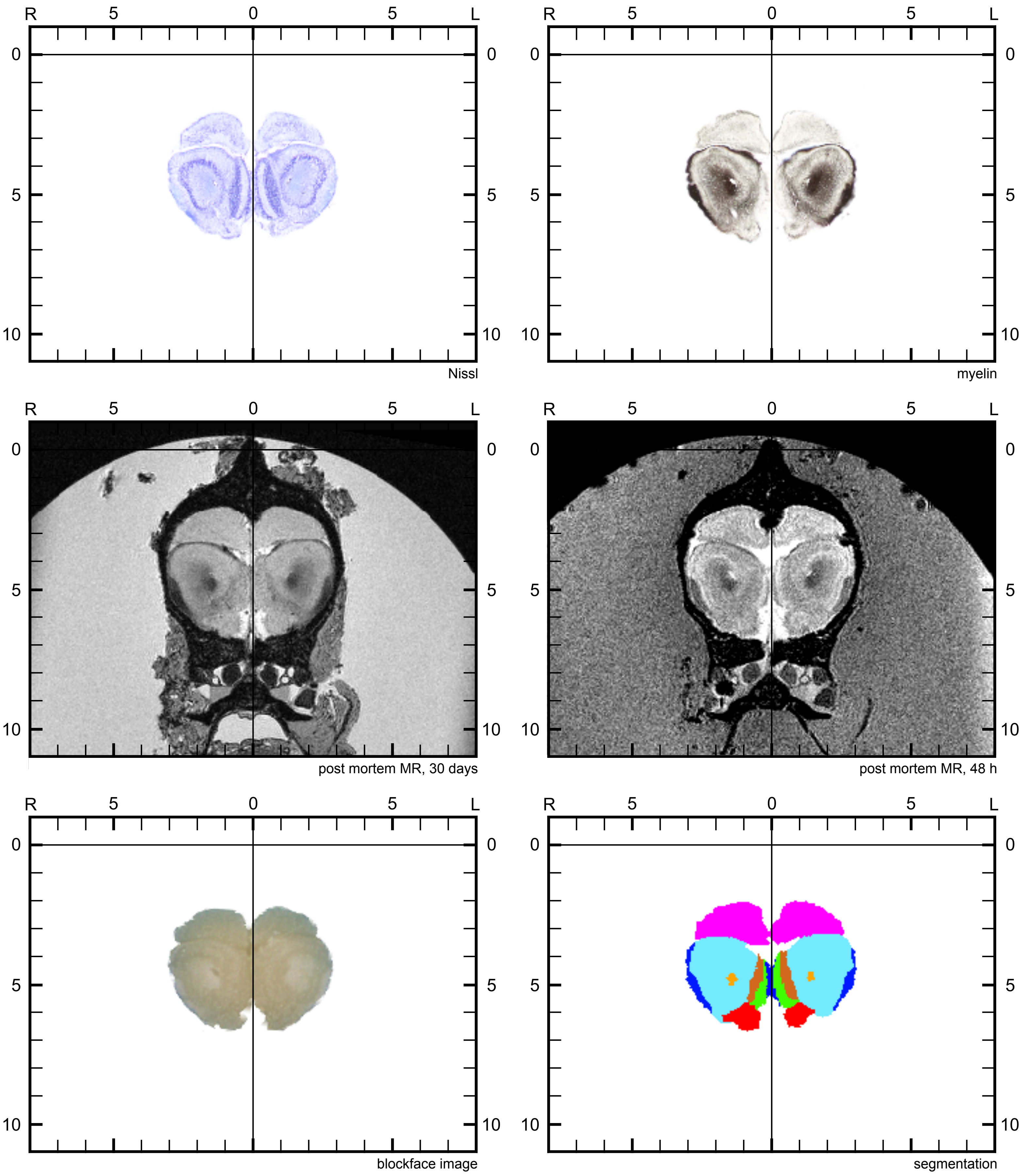

Coronal cross-section 15
10.5 mm rostral from bregma

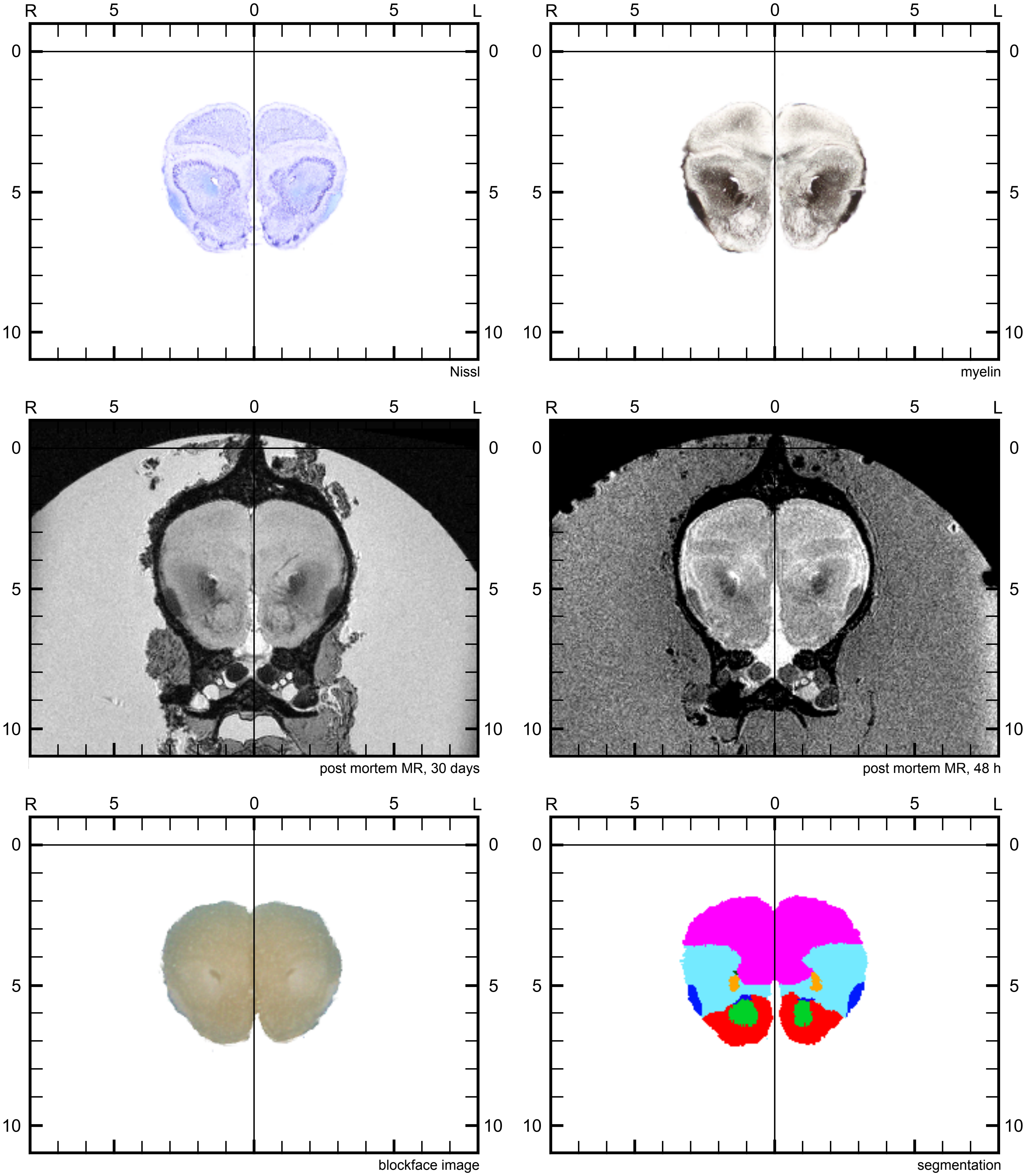

Coronal cross-section 16
10.0 mm rostral from bregma

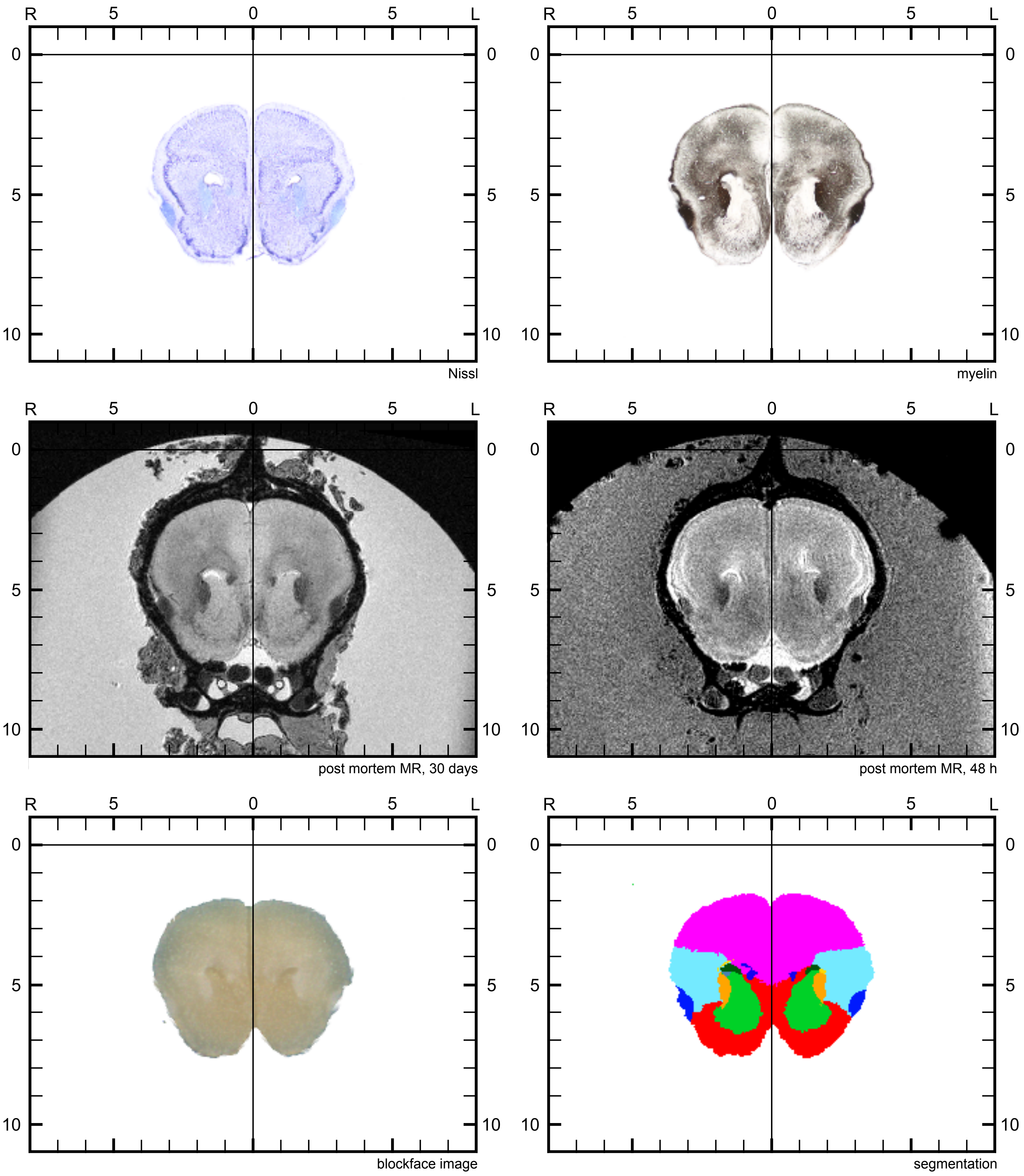

Coronal cross-section 17
9.5 mm rostral from bregma

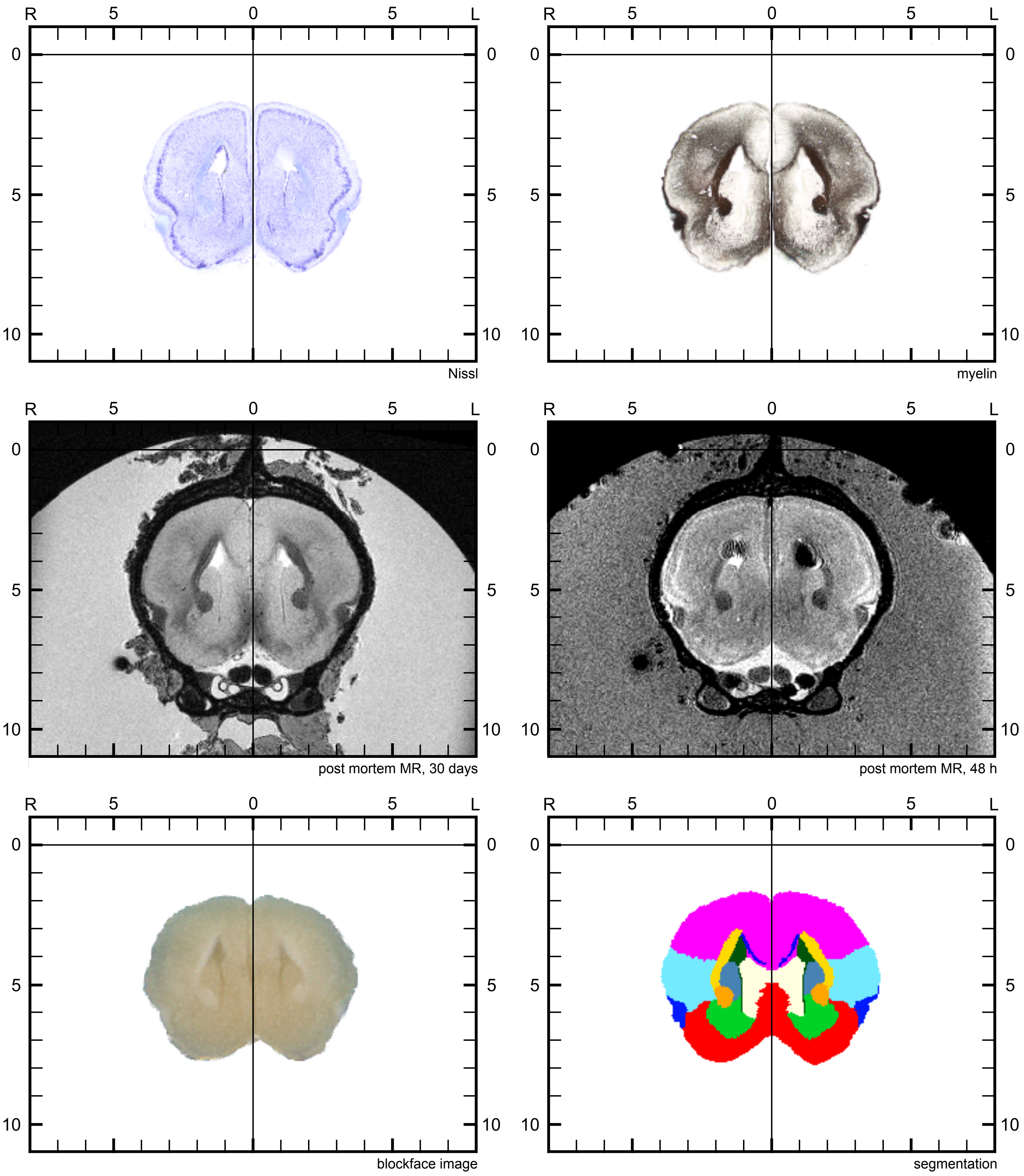

Coronal cross-section 18
9.0 mm rostral from bregma

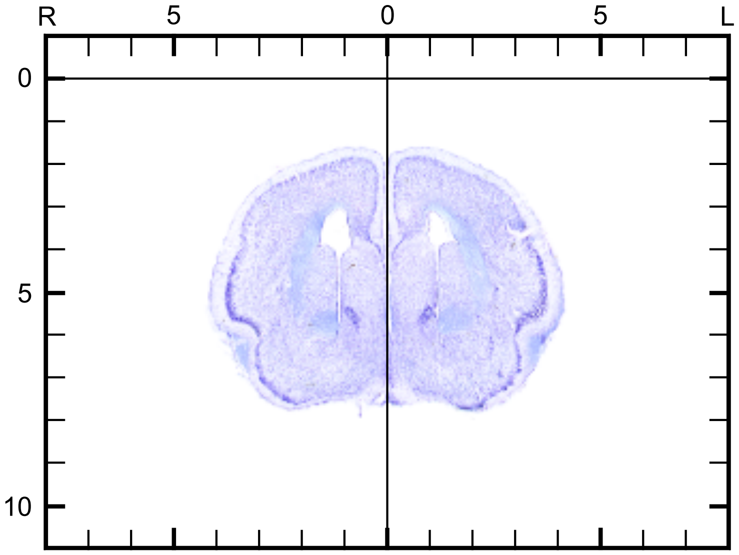

Nissl

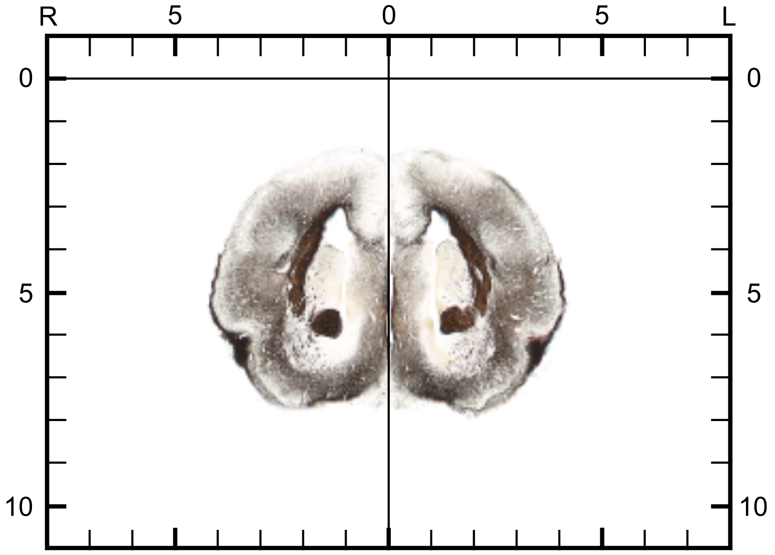

myelin

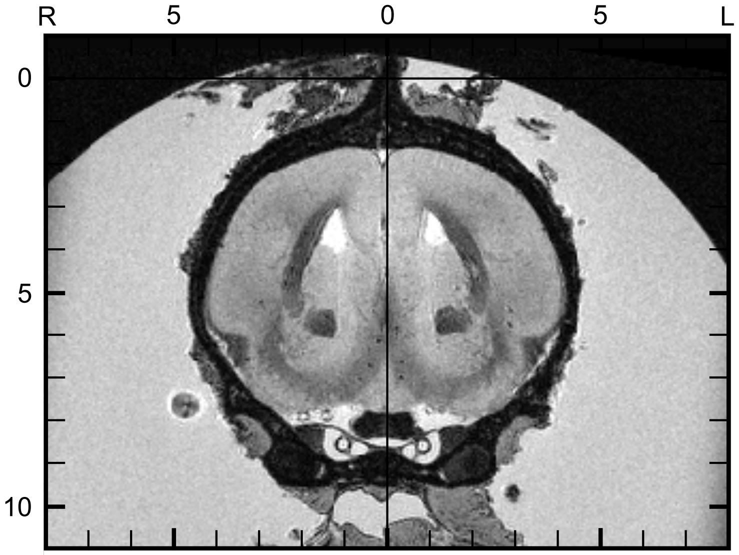

post mortem MR, 30 days

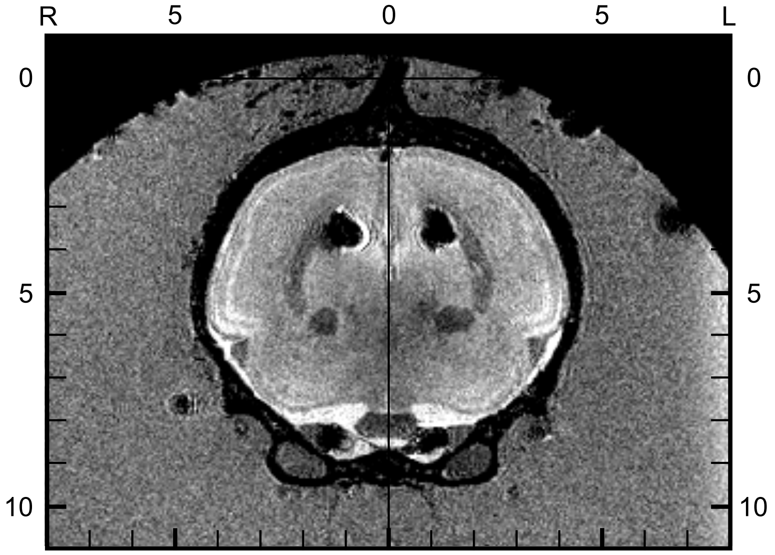

post mortem MR, 48 h

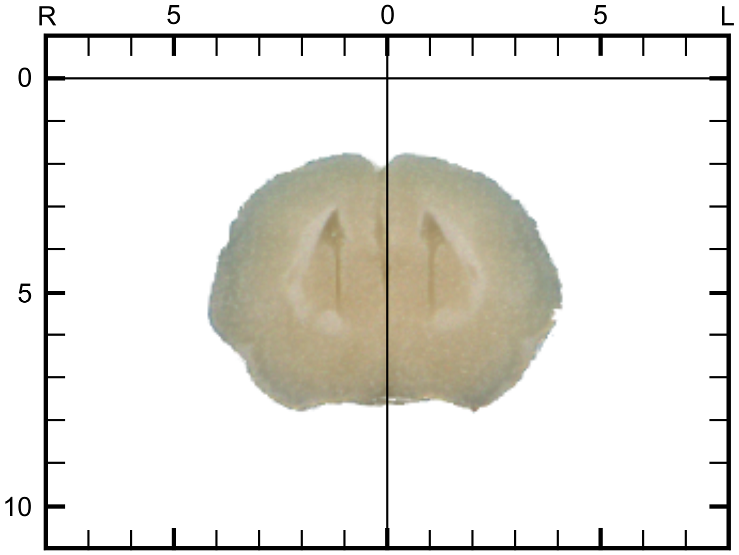

blockface image

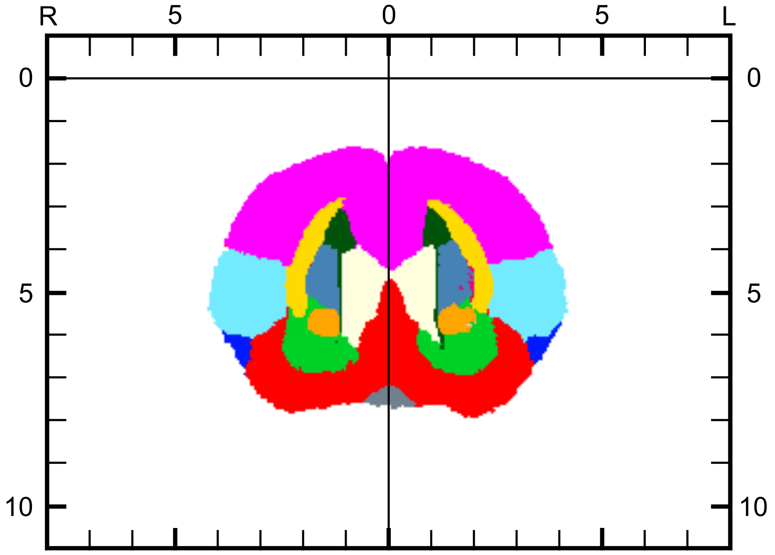

segmentation

Coronal cross-section 19
8.5 mm rostral from bregma

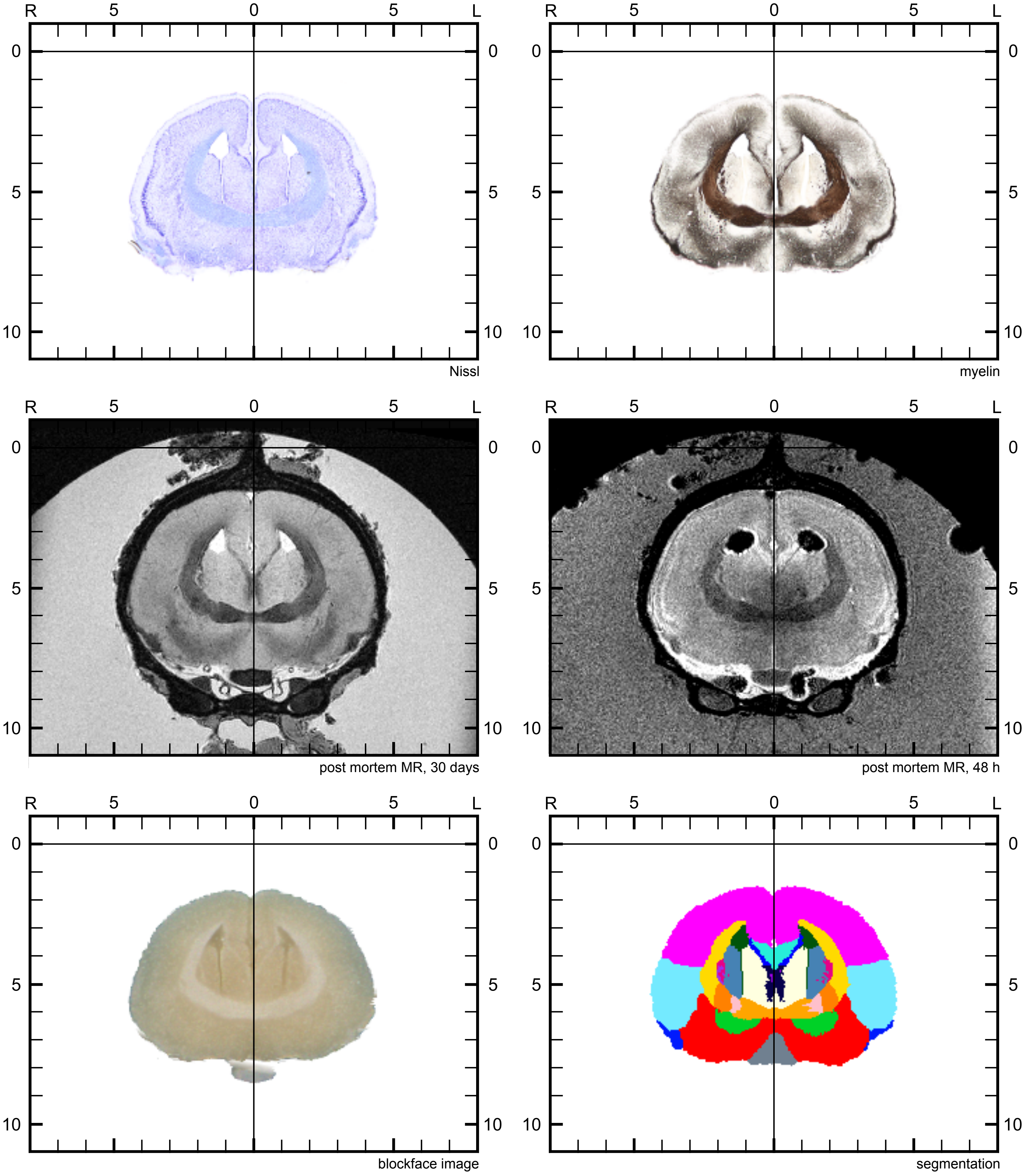

Coronal cross-section 20
8.0 mm rostral from bregma

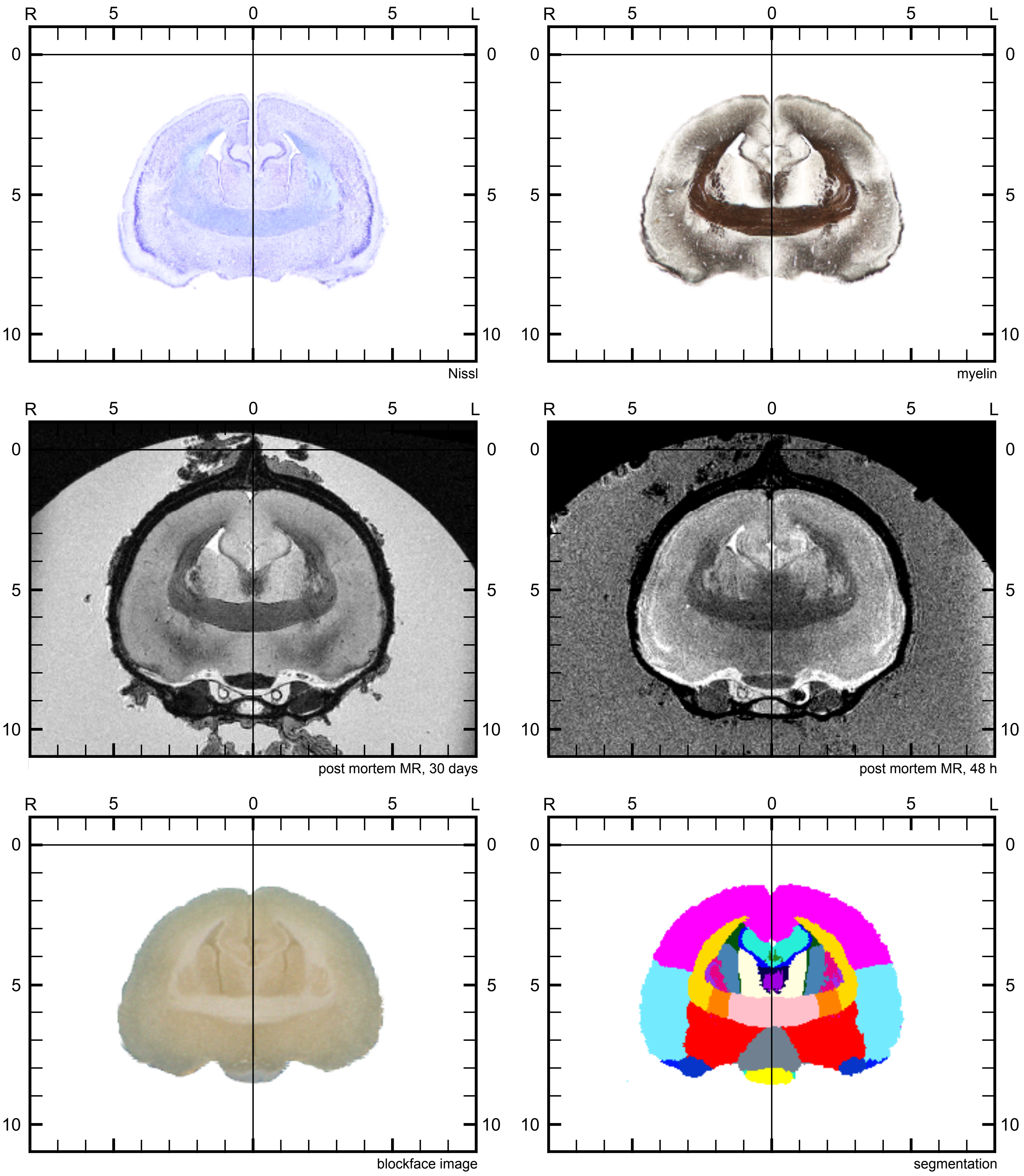

Coronal cross-section 21
7.5 mm rostral from bregma

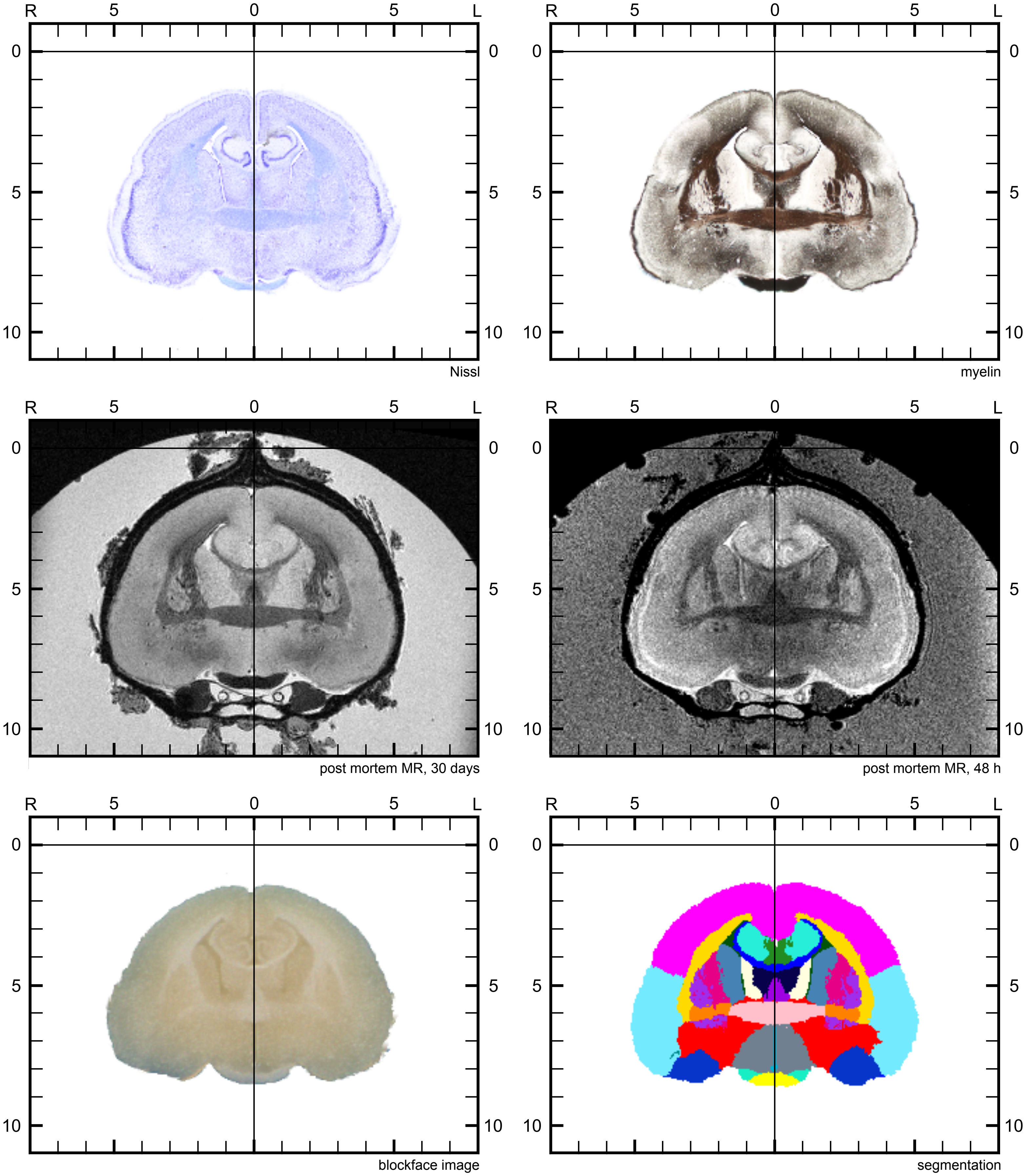

Coronal cross-section 22
7.0 mm rostral from bregma

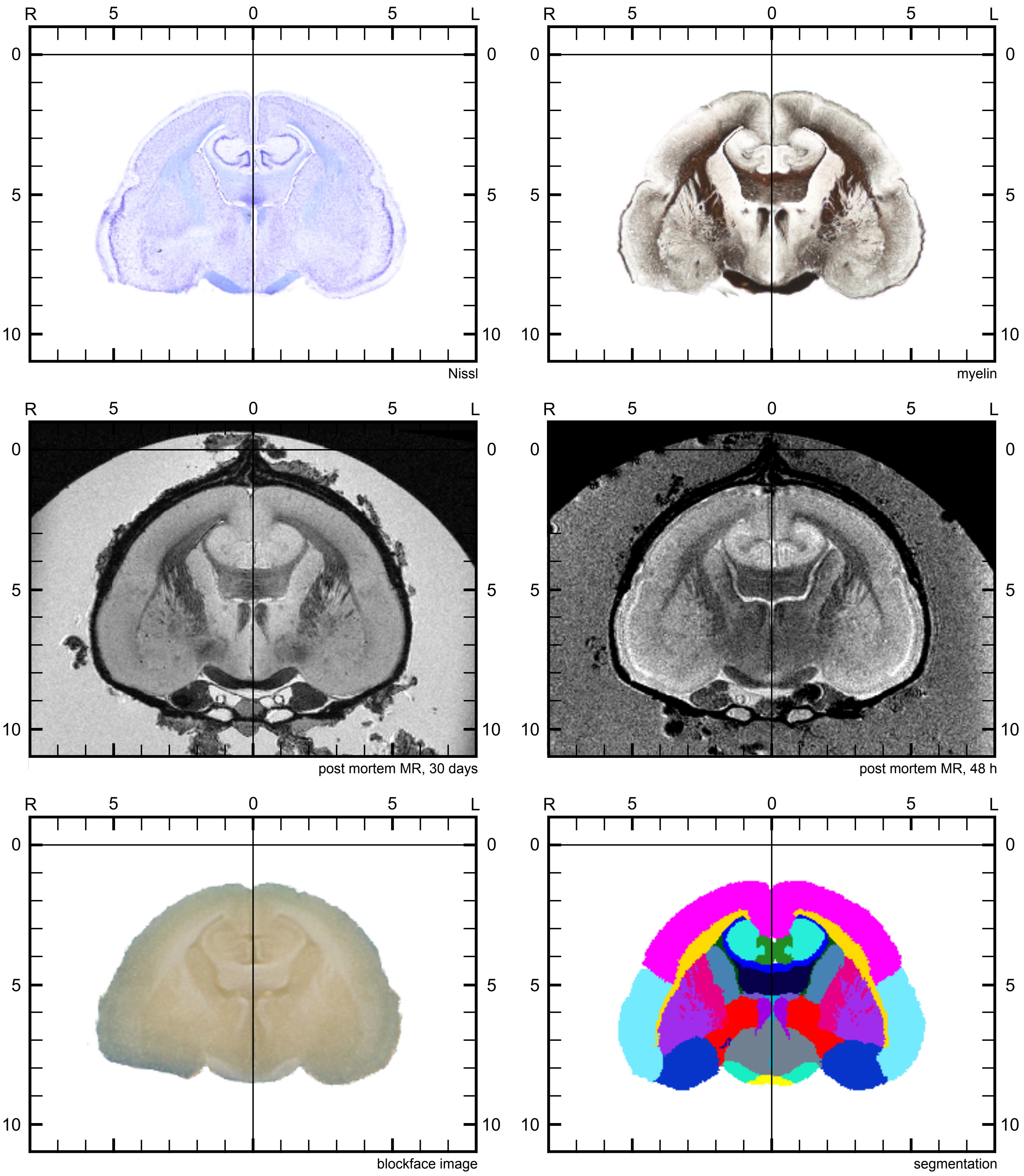

Coronal cross-section 23
6.5 mm rostral from bregma

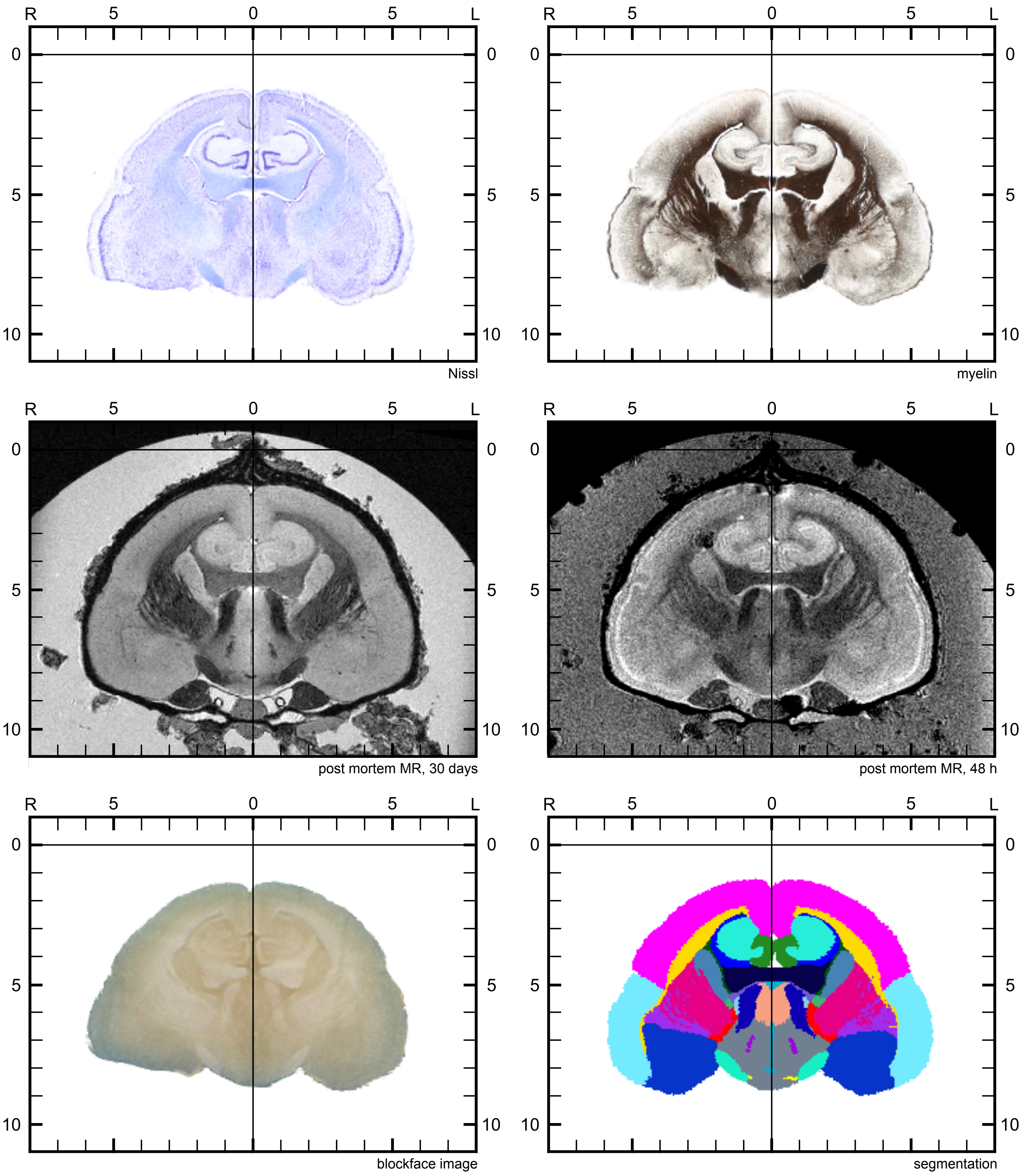

Coronal cross-section 24
6.0 mm rostral from bregma

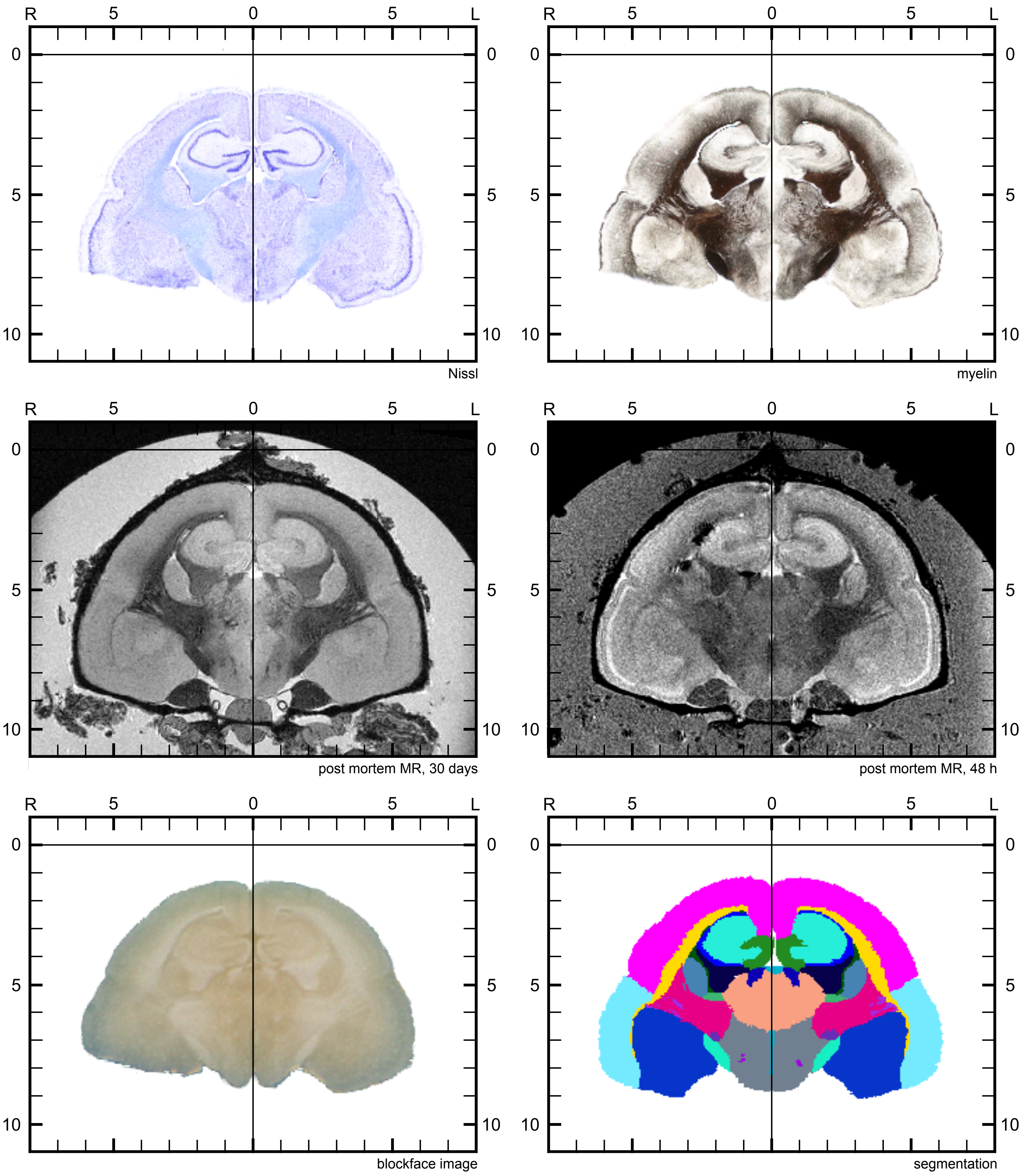

Coronal cross-section 25
5.5 mm rostral from bregma

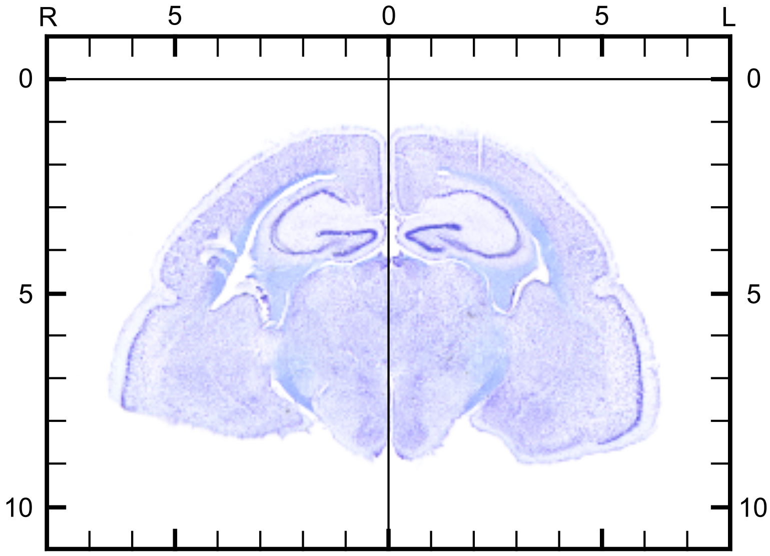

Nissl

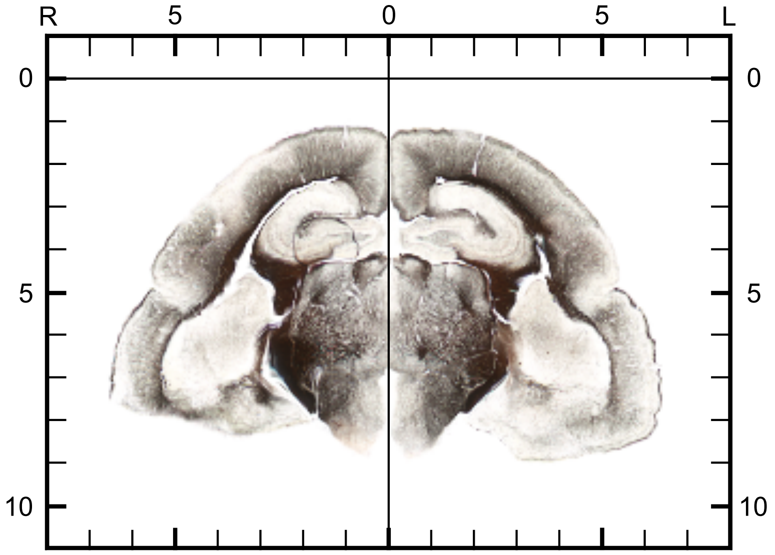

myelin

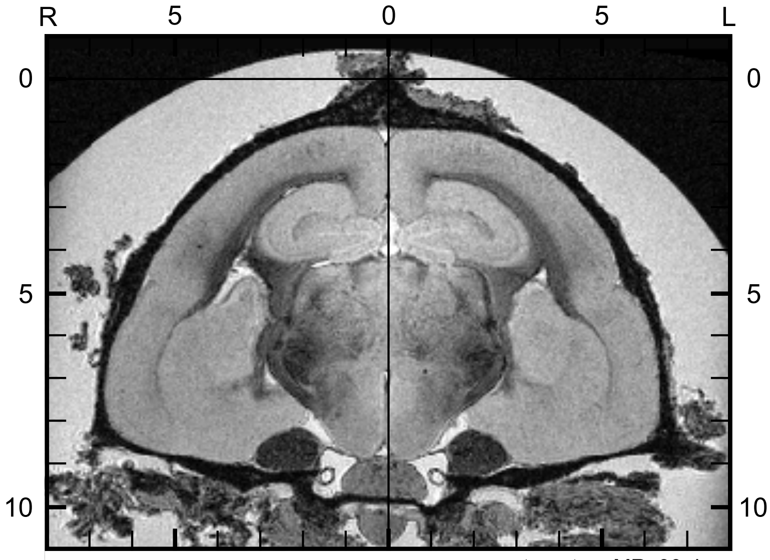

post mortem MR, 30 days

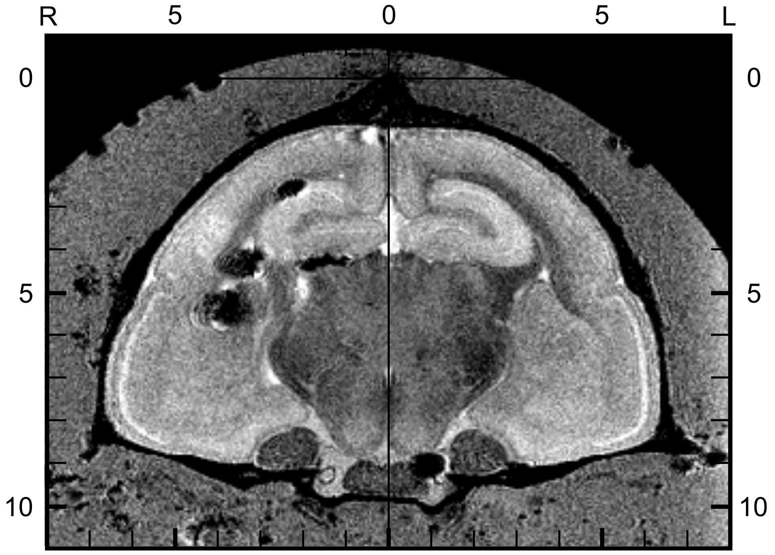

post mortem MR, 48 h

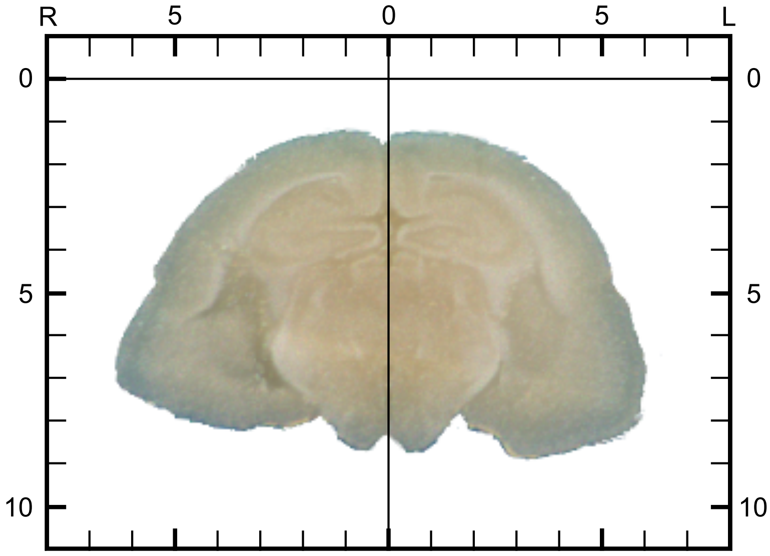

blockface image

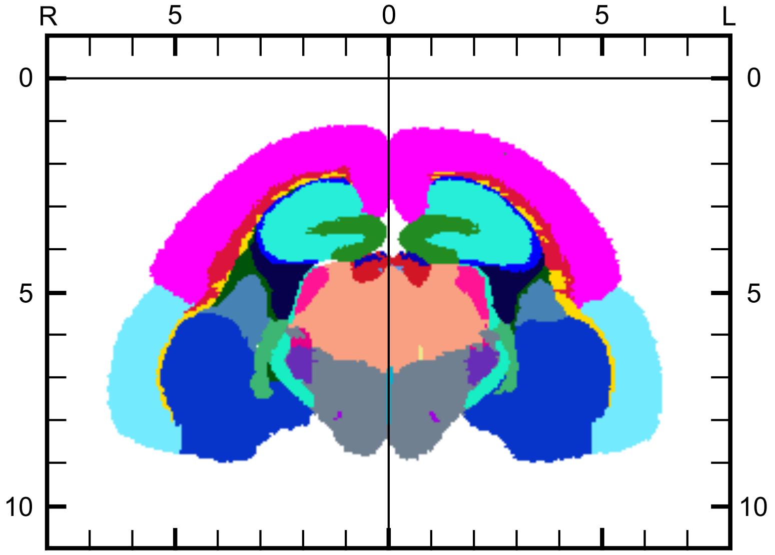

segmentation

Coronal cross-section 26
5.0 mm rostral from bregma

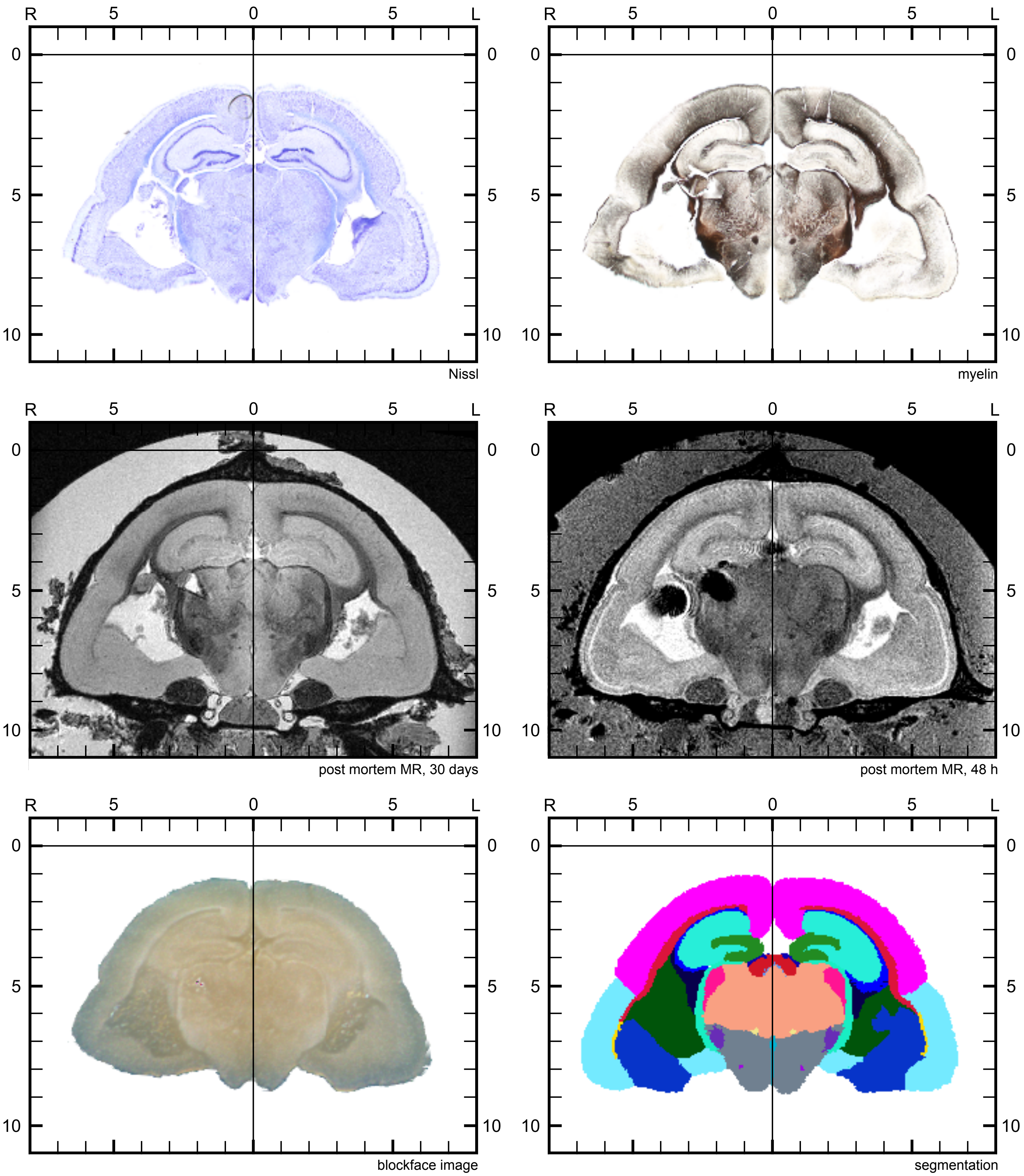

Coronal cross-section 27
4.5 mm rostral from bregma

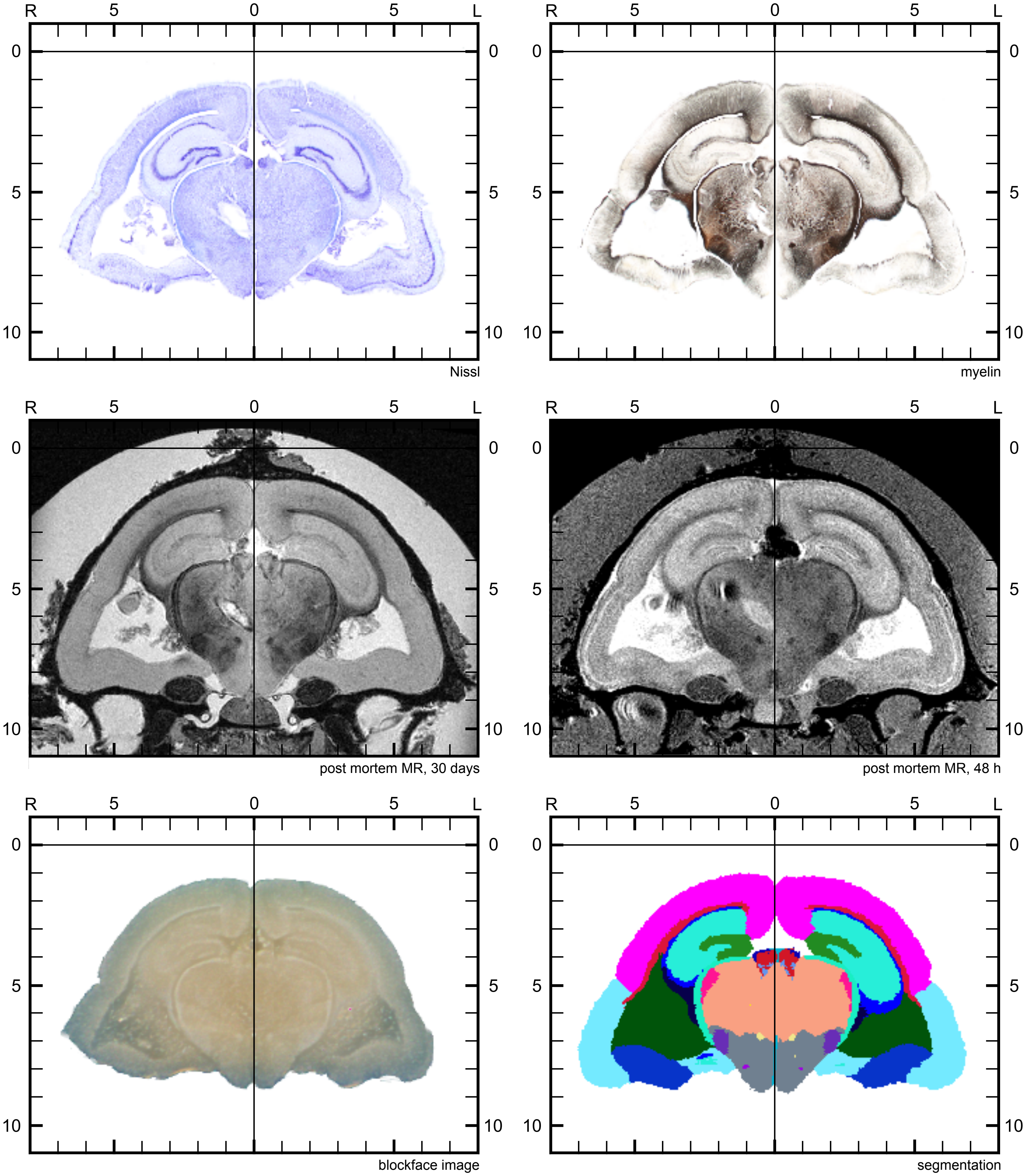

Coronal cross-section 28
4.0 mm rostral from bregma

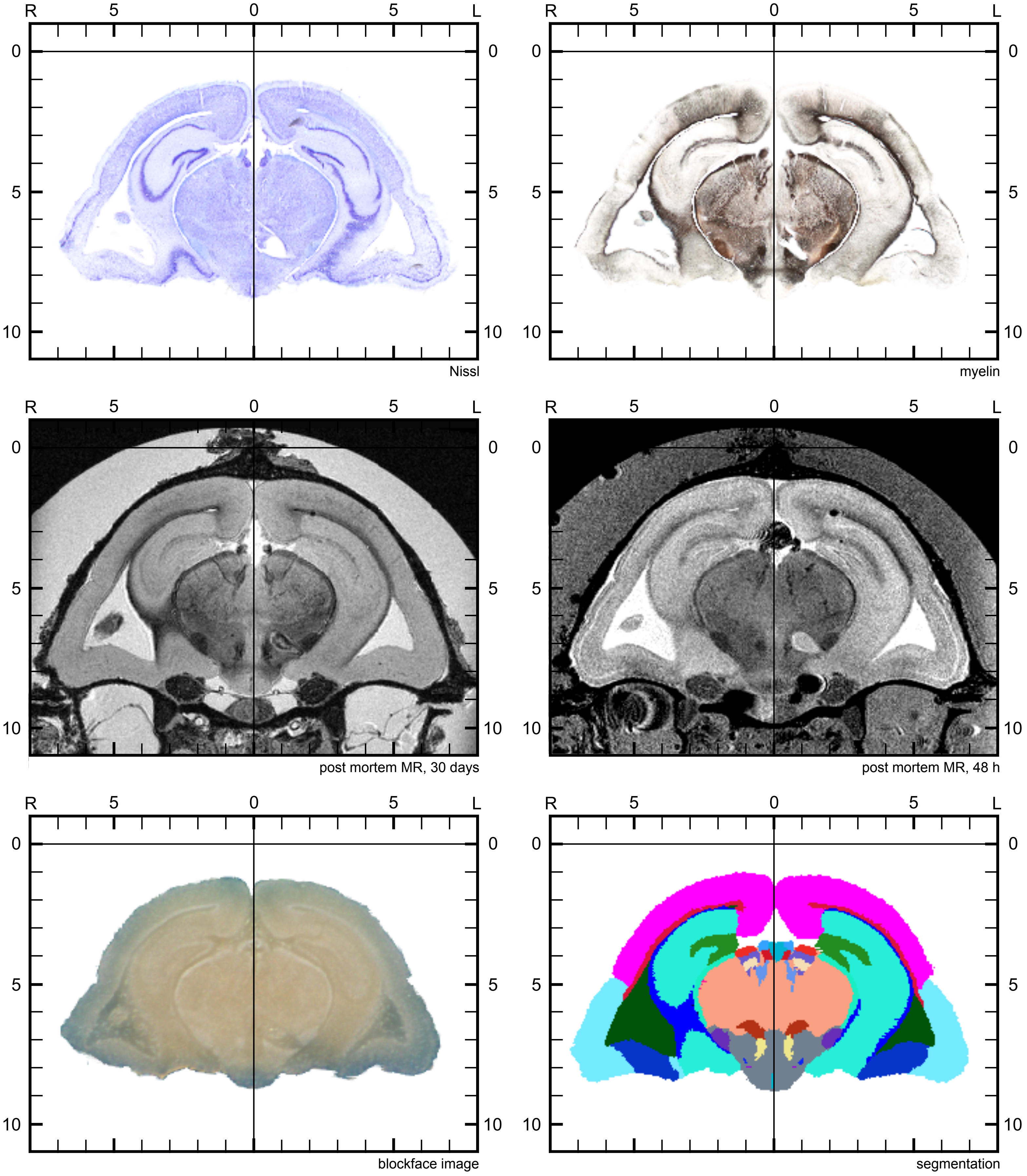

Coronal cross-section 29
3.5 mm rostral from bregma

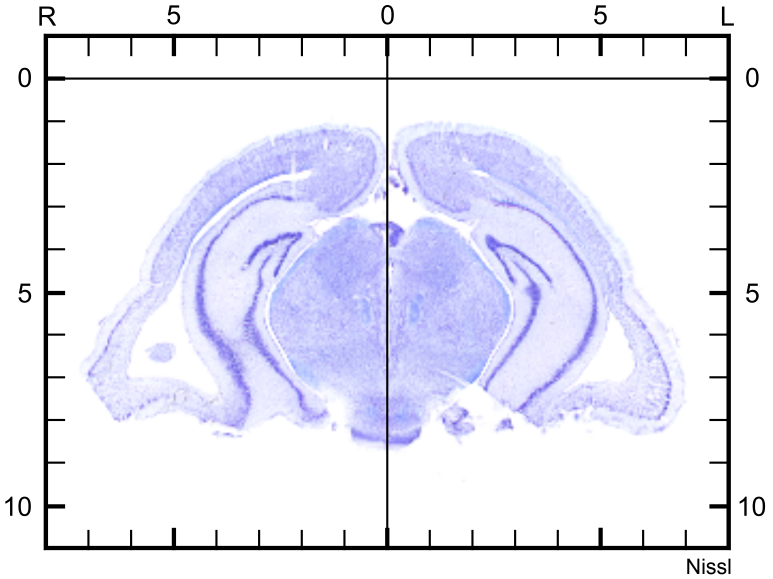

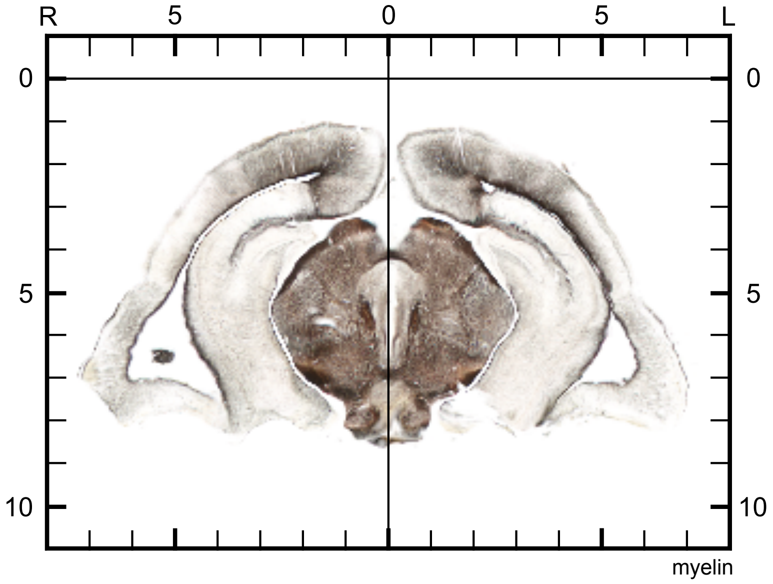

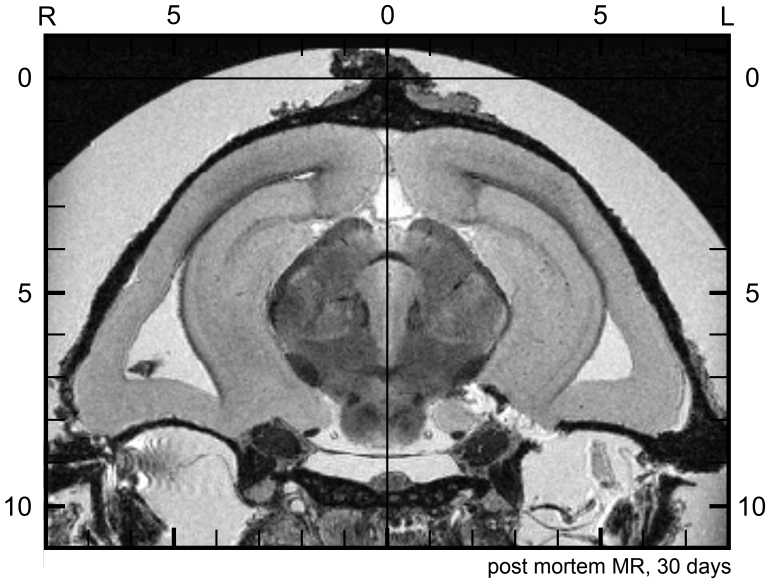

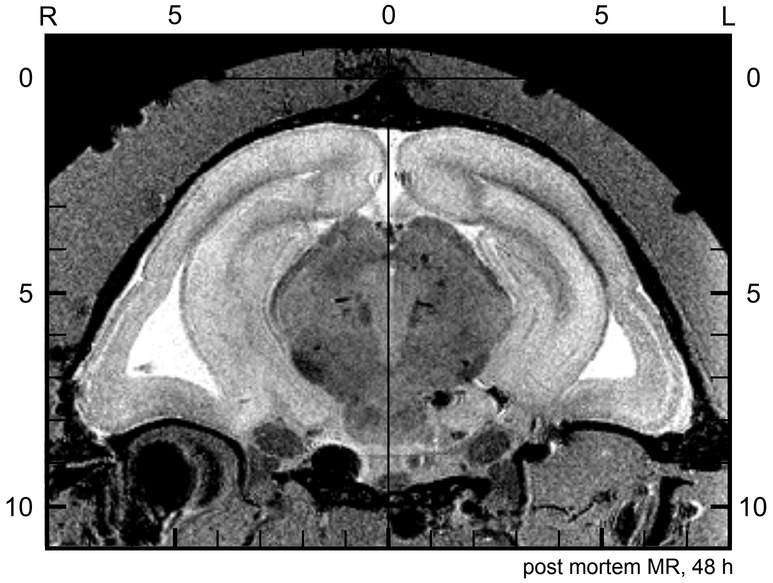

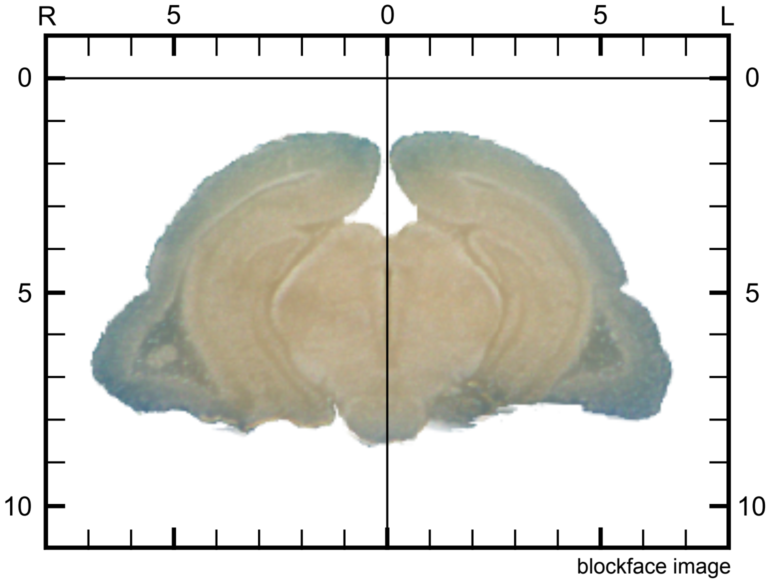

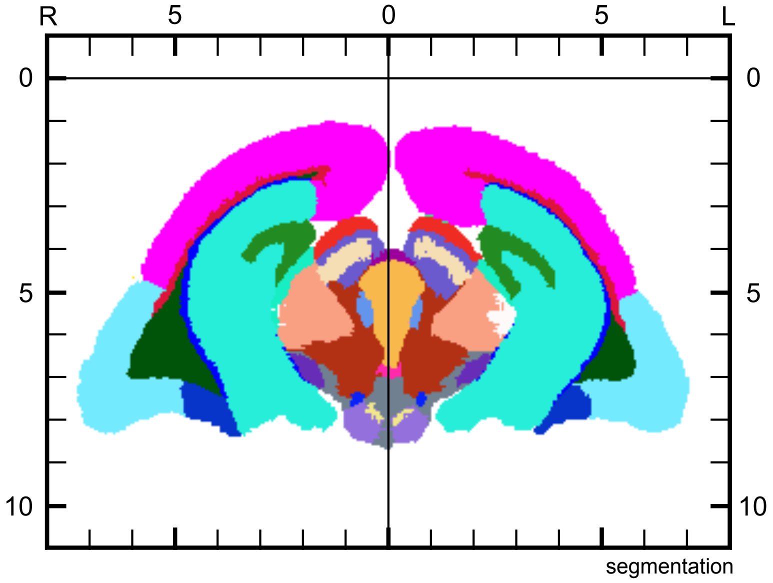

Coronal cross-section 30
3.0 mm rostral from bregma

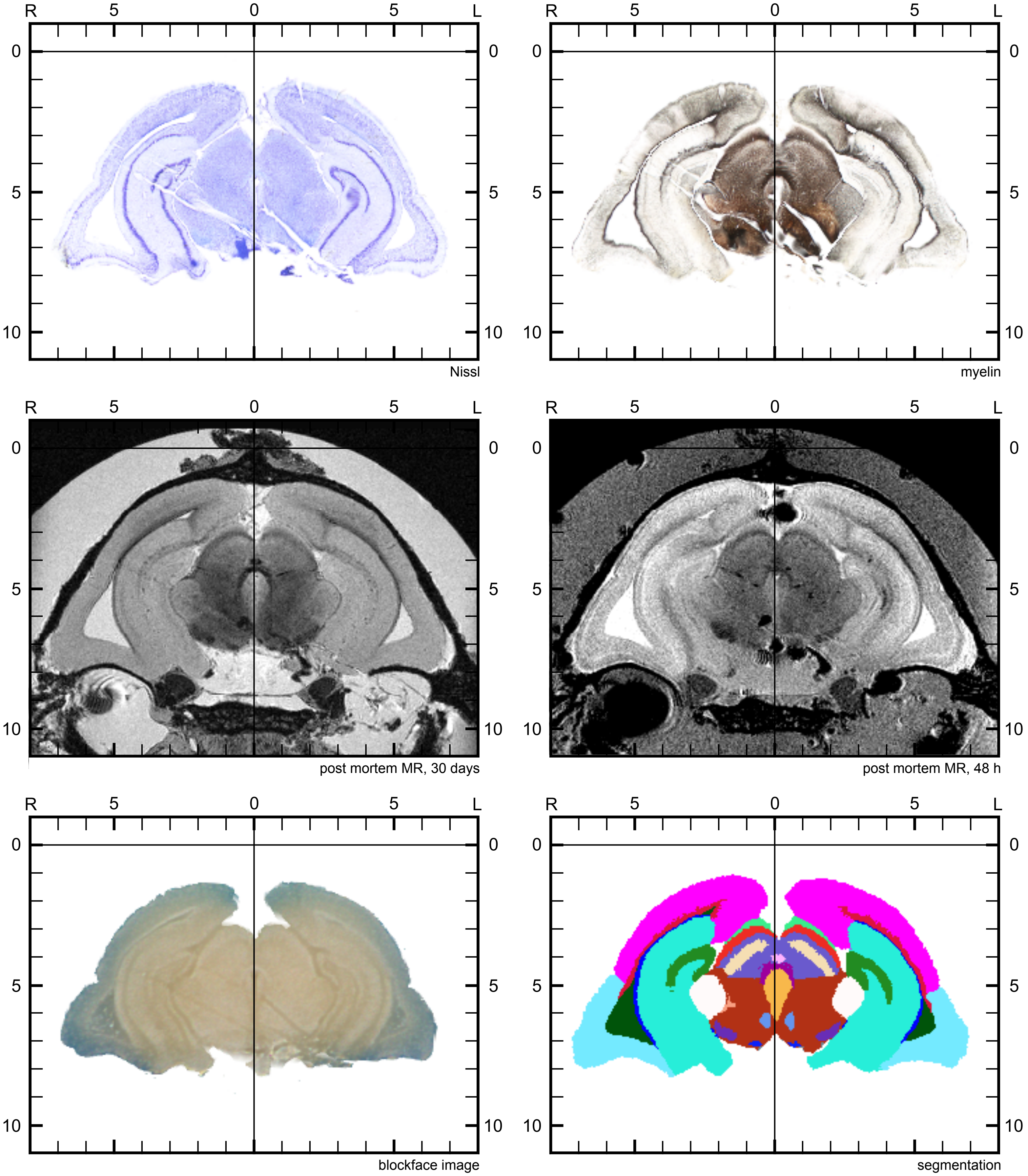

Coronal cross-section 31
2.5 mm rostral from bregma

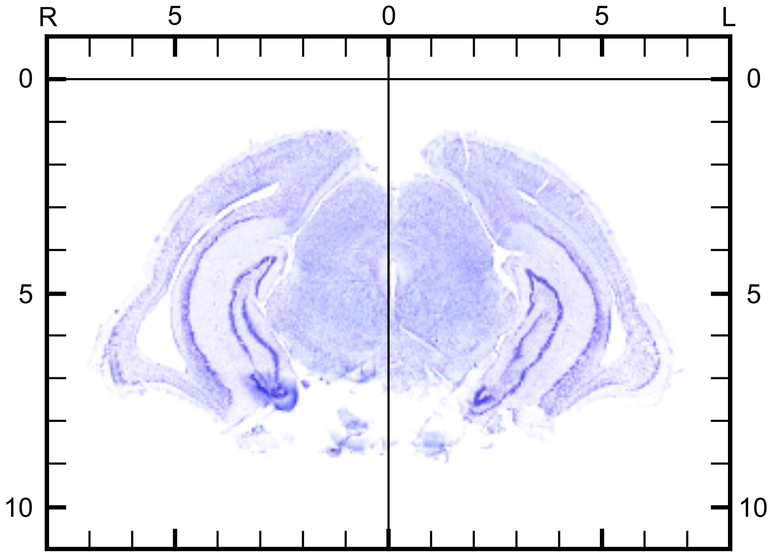

Nissl

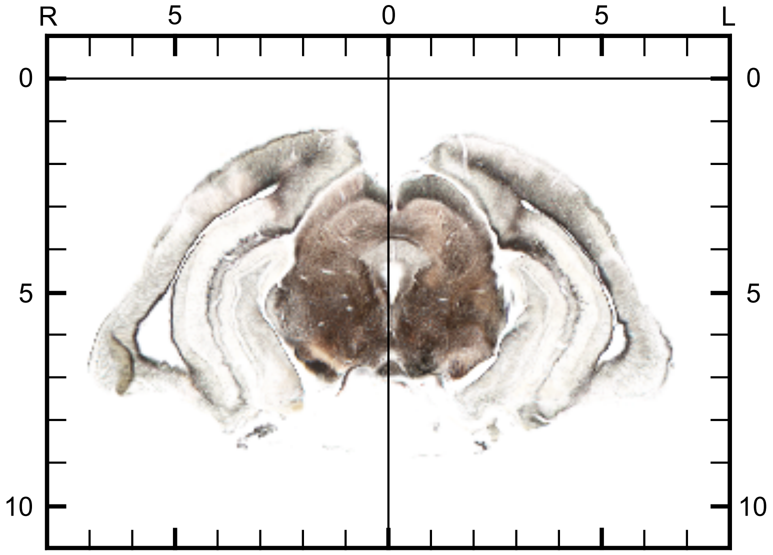

myelin

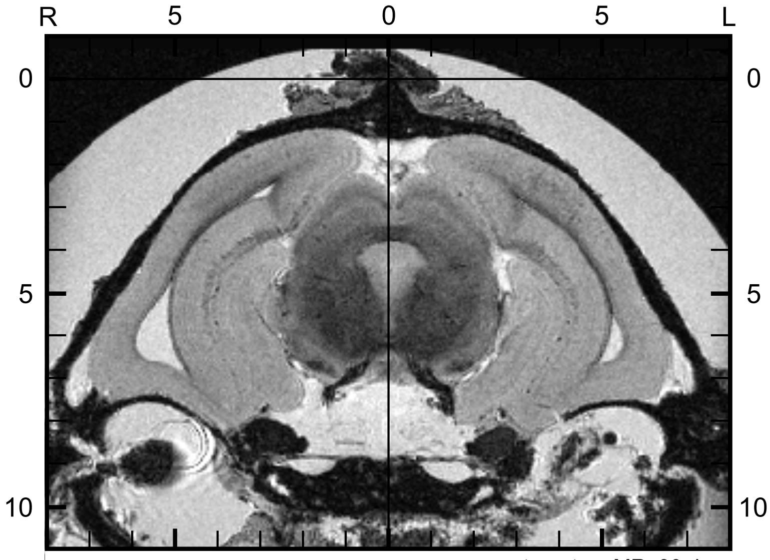

post mortem MR, 30 days

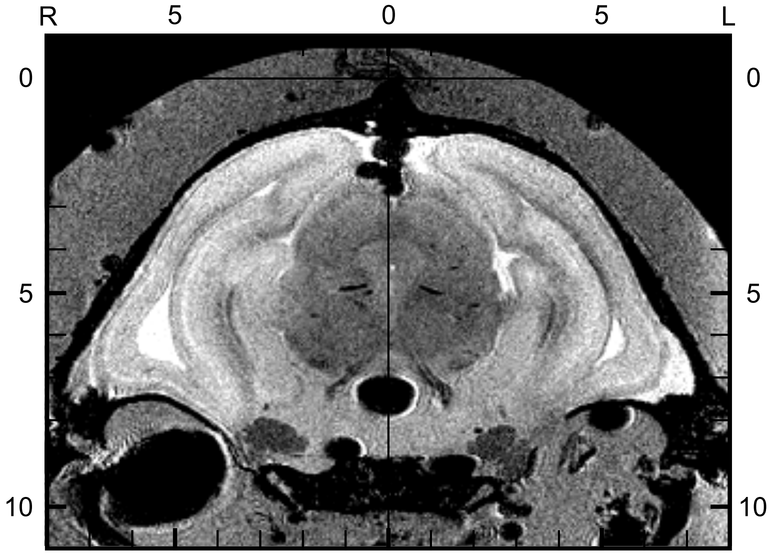

post mortem MR, 48 h

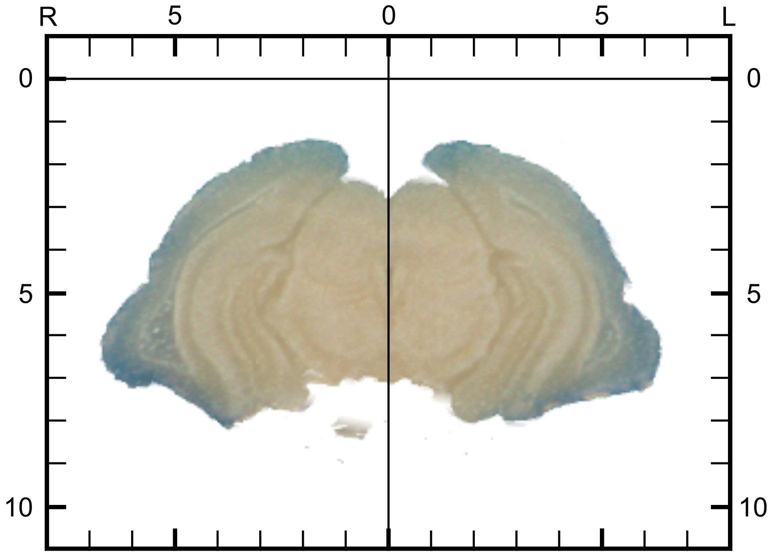

blockface image

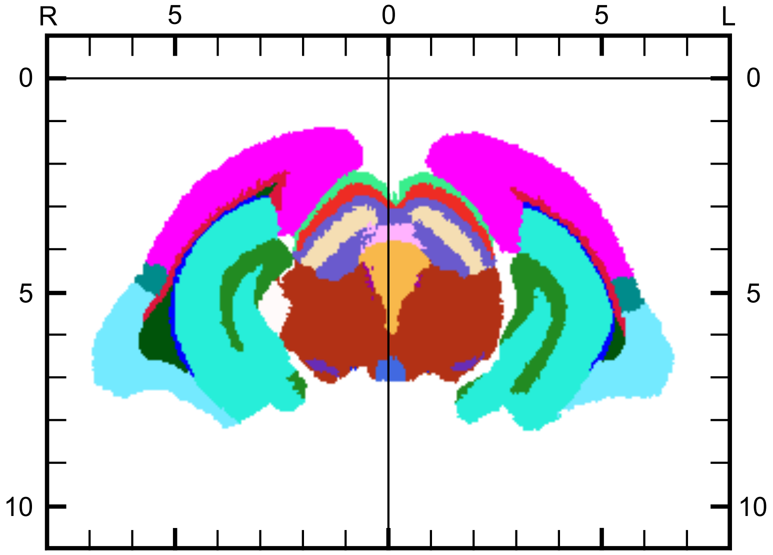

segmentation

Coronal cross-section 32
2.0 mm rostral from bregma

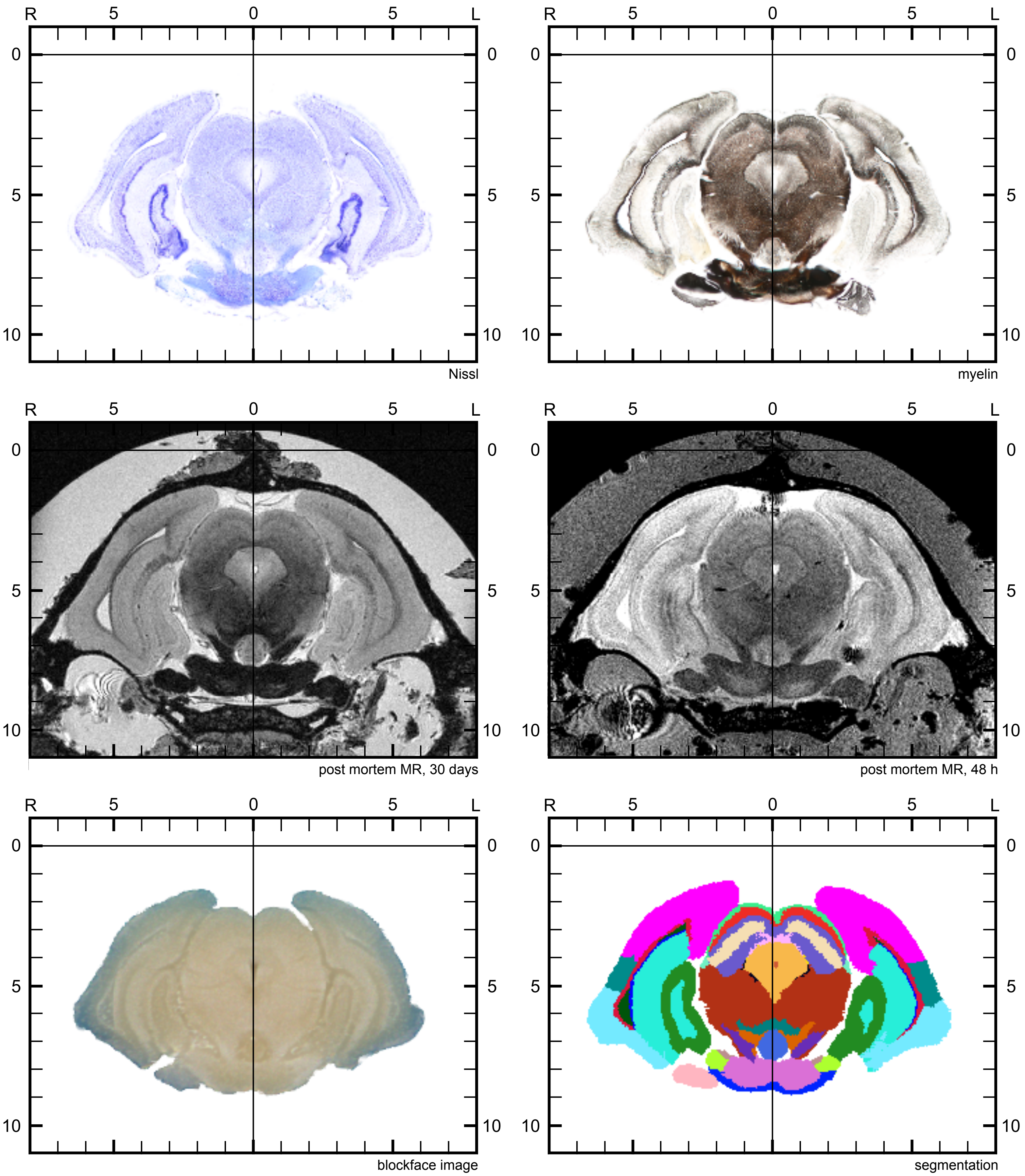

Coronal cross-section 33
1.5 mm rostral from bregma

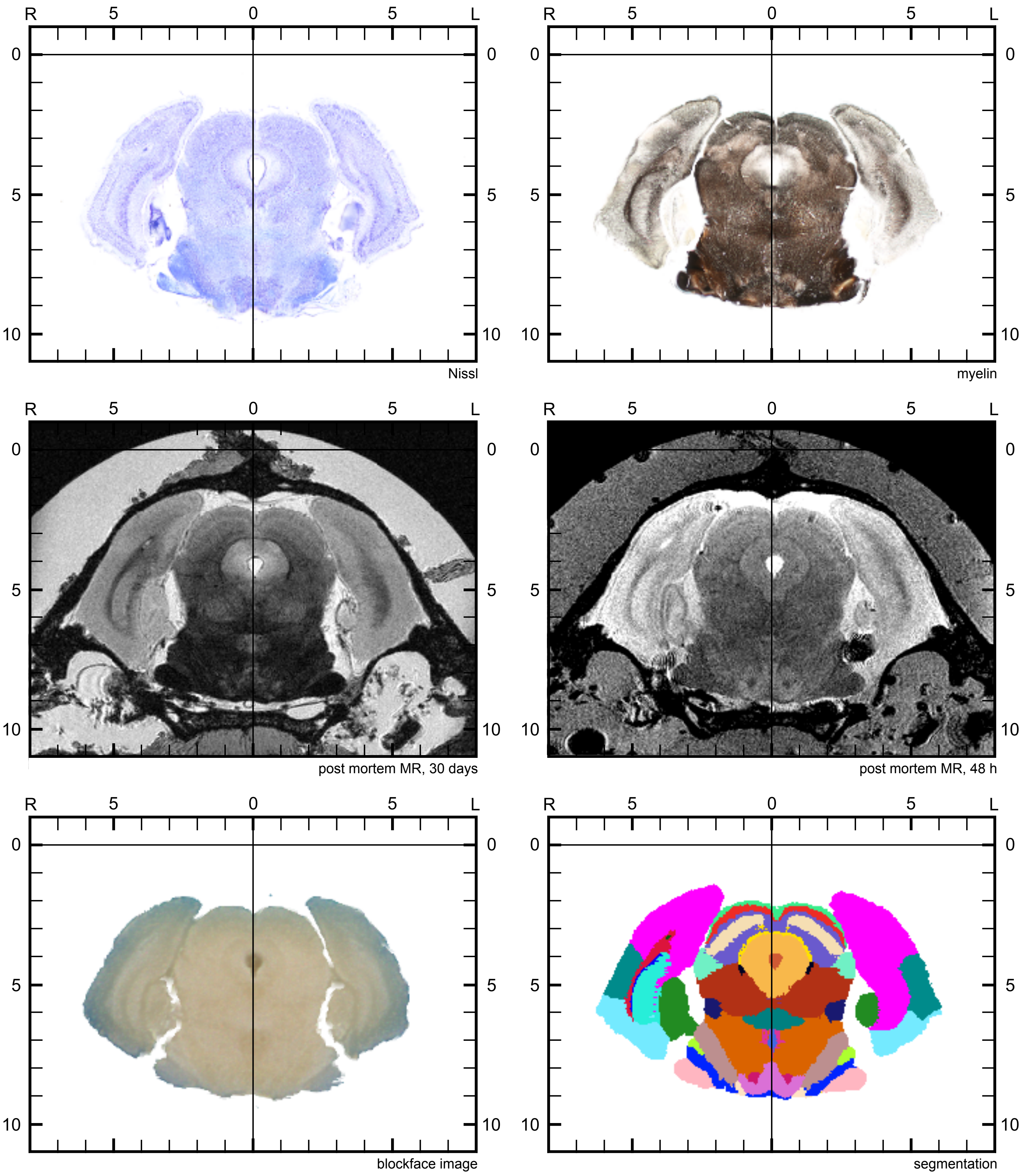

Coronal cross-section 34
1.0 mm rostral from bregma

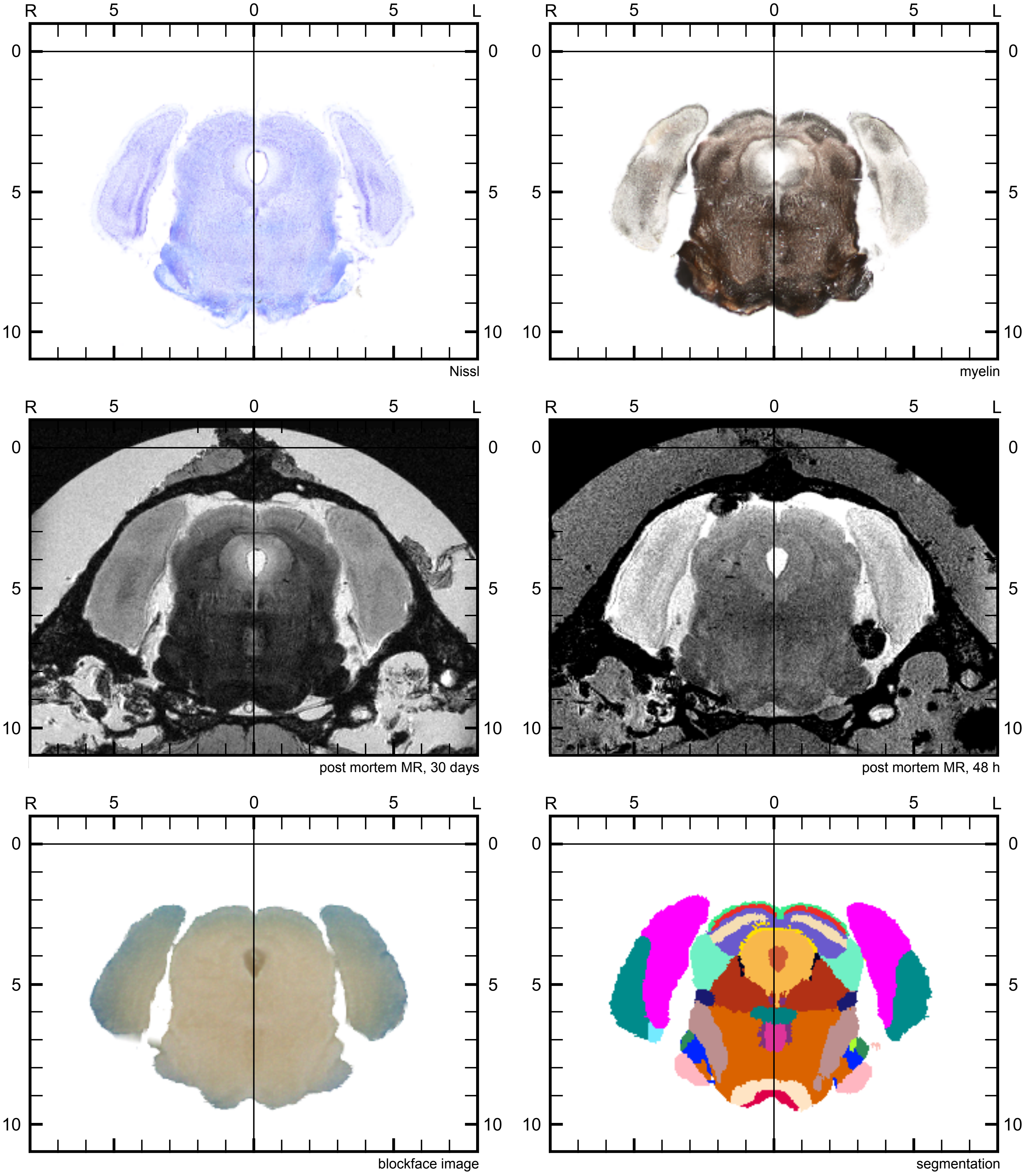

Coronal cross-section 35
0.5 mm rostral from bregma

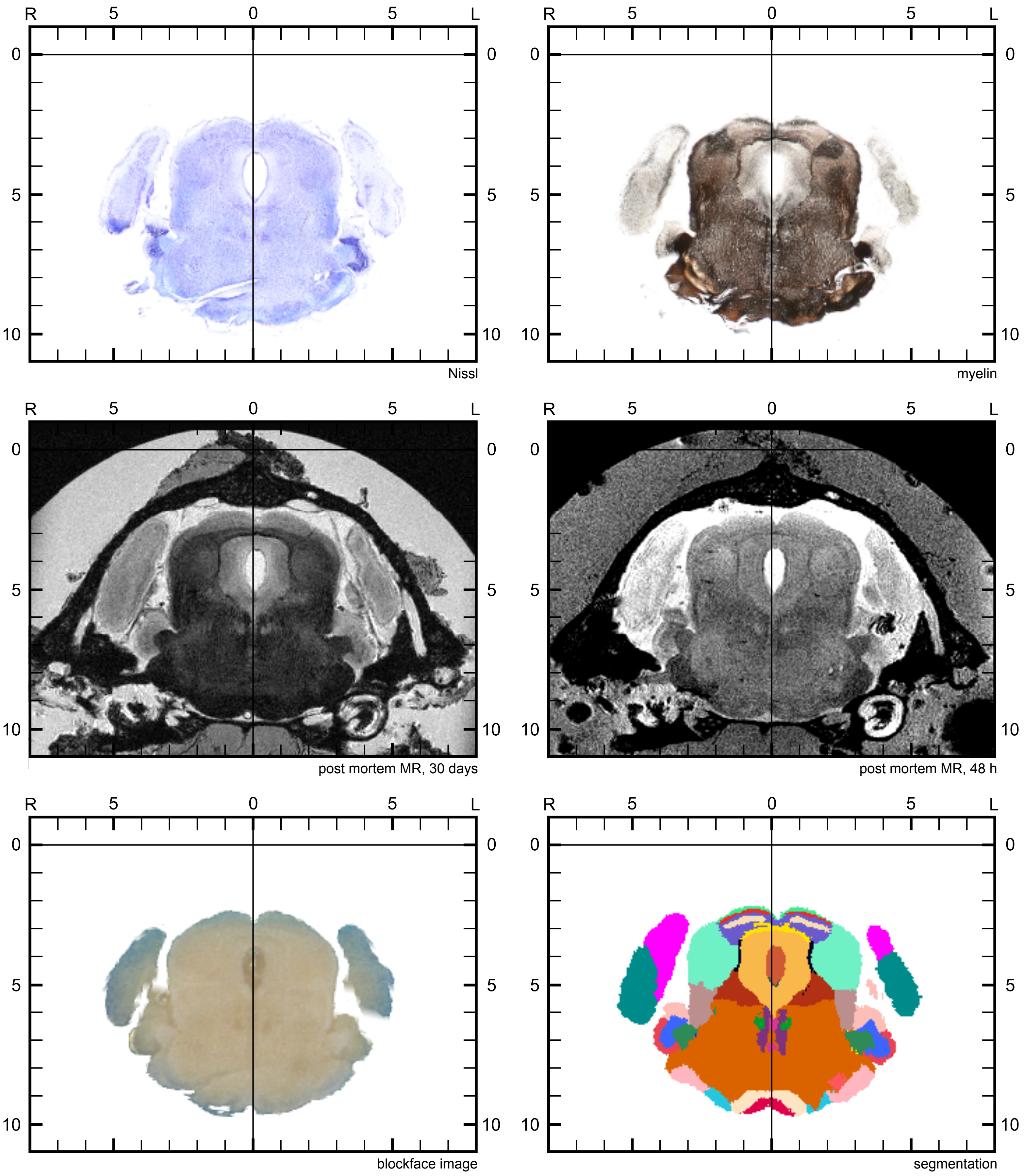

Coronal cross-section 36
bregma level

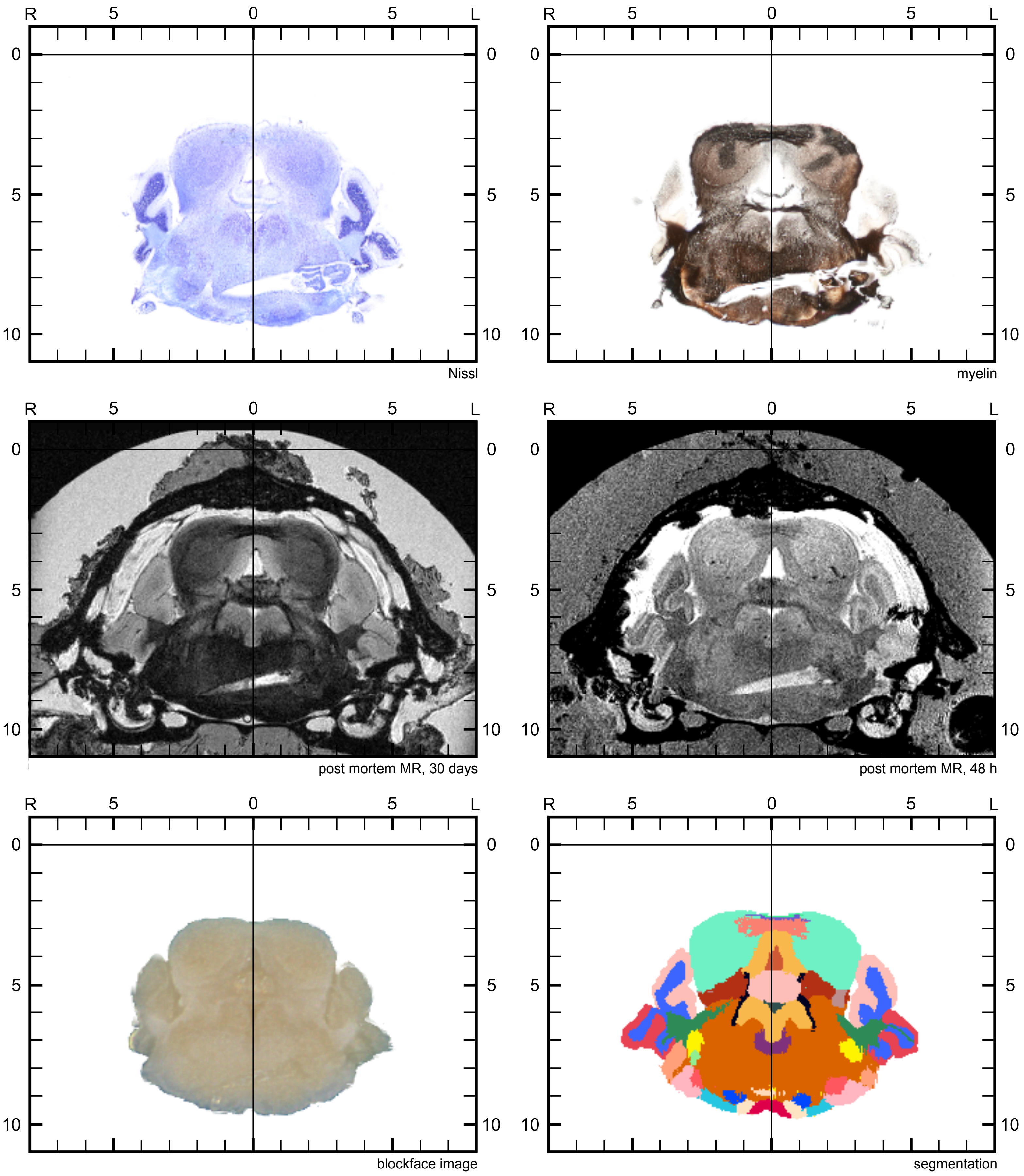

Coronal cross-section 37
0.5 mm caudal from bregma

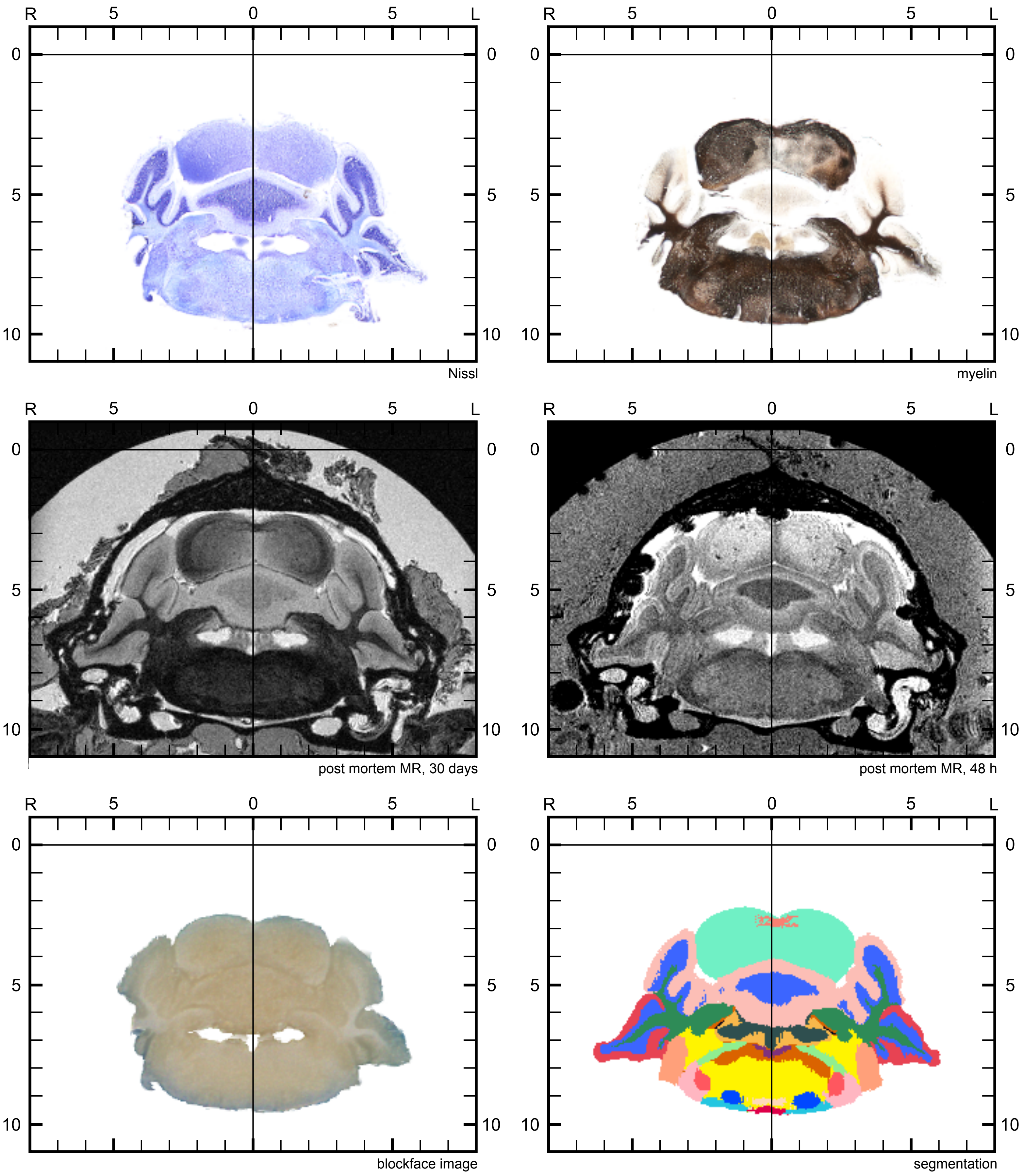

Coronal cross-section 38
1.0 mm caudal from bregma

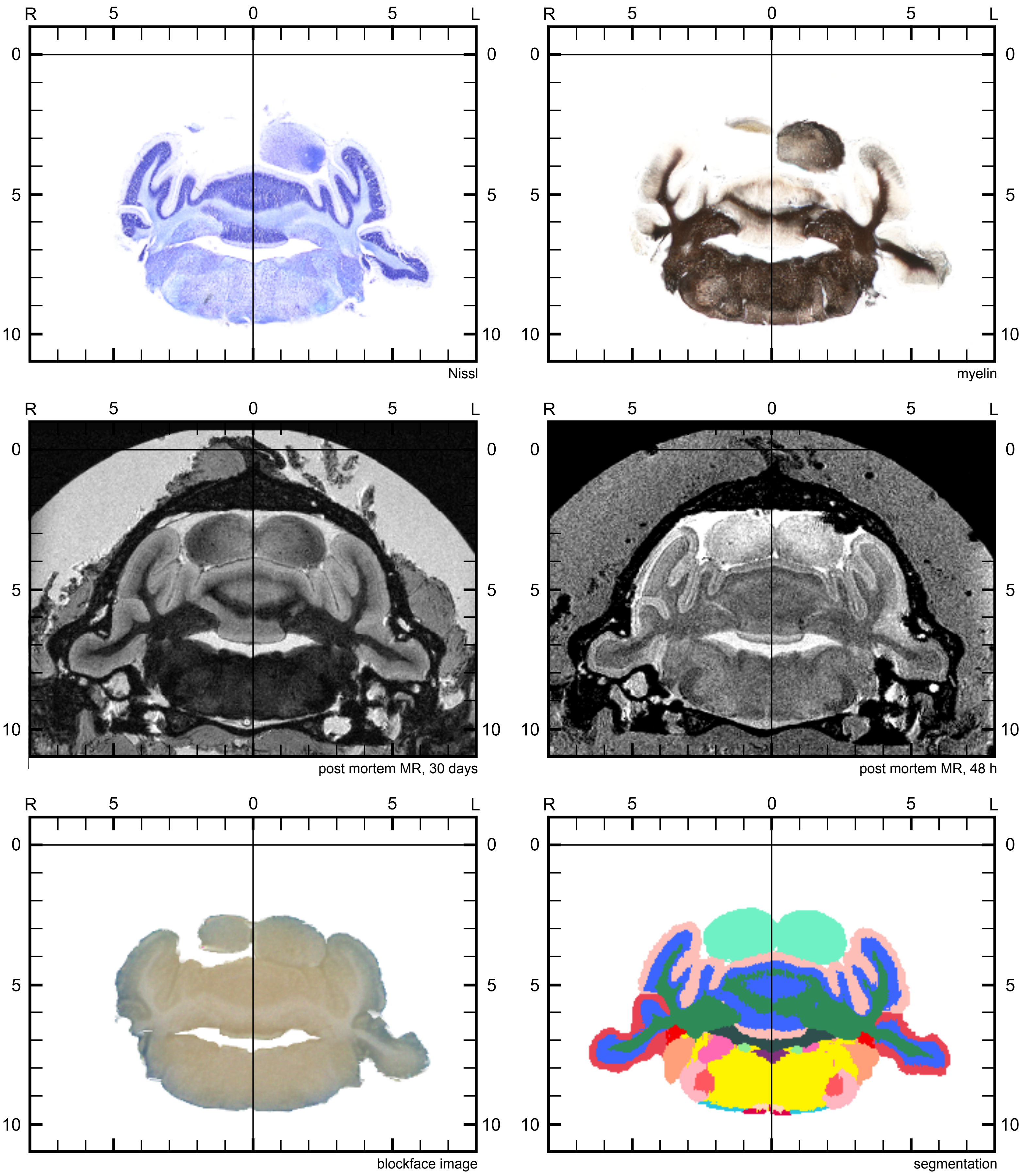

Coronal cross-section 39
1.5 mm caudal from bregma

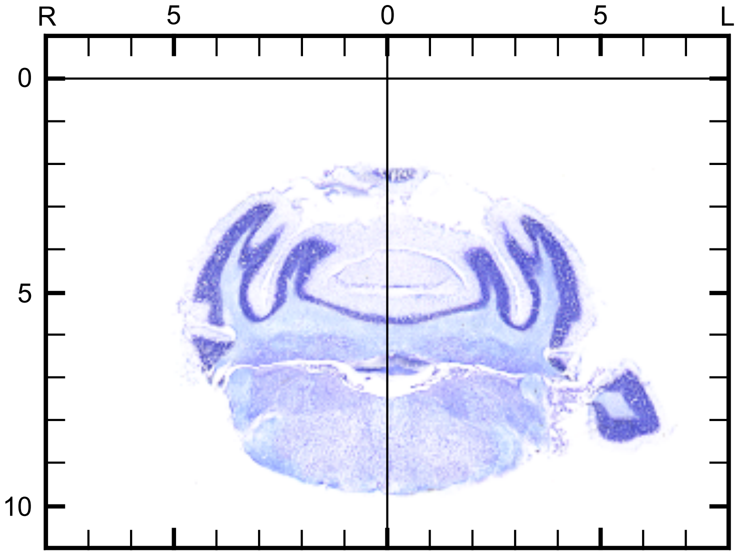

Nissl

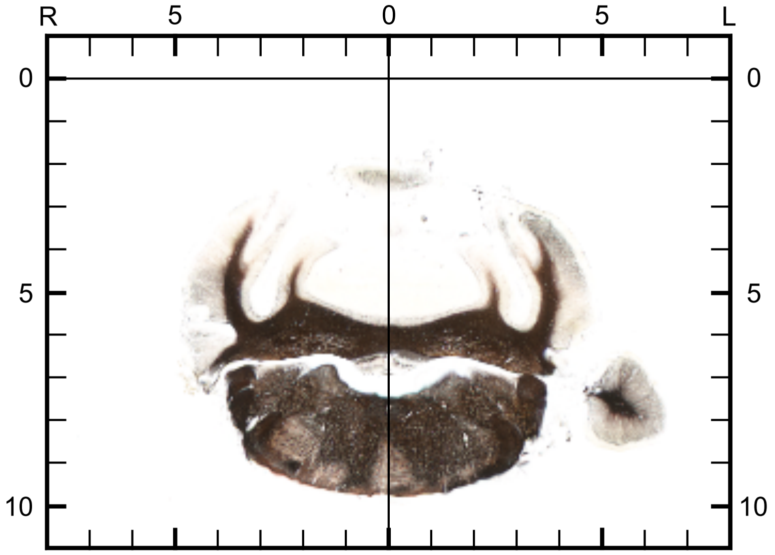

myelin

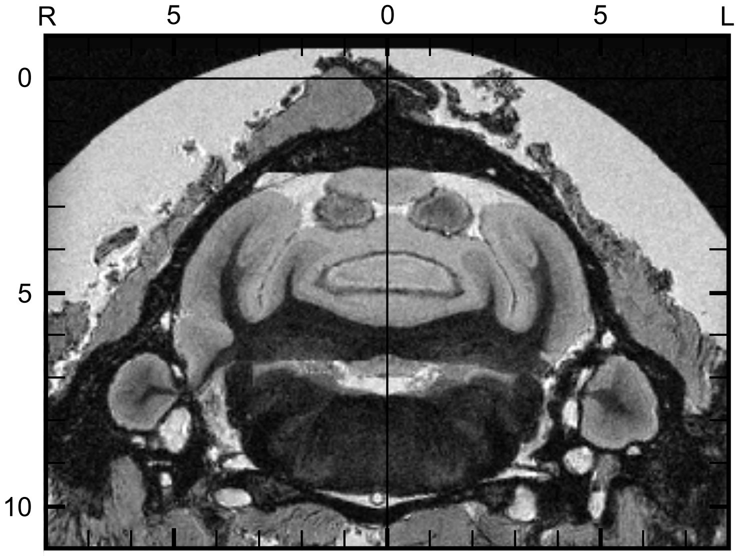

post mortem MR, 30 days

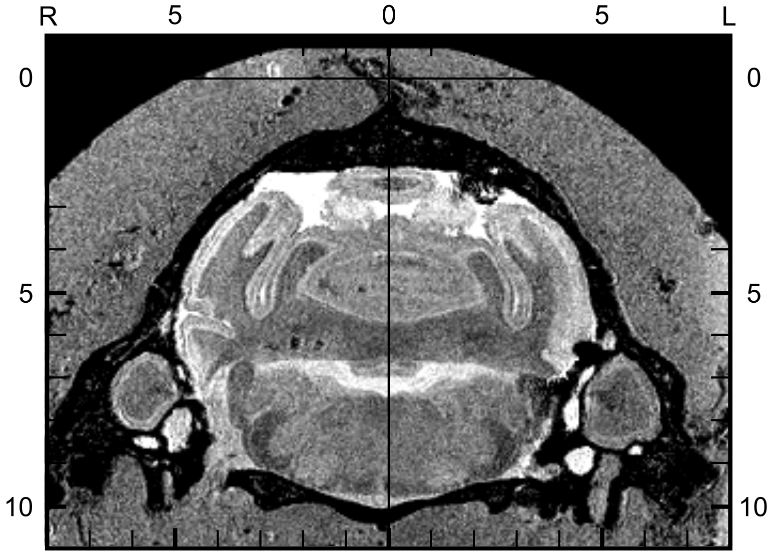

post mortem MR, 48 h

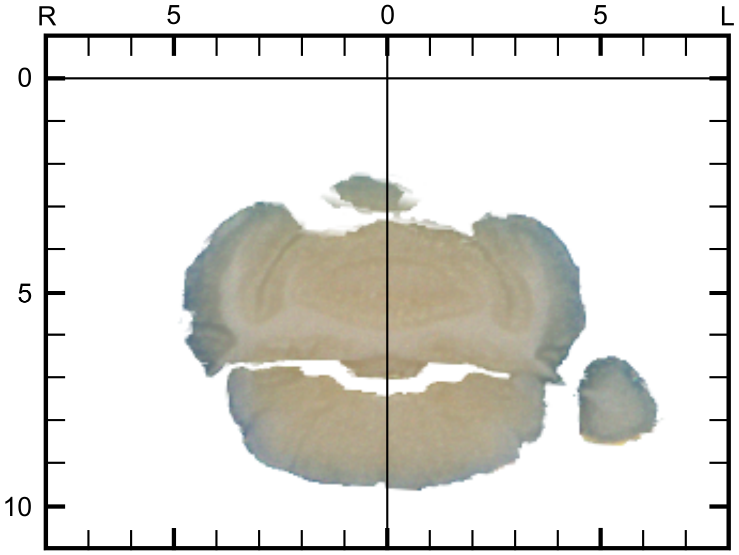

blockface image

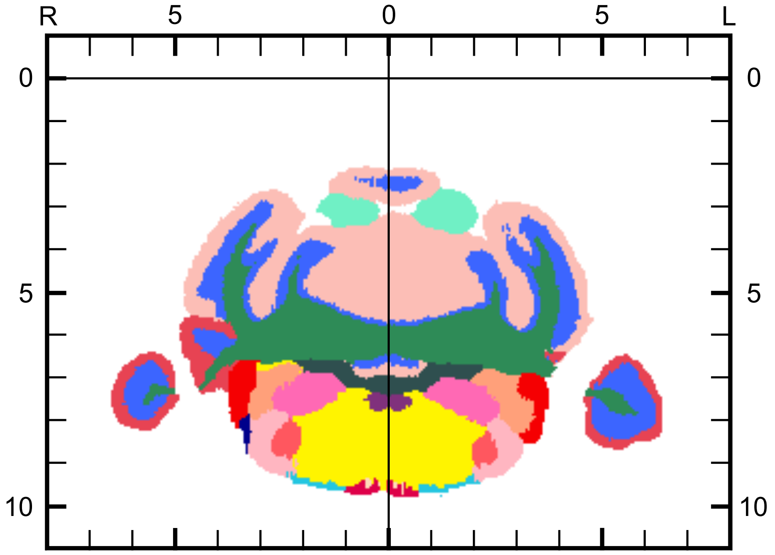

segmentation

Coronal cross-section 40
2.0 mm caudal from bregma

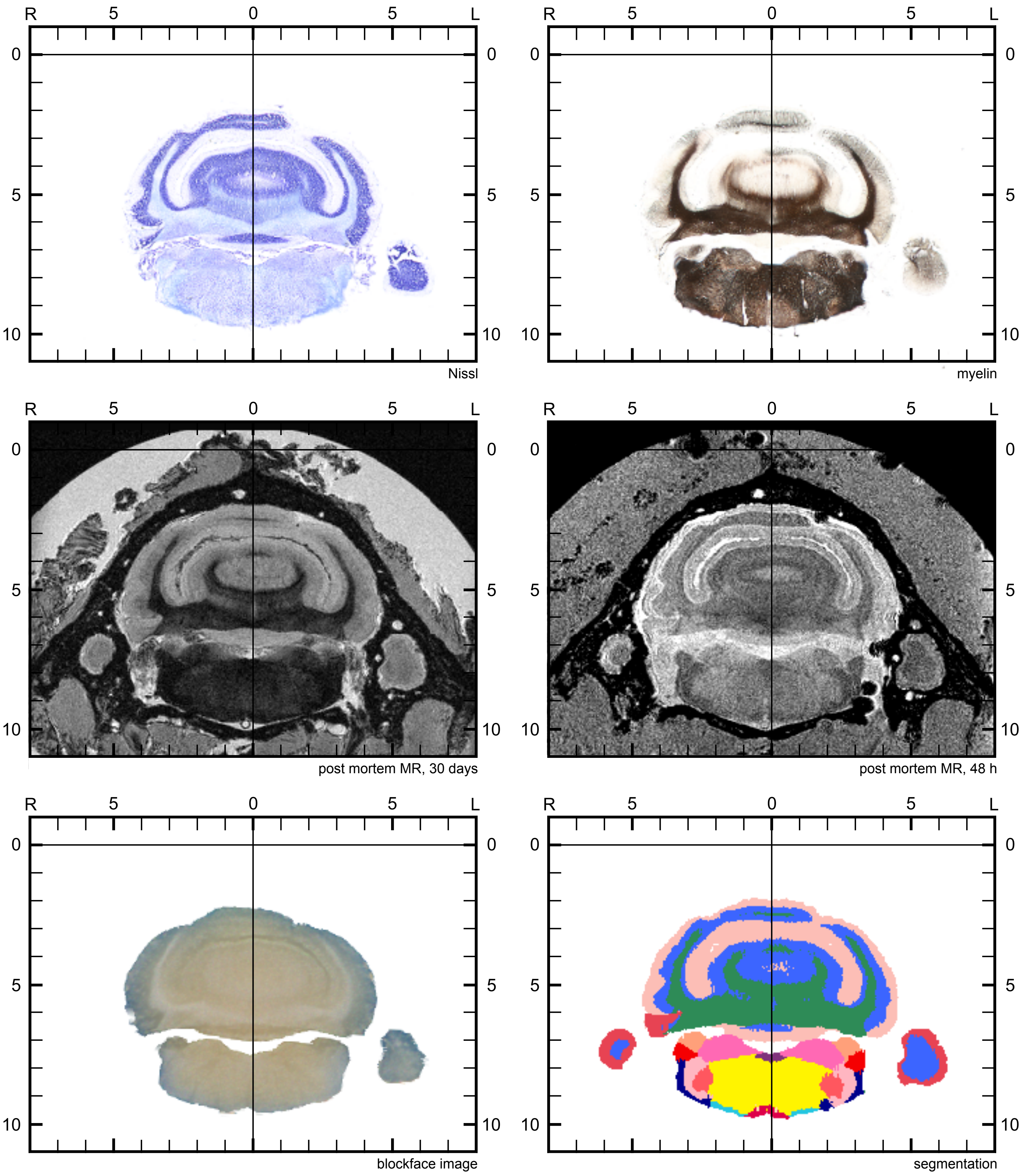

Coronal cross-section 41
2.5 mm caudal from bregma

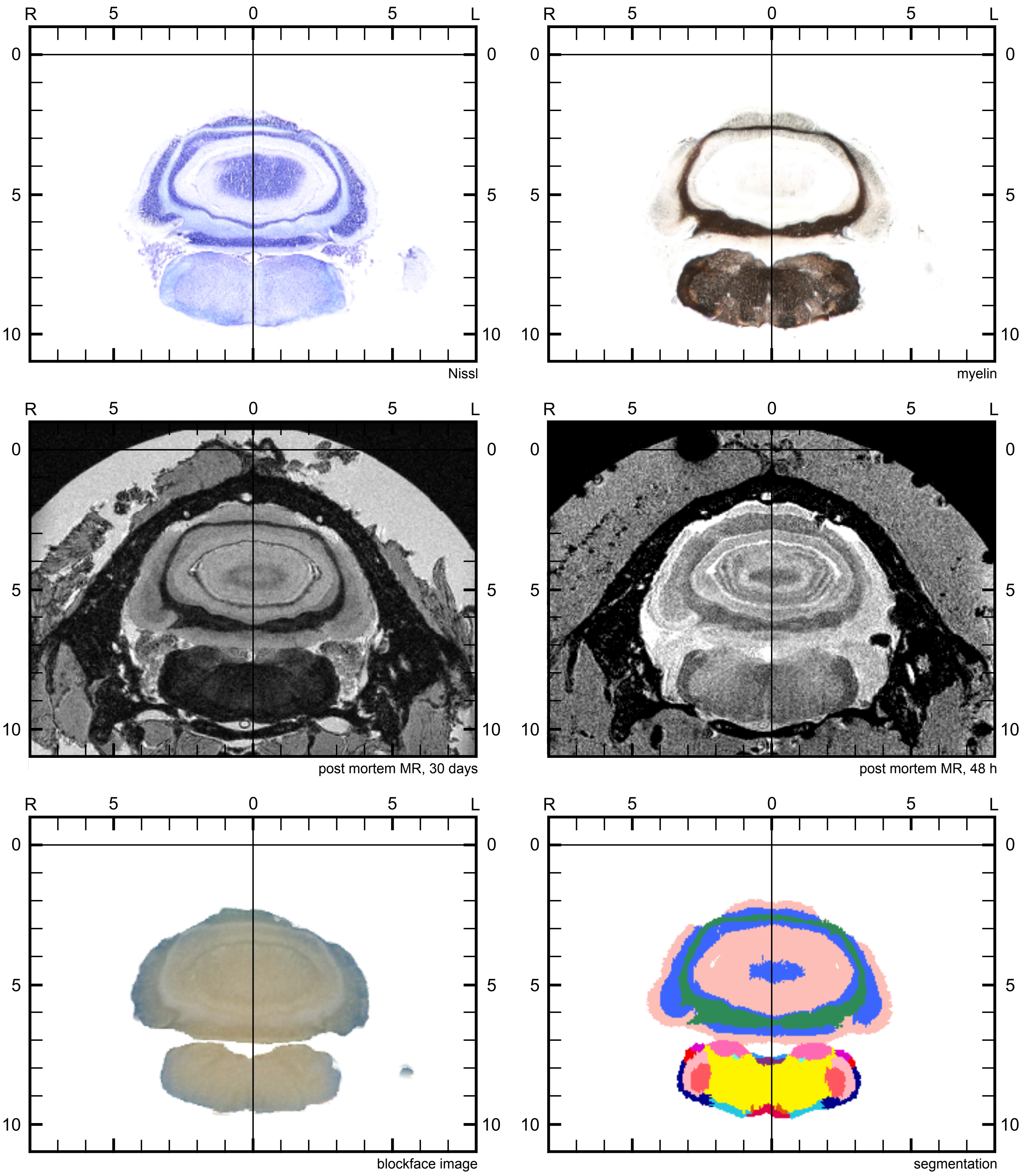

Coronal cross-section 42
3.0 mm caudal from bregma

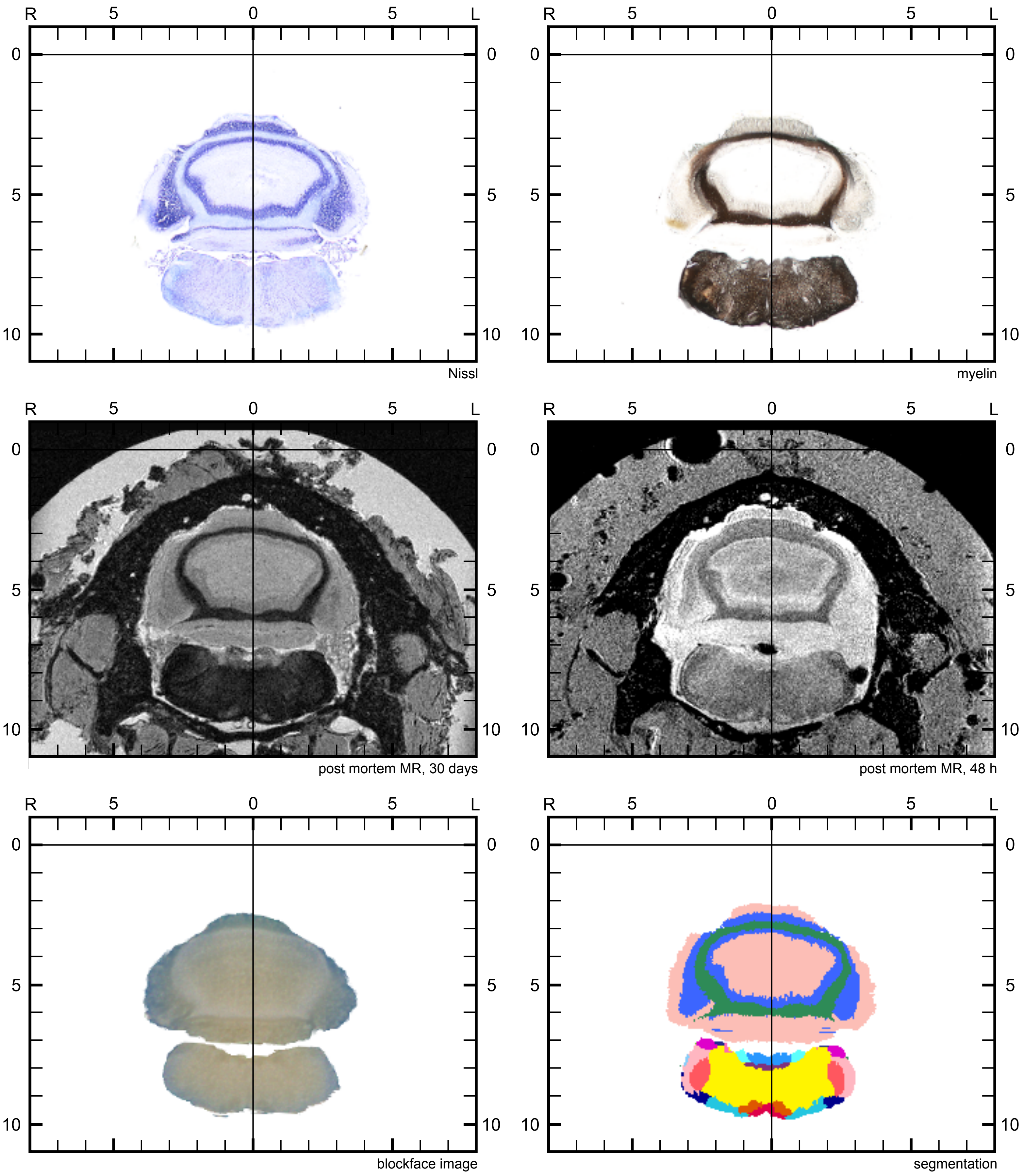

Coronal cross-section 43
3.5 mm caudal from bregma

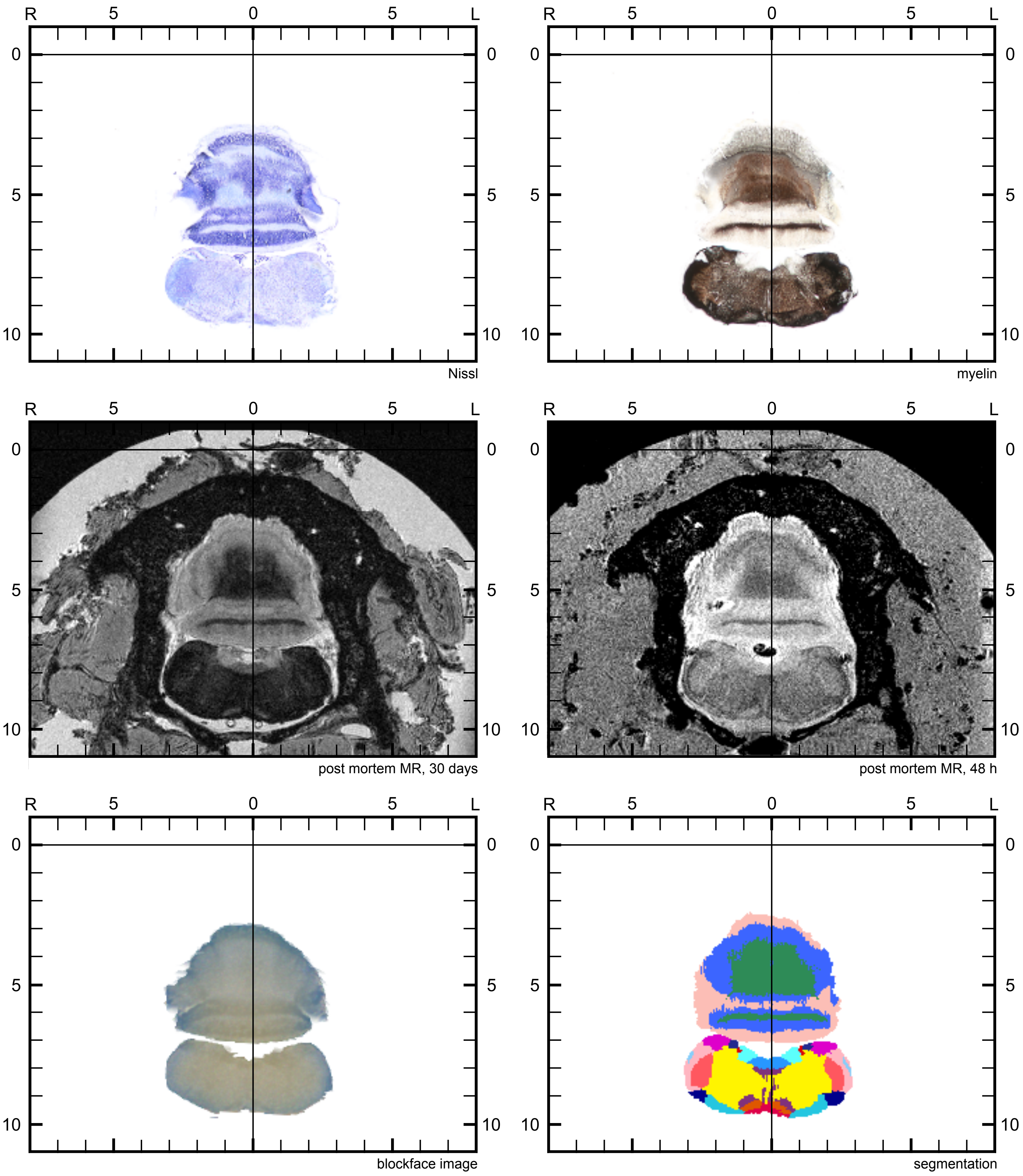

Coronal cross-section 44
4.0 mm caudal from bregma

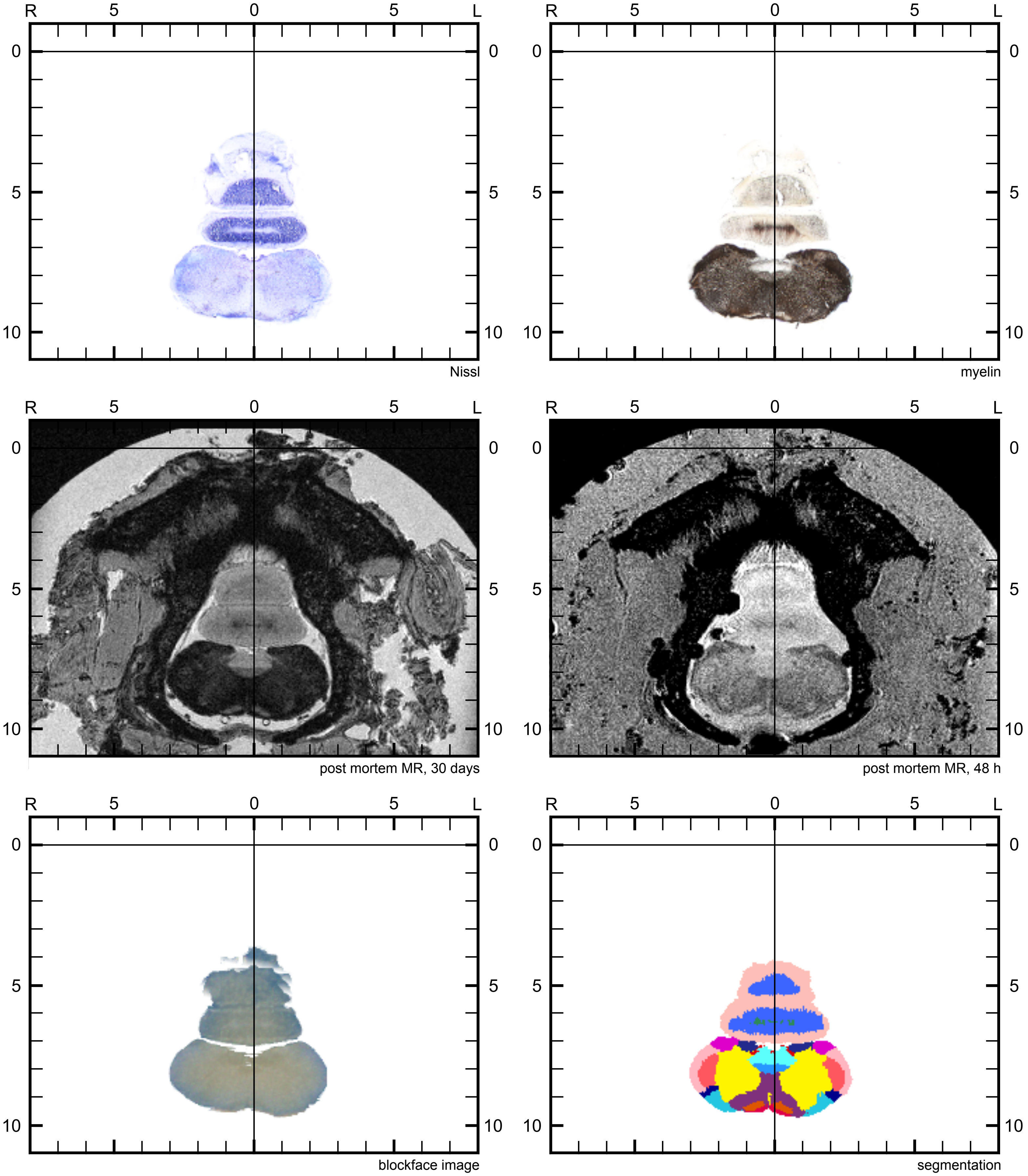

Coronal cross-section 45
4.5 mm caudal from bregma

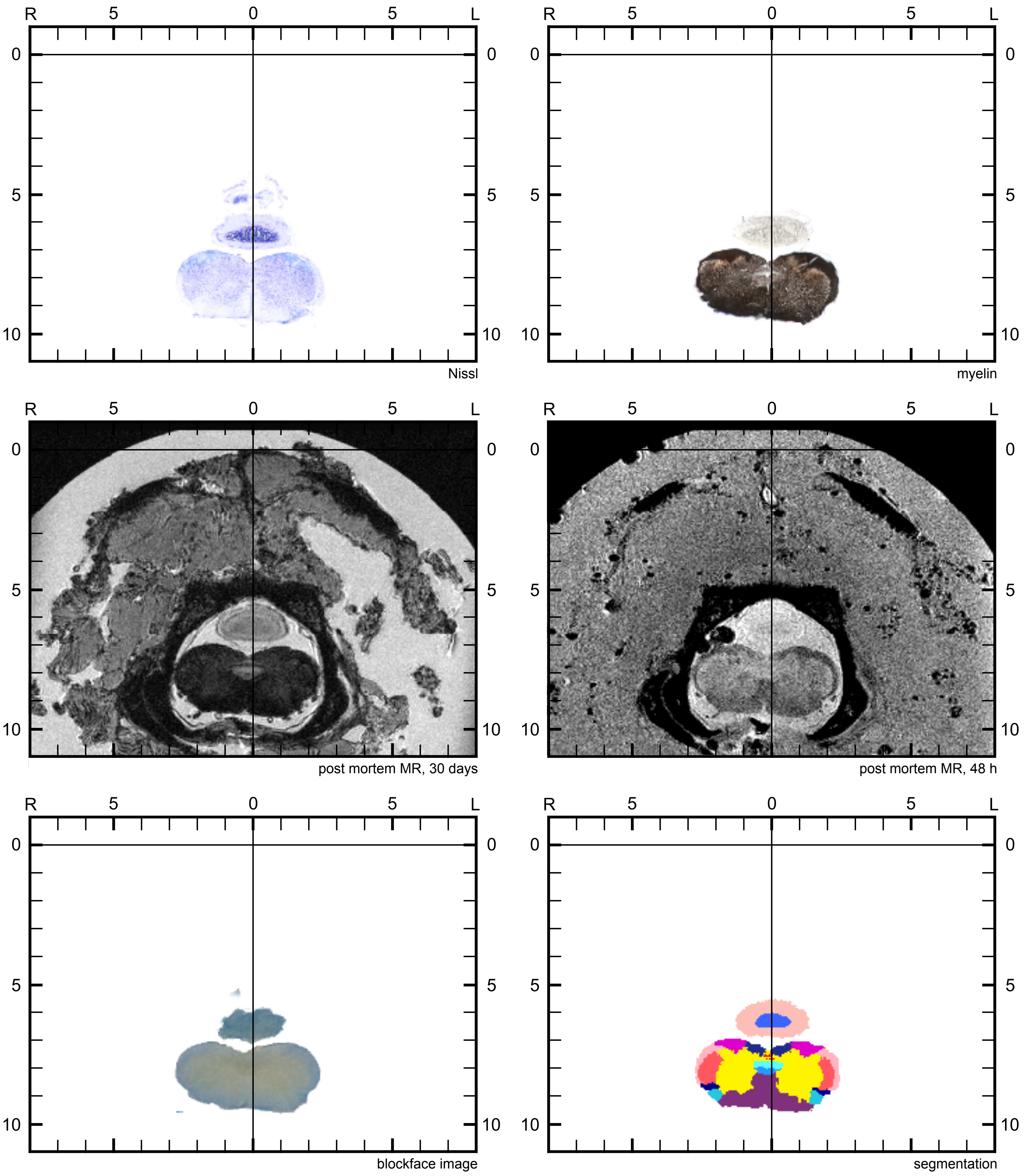

Coronal cross-section 46
5.0 mm caudal from bregma

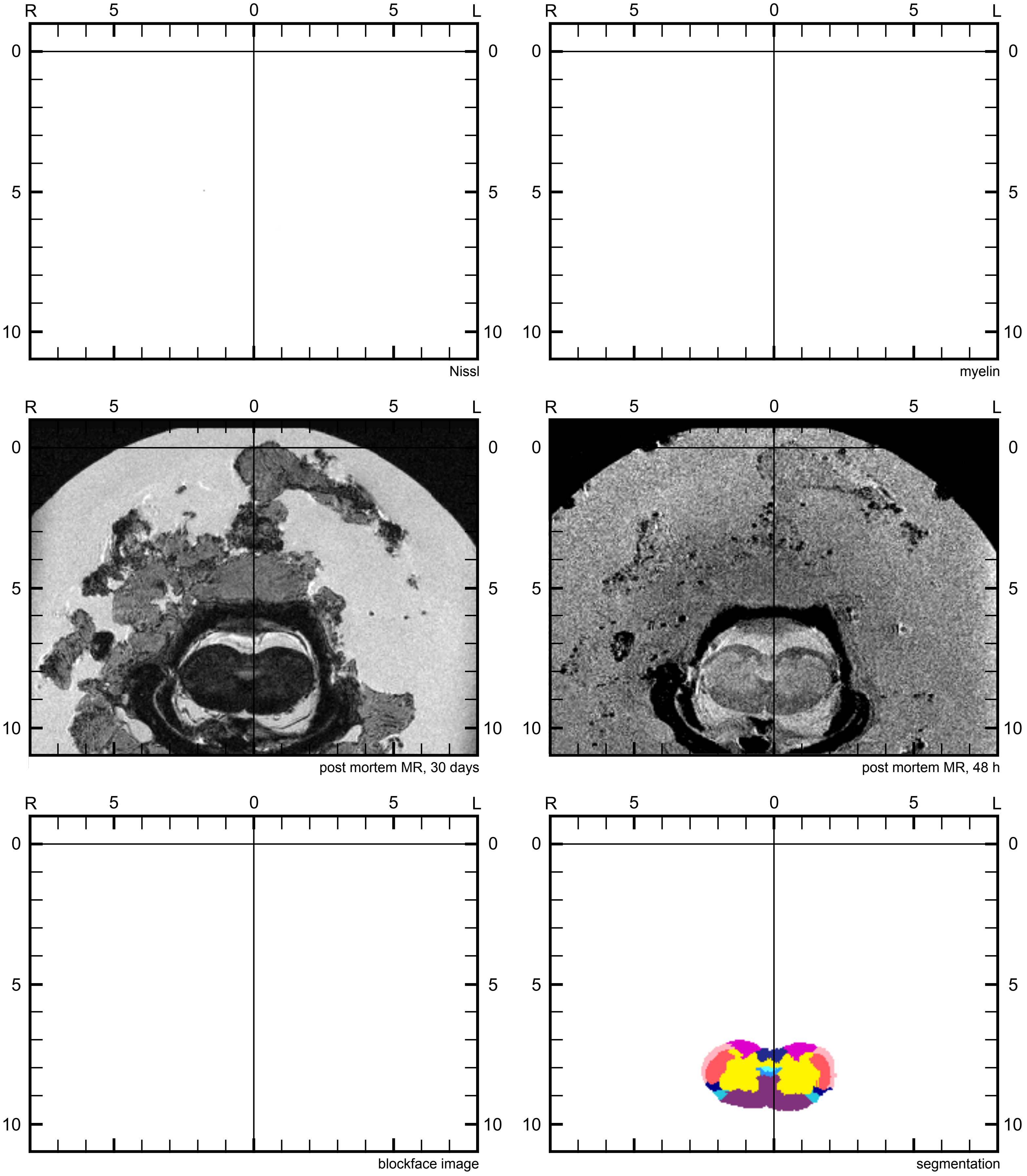

Horizontal cross-section 1
bregma level

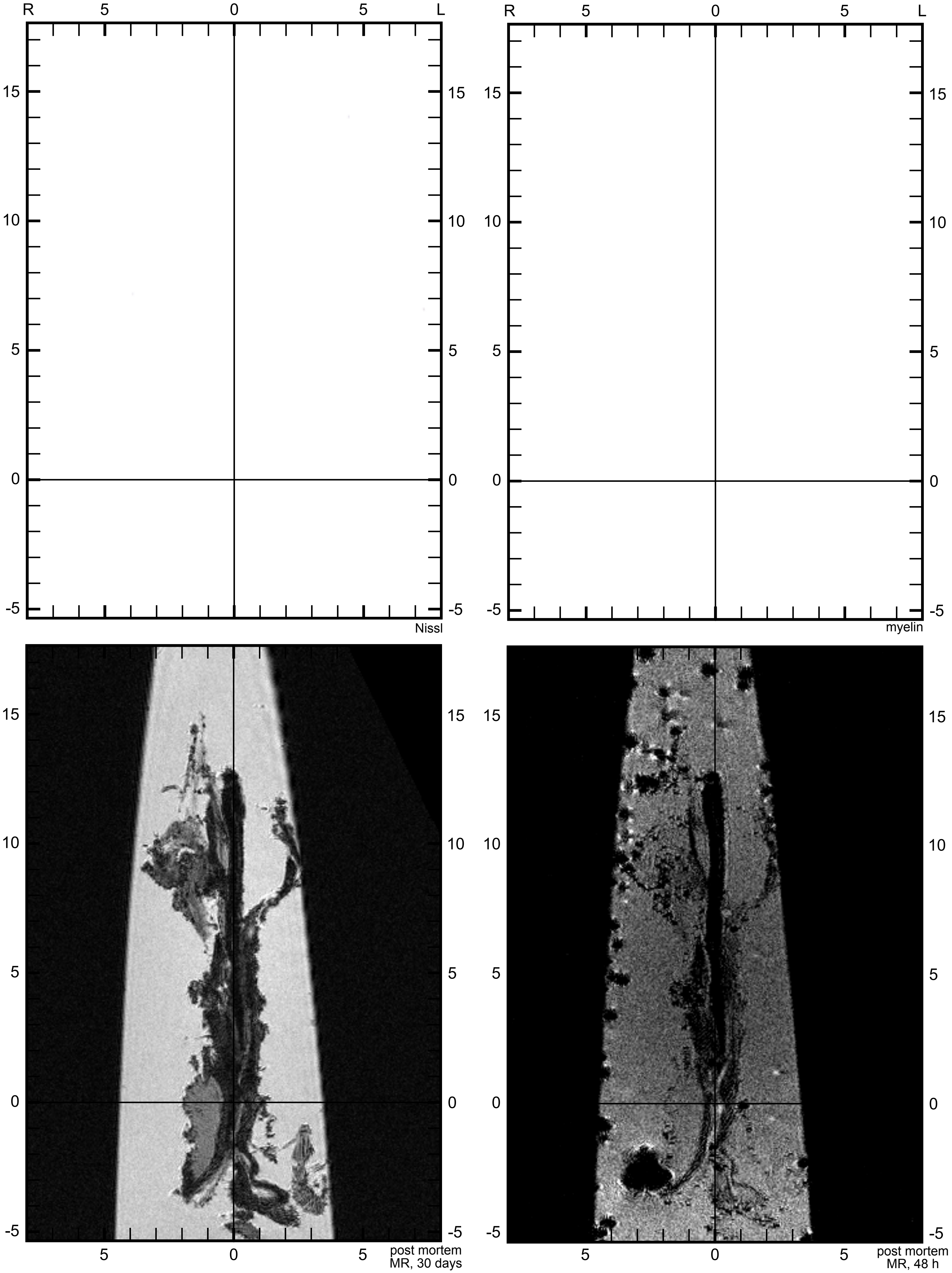

Horizontal cross-section 1 (continued)
bregma level

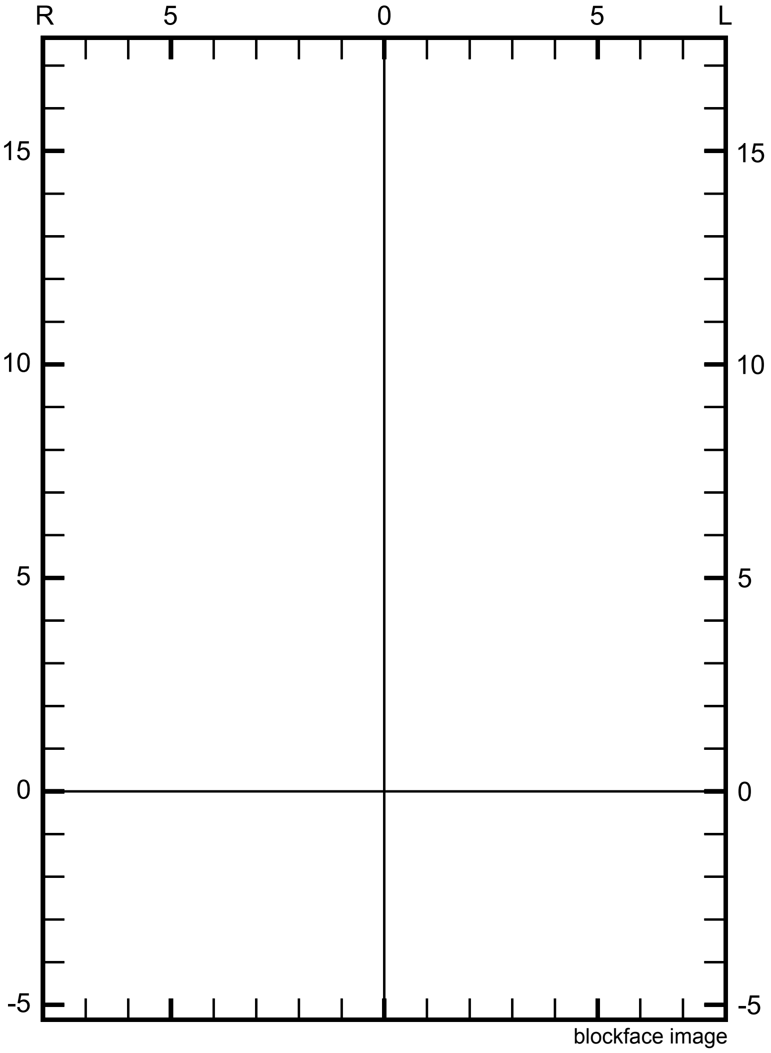

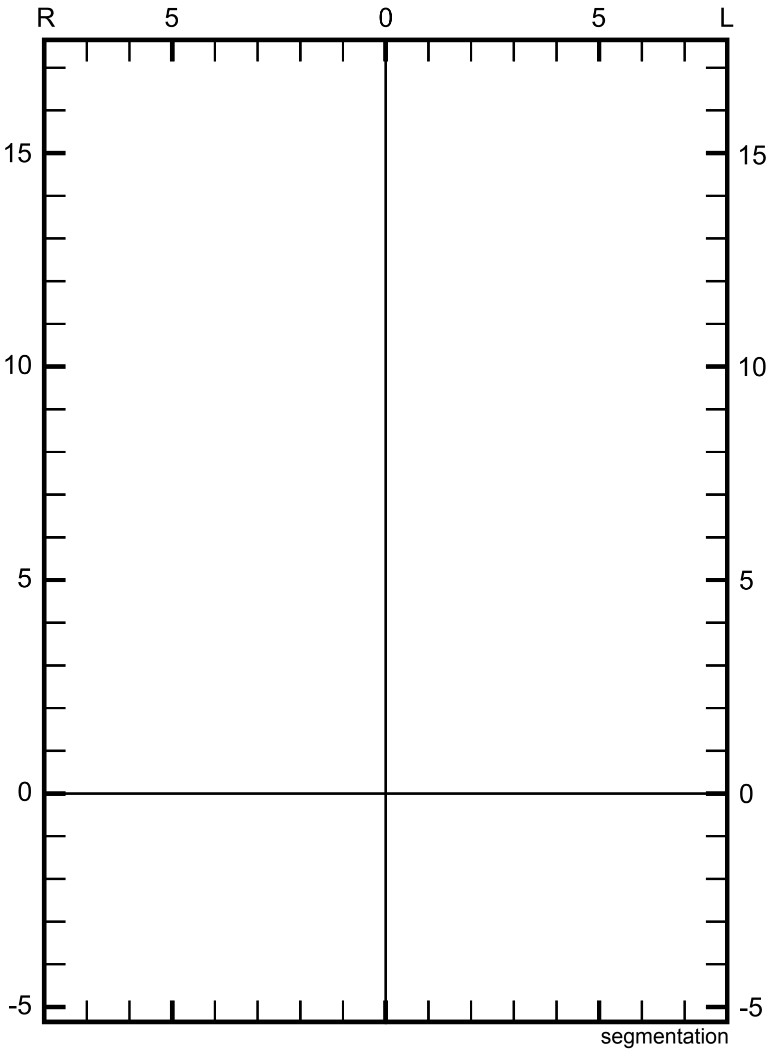

Horizontal cross-section 2
0.5 mm ventral from bregma

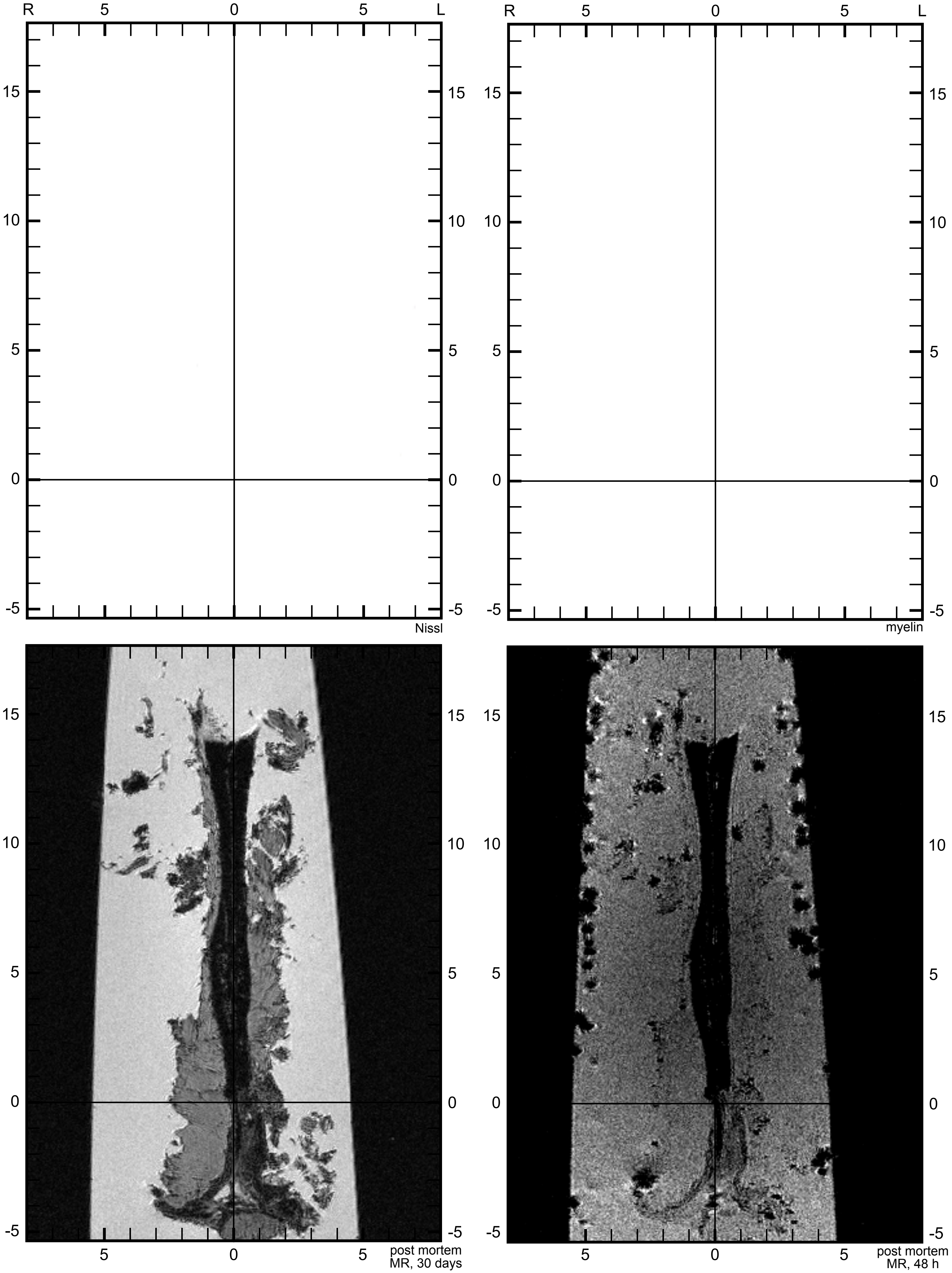

Horizontal cross-section 2 (continued)
0.5 mm ventral from bregma

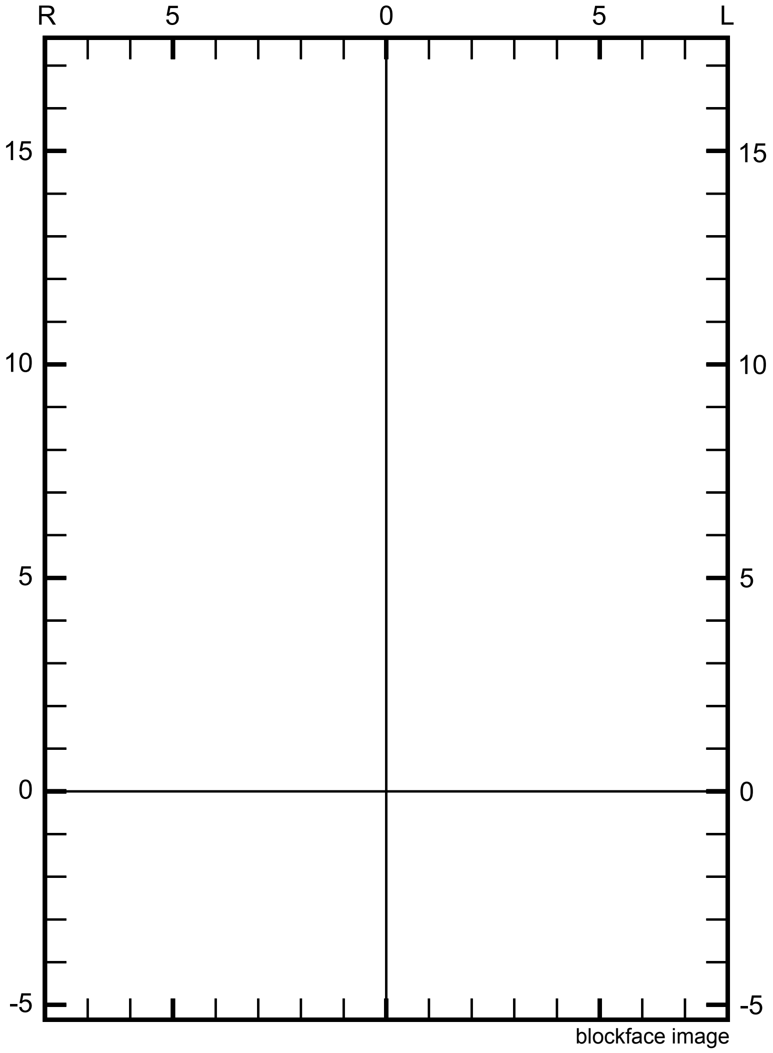

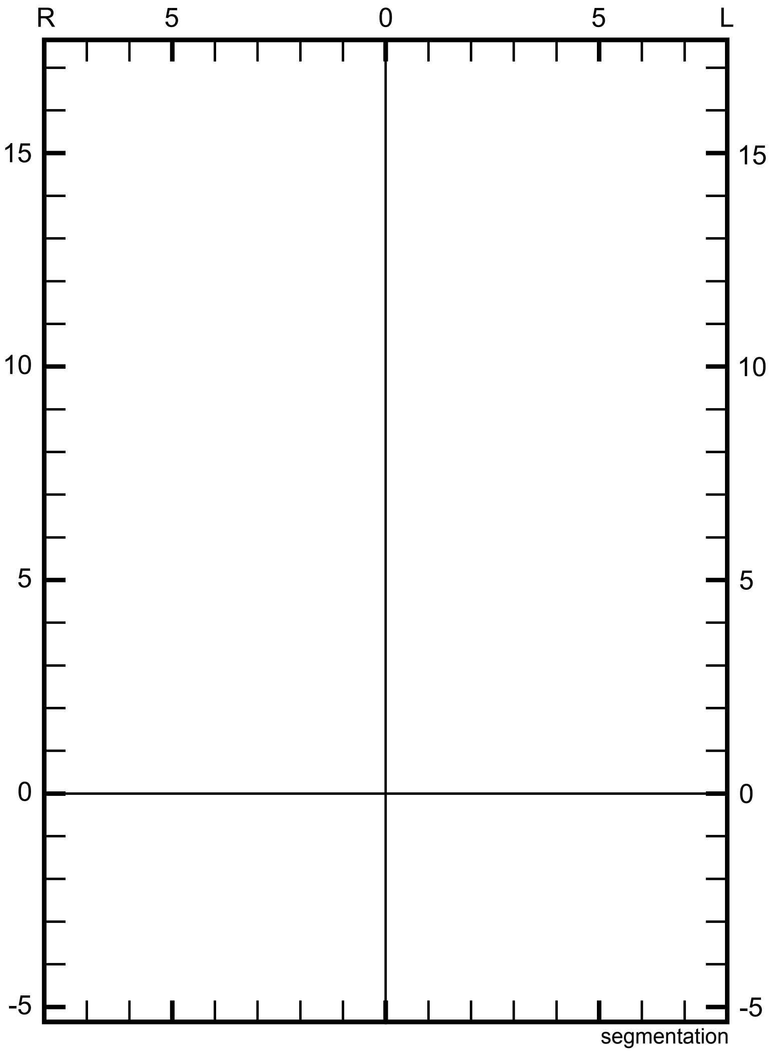

Horizontal cross-section 3
1.0 mm ventral from bregma

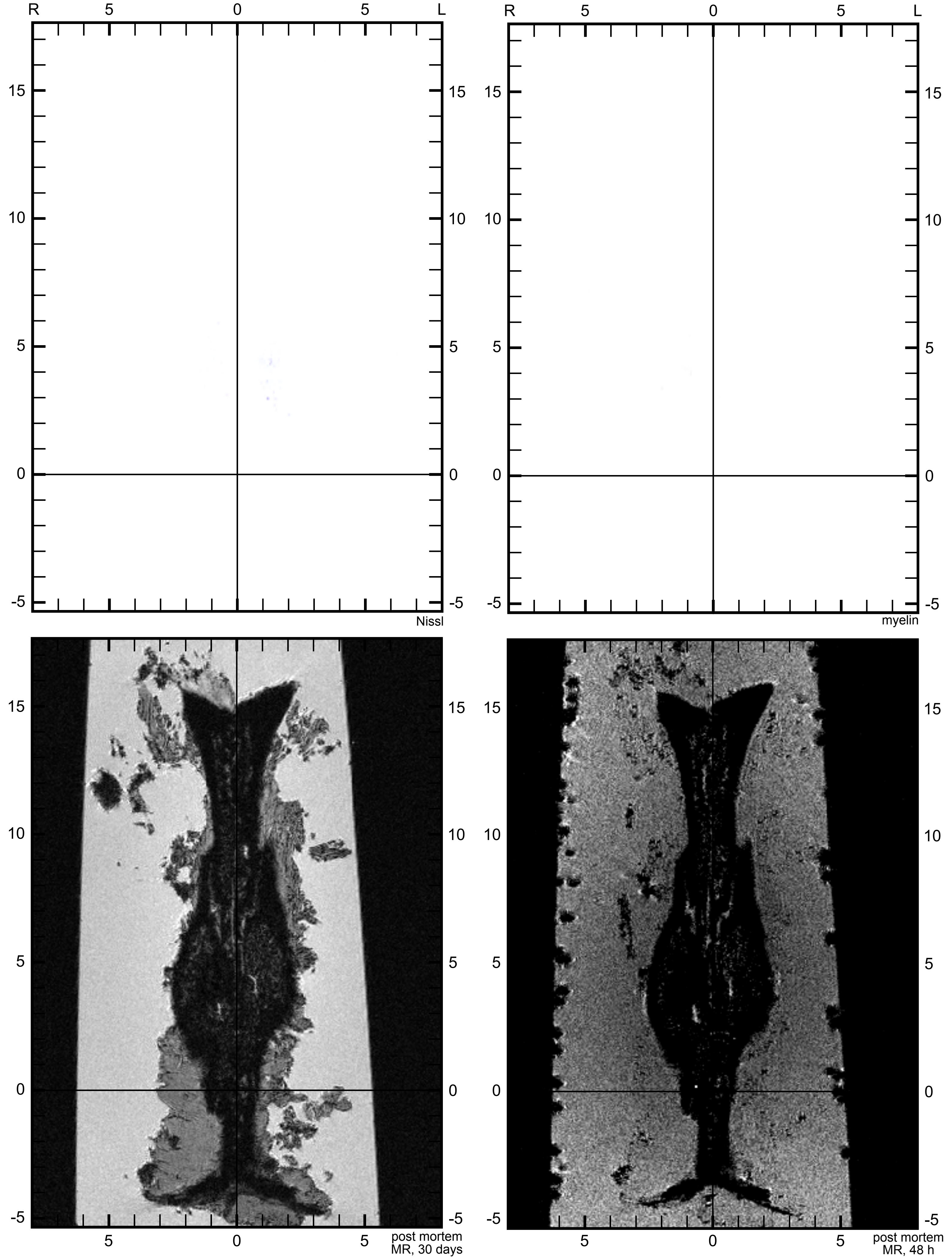

Horizontal cross-section 3 (continued)
1.0 mm ventral from bregma

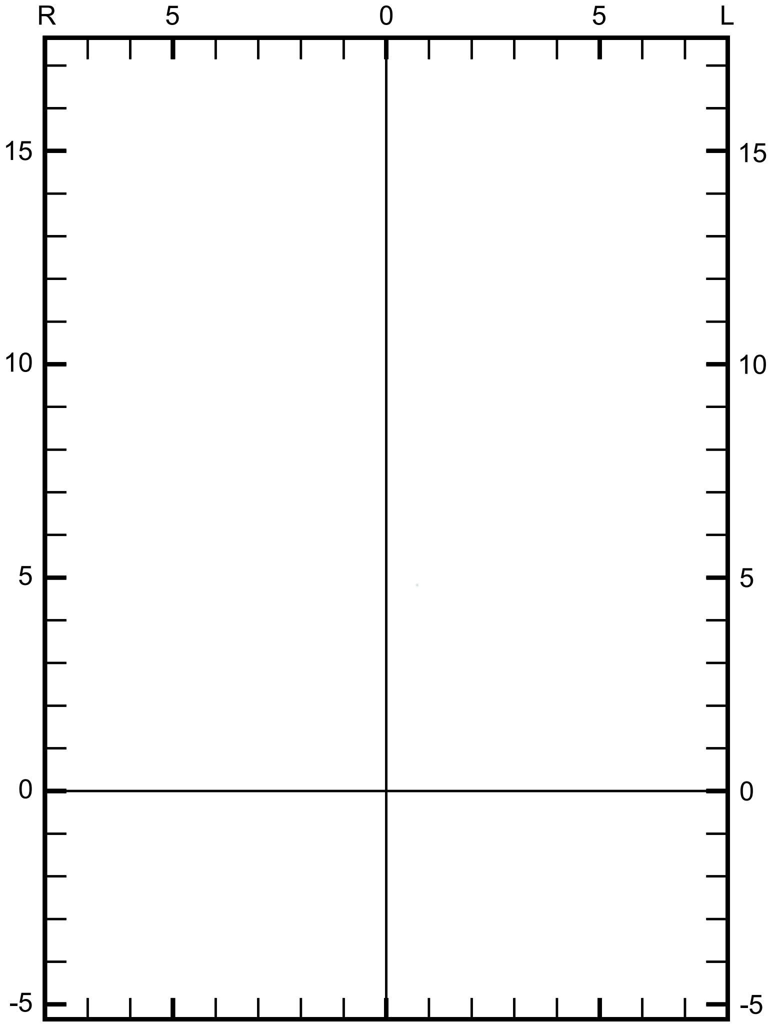

blockface image

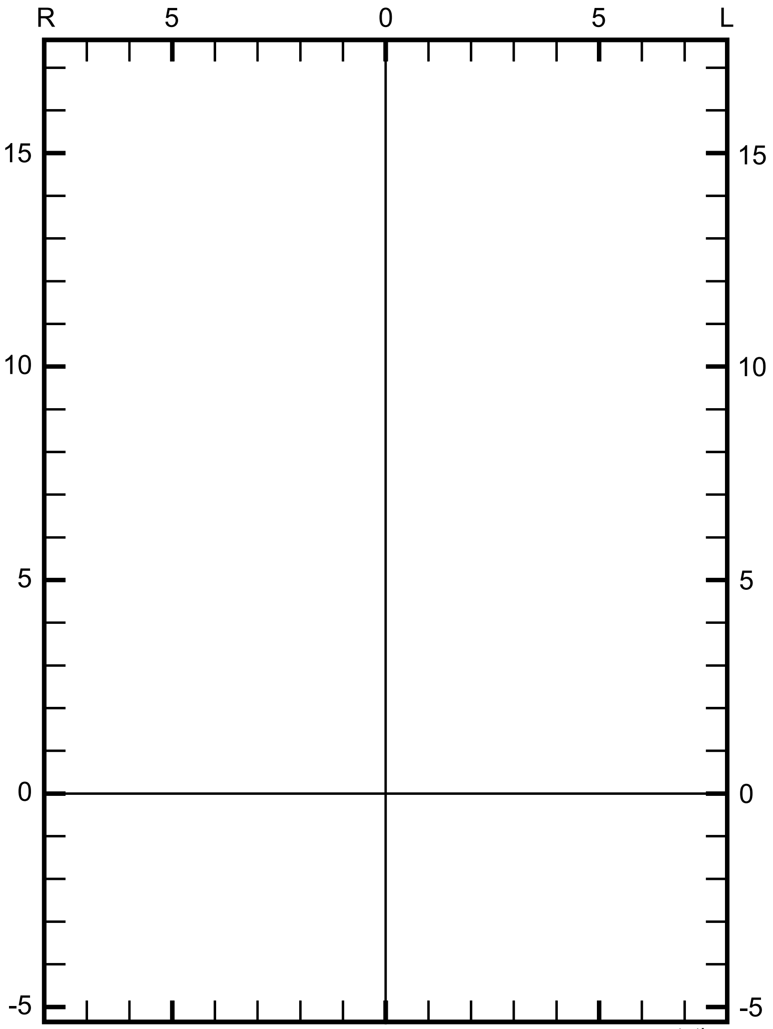

segmentation

Horizontal cross-section 4
1.5 mm ventral from bregma

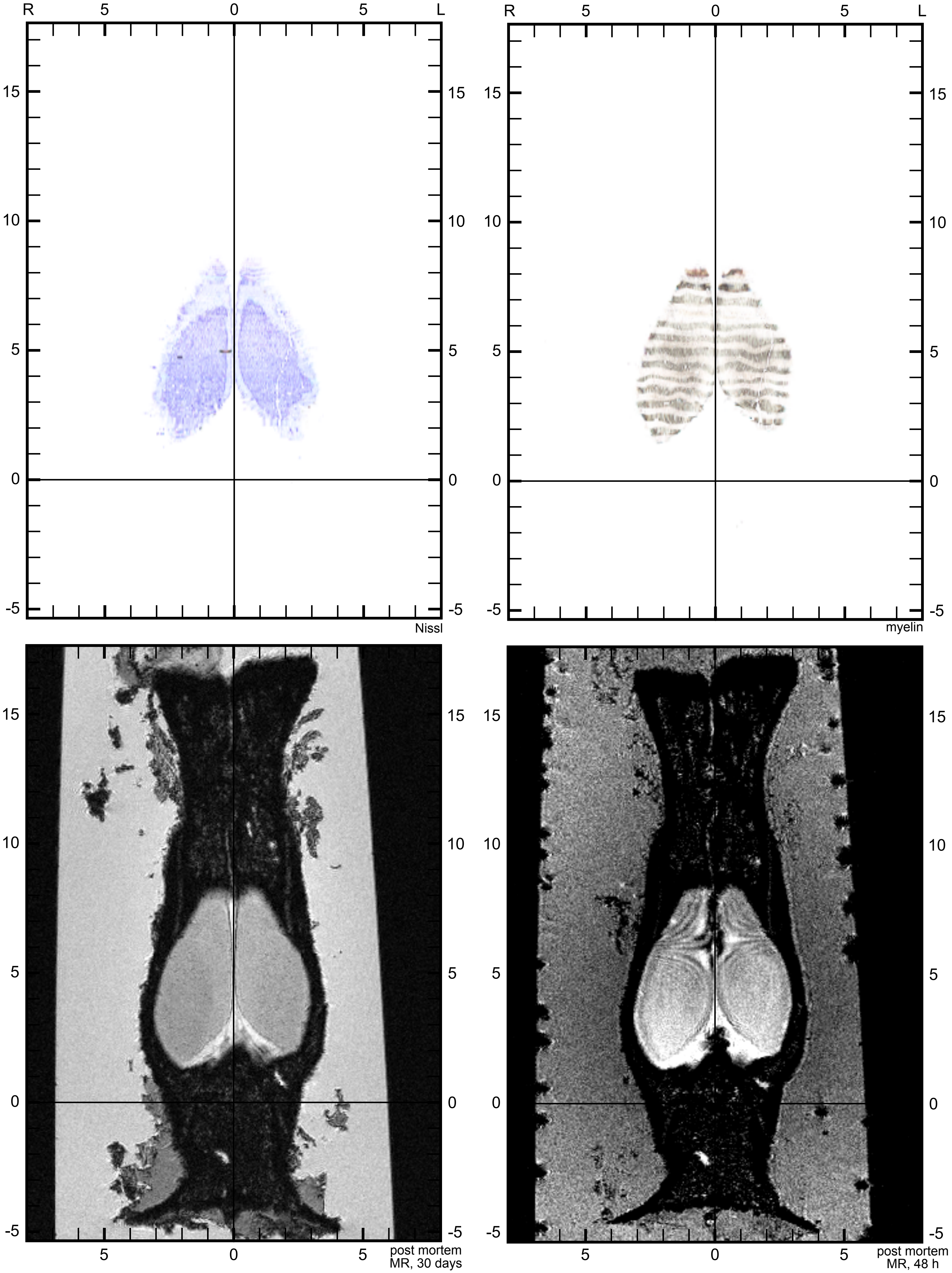

Horizontal cross-section 4 (continued)
1.5 mm ventral from bregma

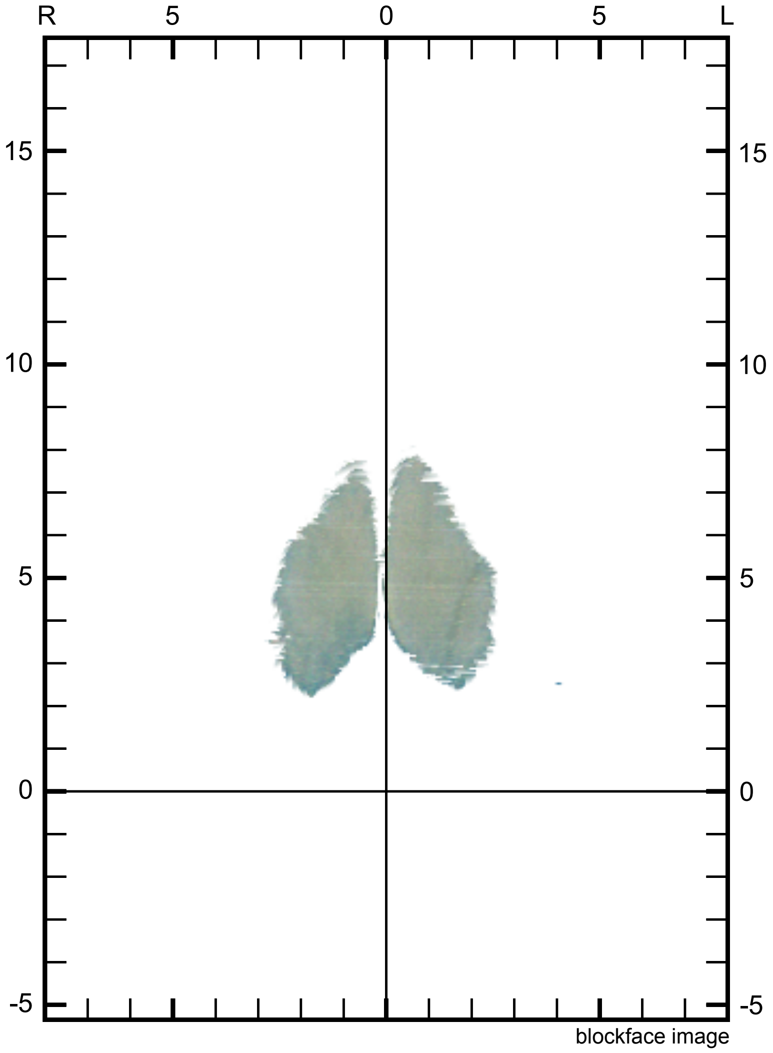

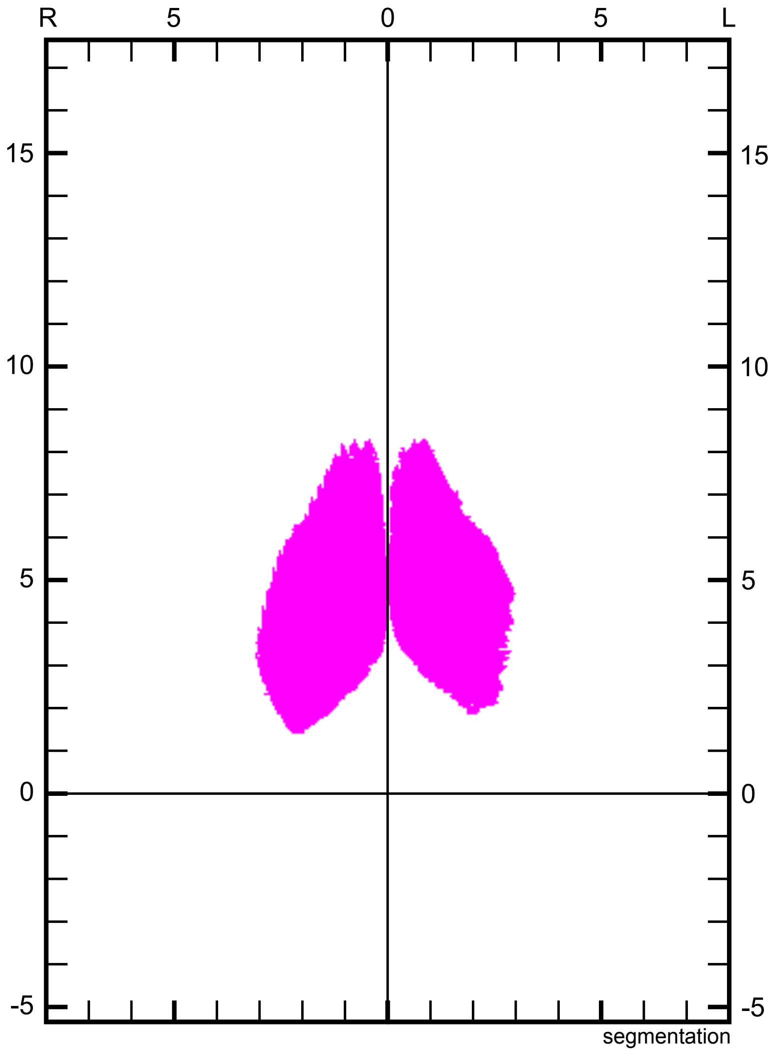

Horizontal cross-section 5
2.0 mm ventral from bregma

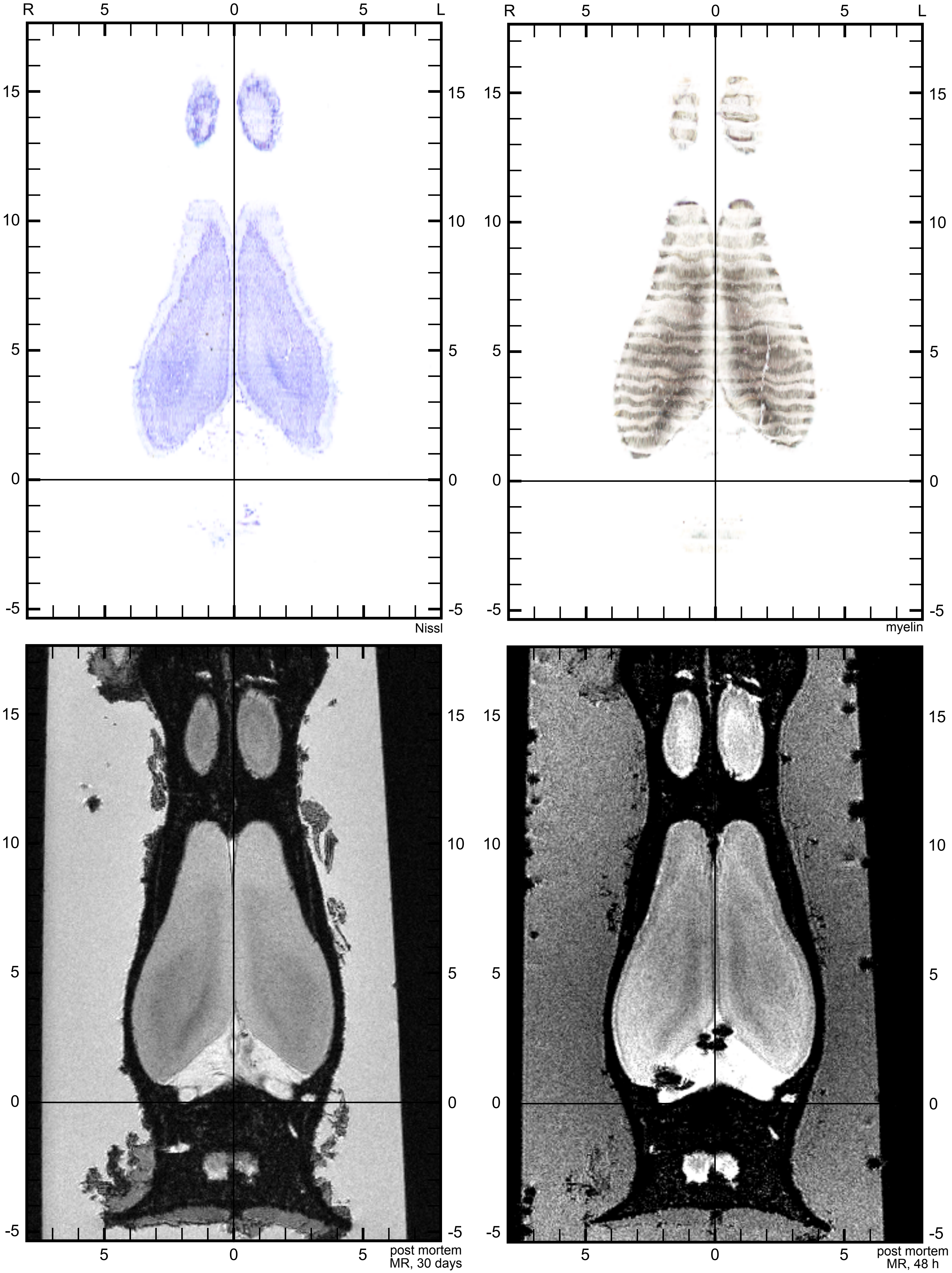

Horizontal cross-section 5 (continued)
2.0 mm ventral from bregma

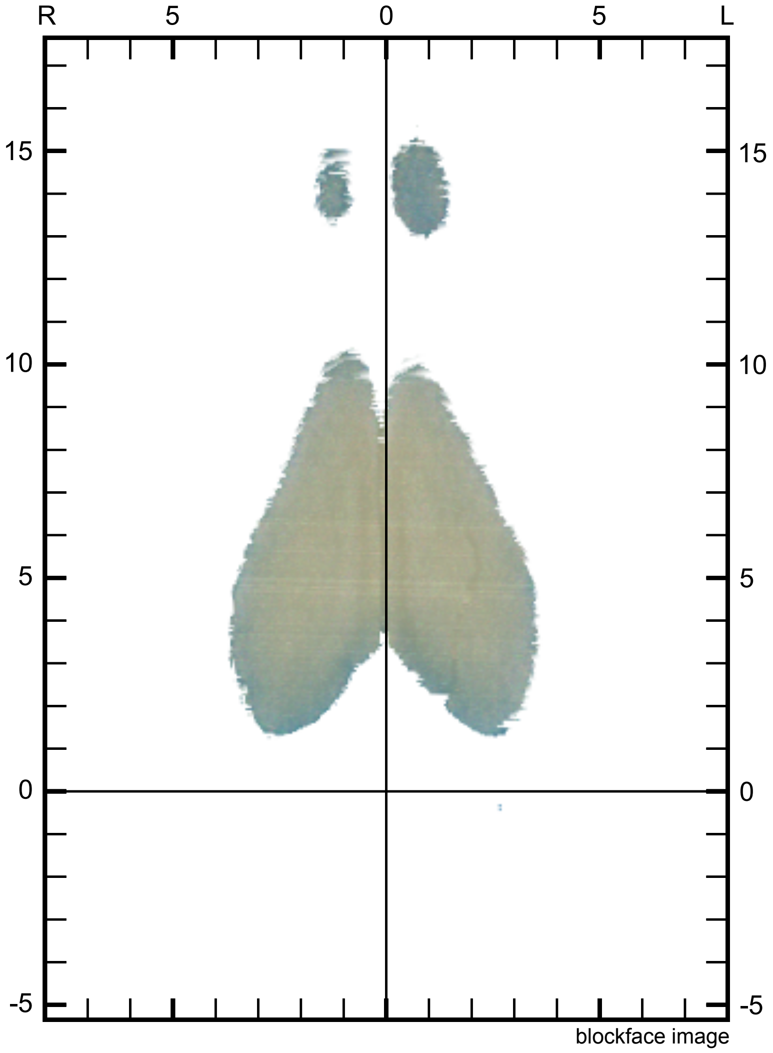

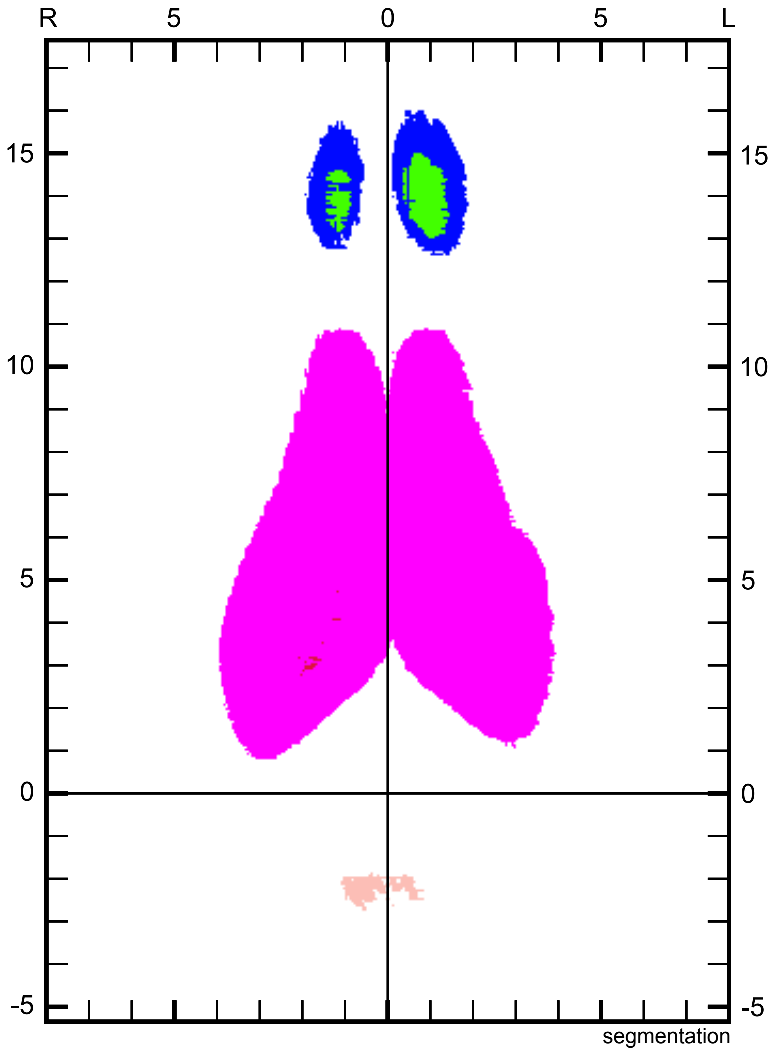

Horizontal cross-section 6
2.5 mm ventral from bregma

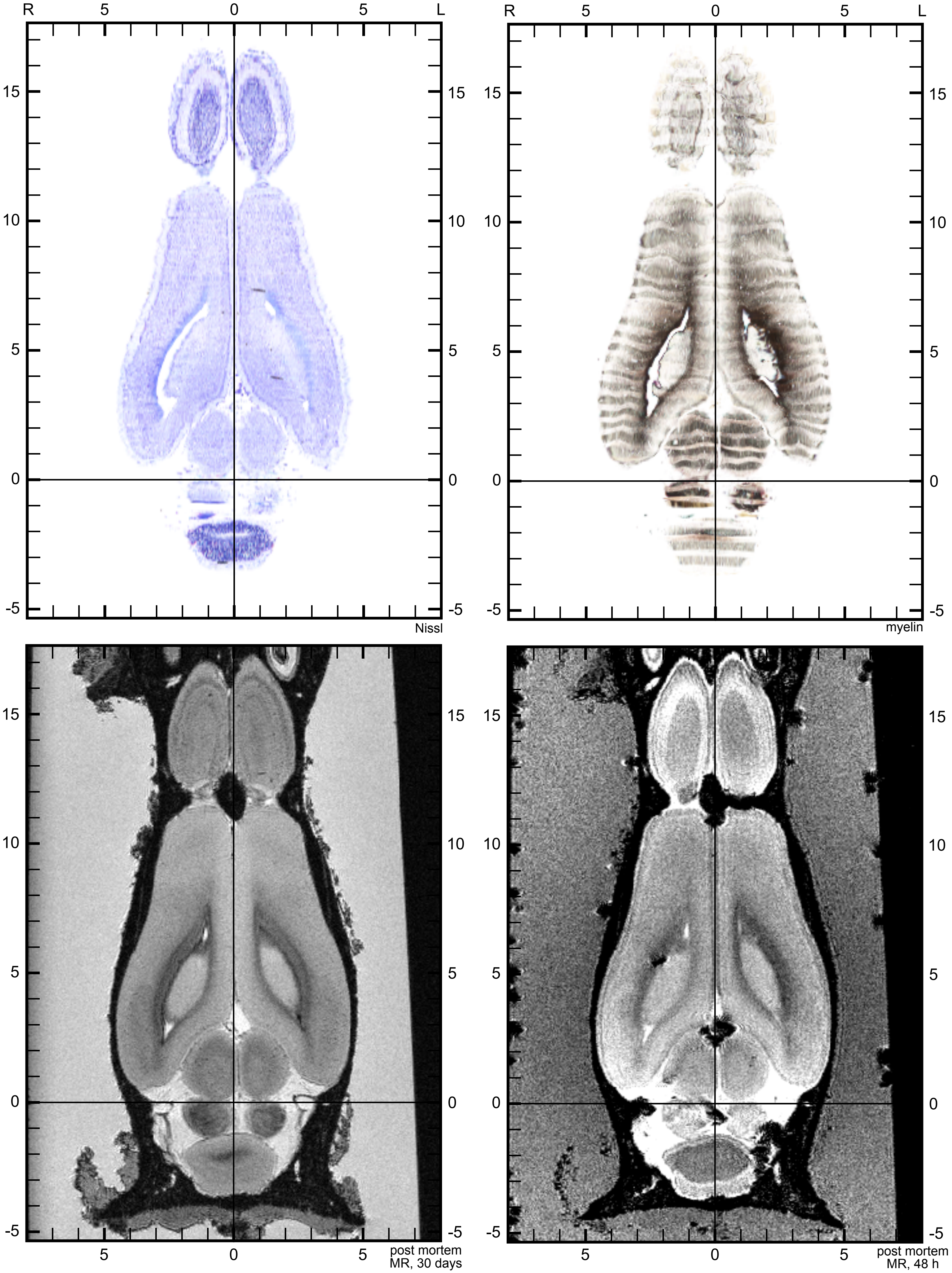

Horizontal cross-section 6 (continued)
2.5 mm ventral from bregma

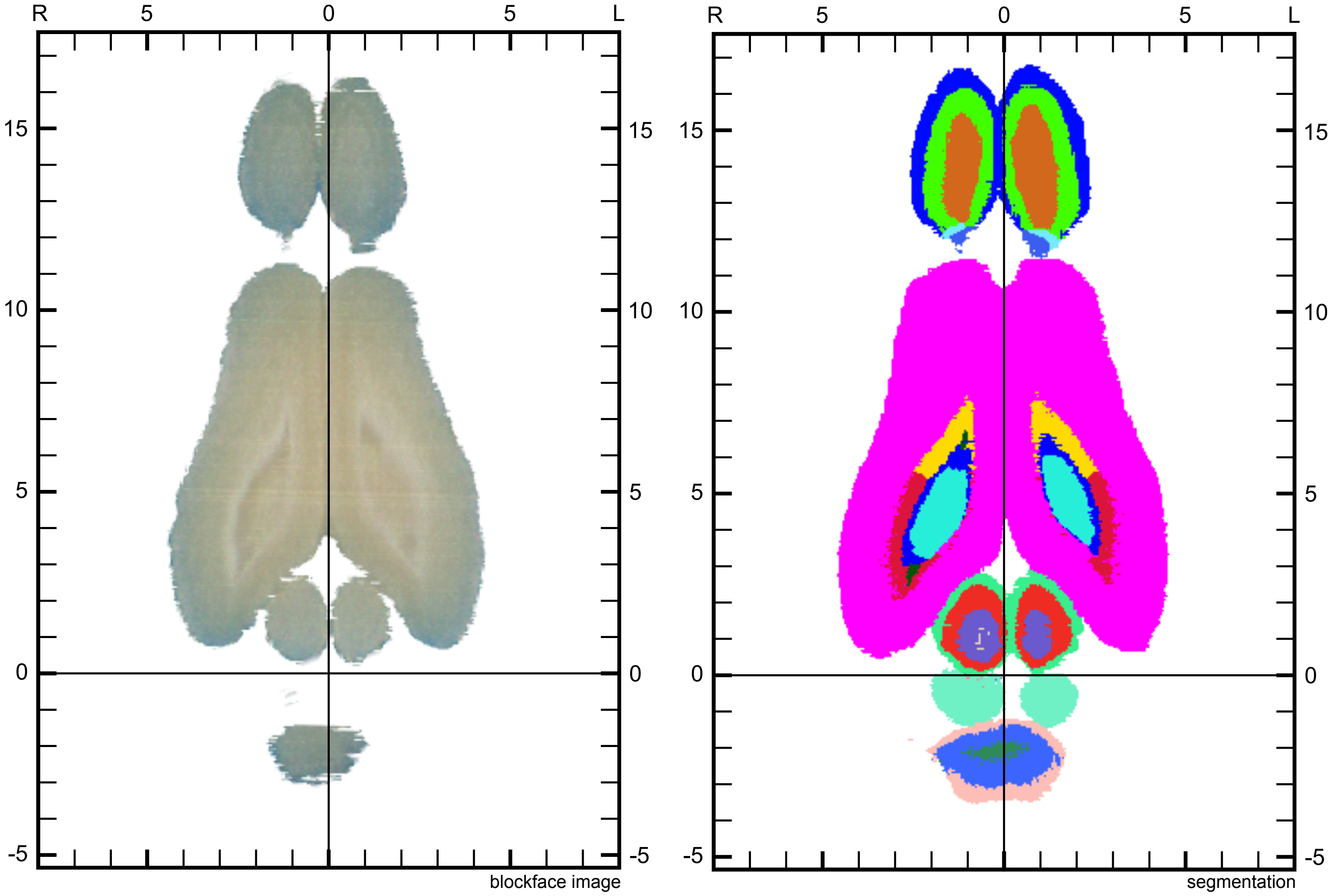

Horizontal cross-section 7
3.0 mm ventral from bregma

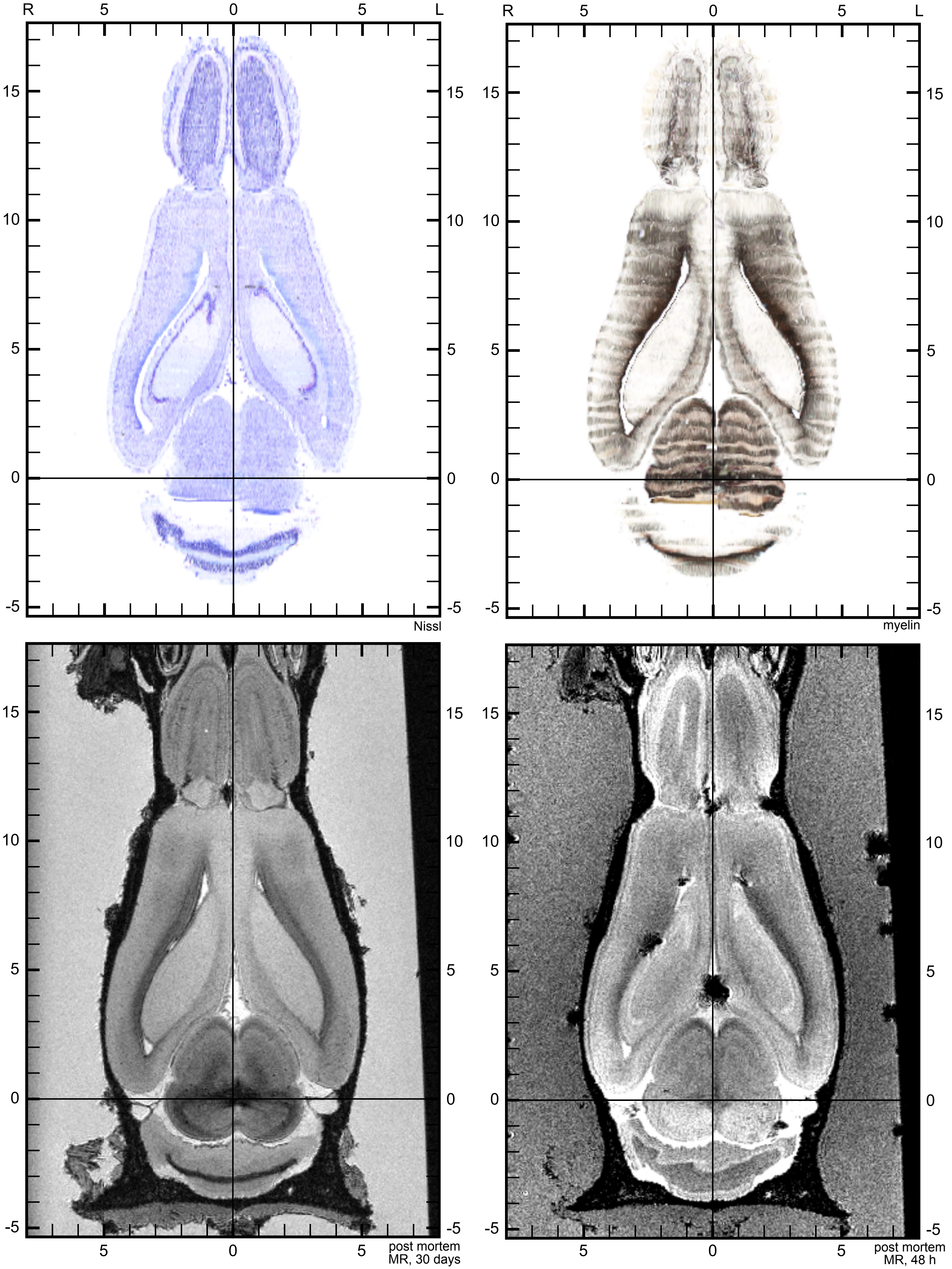

Horizontal cross-section 7 (continued)
3.0 mm ventral from bregma

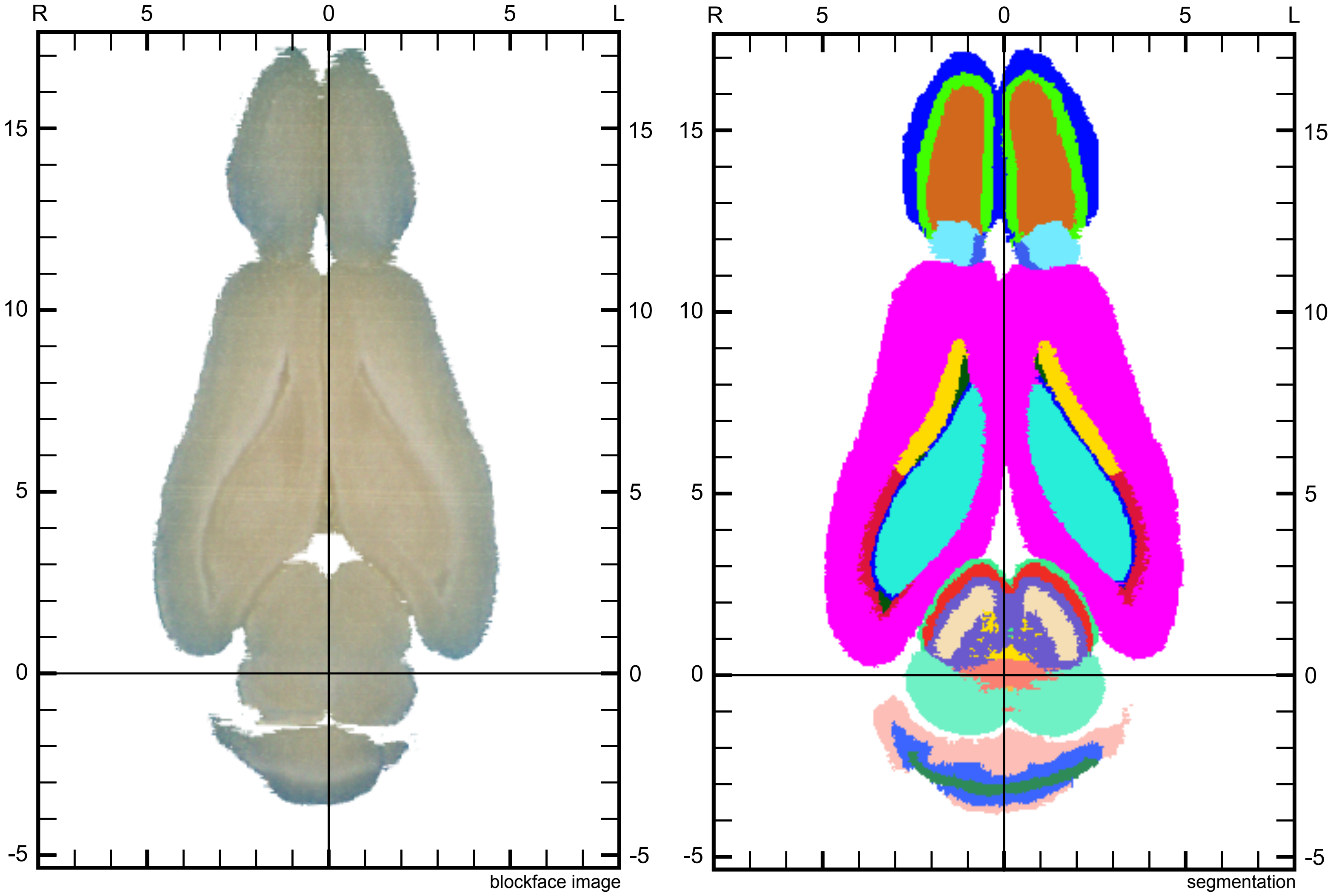

Horizontal cross-section 8
3.5 mm ventral from bregma

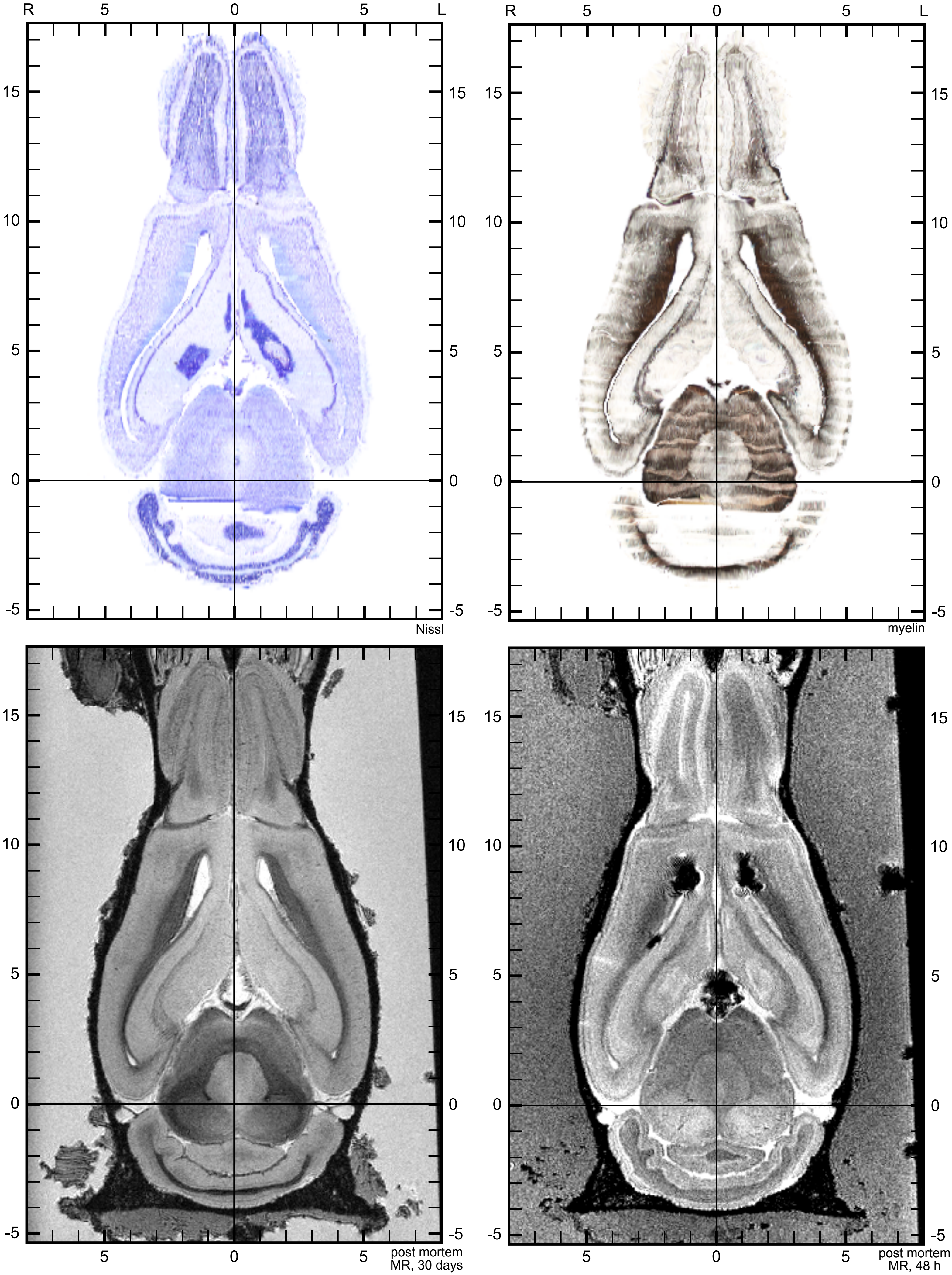

Horizontal cross-section 8 (continued)
3.5 mm ventral from bregma

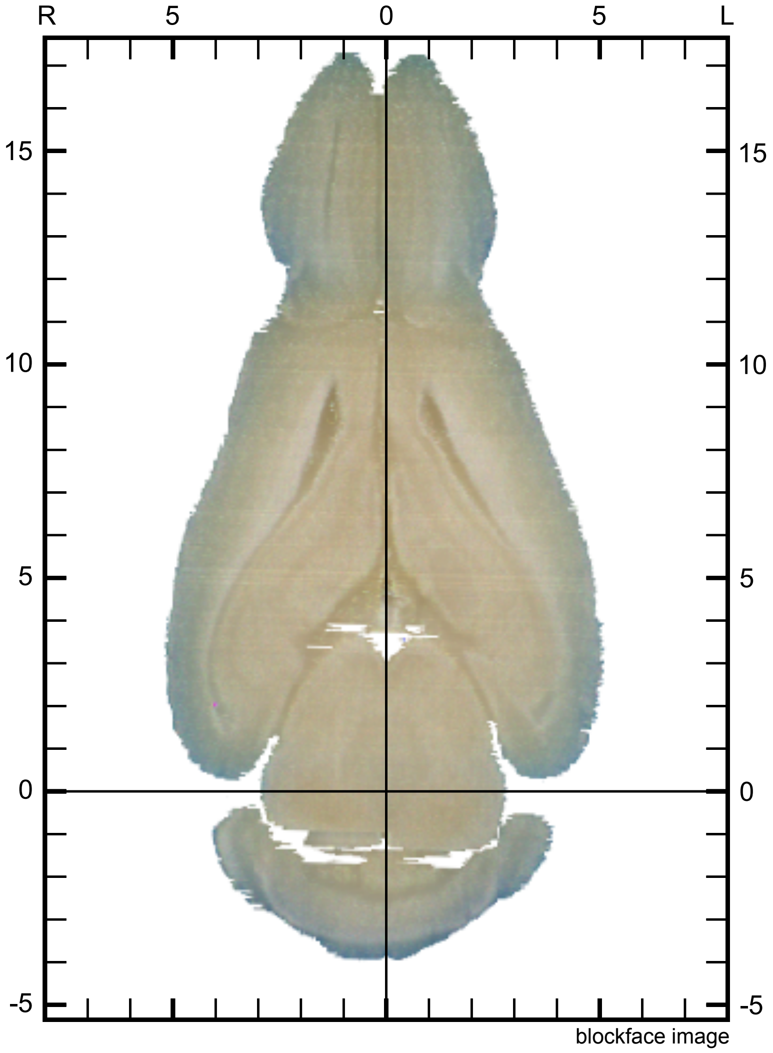

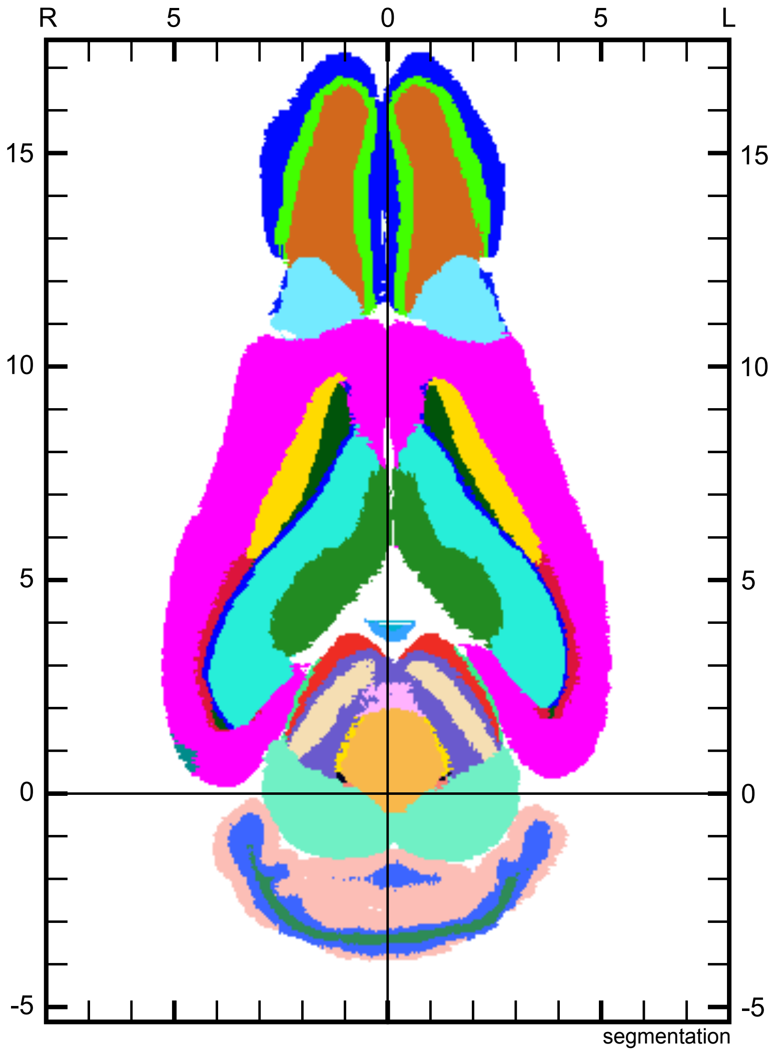

Horizontal cross-section 9
4.0 mm ventral from bregma

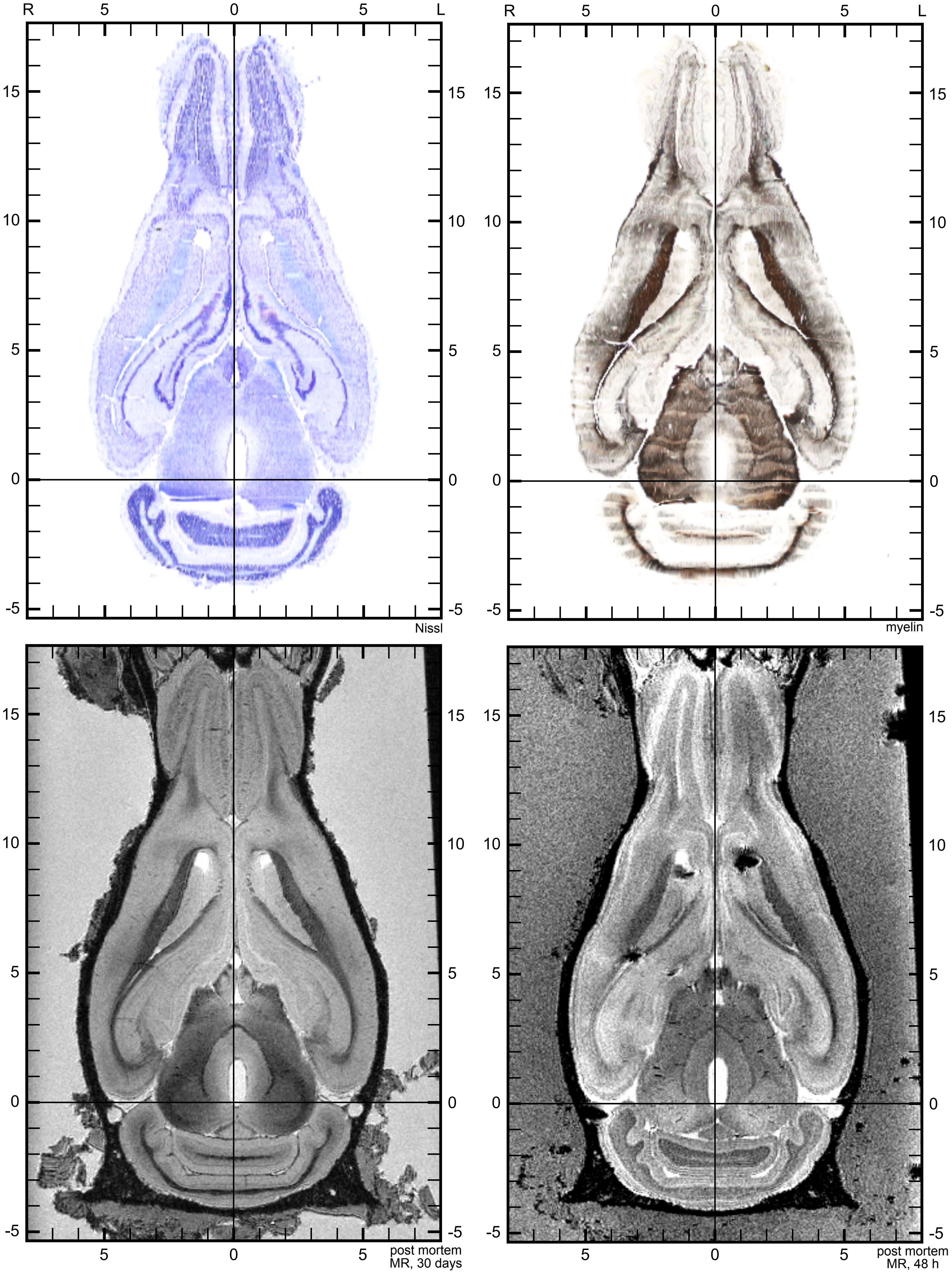

Horizontal cross-section 9 (continued)
4.0 mm ventral from bregma

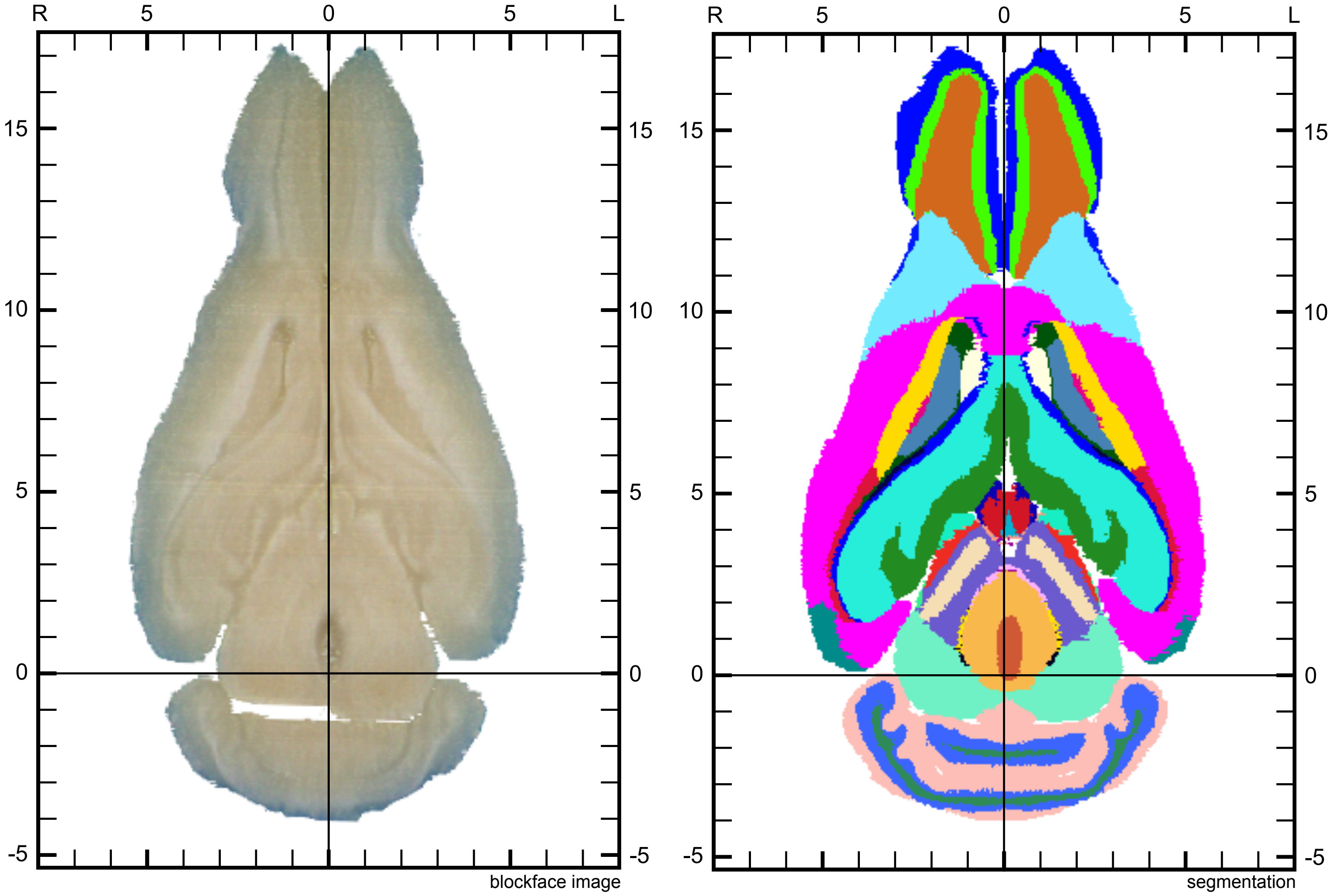

Horizontal cross-section 10
4.5 mm ventral from bregma

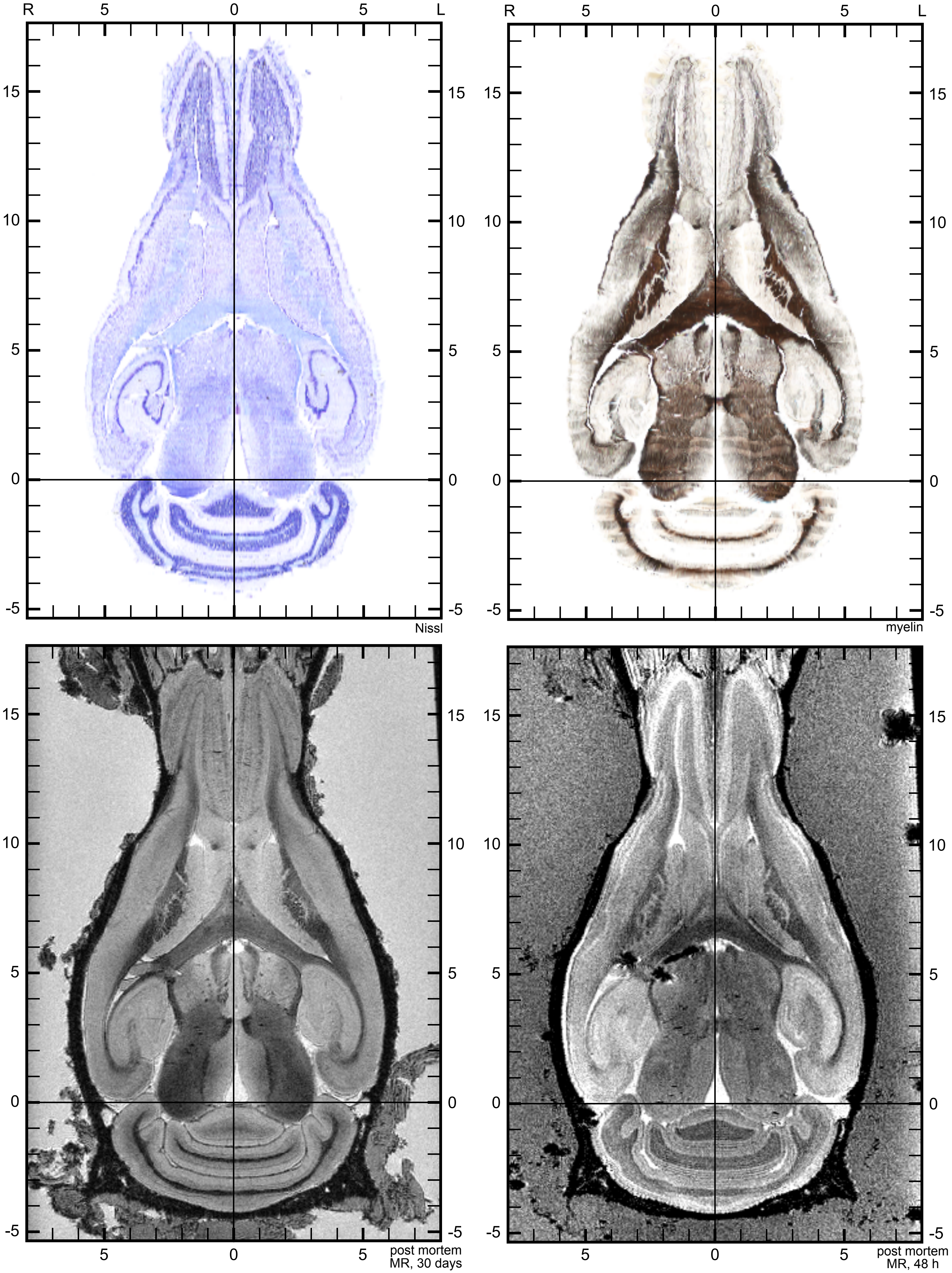

Horizontal cross-section 10 (continued)
4.5 mm ventral from bregma

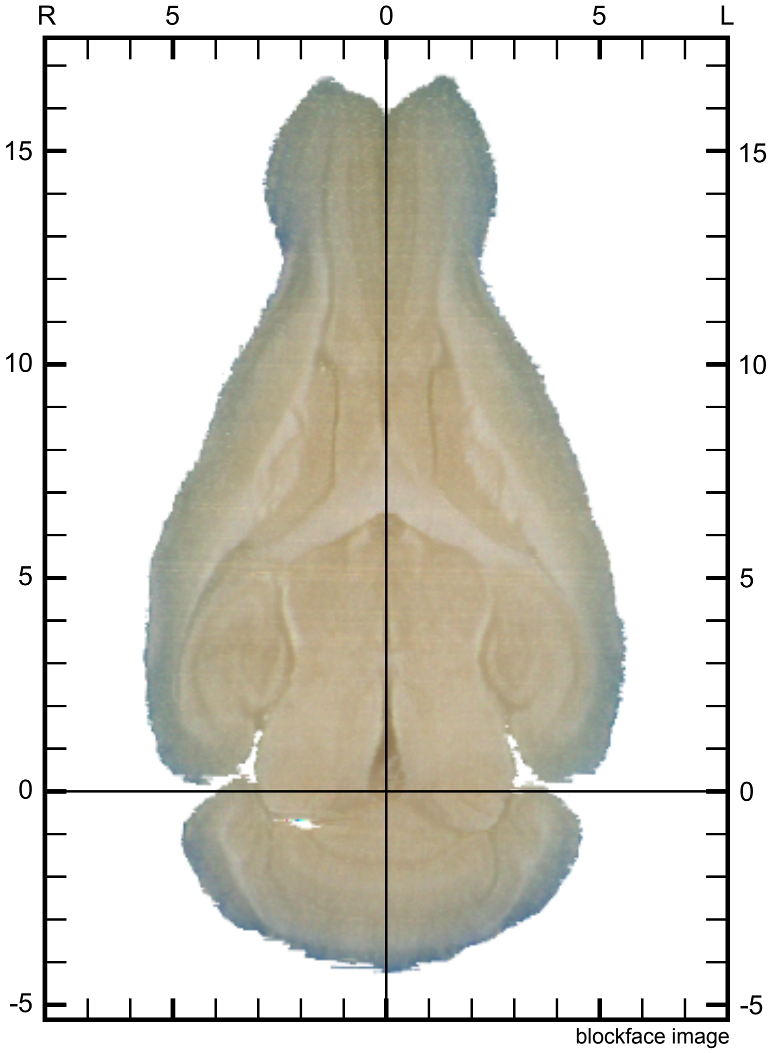

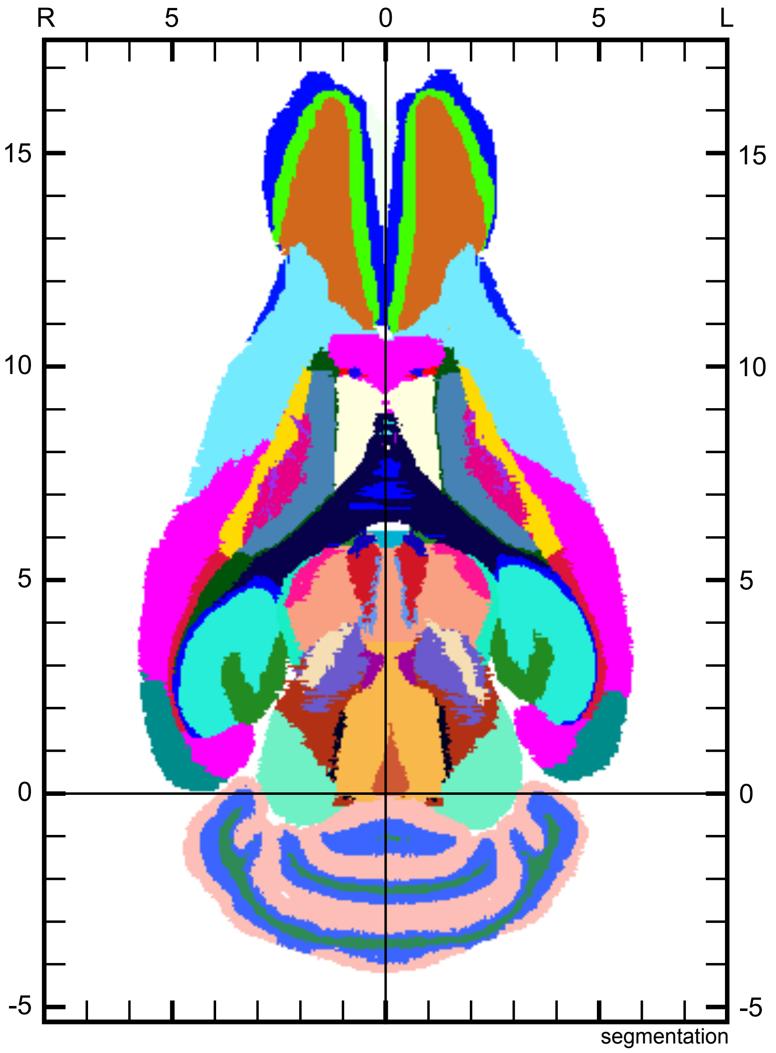

Horizontal cross-section 11
5.0 mm ventral from bregma

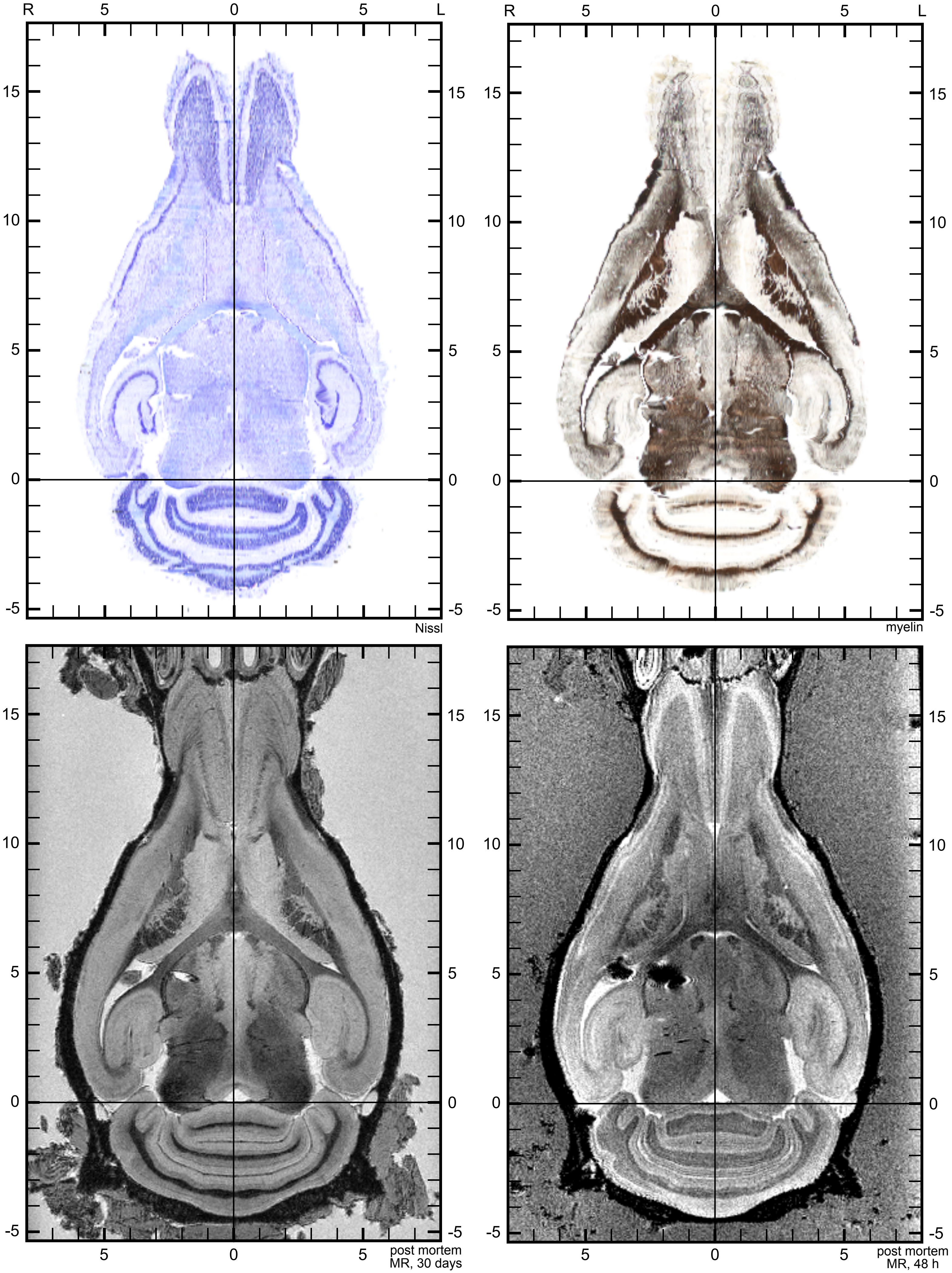

Horizontal cross-section 11 (continued)
5.0 mm ventral from bregma

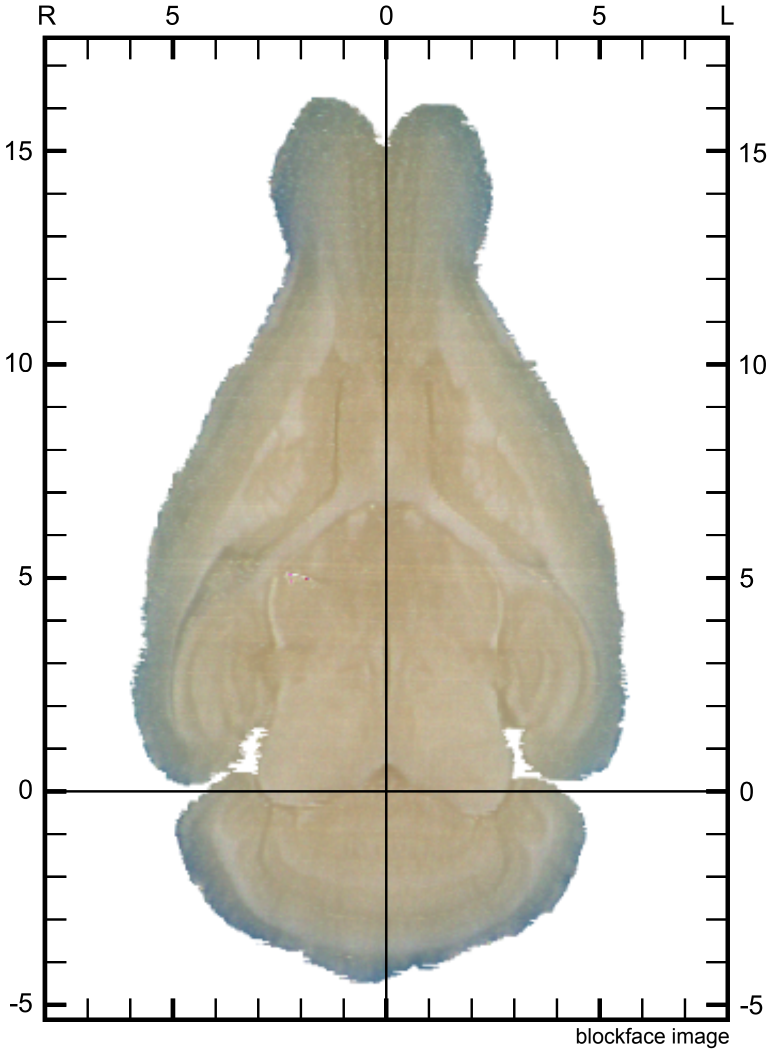

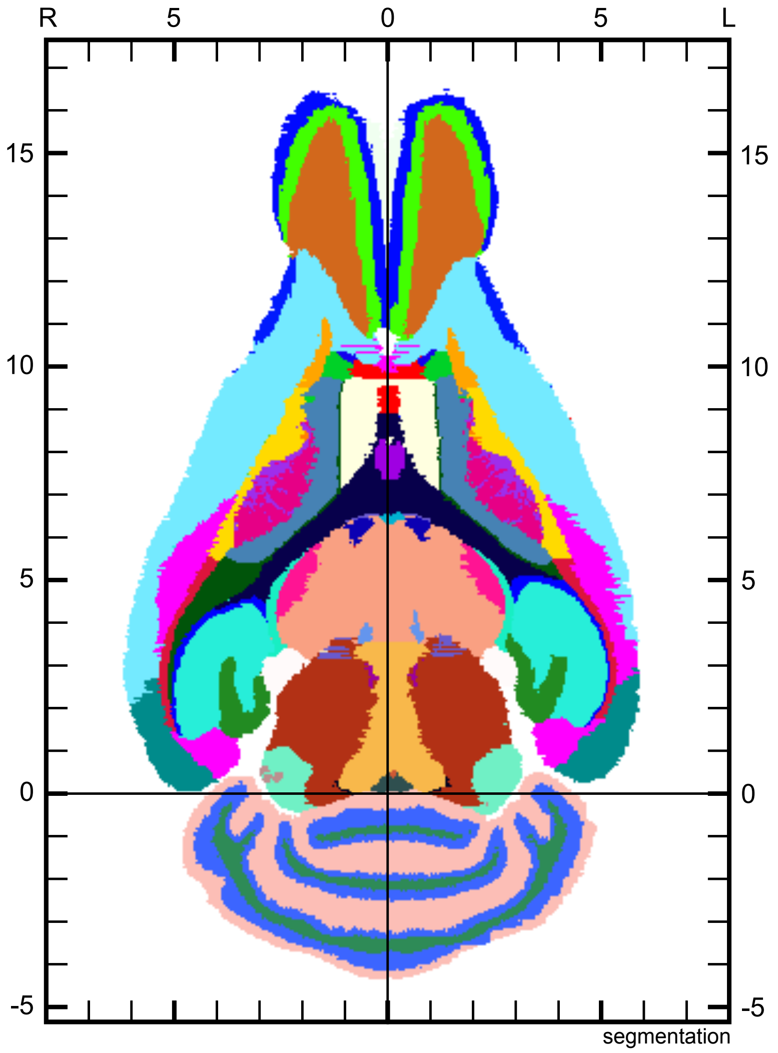

Horizontal cross-section 12
5.5 mm ventral from bregma

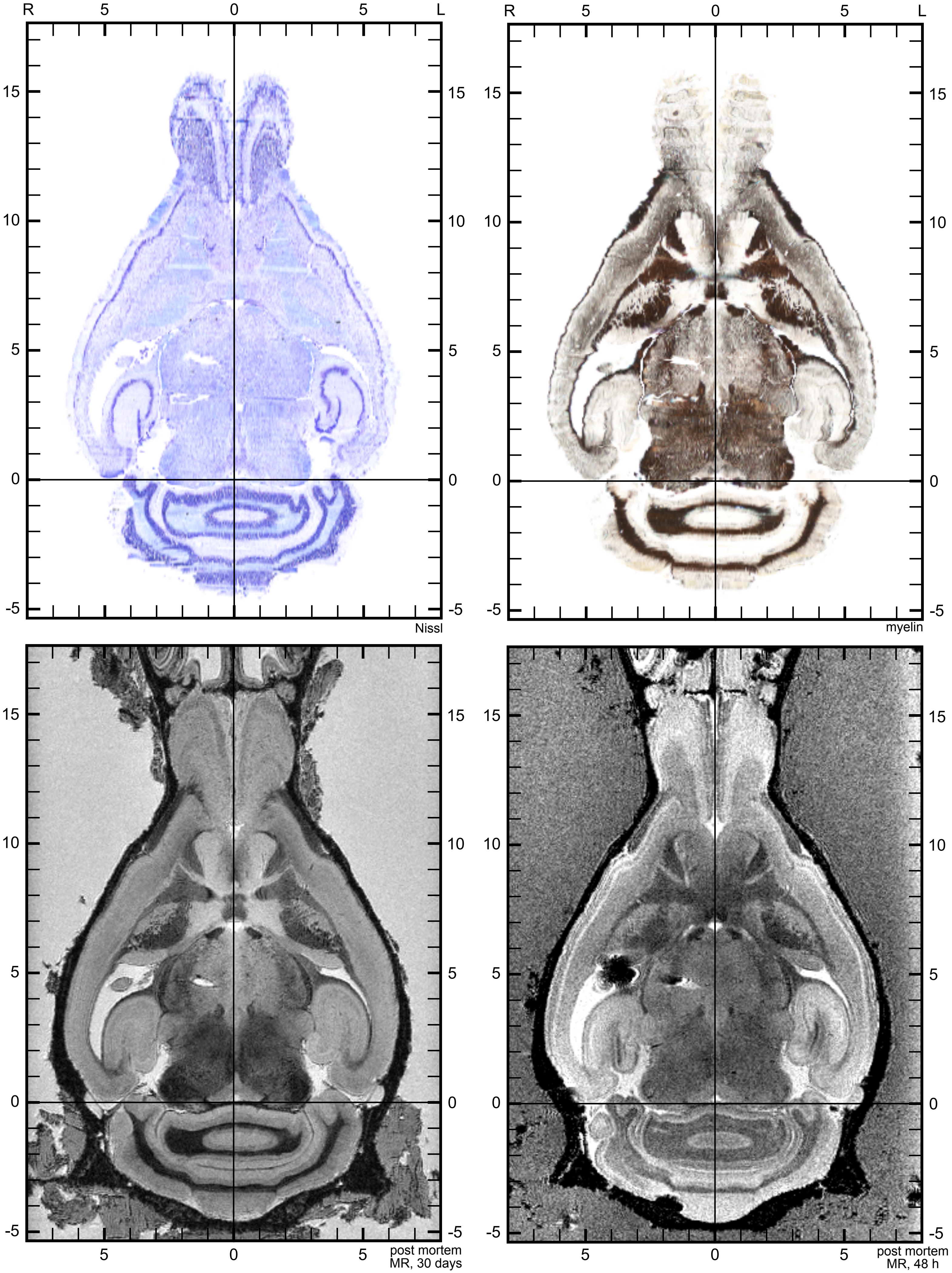

Horizontal cross-section 12 (continued)
5.5 mm ventral from bregma

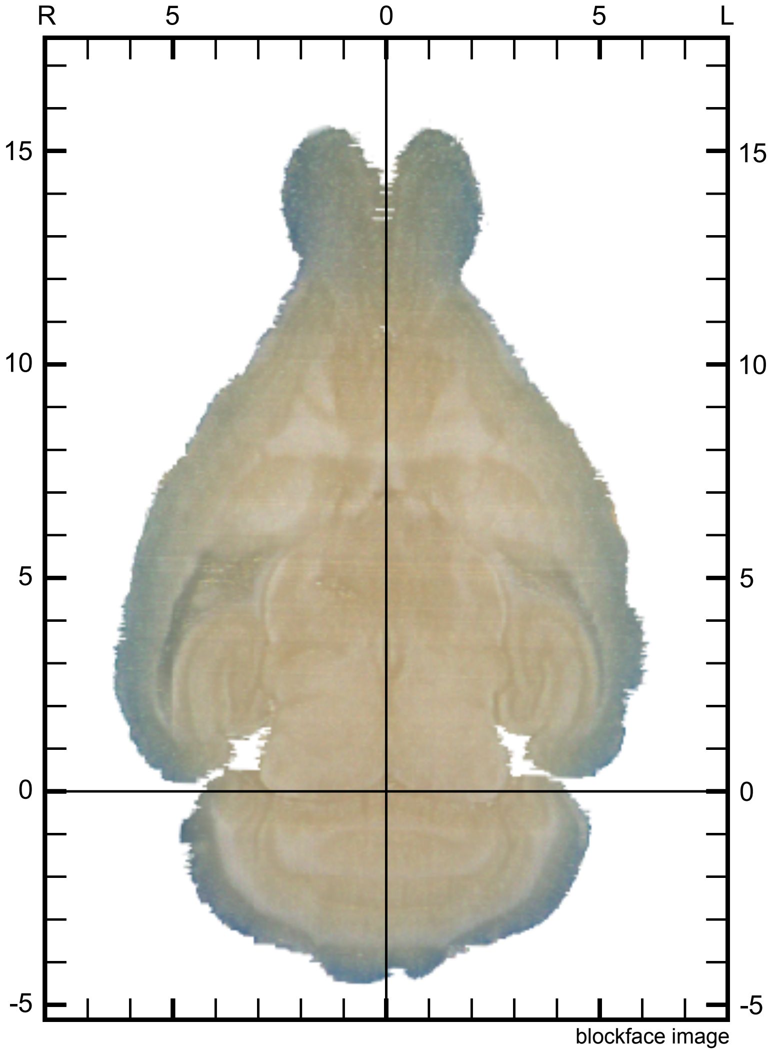

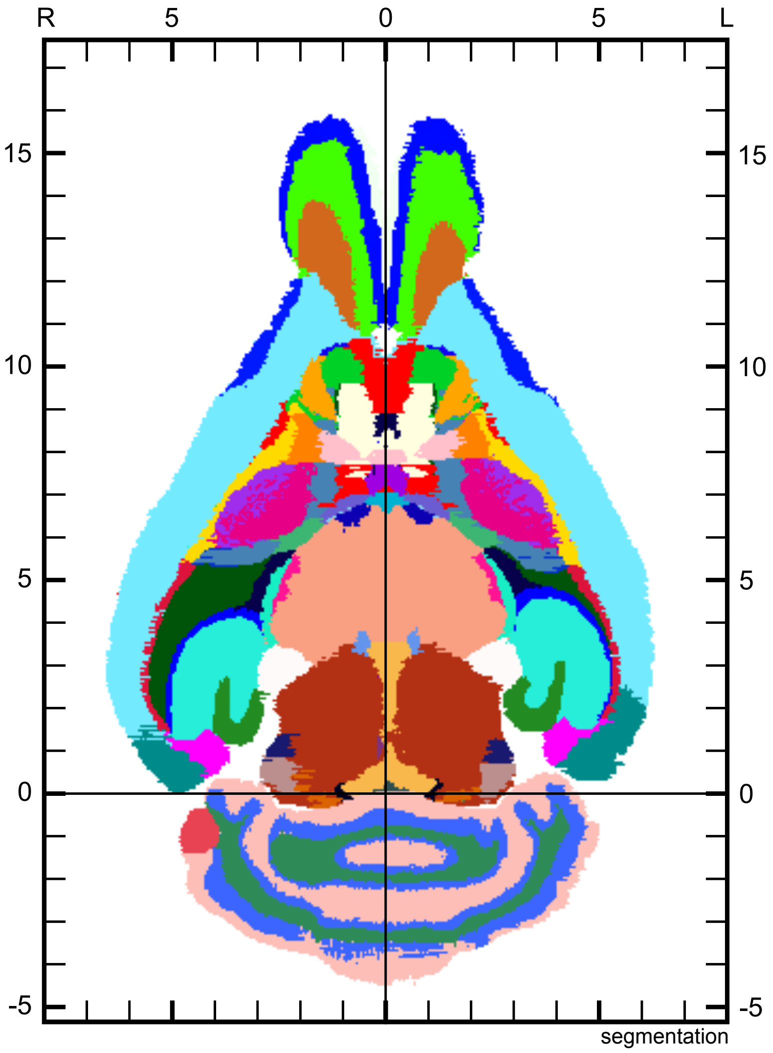

Horizontal cross-section 13
6.0 mm ventral from bregma

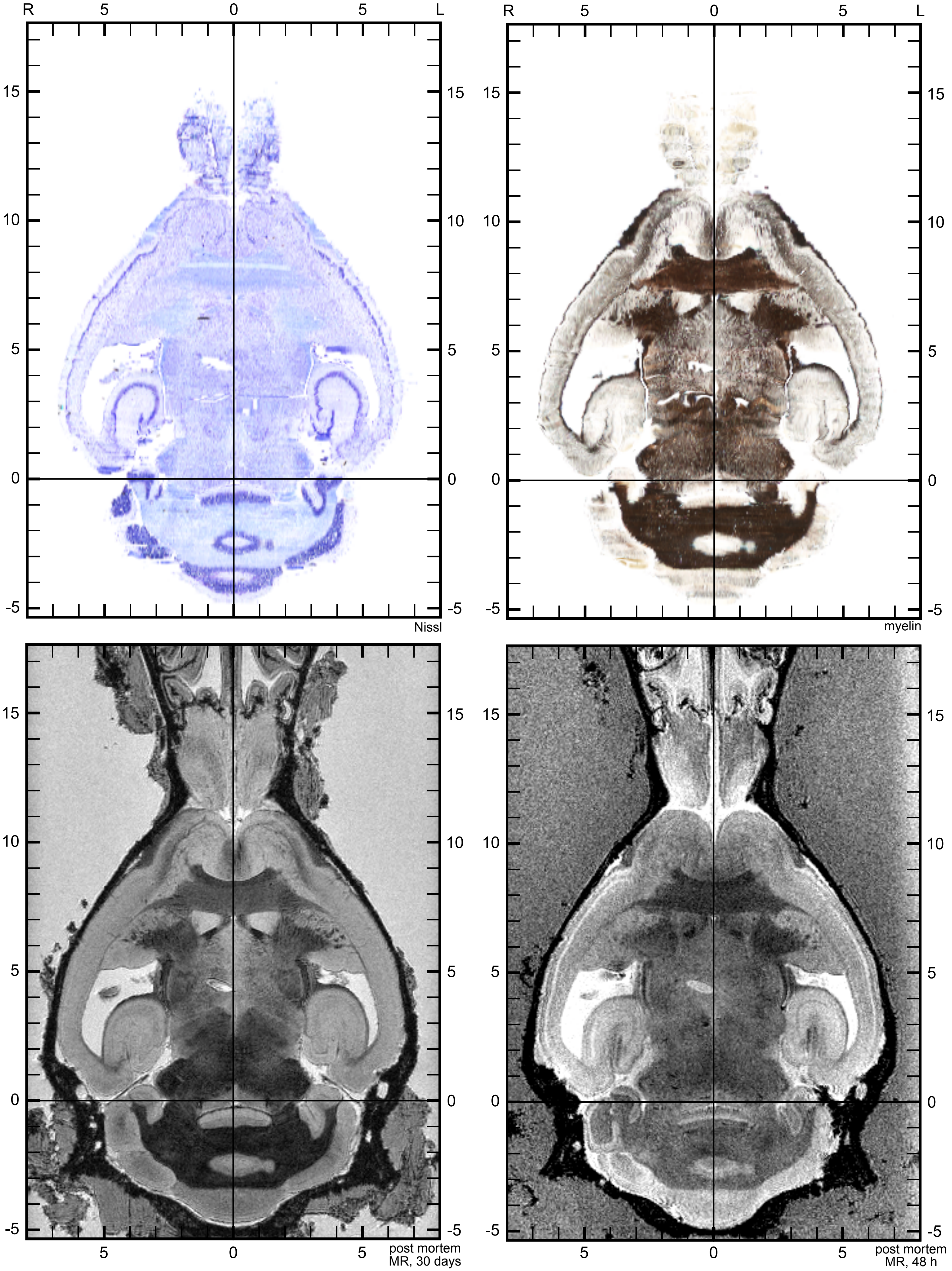

Horizontal cross-section 13 (continued)
6.0 mm ventral from bregma

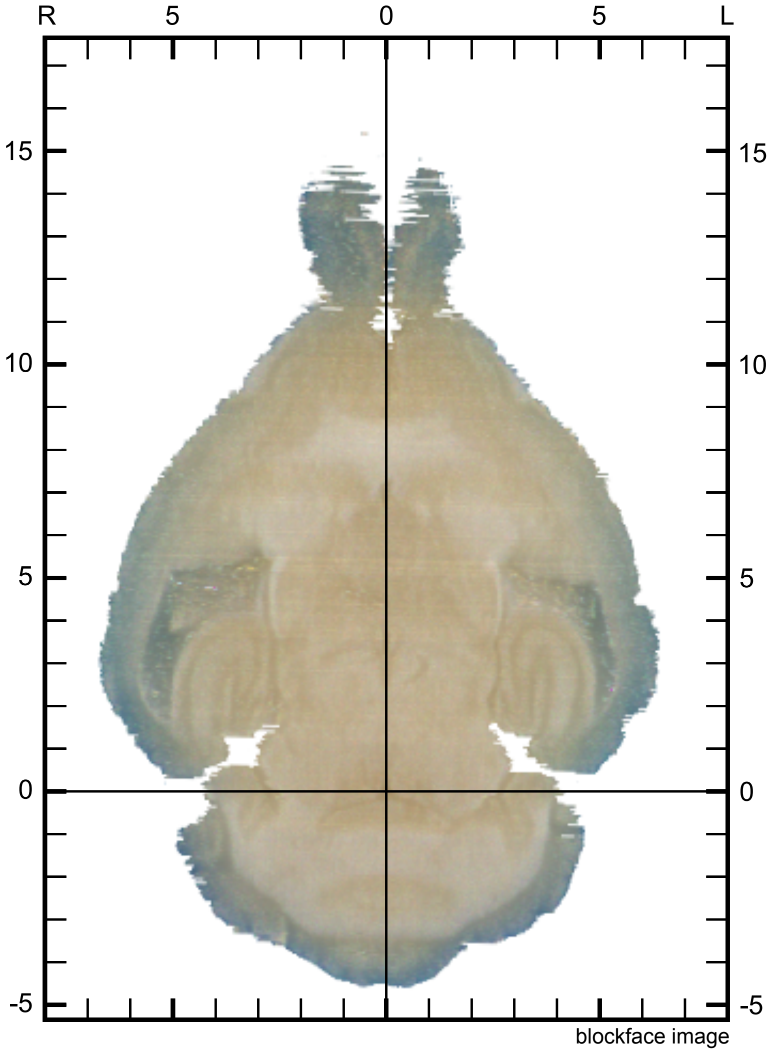

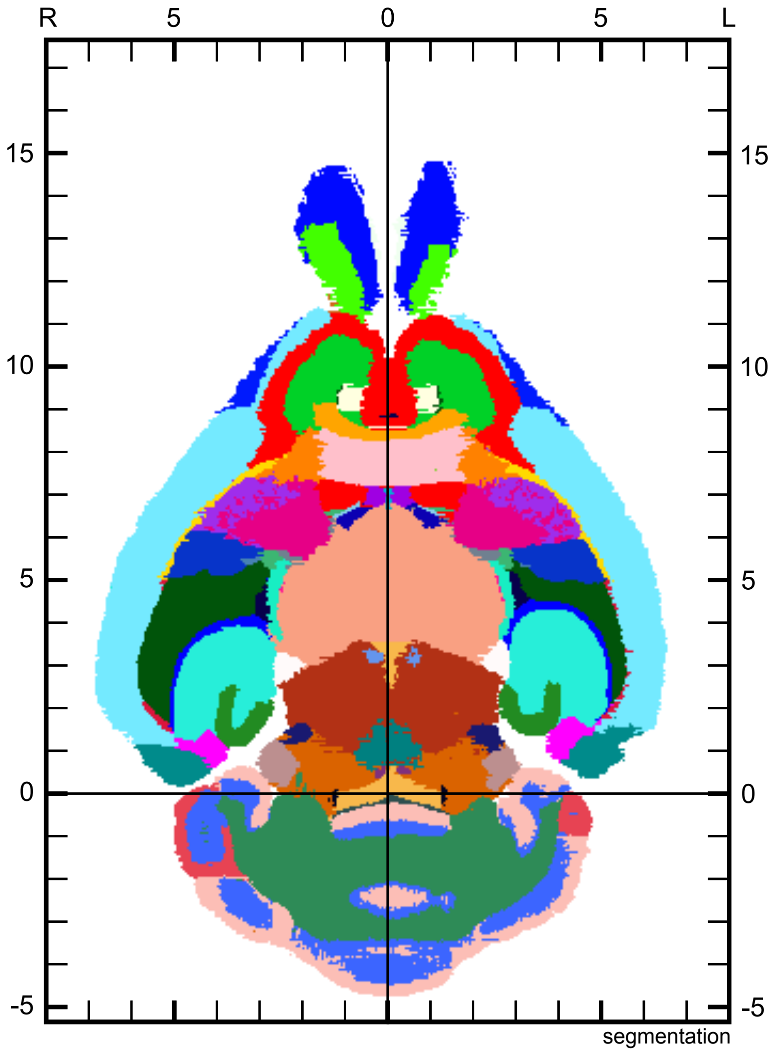

Horizontal cross-section 14
6.5 mm ventral from bregma

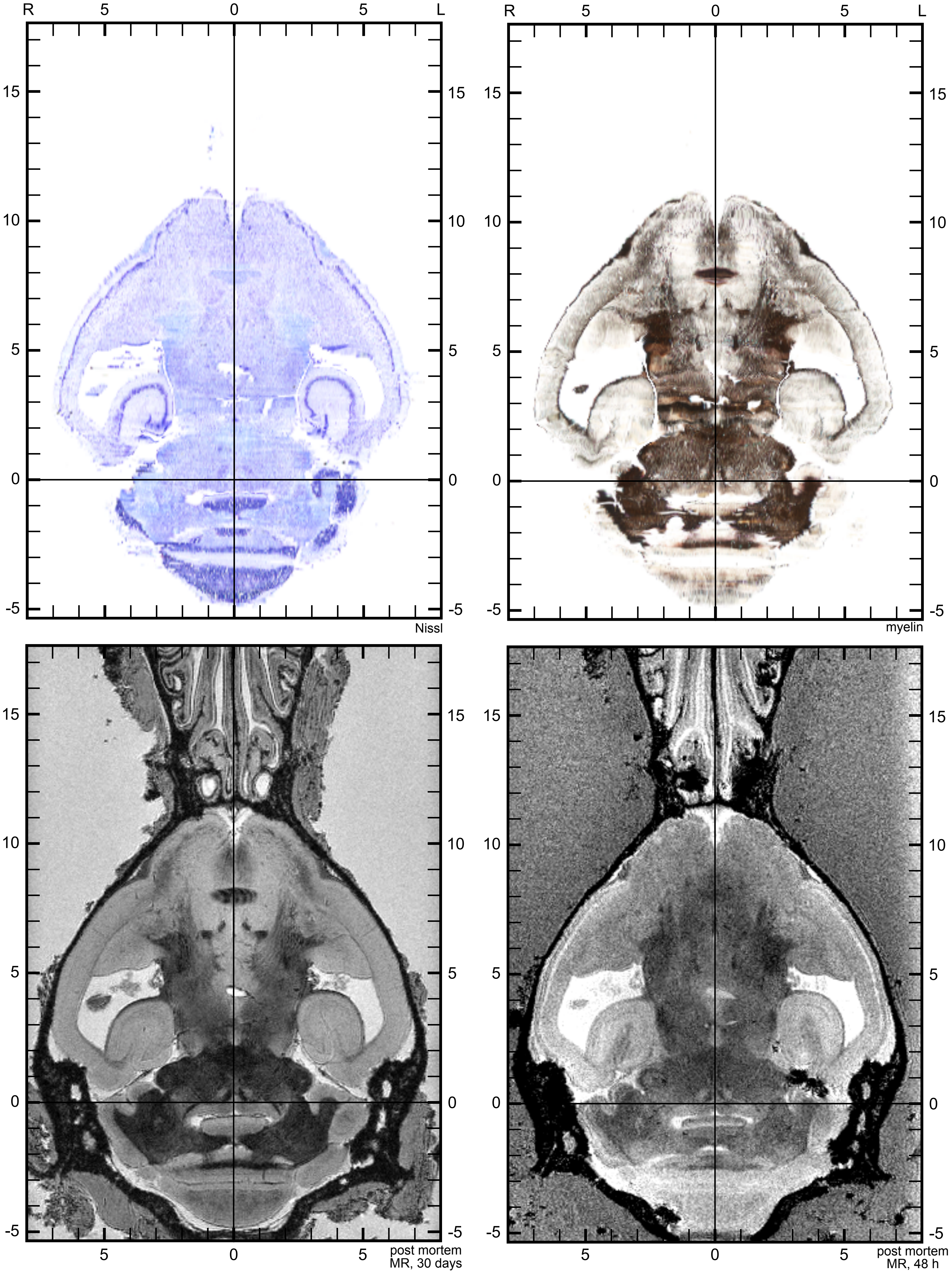

Horizontal cross-section 14 (continued)
6.5 mm ventral from bregma

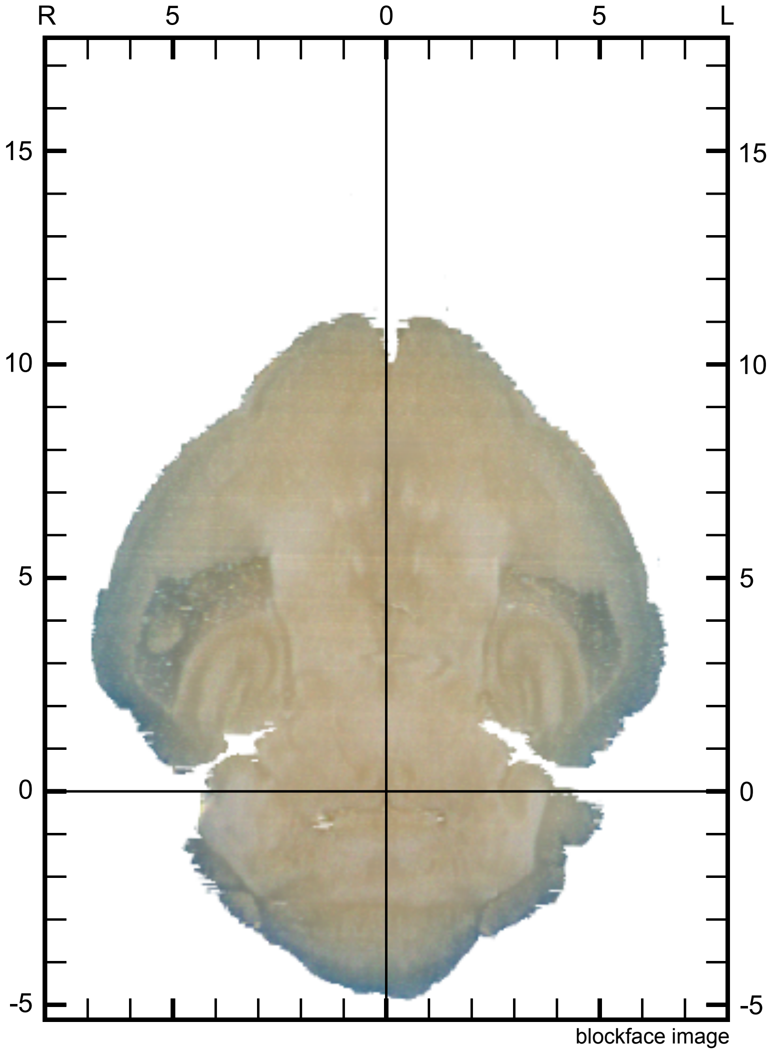

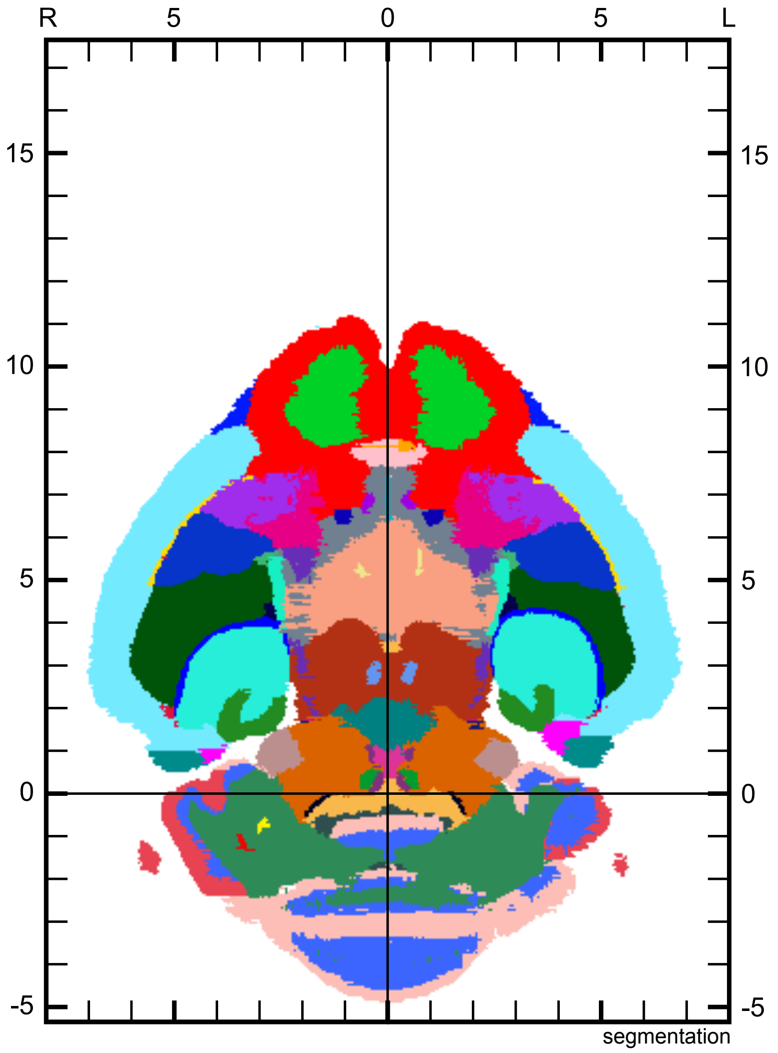

Horizontal cross-section 15
7.0 mm ventral from bregma

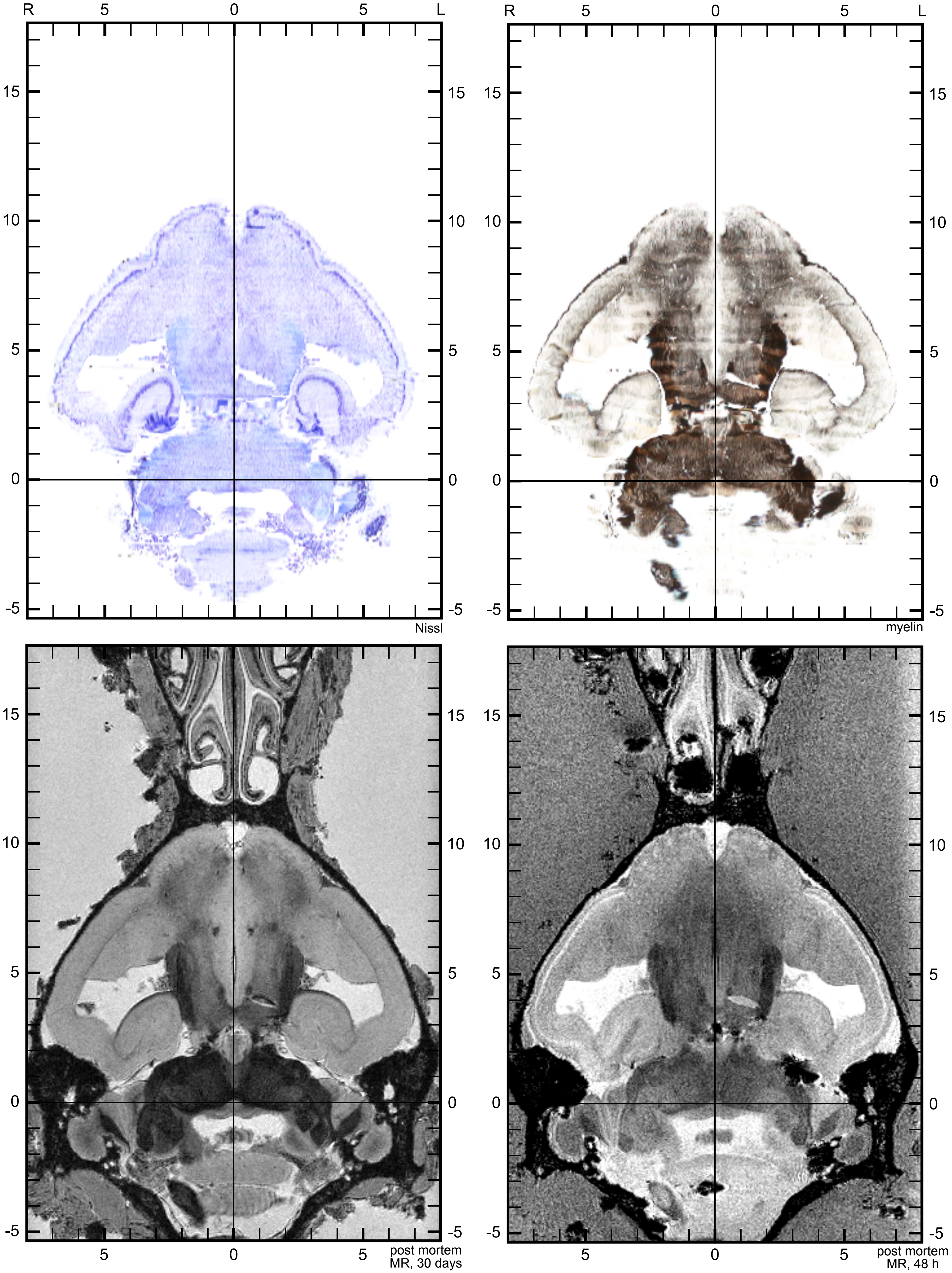

Horizontal cross-section 15 (continued)
7.0 mm ventral from bregma

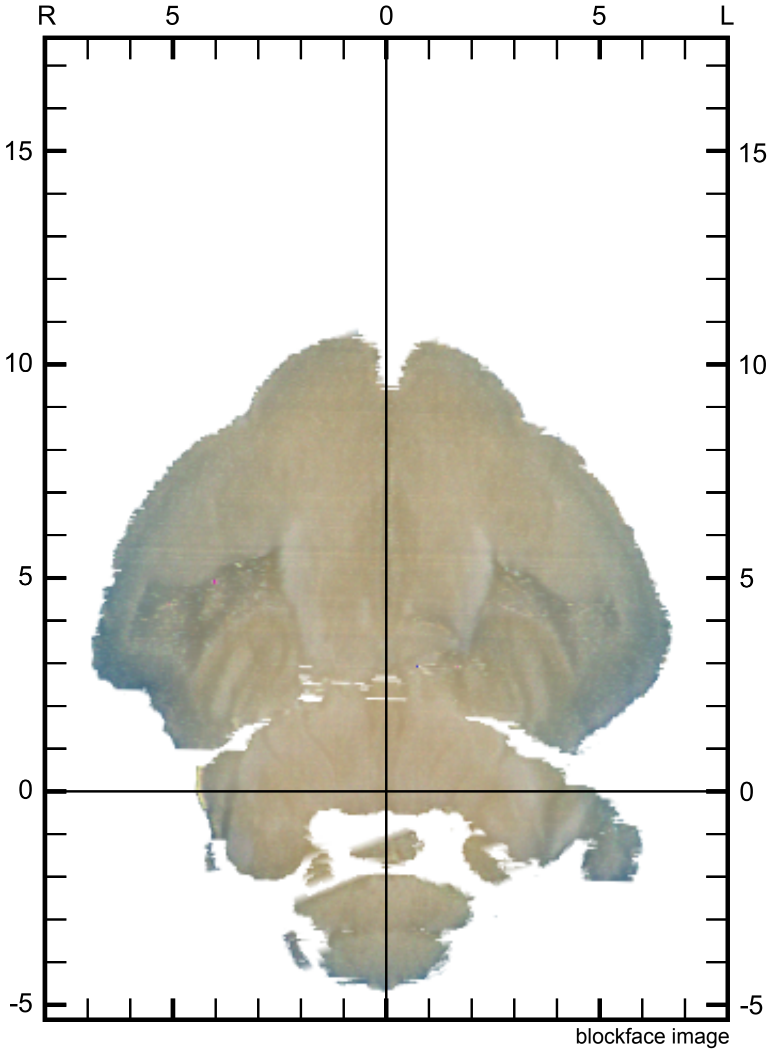

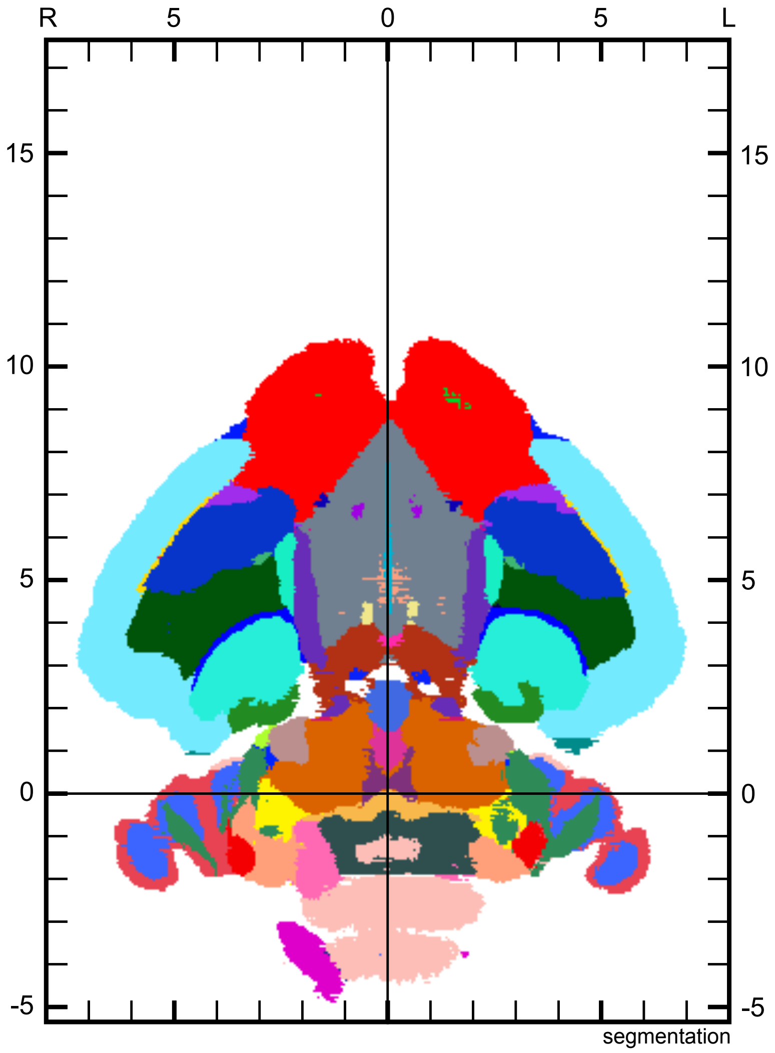

Horizontal cross-section 16
7.5 mm ventral from bregma

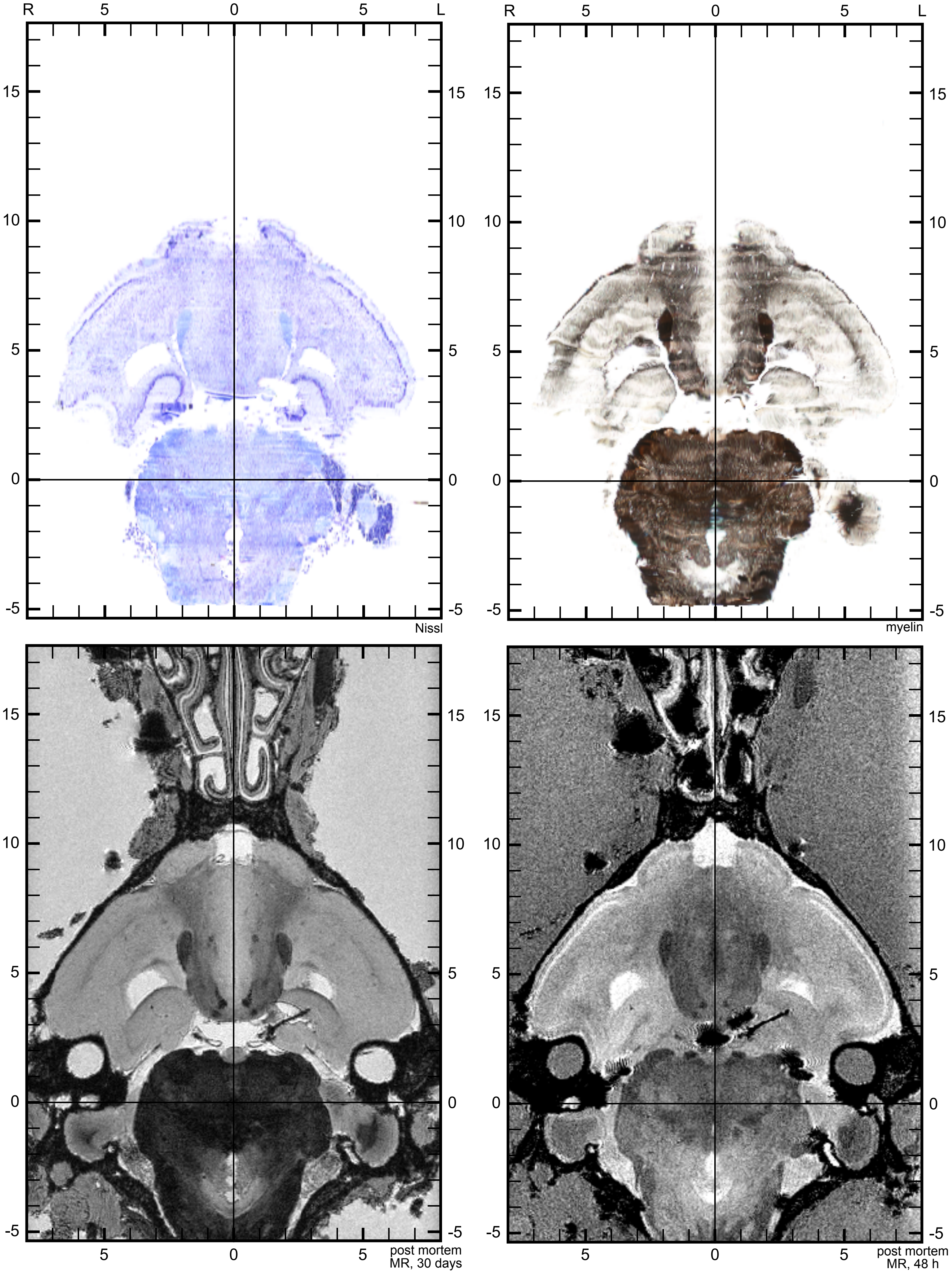

Horizontal cross-section 16 (continued)
7.5 mm ventral from bregma

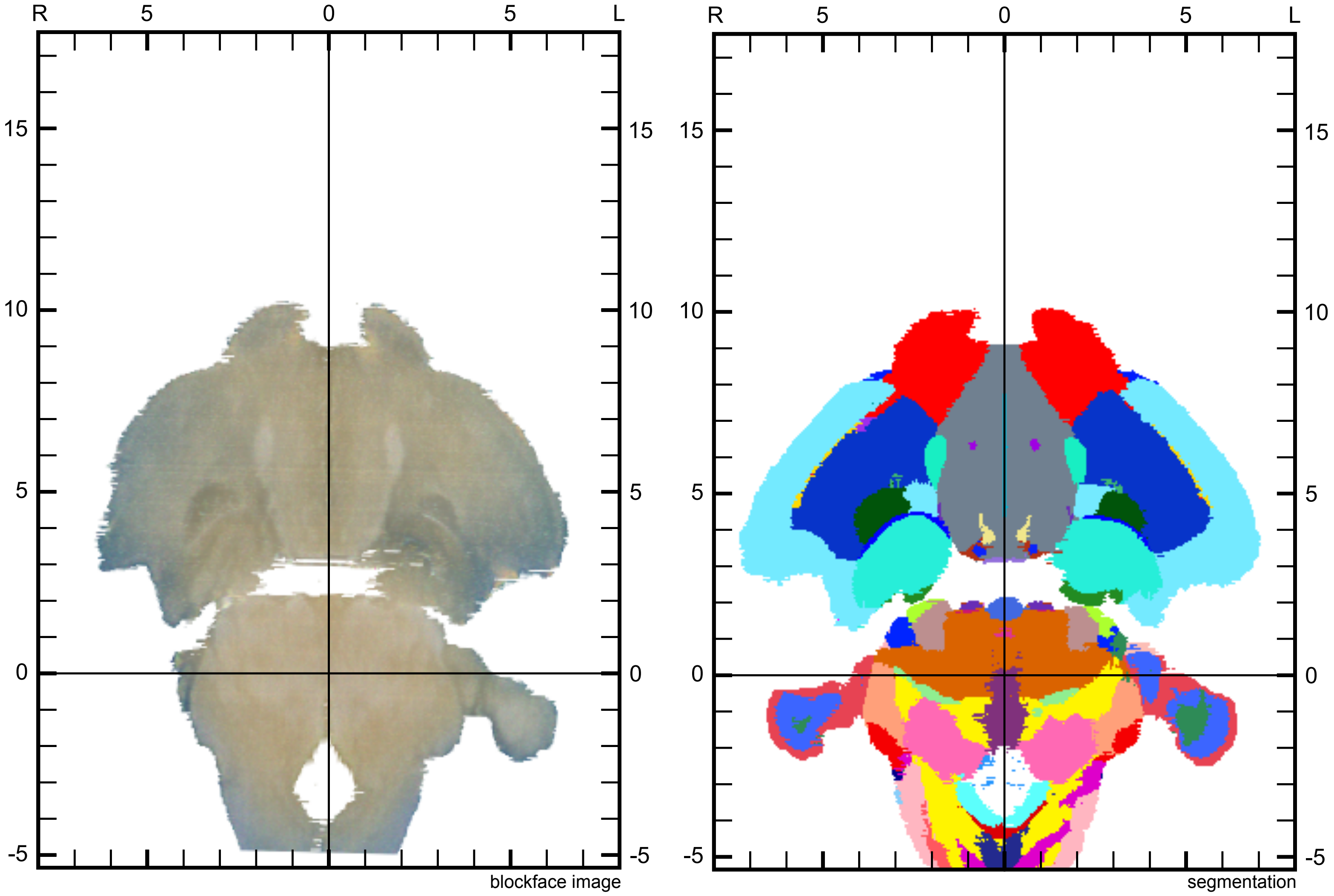

Horizontal cross-section 17
8.0 mm ventral from bregma

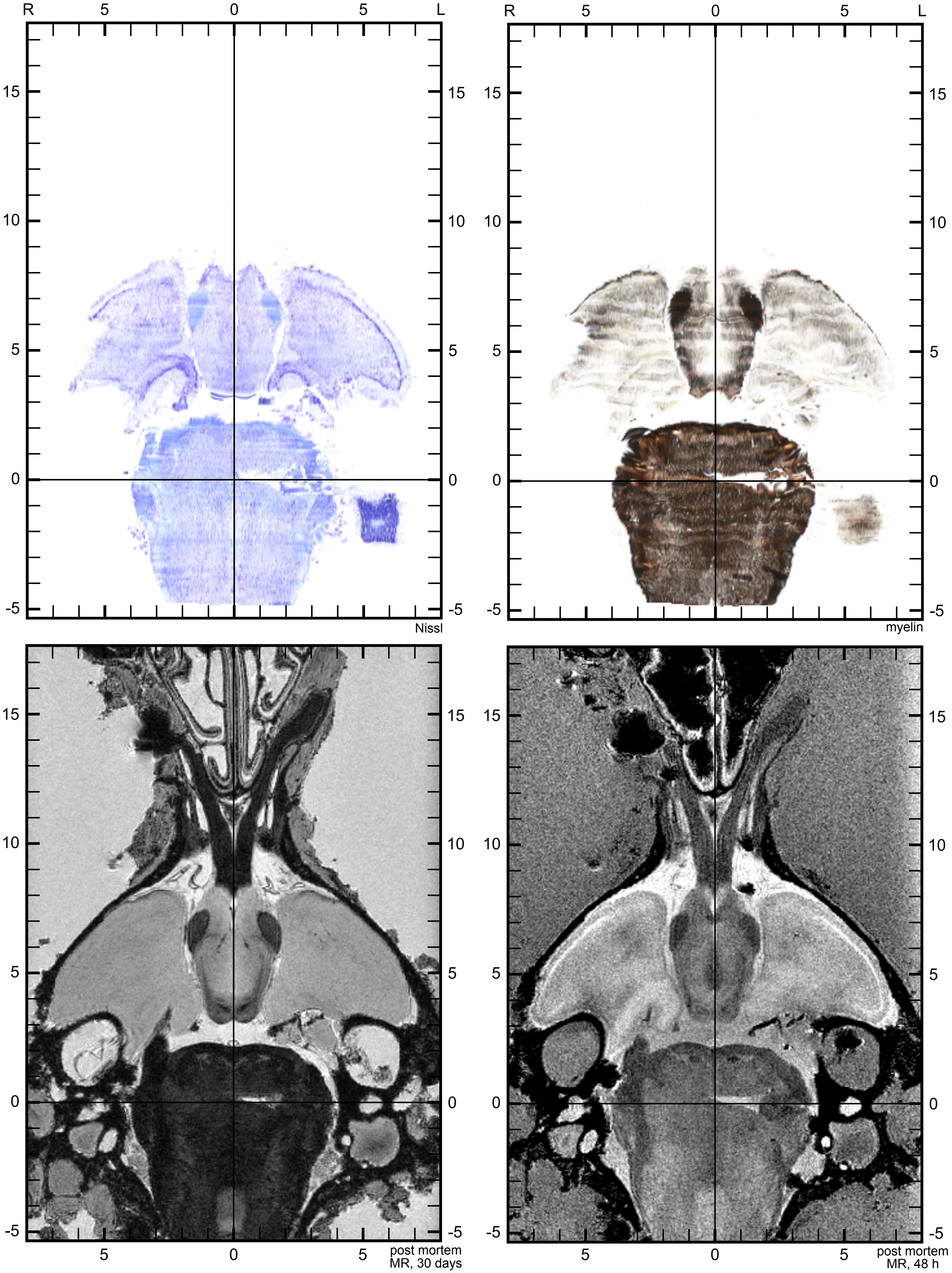

Horizontal cross-section 17 (continued)
8.0 mm ventral from bregma

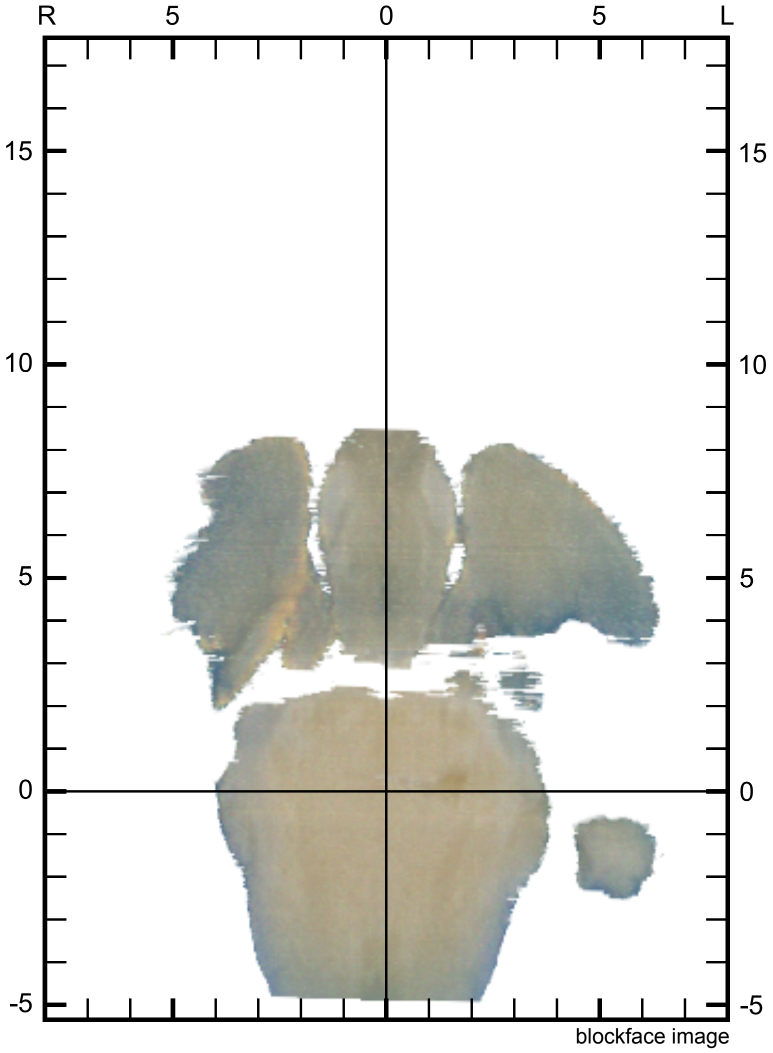

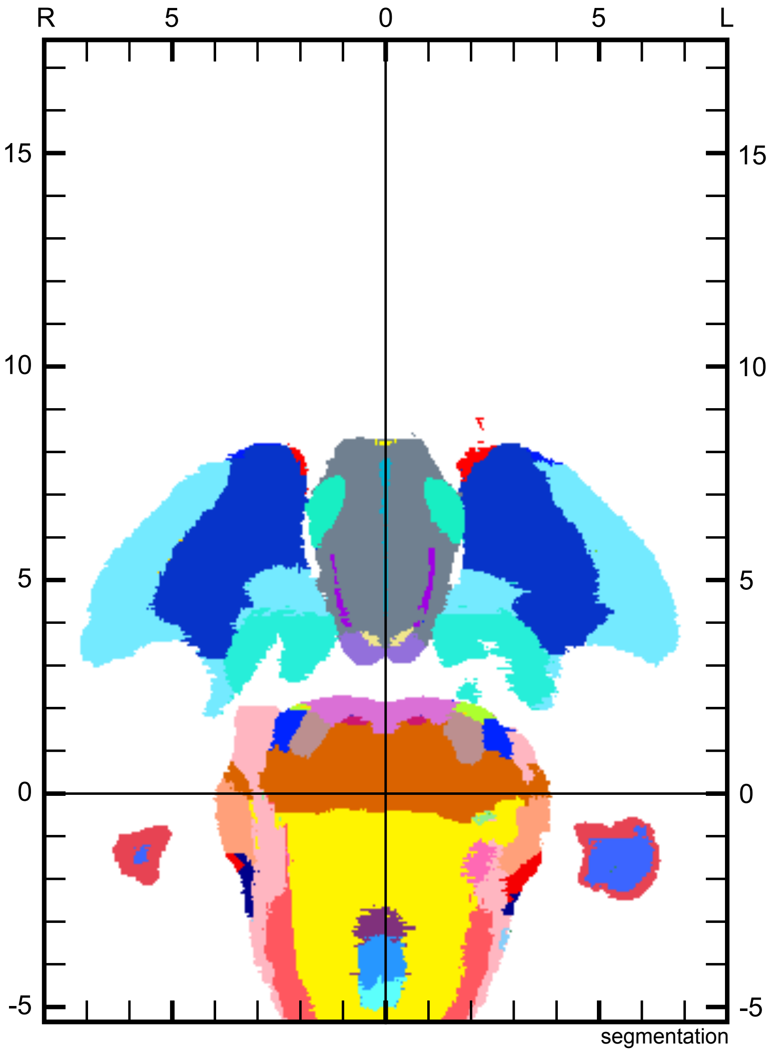

Horizontal cross-section 18
8.5 mm ventral from bregma

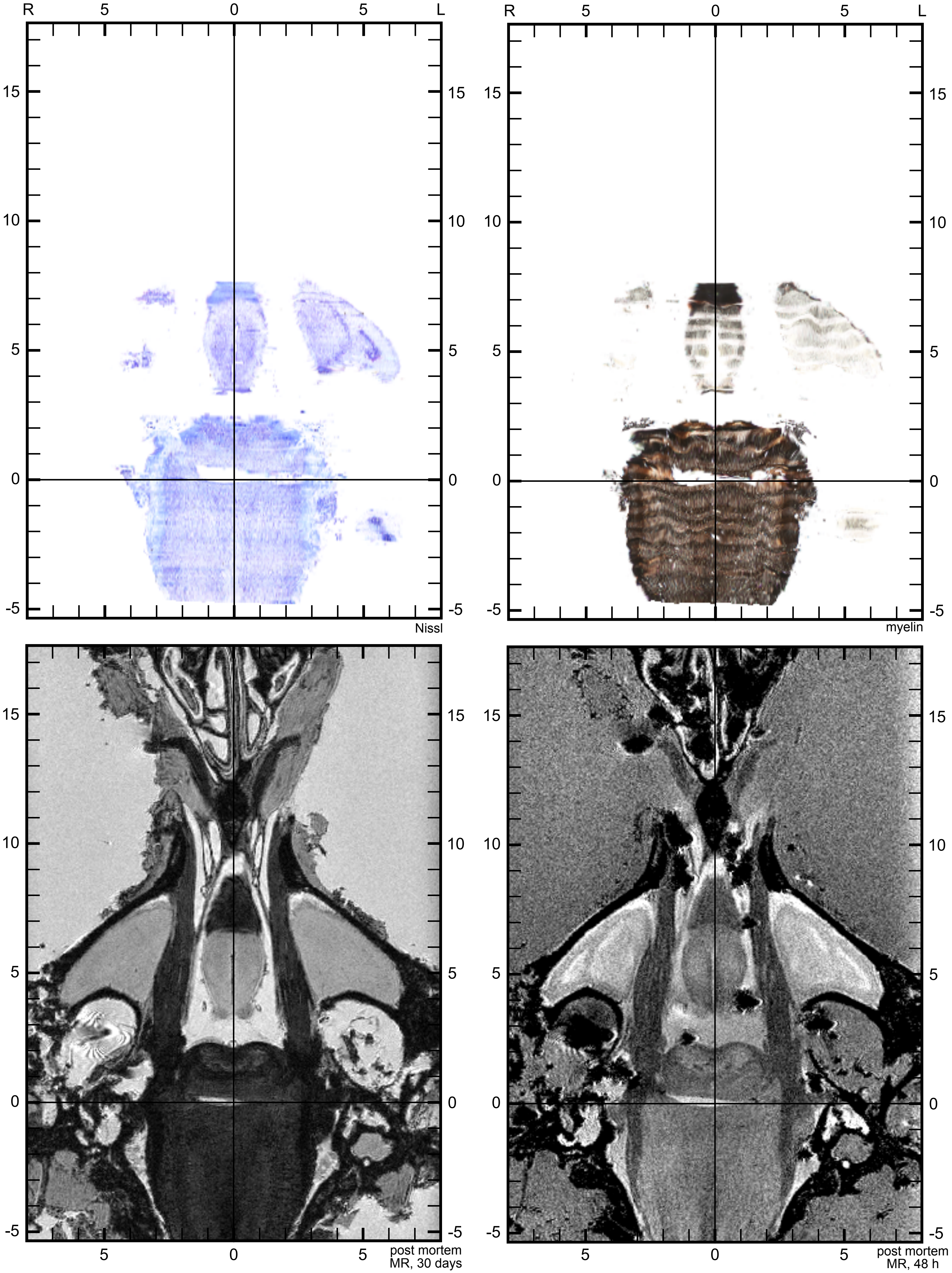

Horizontal cross-section 18 (continued)
8.5 mm ventral from bregma

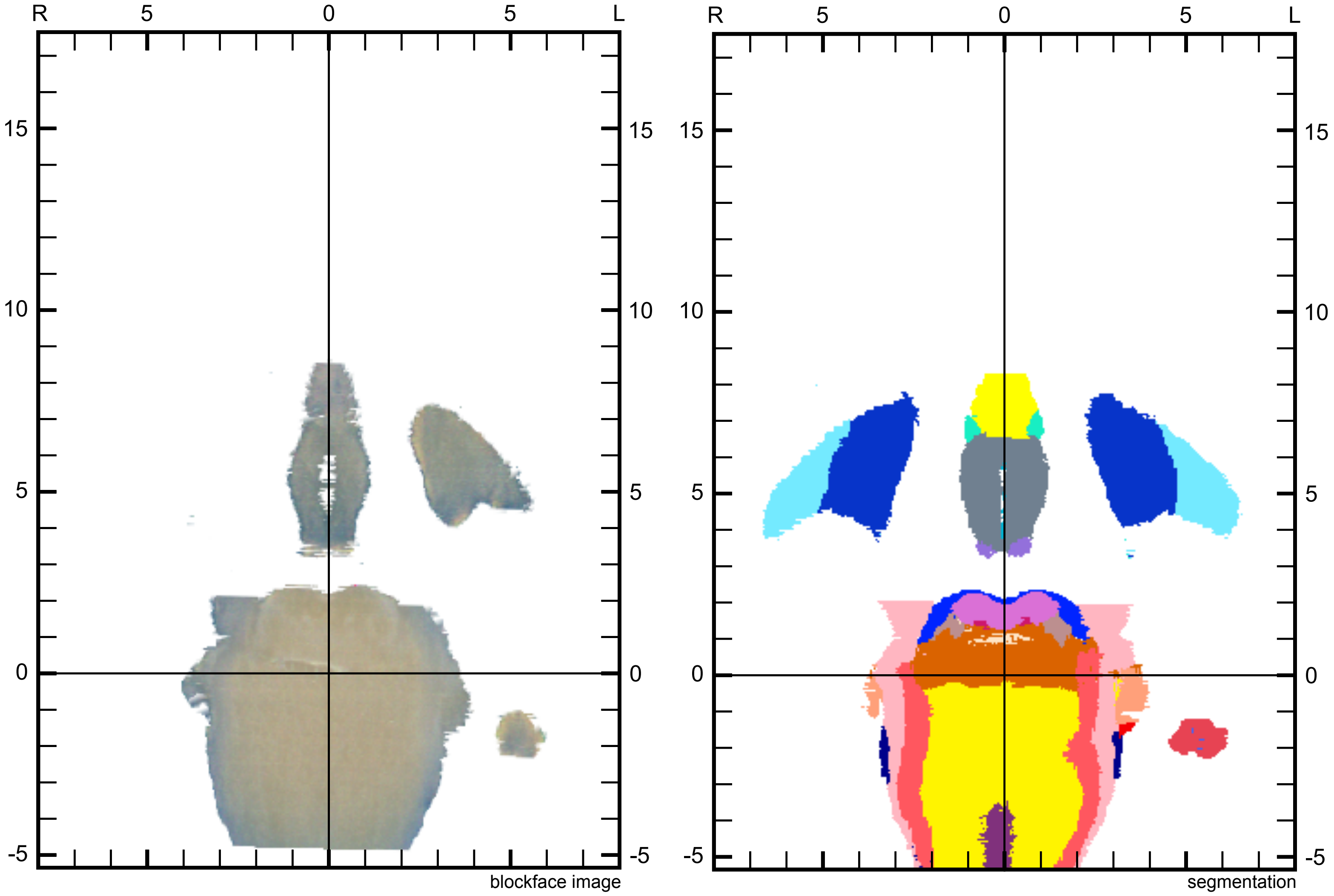

Horizontal cross-section 19
9.0 mm ventral from bregma

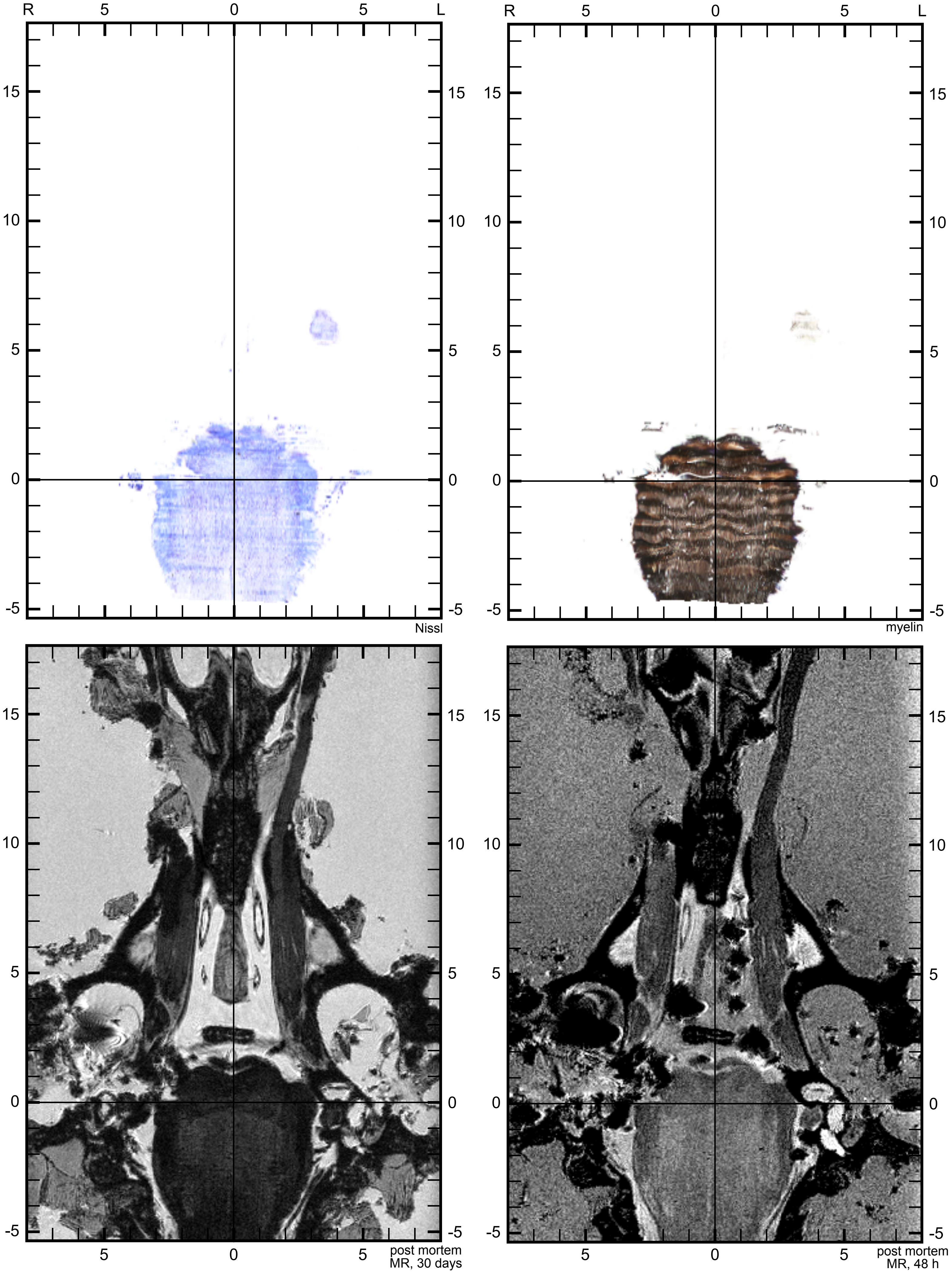

Horizontal cross-section 19 (continued)
9.0 mm ventral from bregma

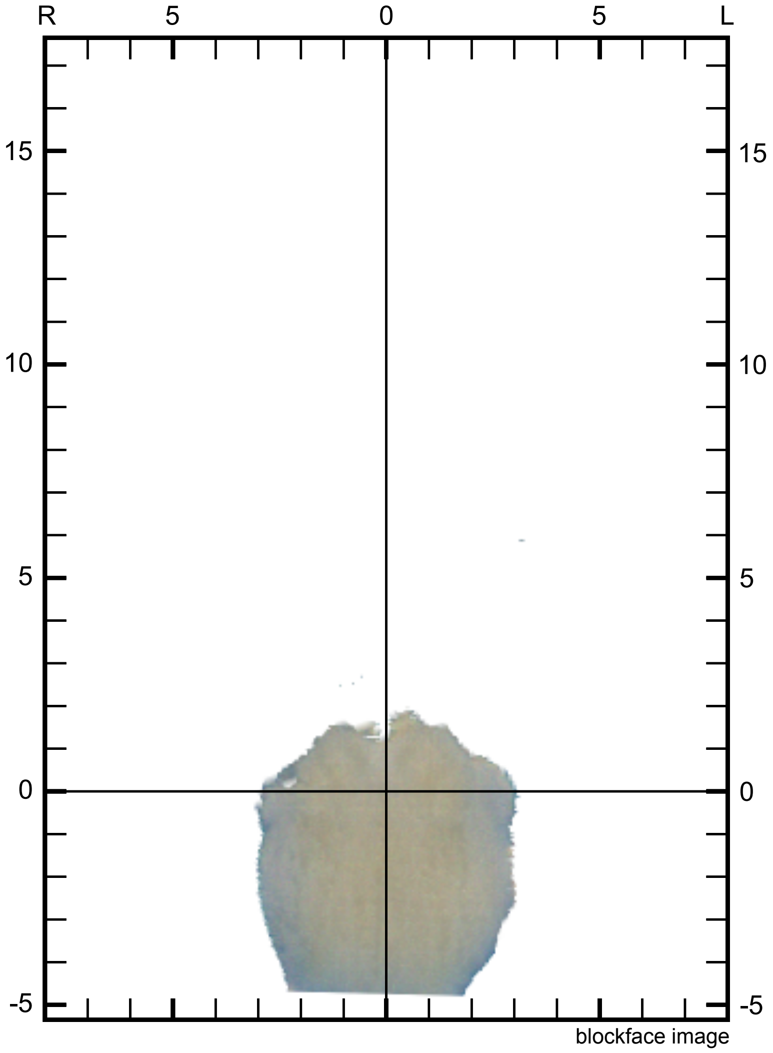

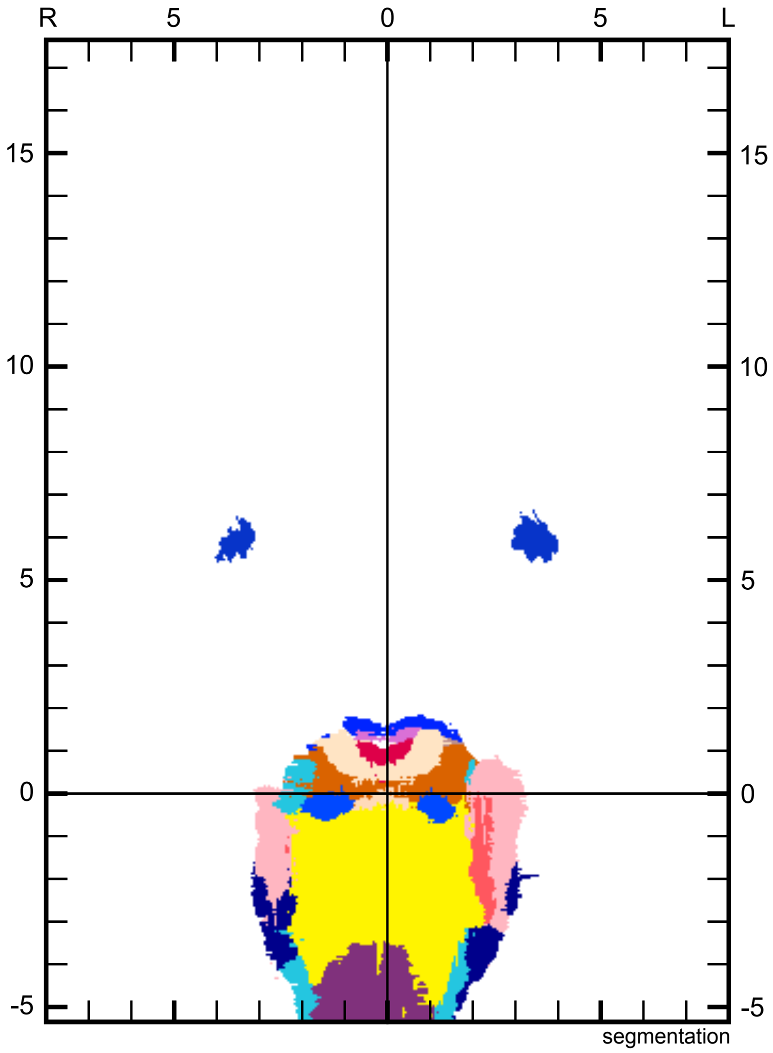

Horizontal cross-section 20
9.5 mm ventral from bregma

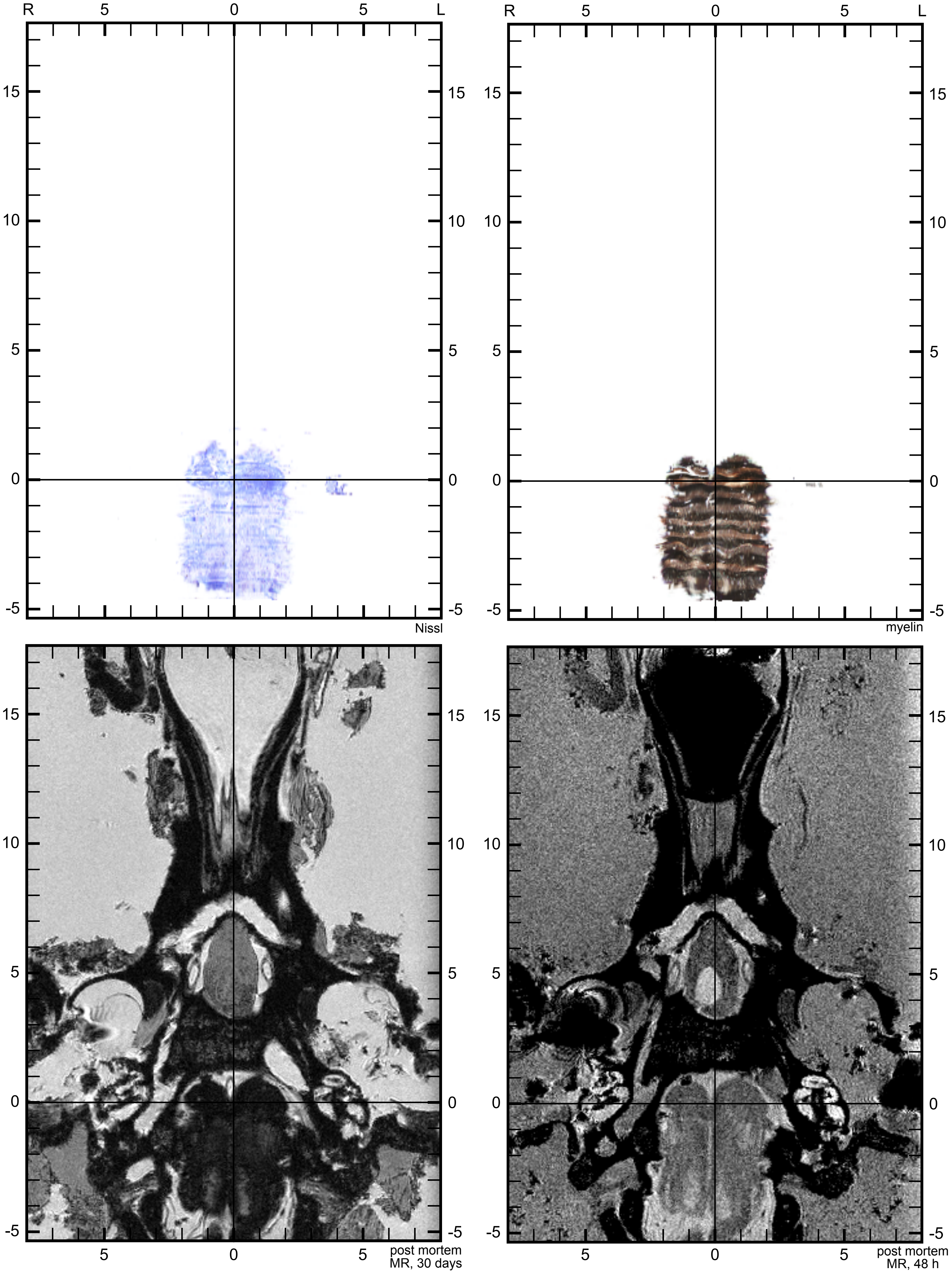

Horizontal cross-section 20 (continued)
9.5 mm ventral from bregma

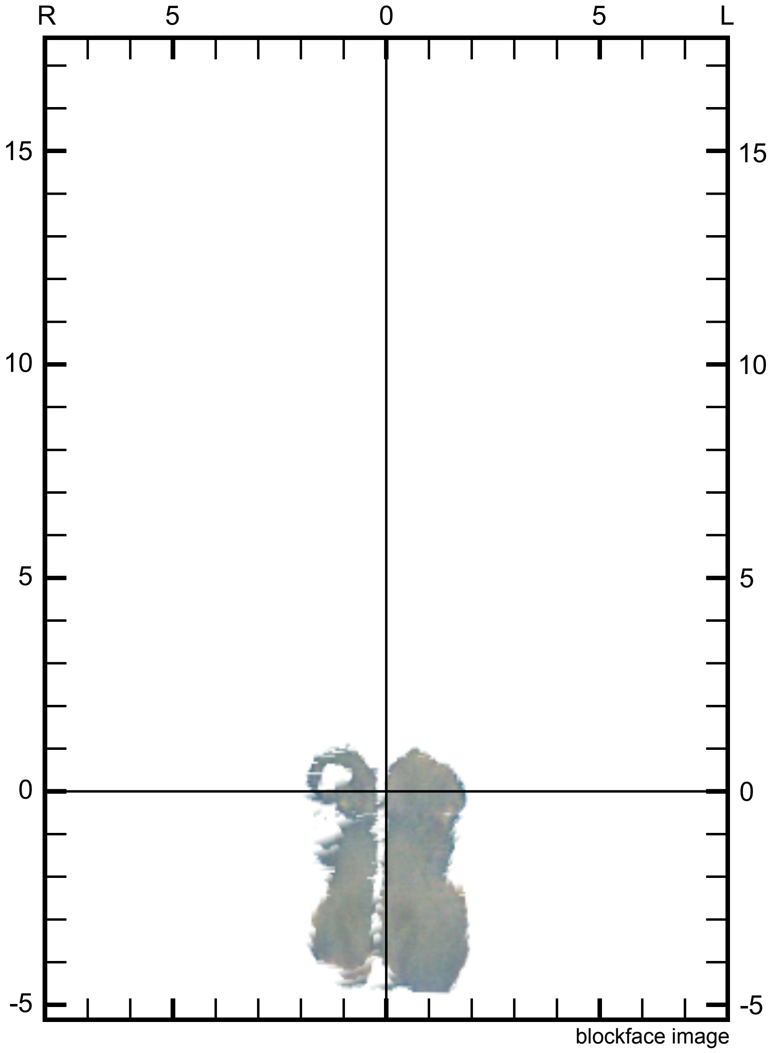

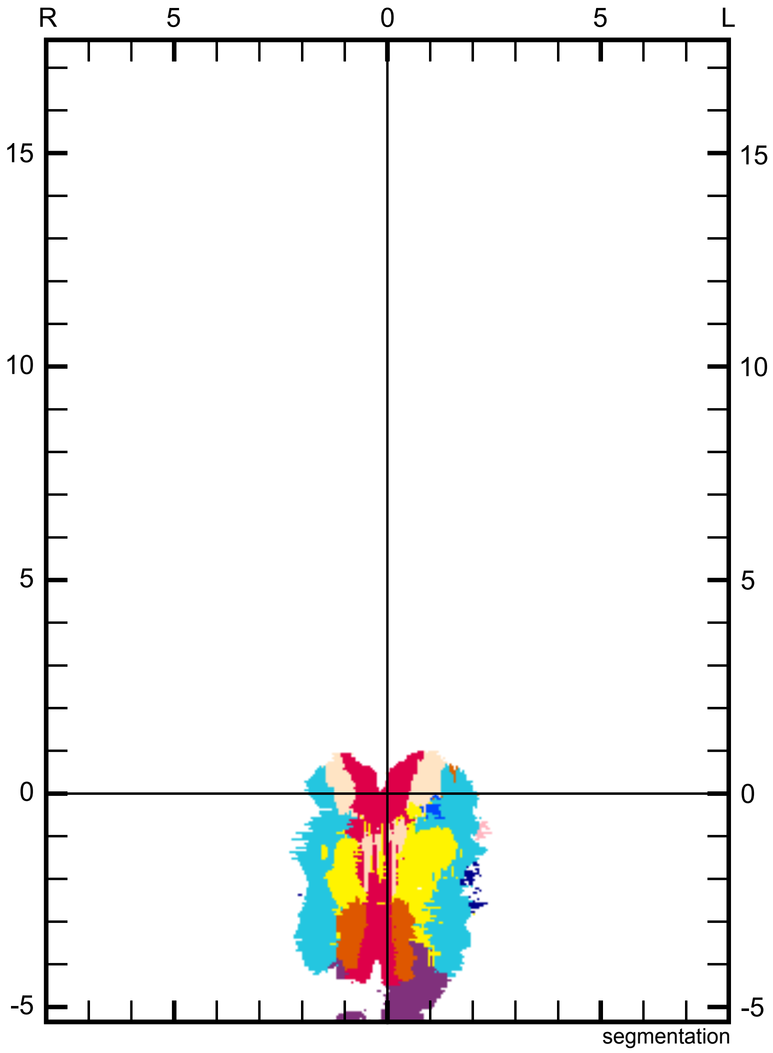

Sagittal cross-section 1
7.0 mm right from midline

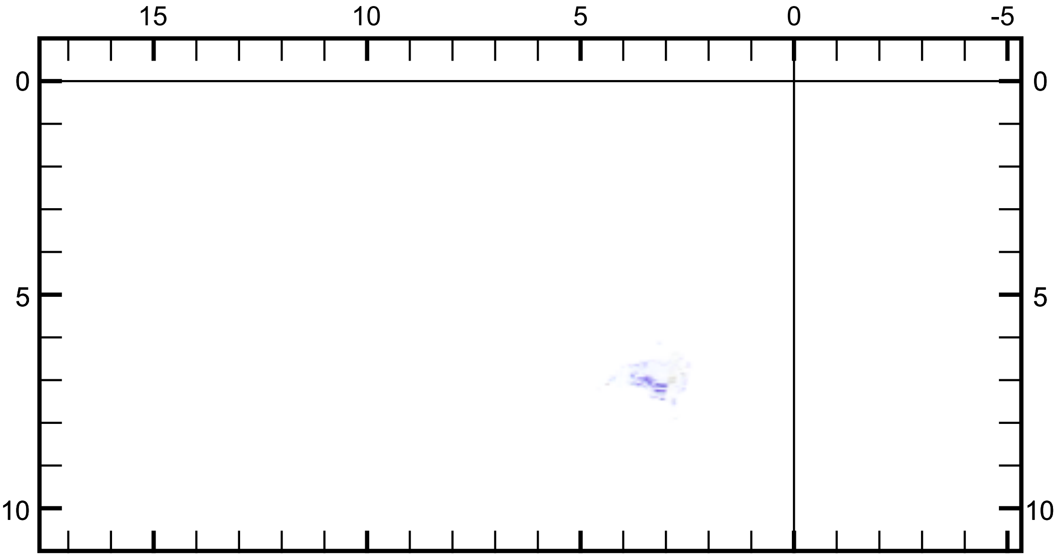

Nissl

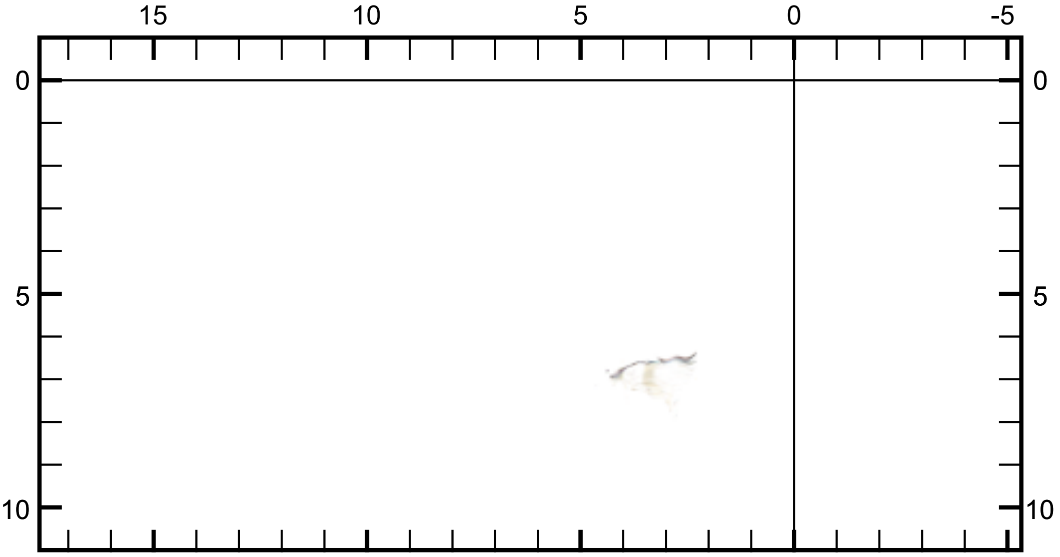

myelin

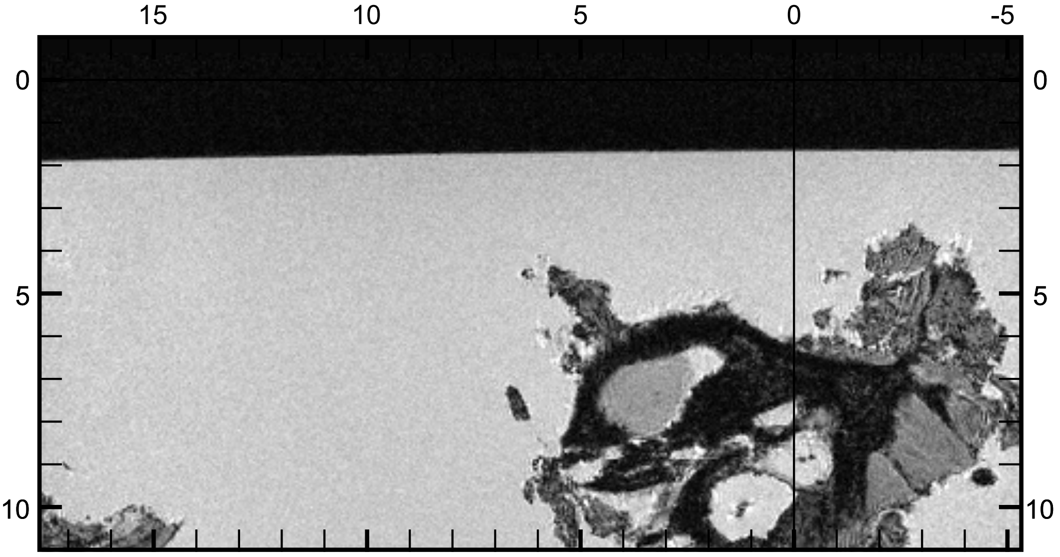

post mortem MR, 30 days

Sagittal cross-section 1 (continued)
7.0 mm right from midline

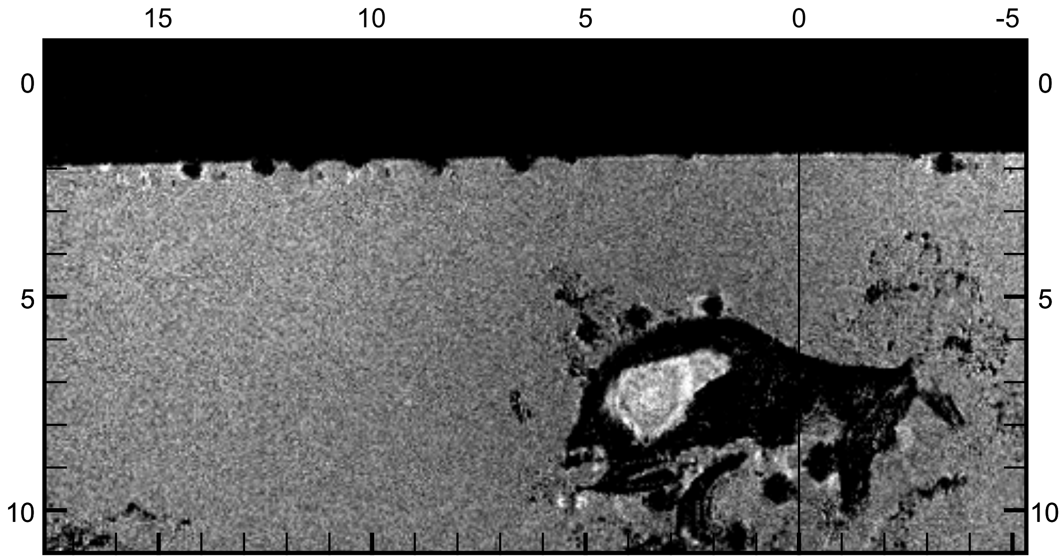

post mortem MR, 48 h

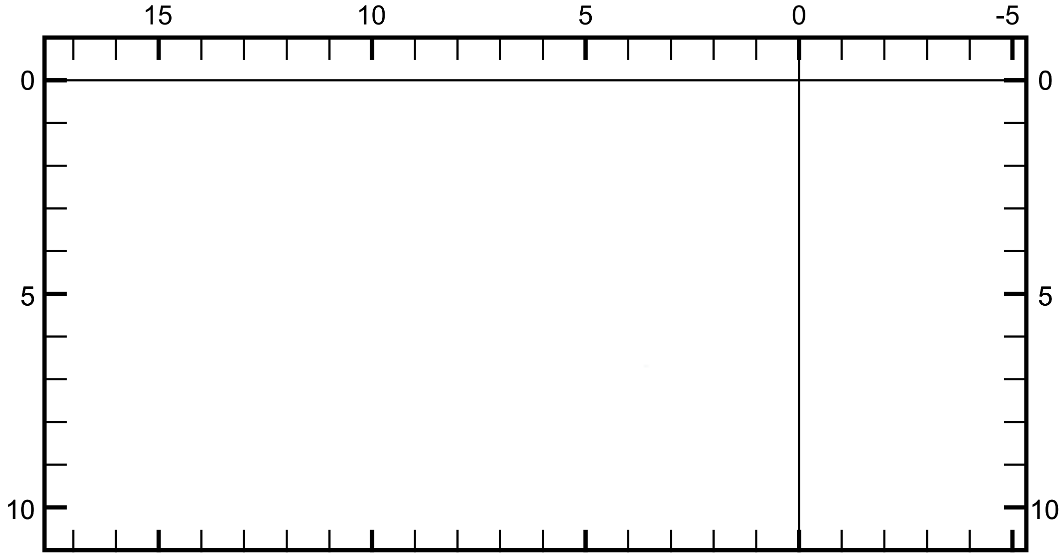

blockface image

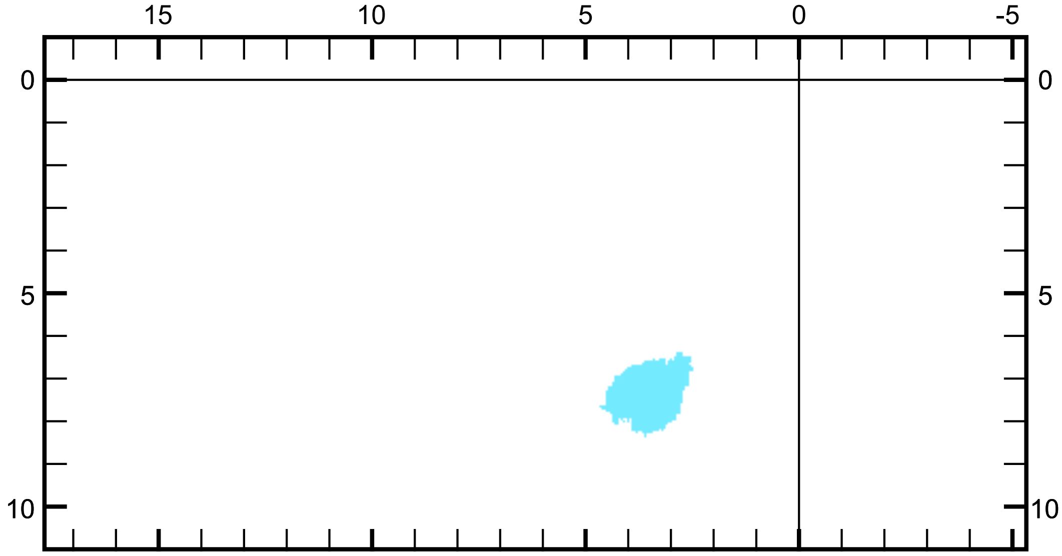

segmentation

Sagittal cross-section 2
6.5 mm right from midline

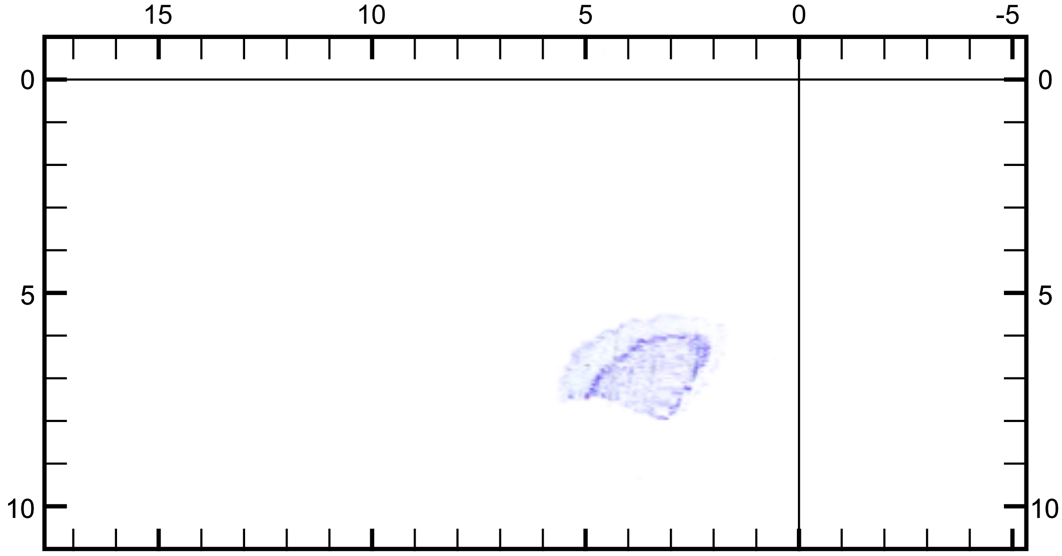

Nissl

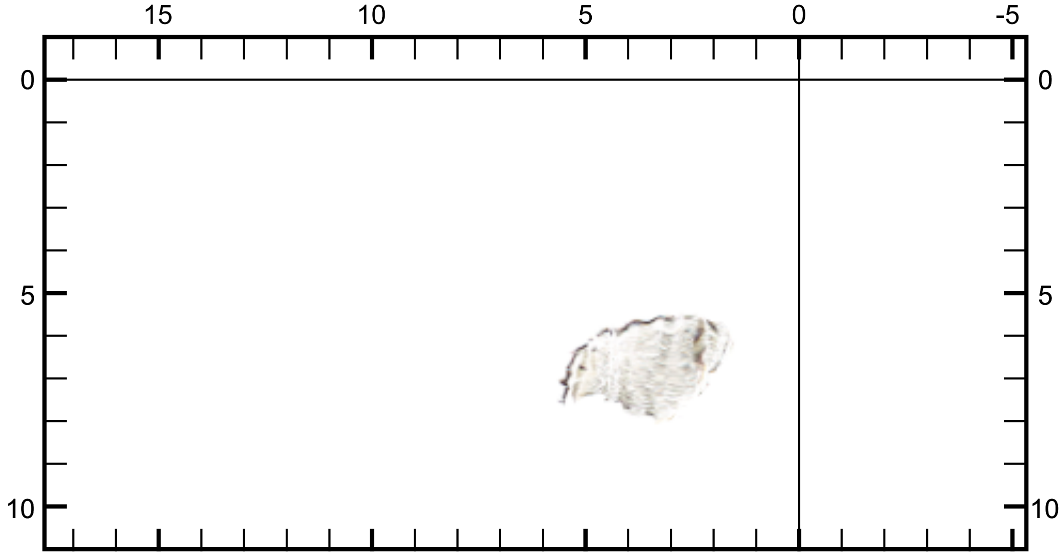

myelin

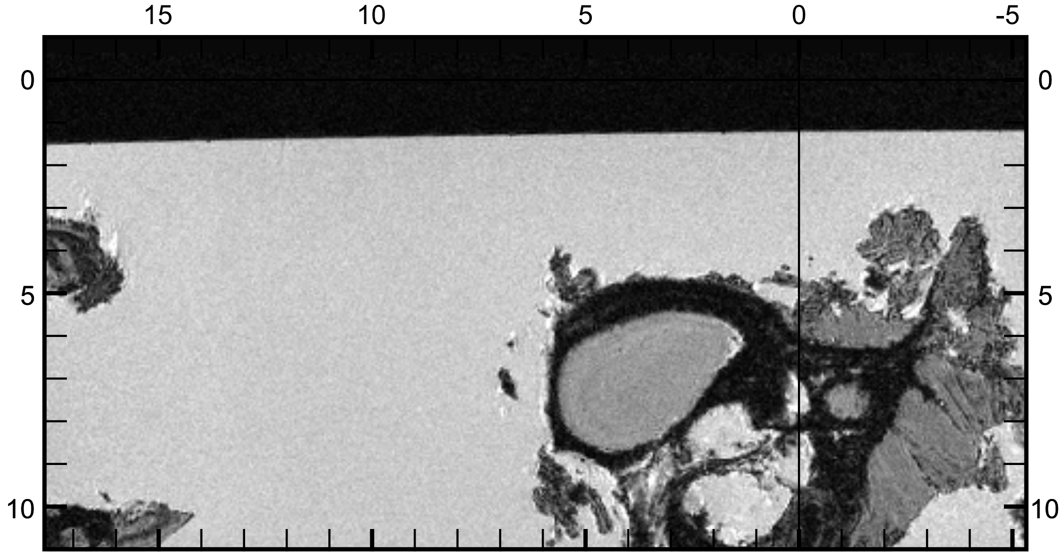

post mortem MR, 30 days

Sagittal cross-section 2 (continued)
6.5 mm right from midline

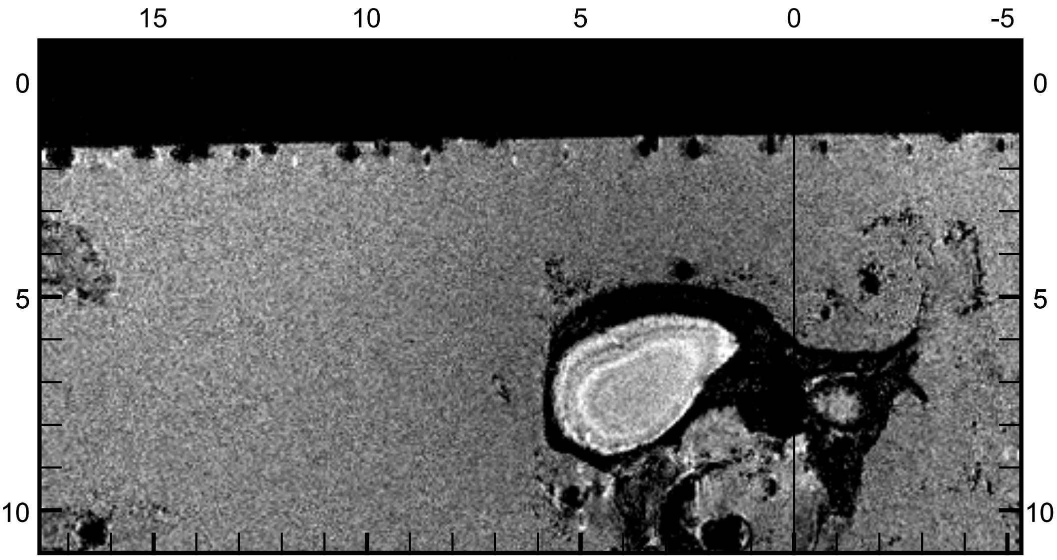

post mortem MR, 48 h

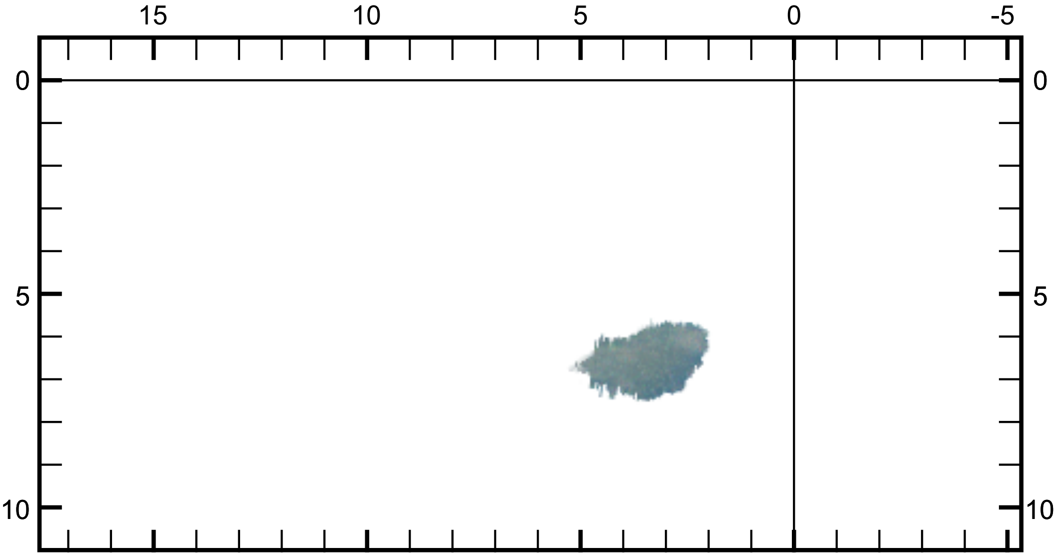

blockface image

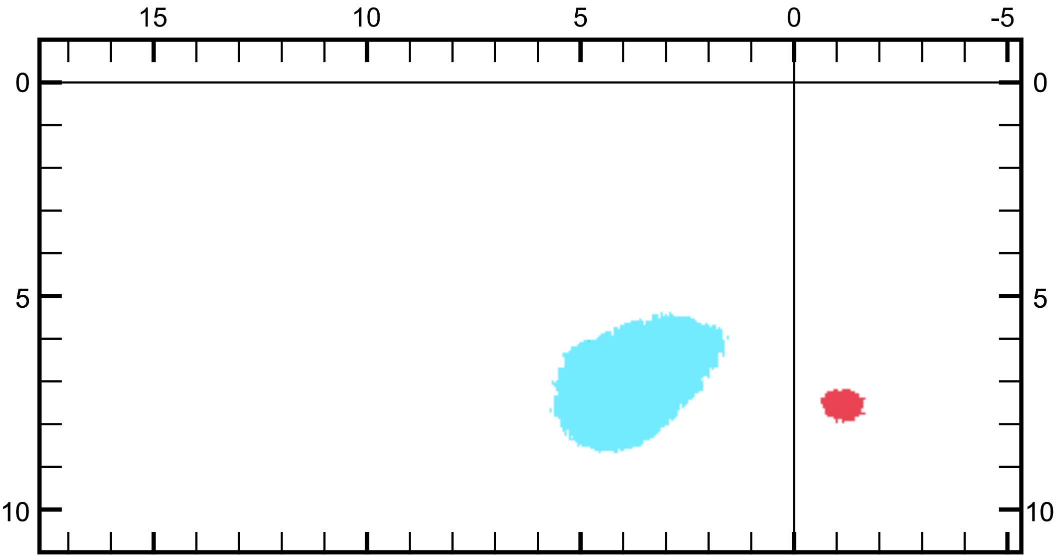

segmentation

Sagittal cross-section 3
6.0 mm right from midline

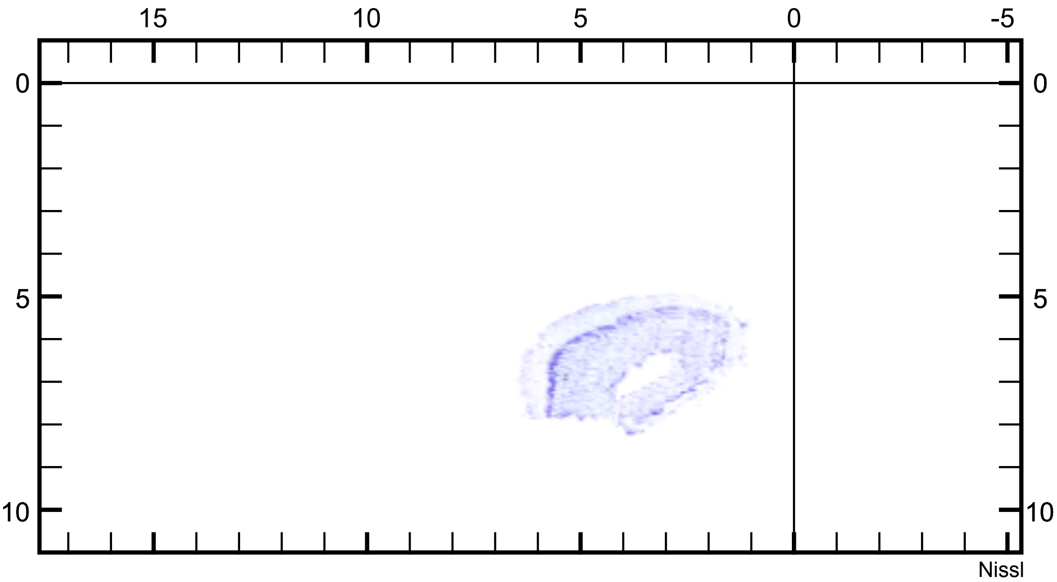

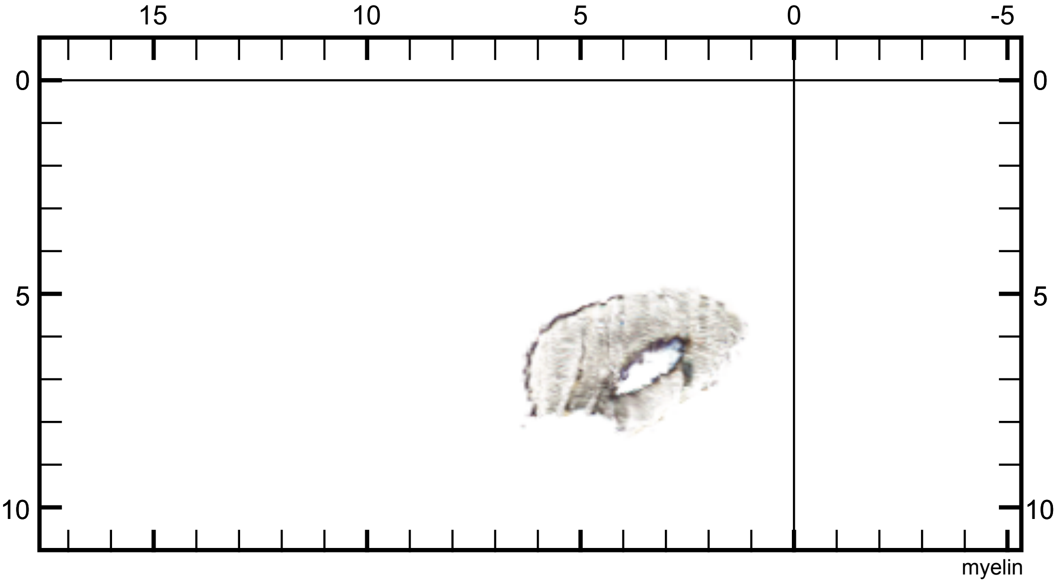

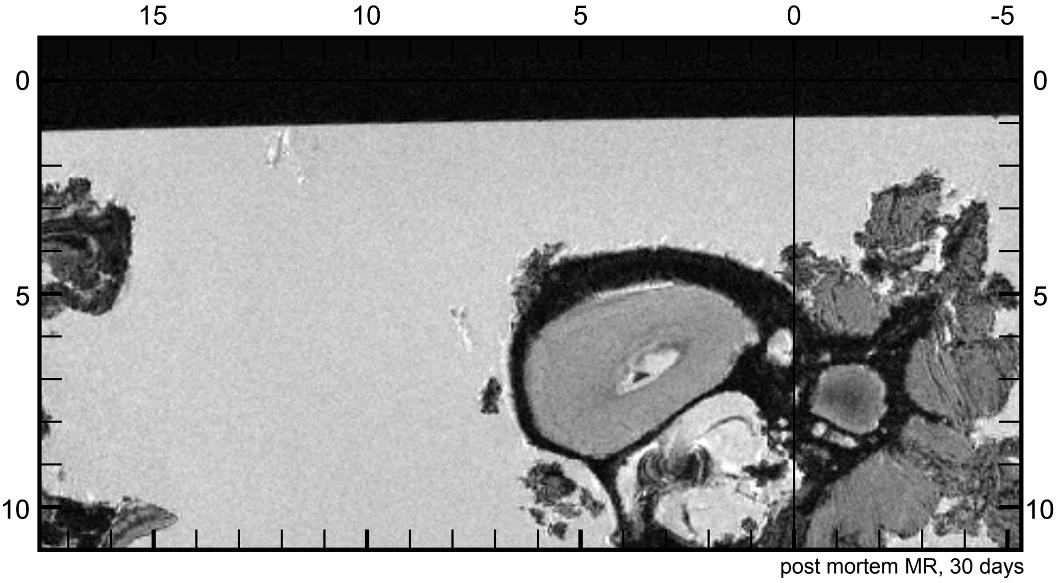

Sagittal cross-section 3 (continued)
6.0 mm right from midline

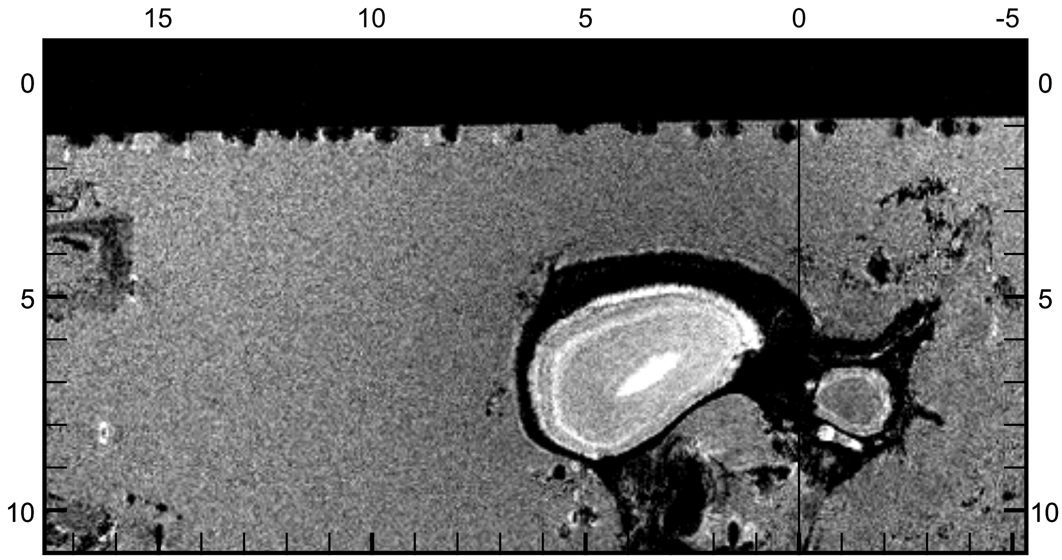

post mortem MR, 48 h

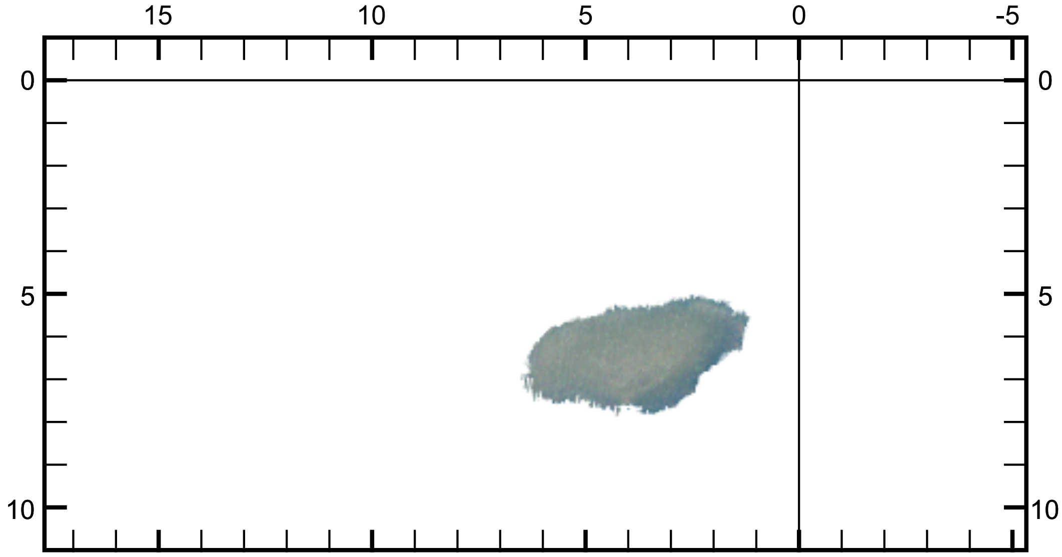

blockface image

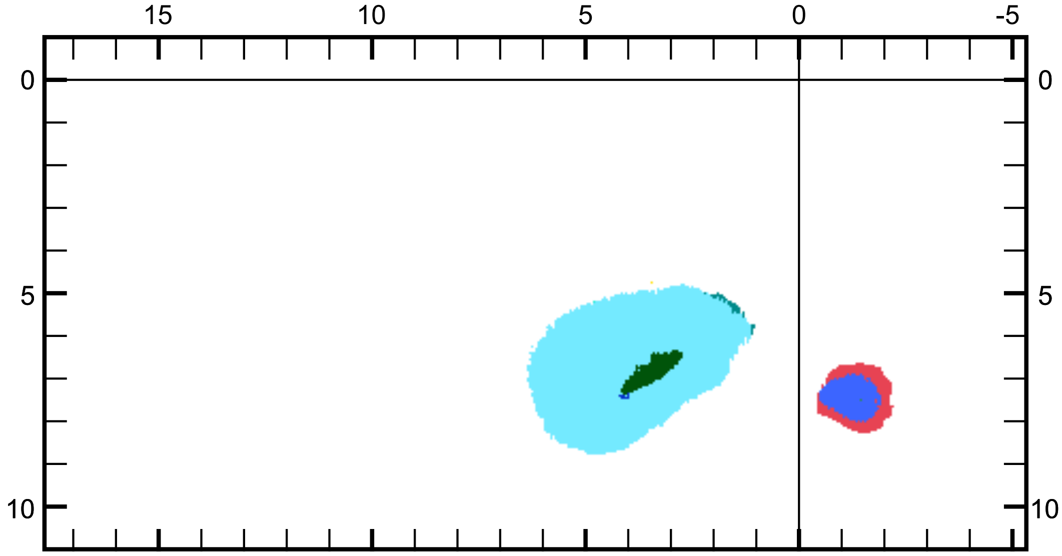

segmentation

Sagittal cross-section 4
5.5 mm right from midline

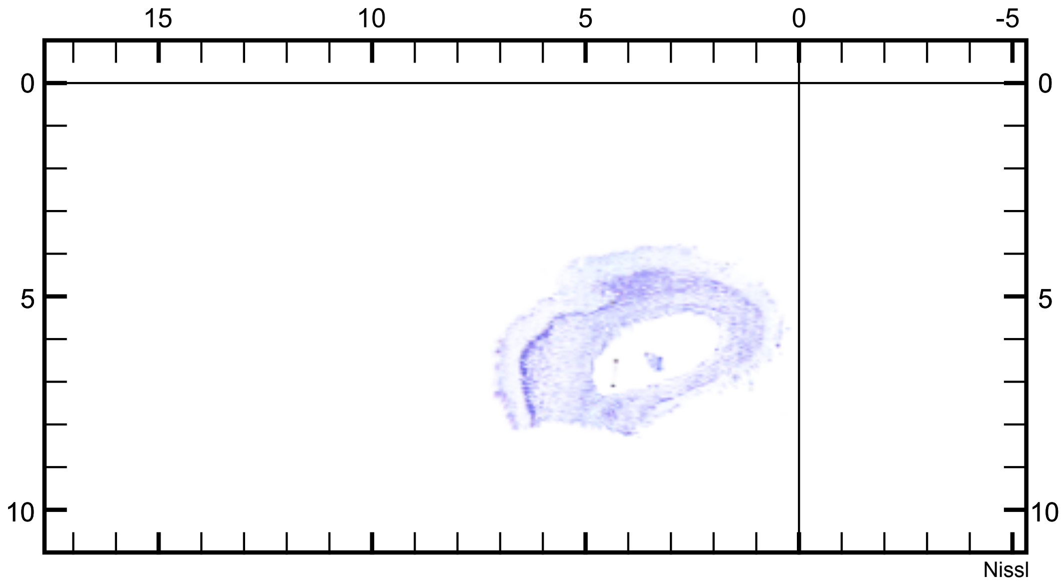

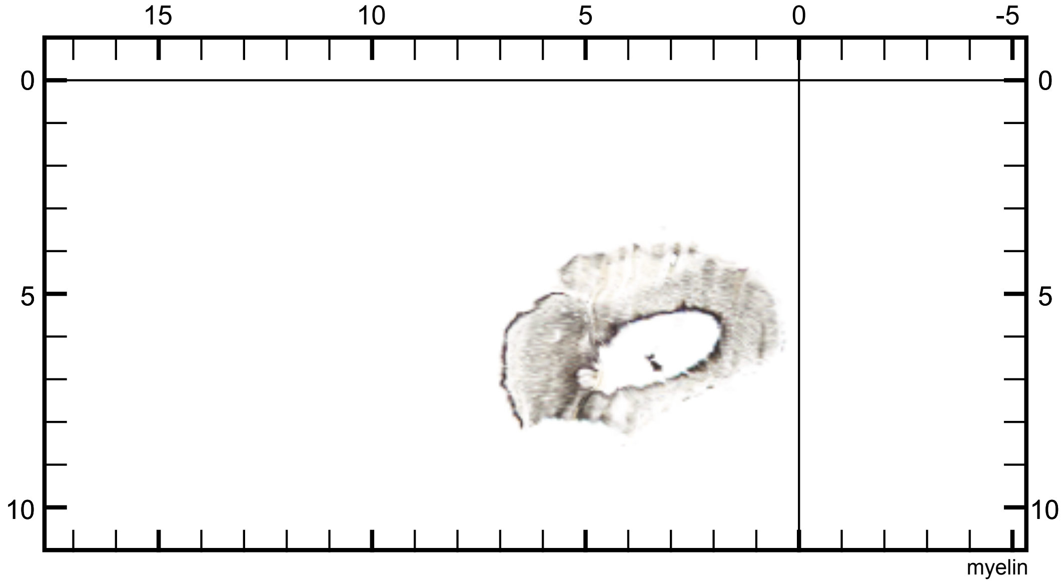

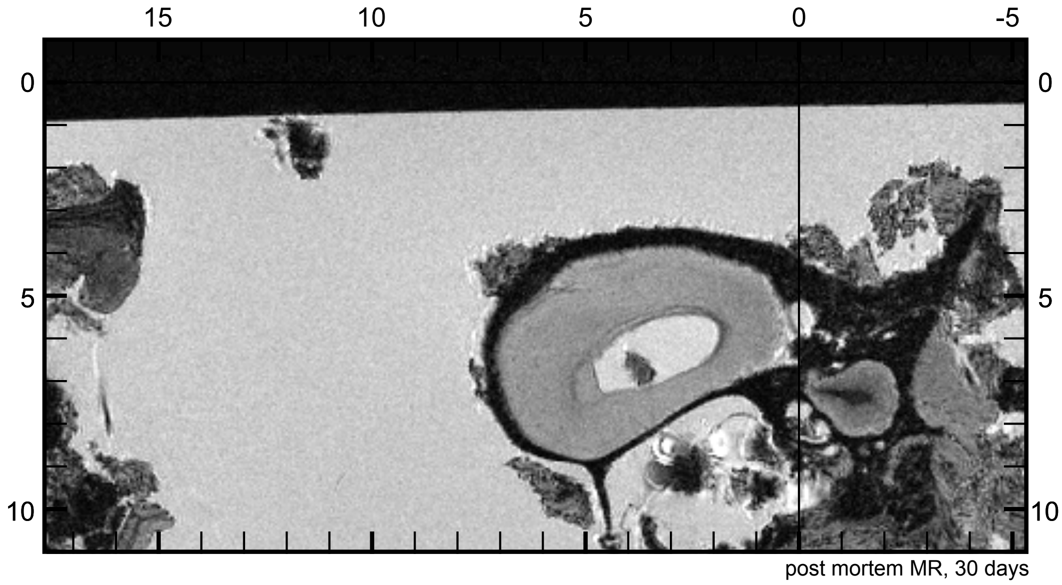

Sagittal cross-section 4 (continued)
5.5 mm right from midline

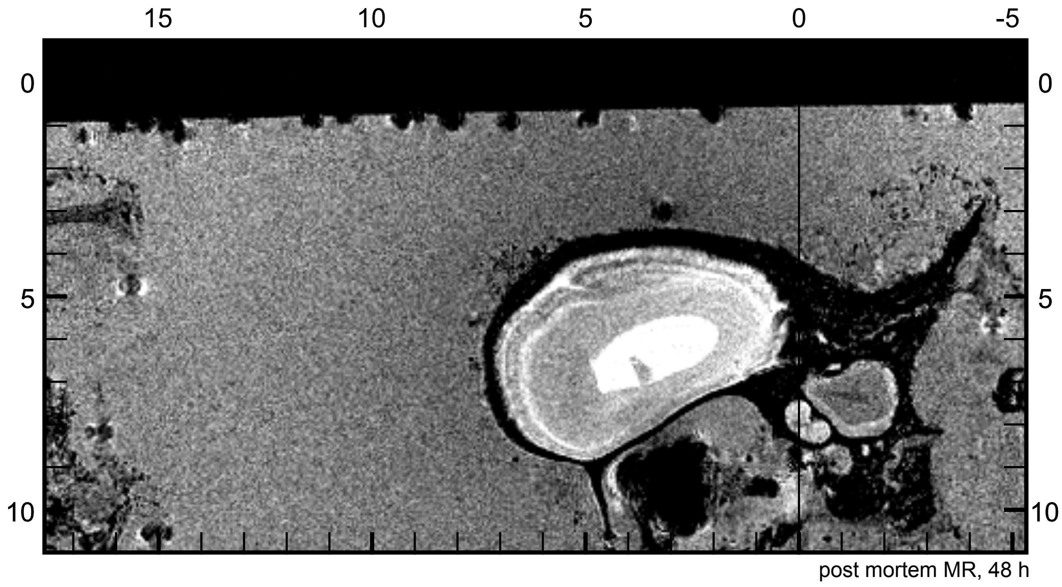

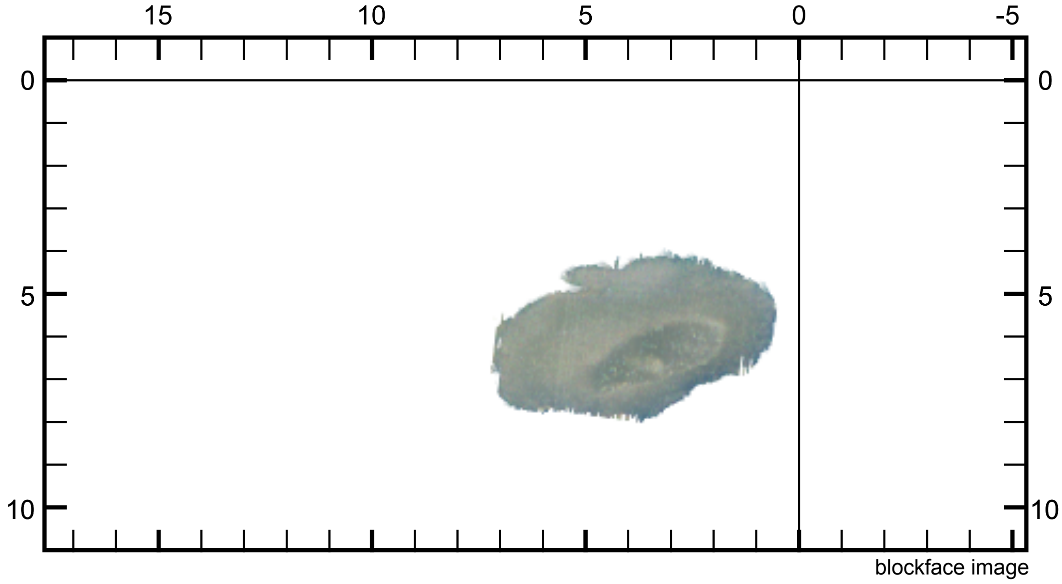

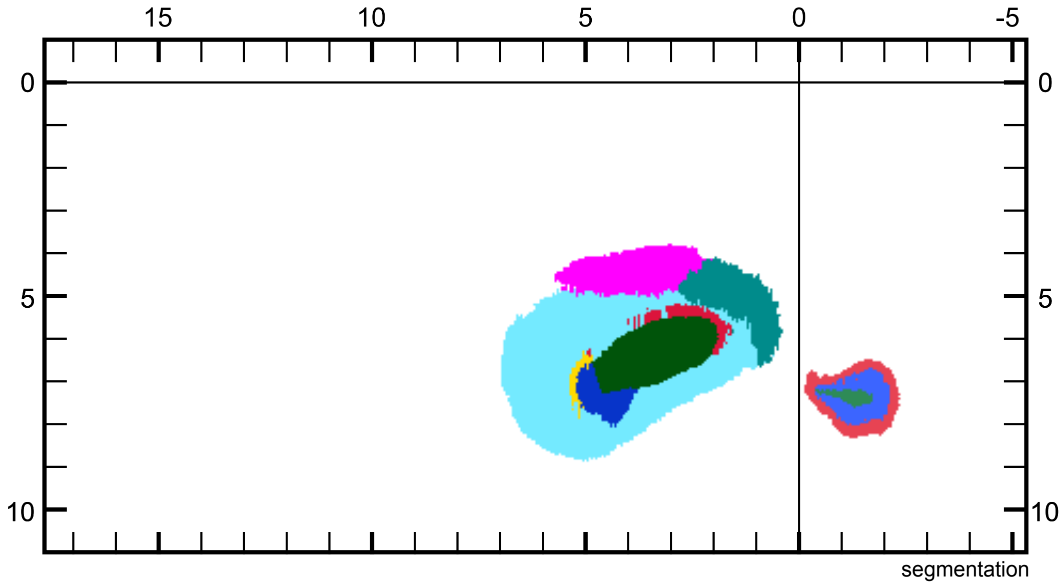

Sagittal cross-section 5
5.0 mm right from midline

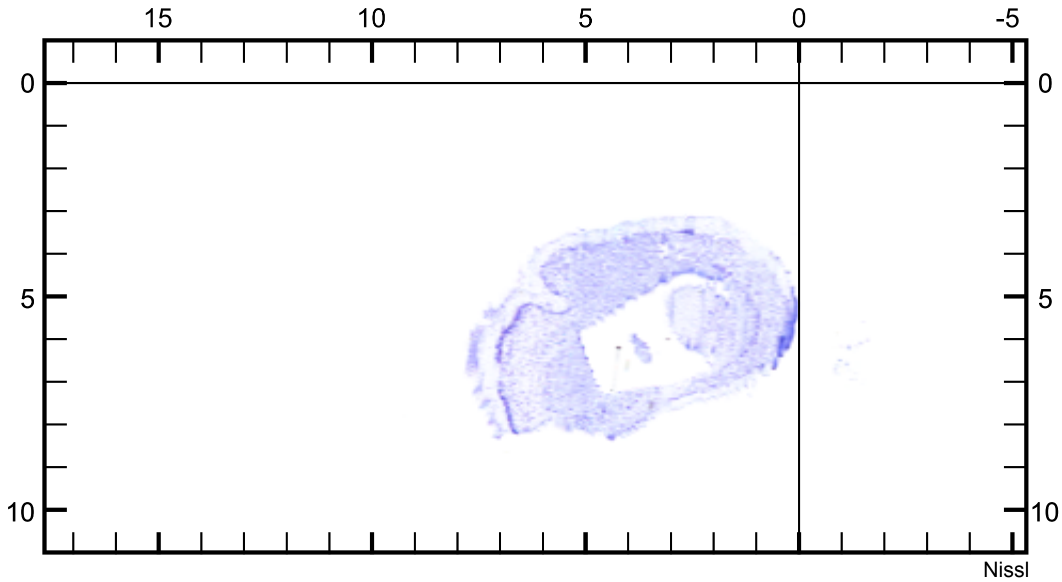

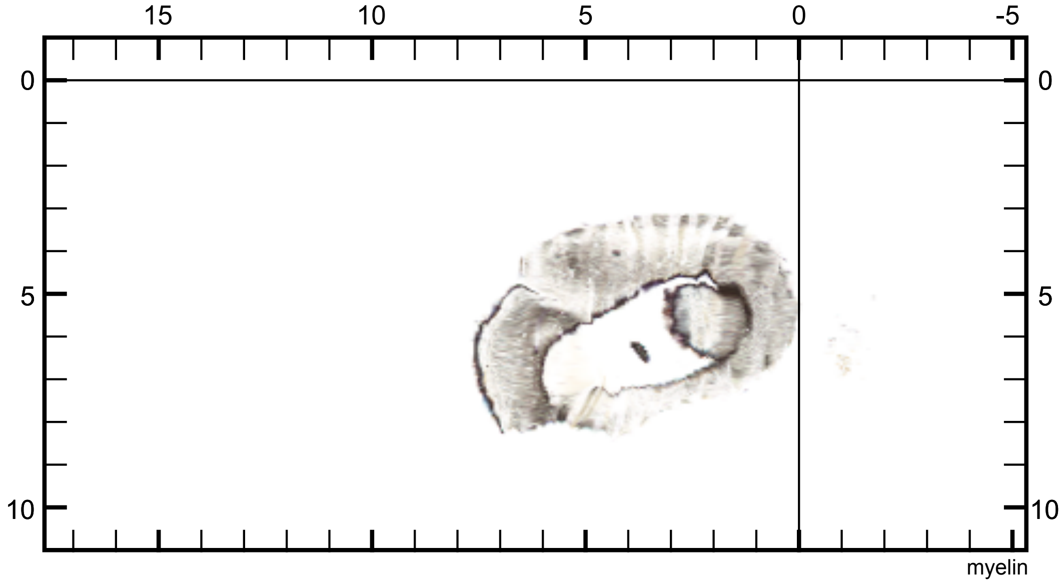

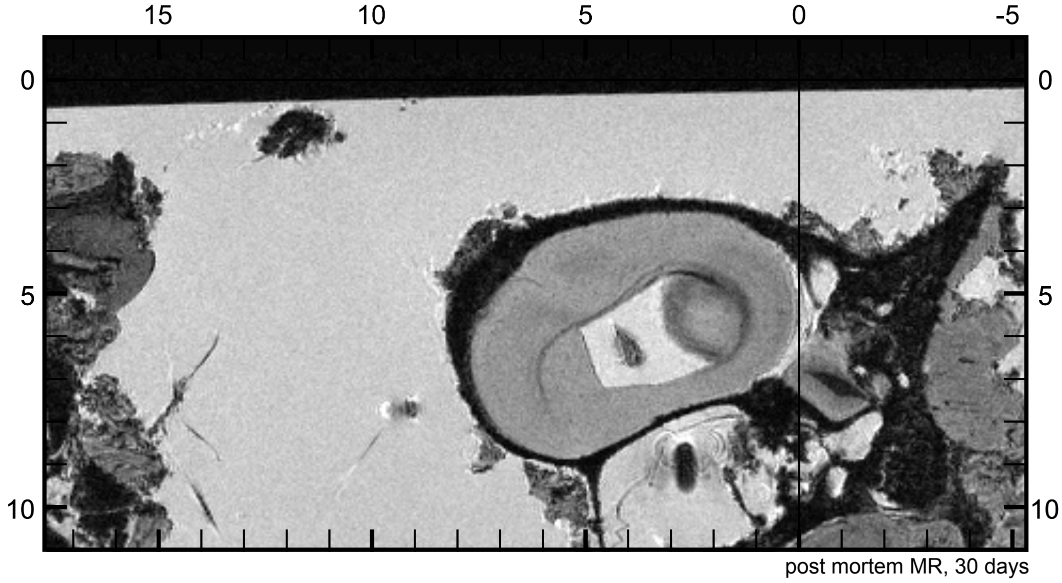

Sagittal cross-section 5 (continued)
5.0 mm right from midline

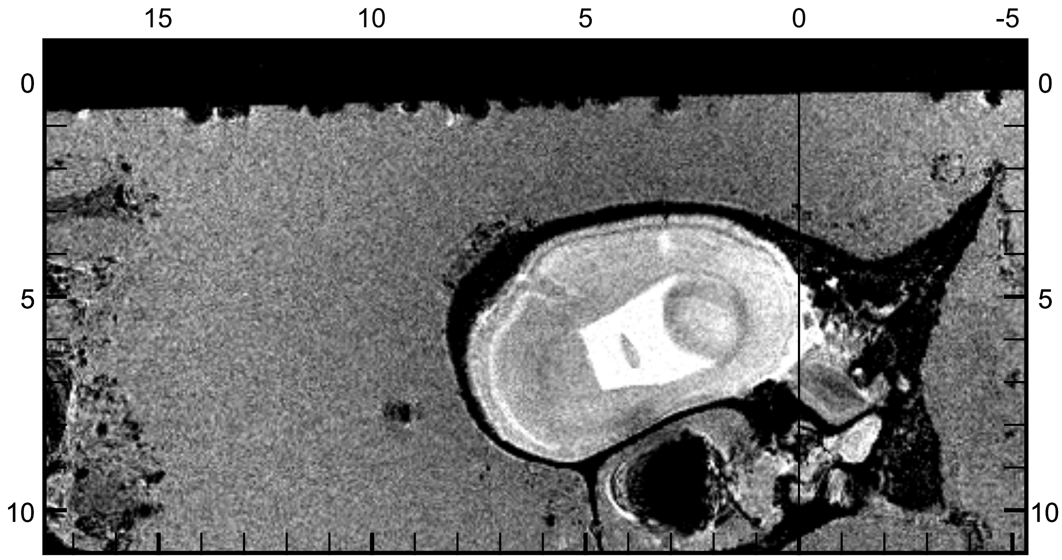

post mortem MR, 48 h

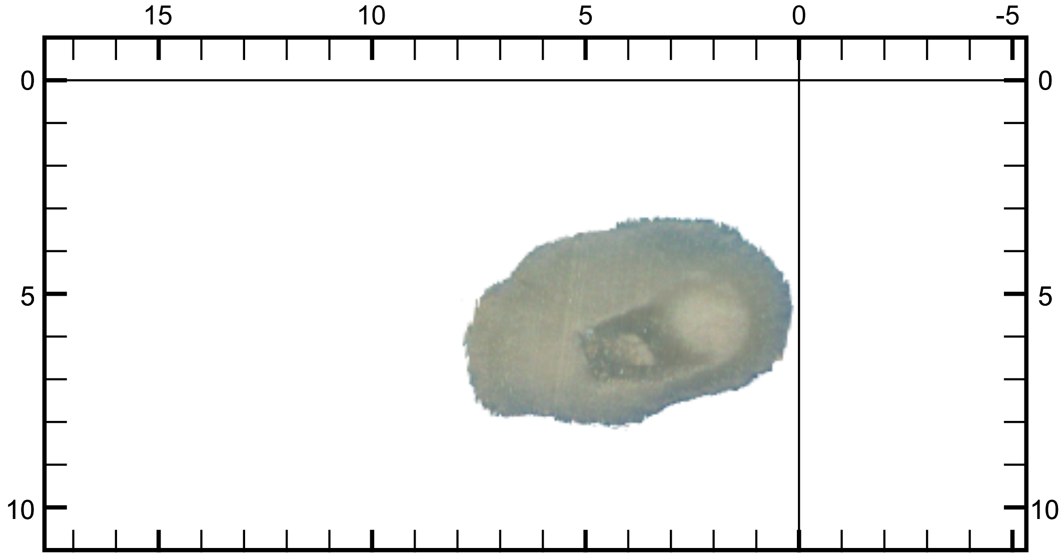

blockface image

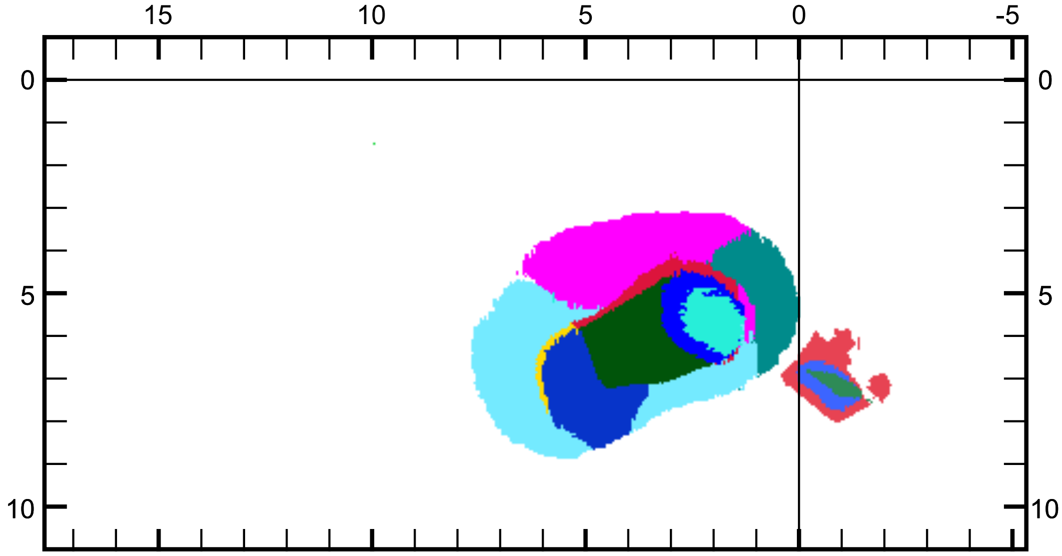

segmentation

Sagittal cross-section 6
4.5 mm right from midline

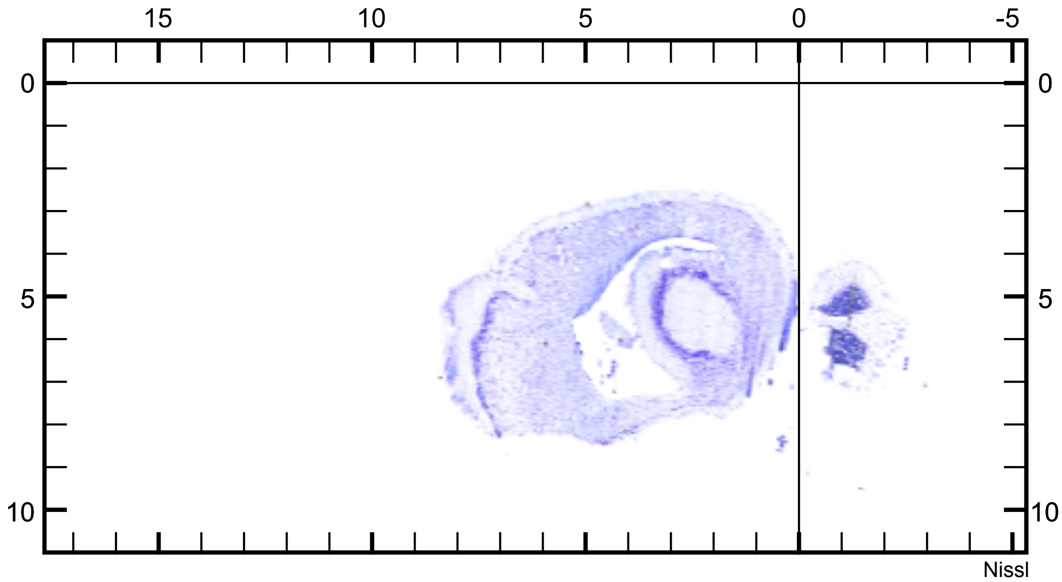

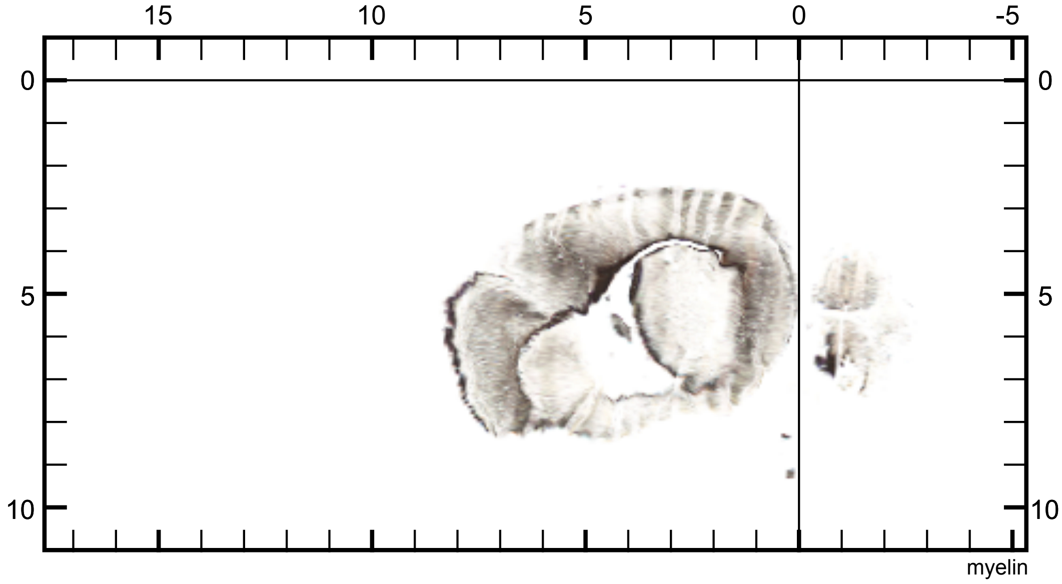

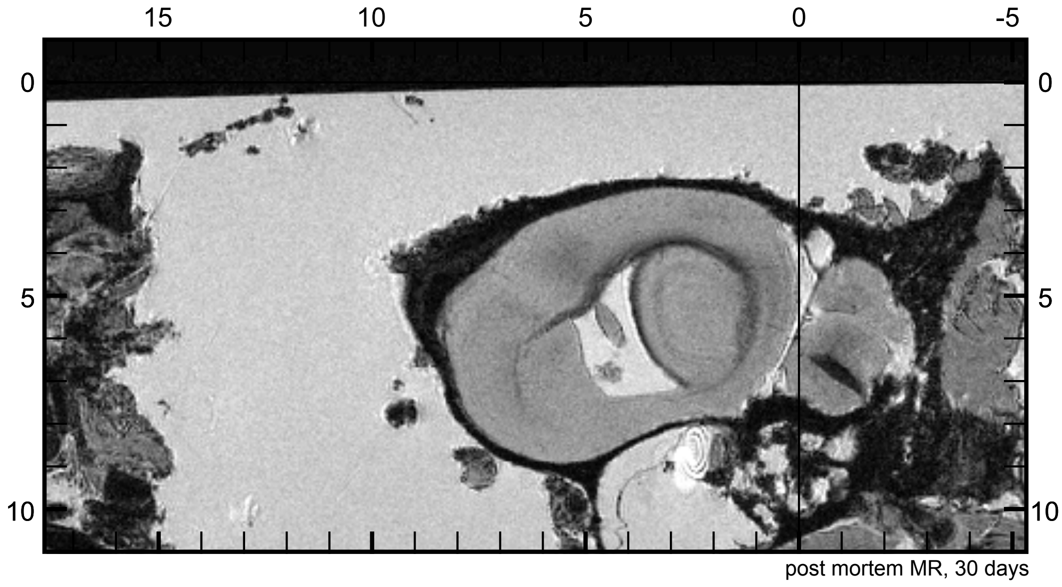

Sagittal cross-section 6 (continued)
4.5 mm right from midline

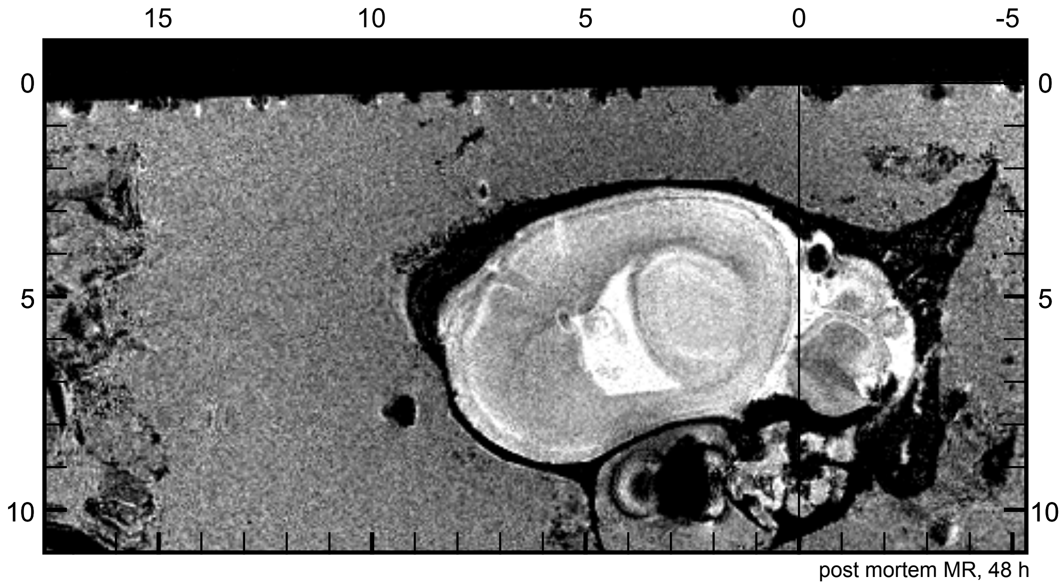

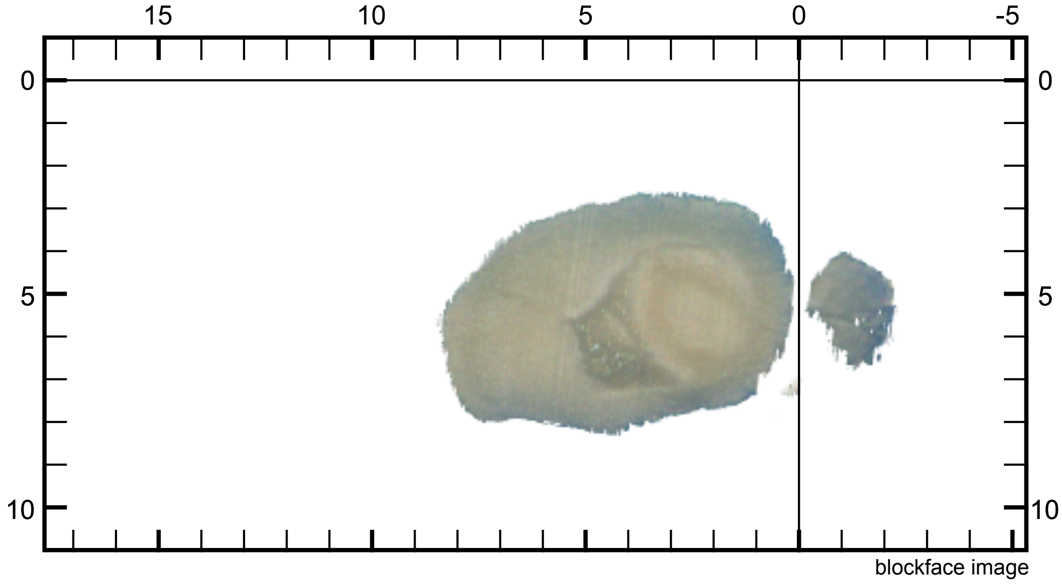

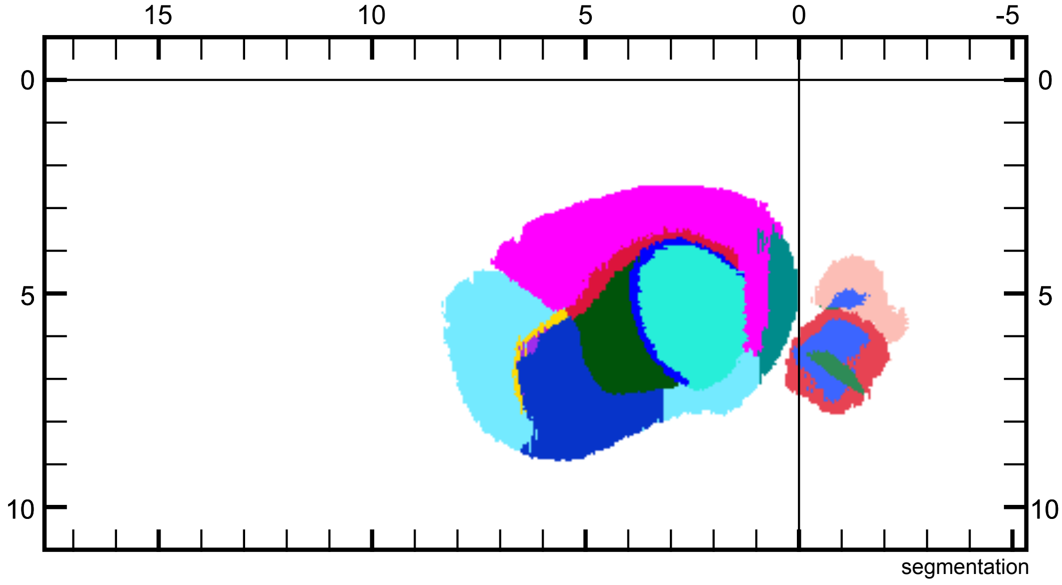

Sagittal cross-section 7
4.0 mm right from midline

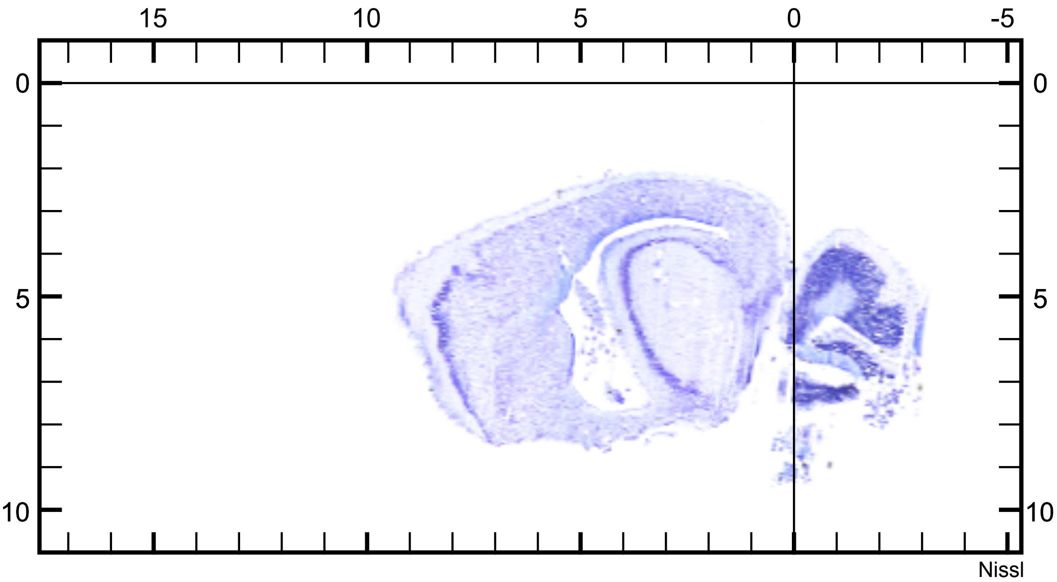

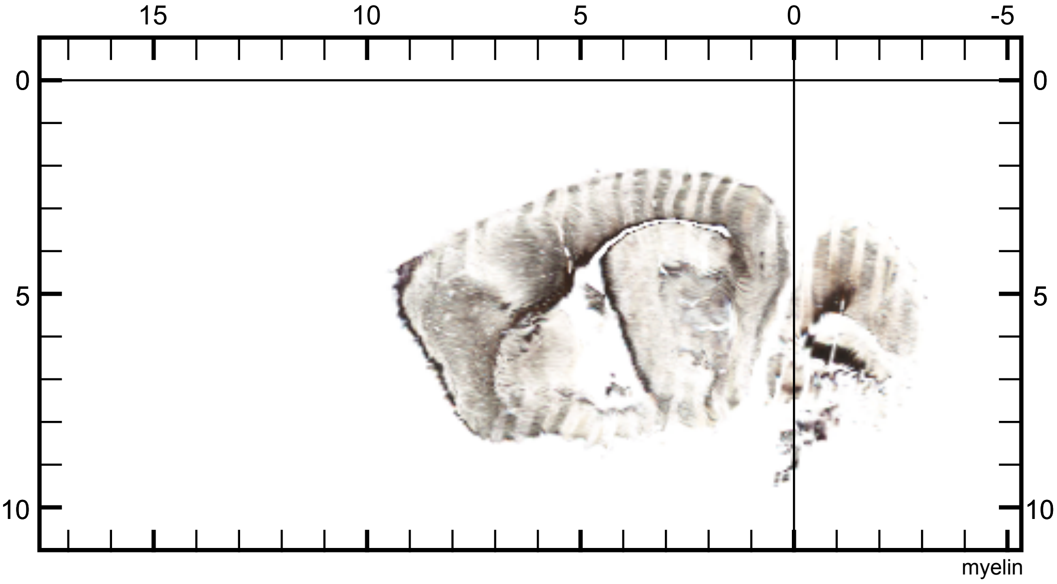

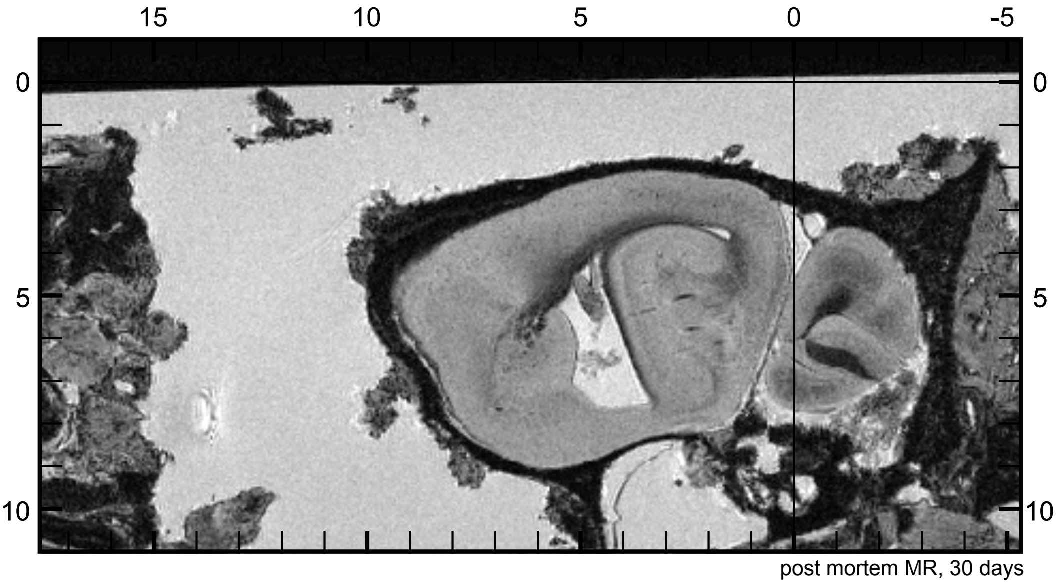

Sagittal cross-section 7 (continued)
4.0 mm right from midline

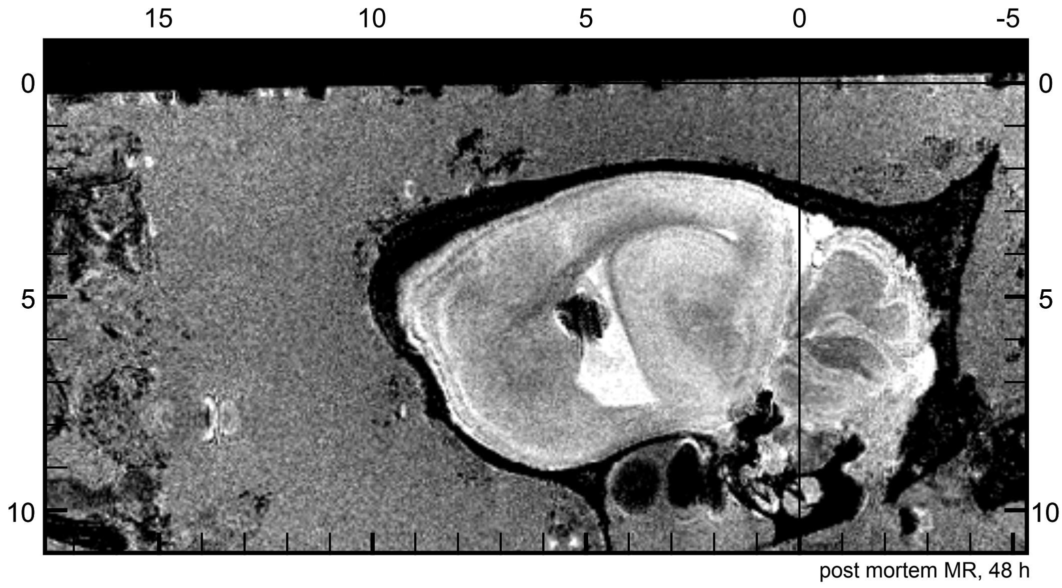

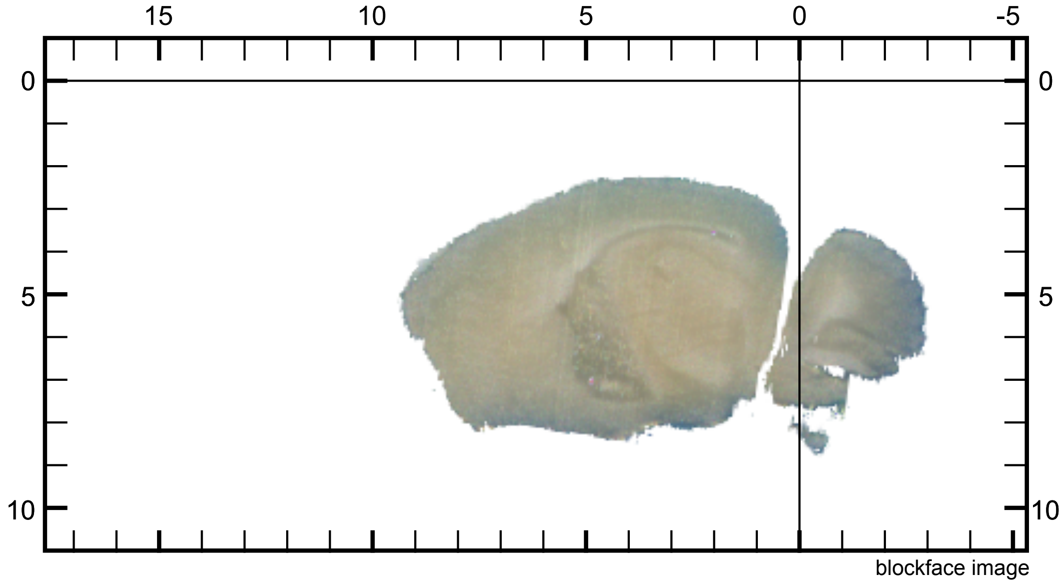

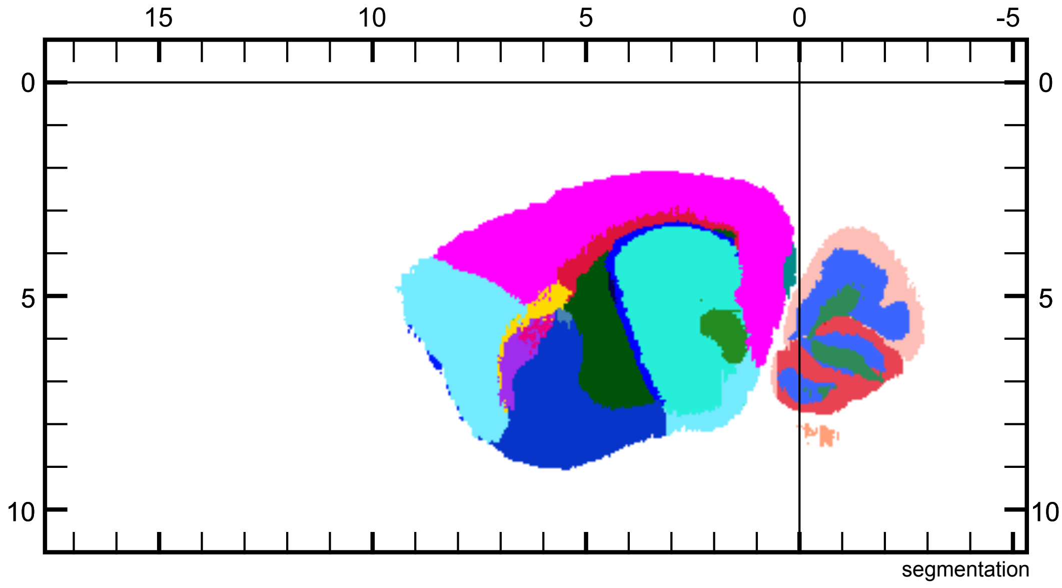

Sagittal cross-section 8
3.5 mm right from midline

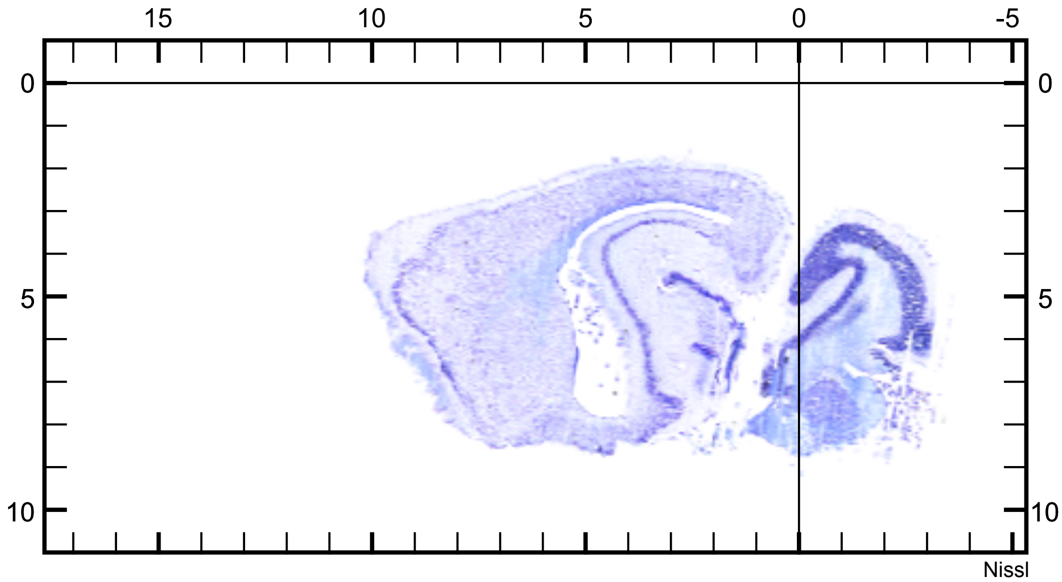

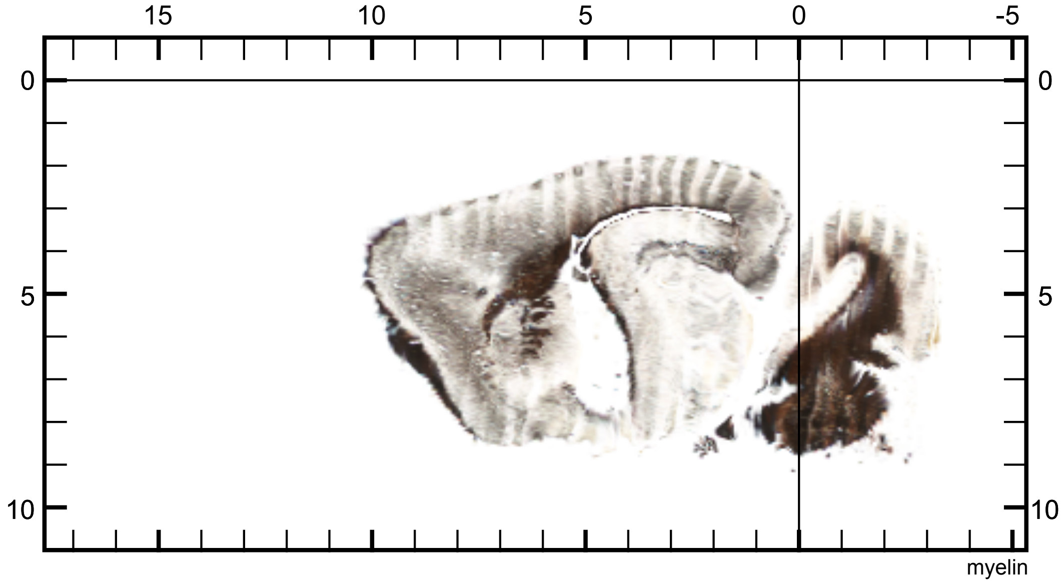

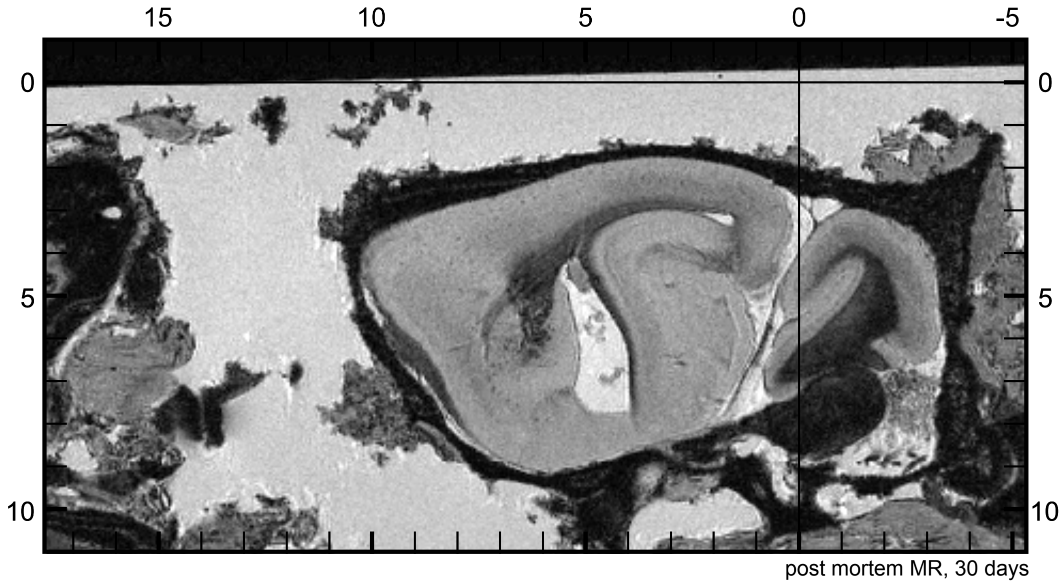

Sagittal cross-section 8 (continued)
3.5 mm right from midline

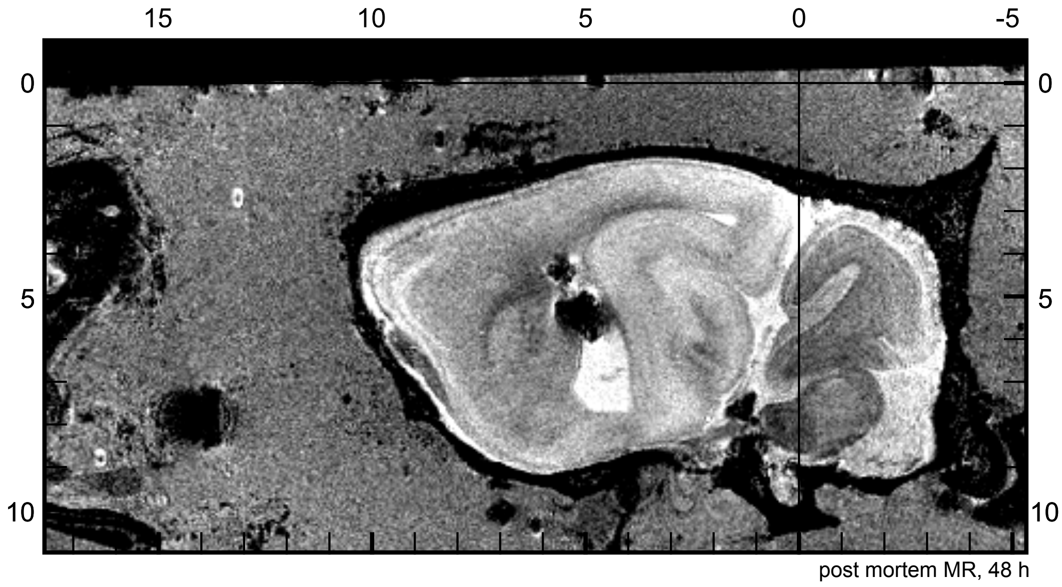

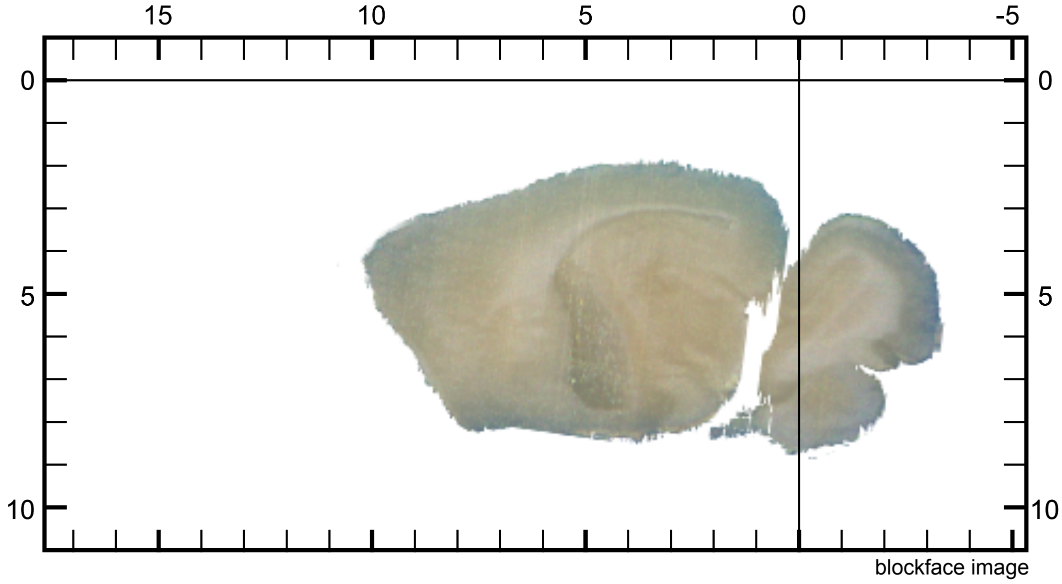

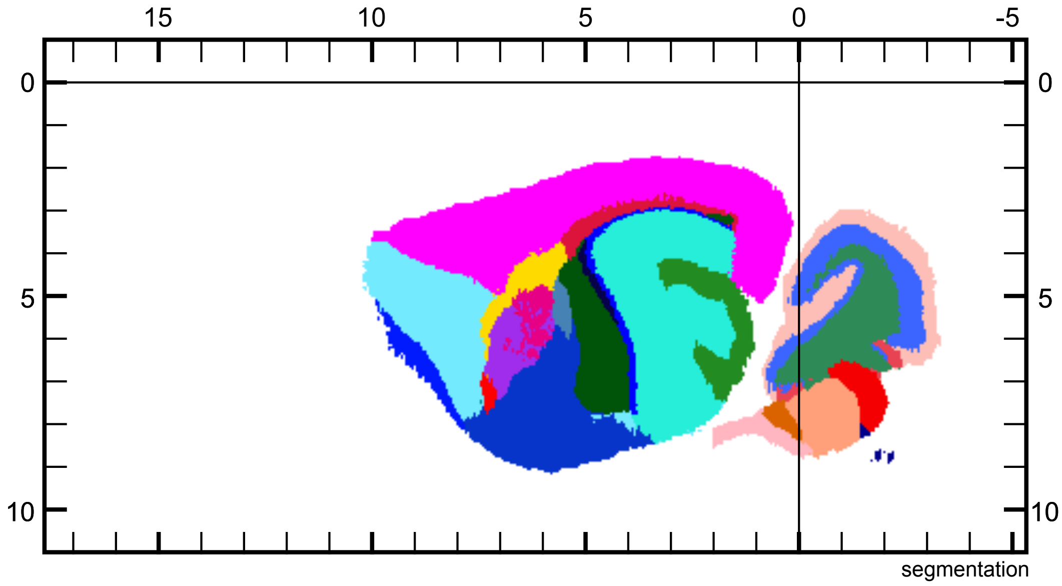

Sagittal cross-section 9
3.0 mm right from midline

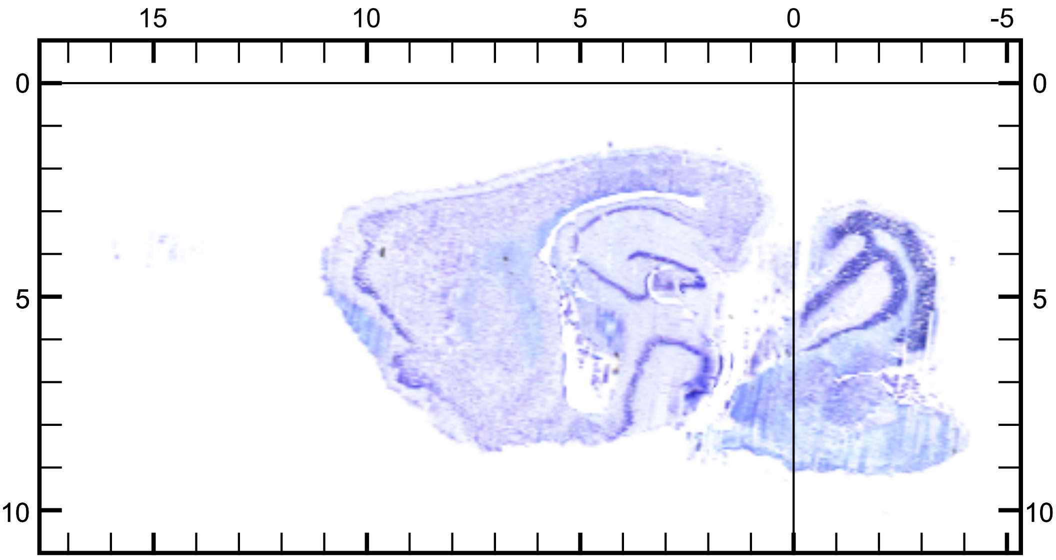

Nissl

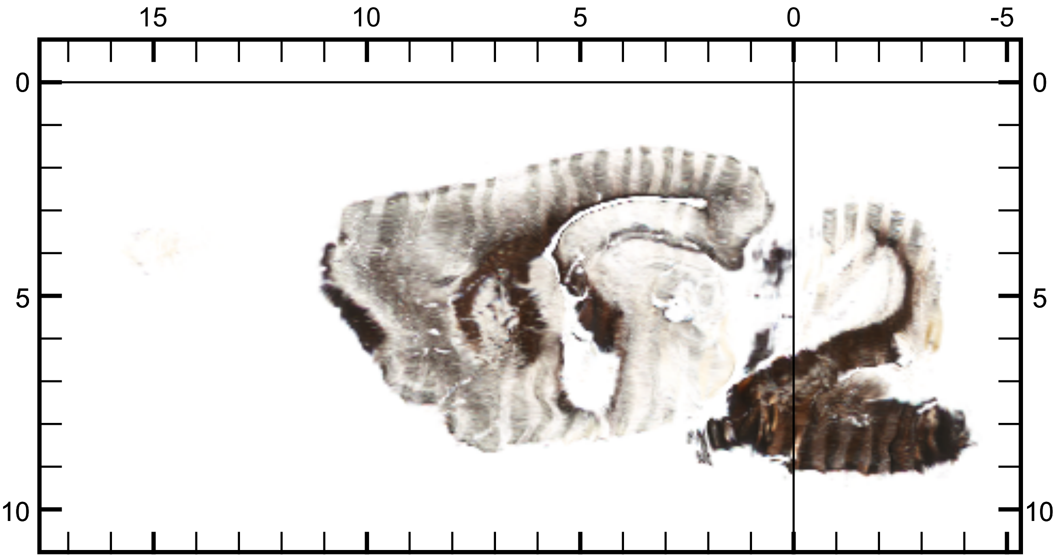

myelin

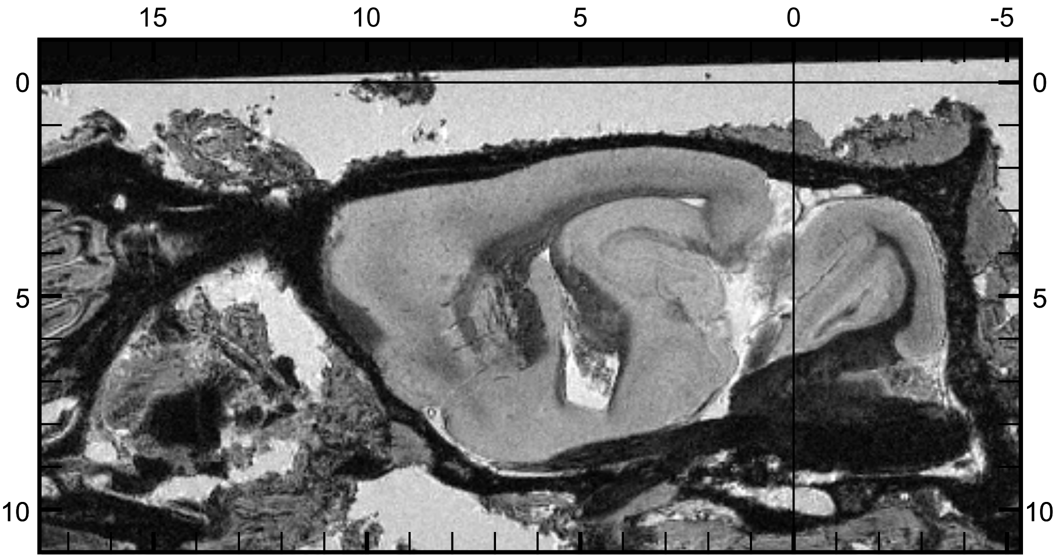

post mortem MR, 30 days

Sagittal cross-section 9 (continued)
3.0 mm right from midline

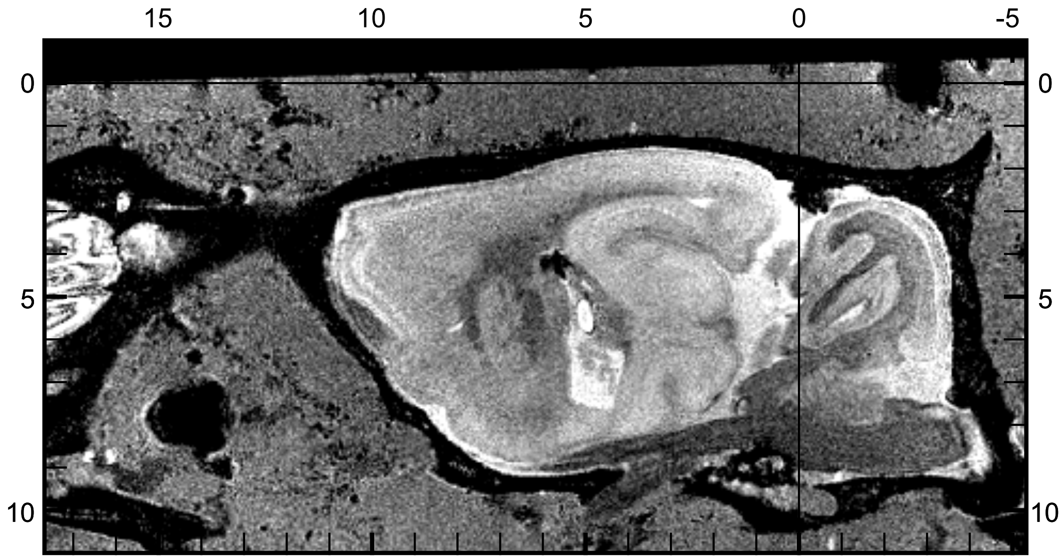

post mortem MR, 48 h

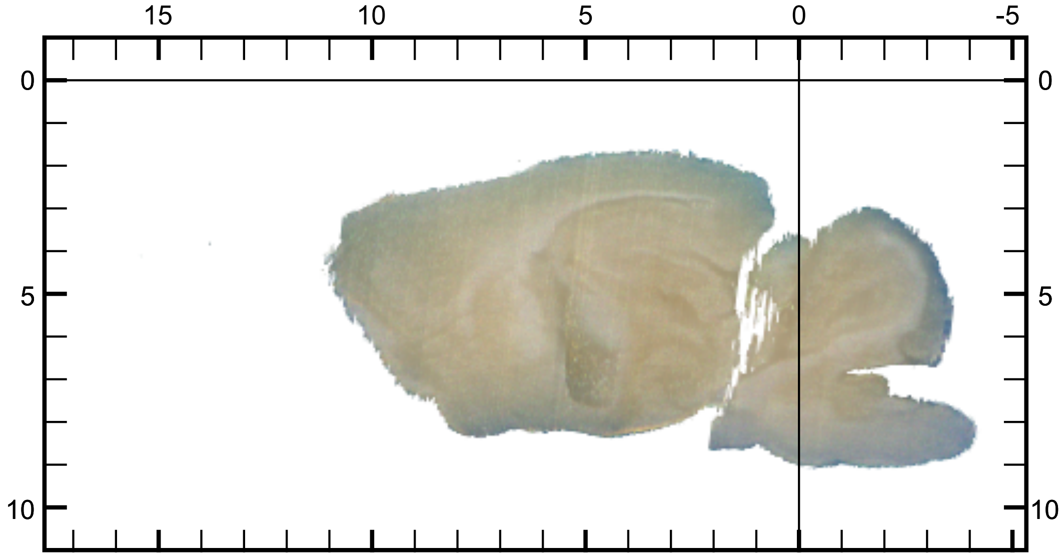

blockface image

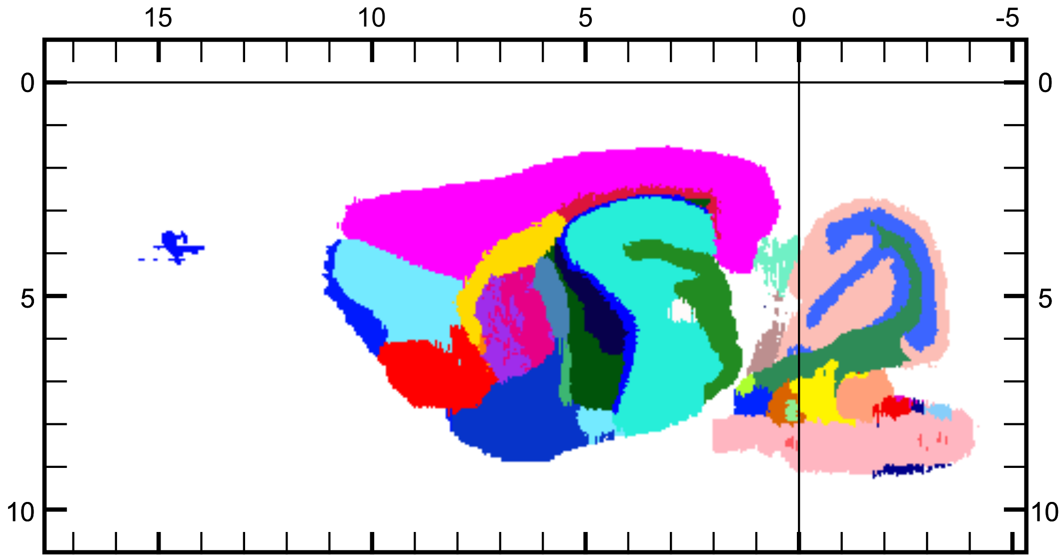

segmentation

Sagittal cross-section 10
2.5 mm right from midline

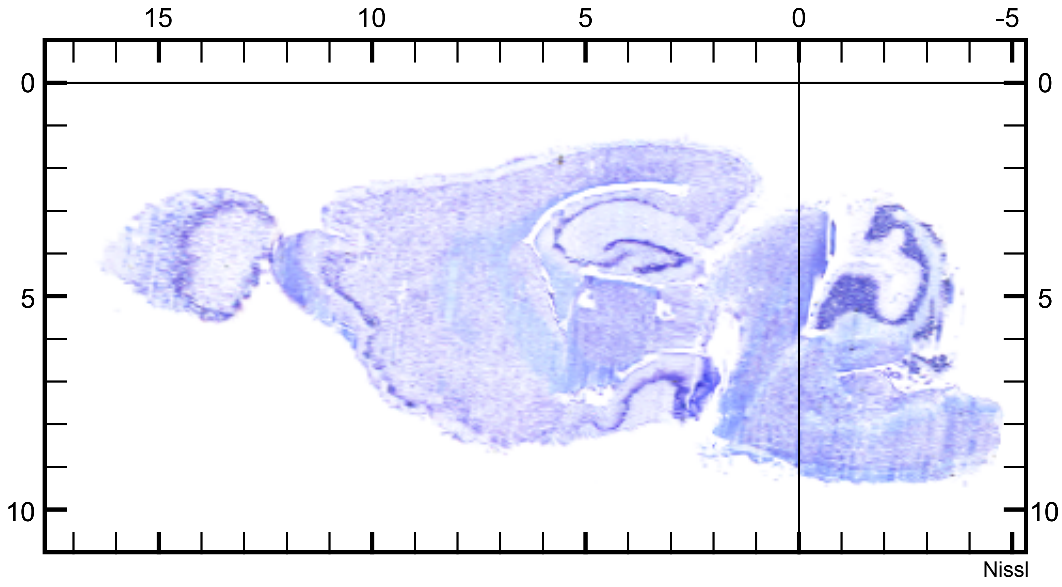

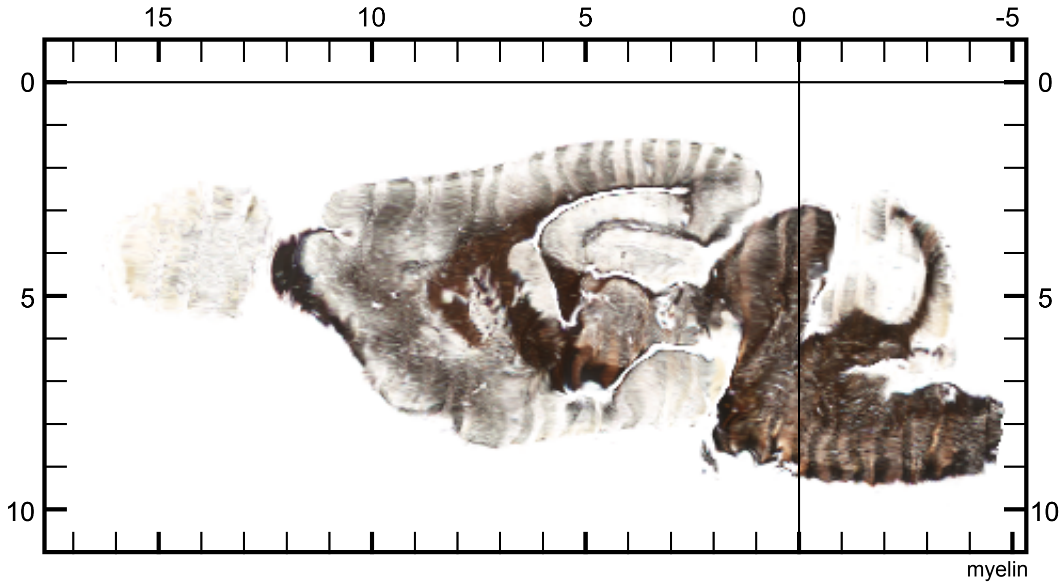

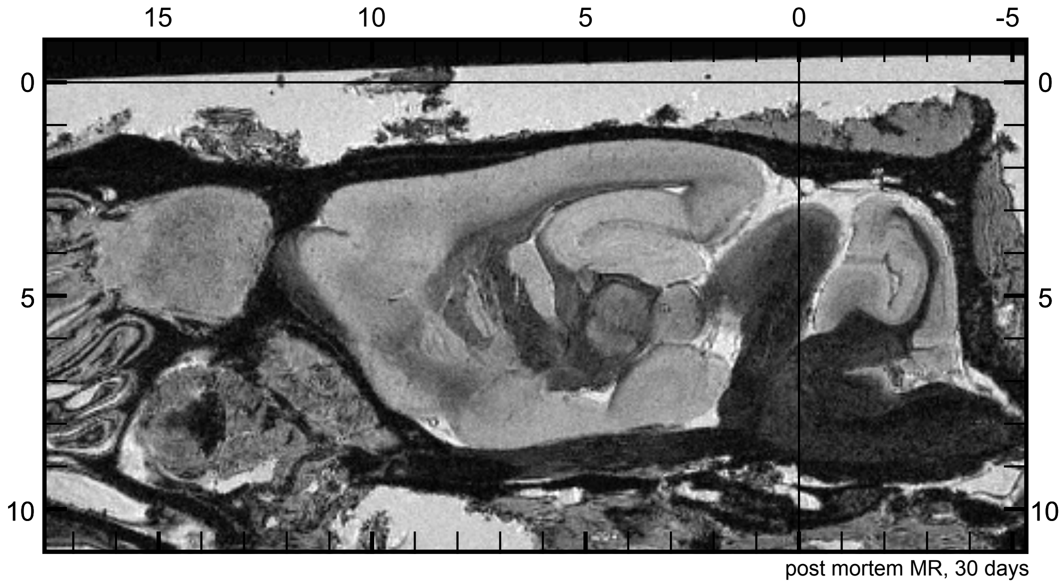

Sagittal cross-section 10 (continued)
2.5 mm right from midline

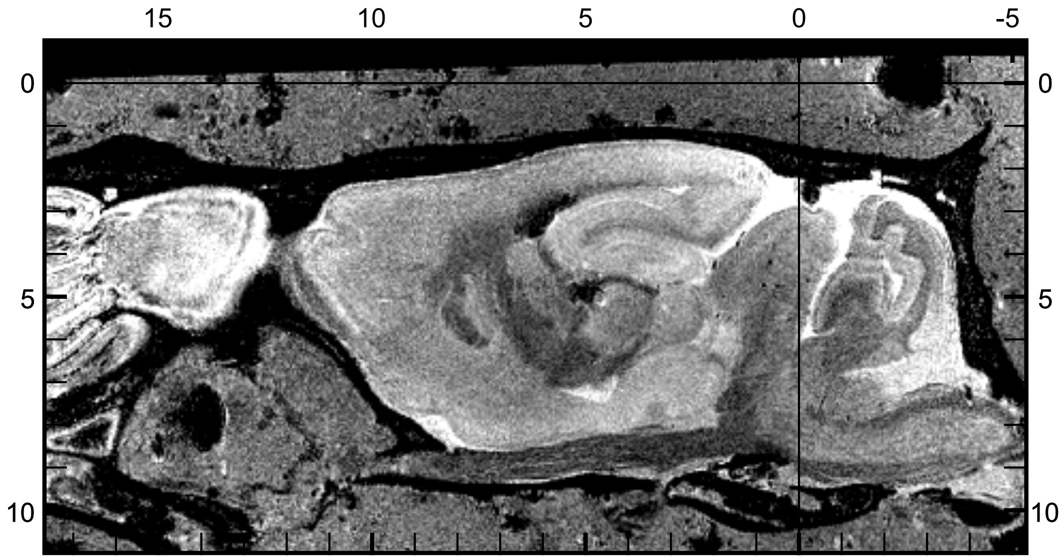

post mortem MR, 48 h

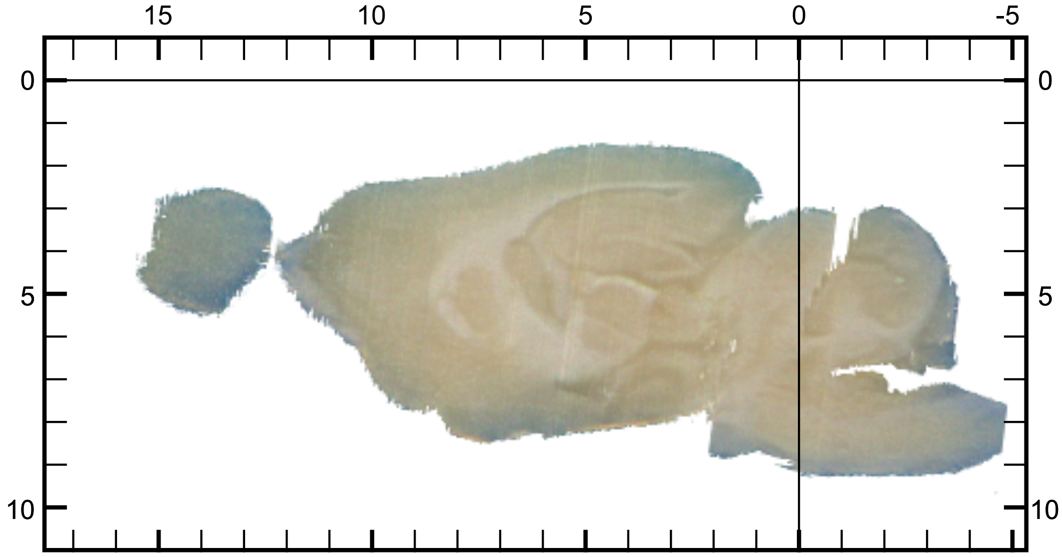

blockface image

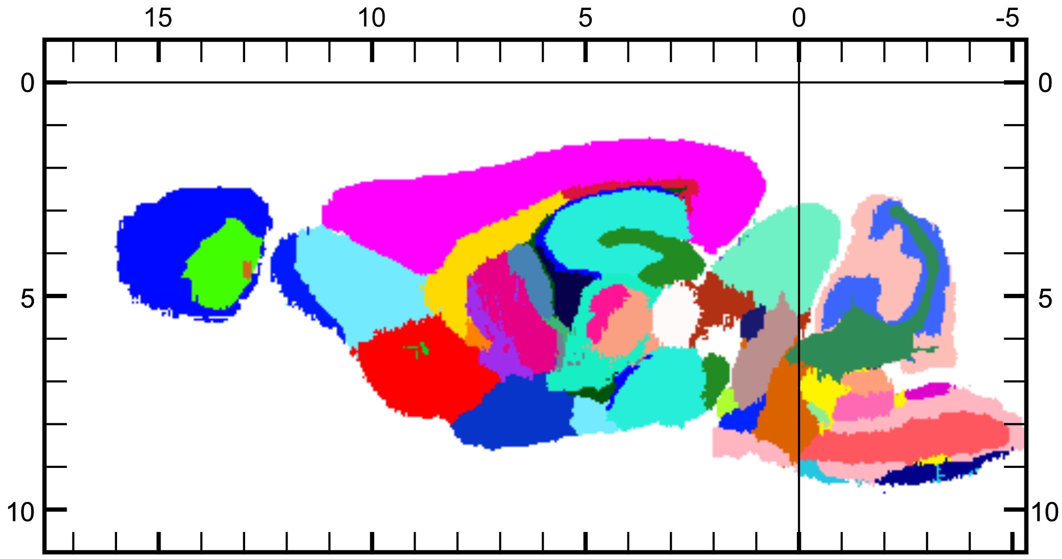

segmentation

Sagittal cross-section 11
2.0 mm right from midline

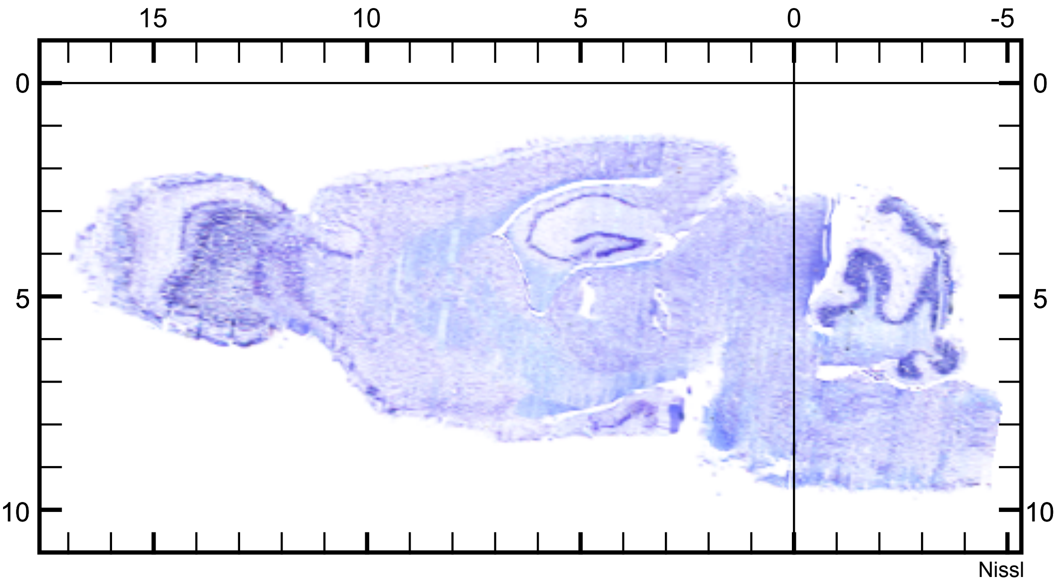

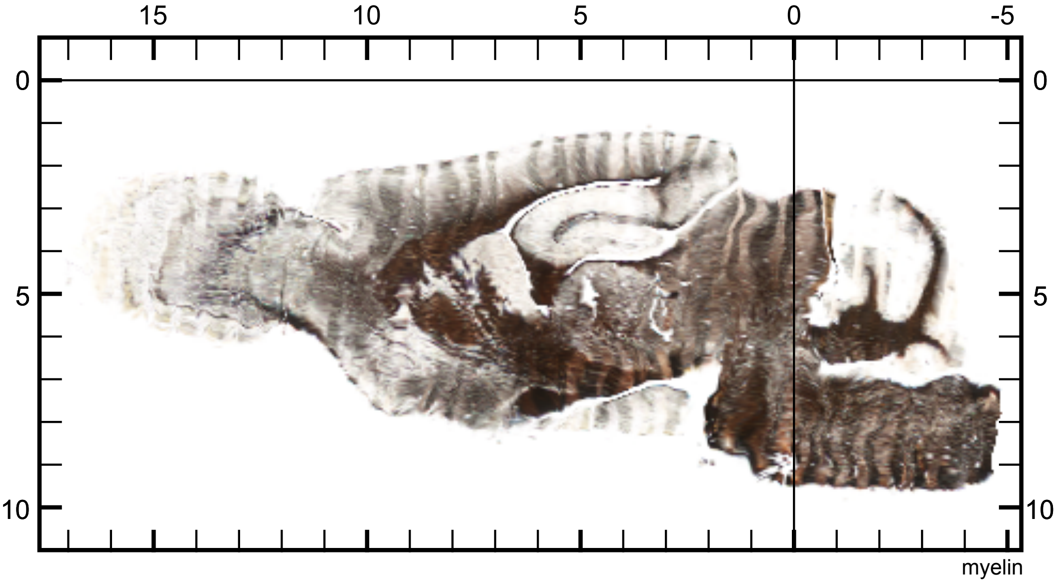

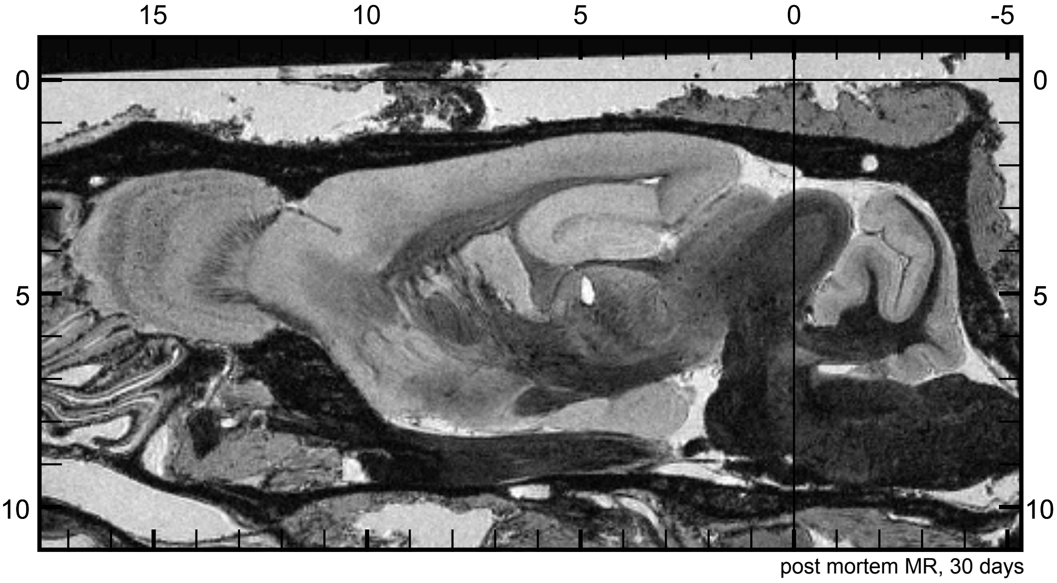

Sagittal cross-section 11 (continued)
2.0 mm right from midline

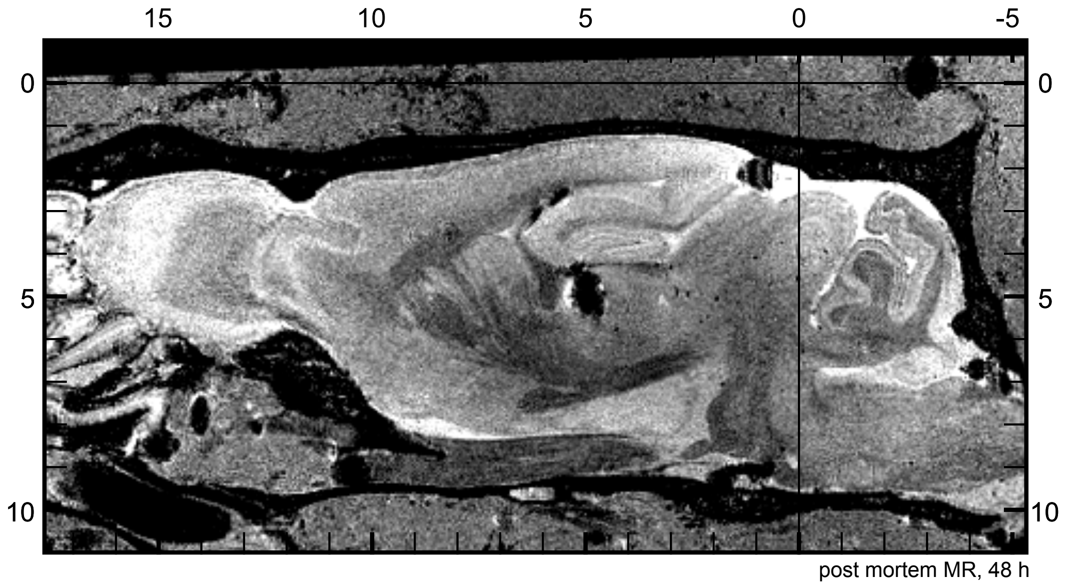

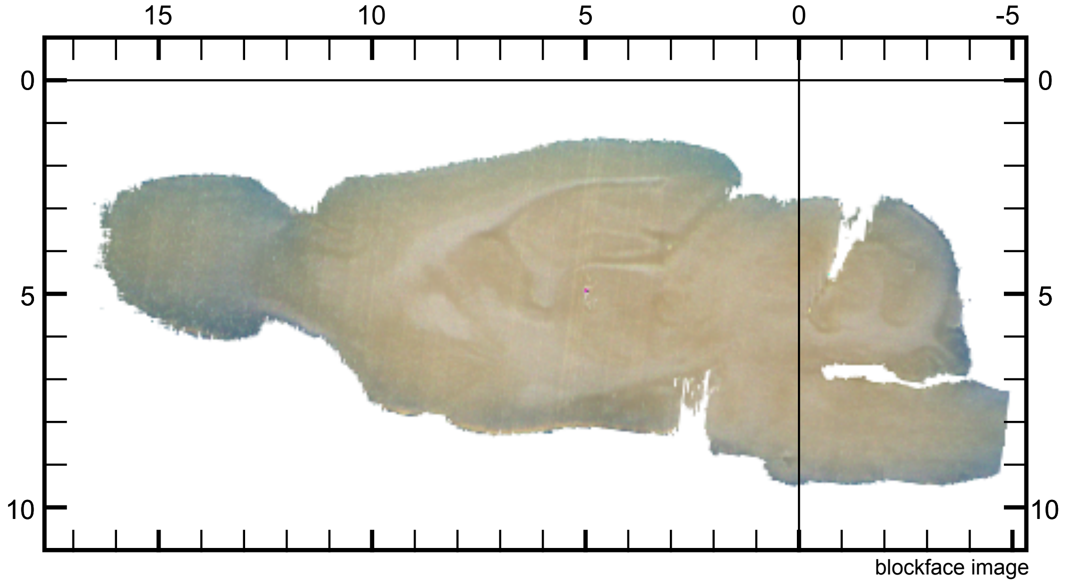

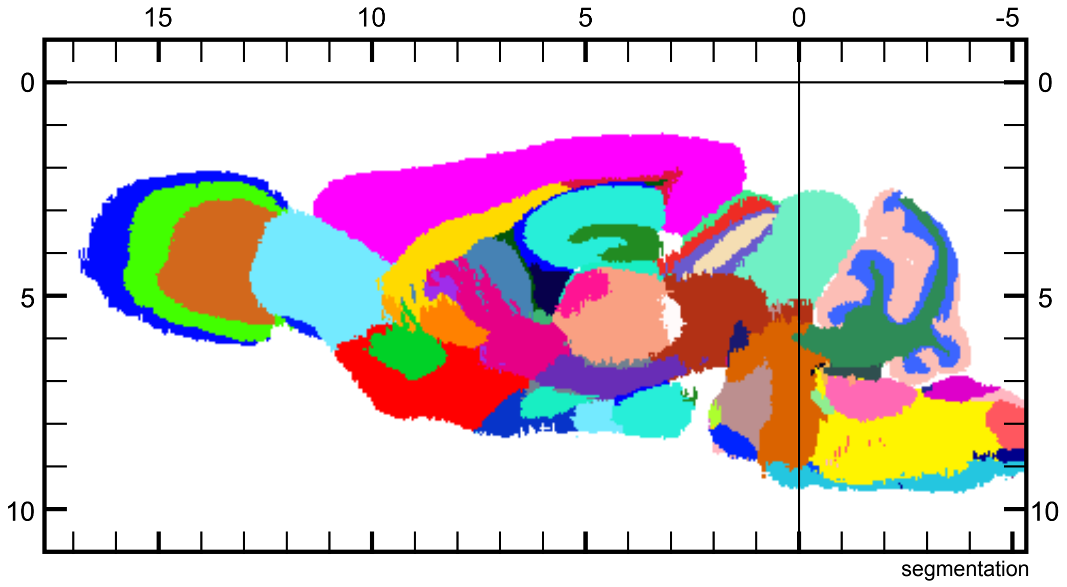

Sagittal cross-section 12
1.5 mm right from midline

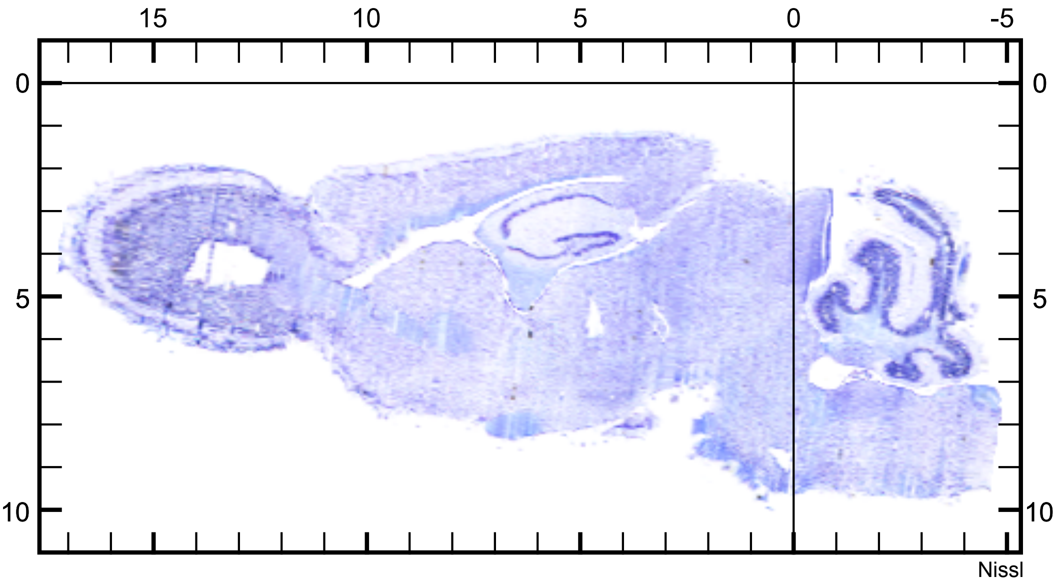

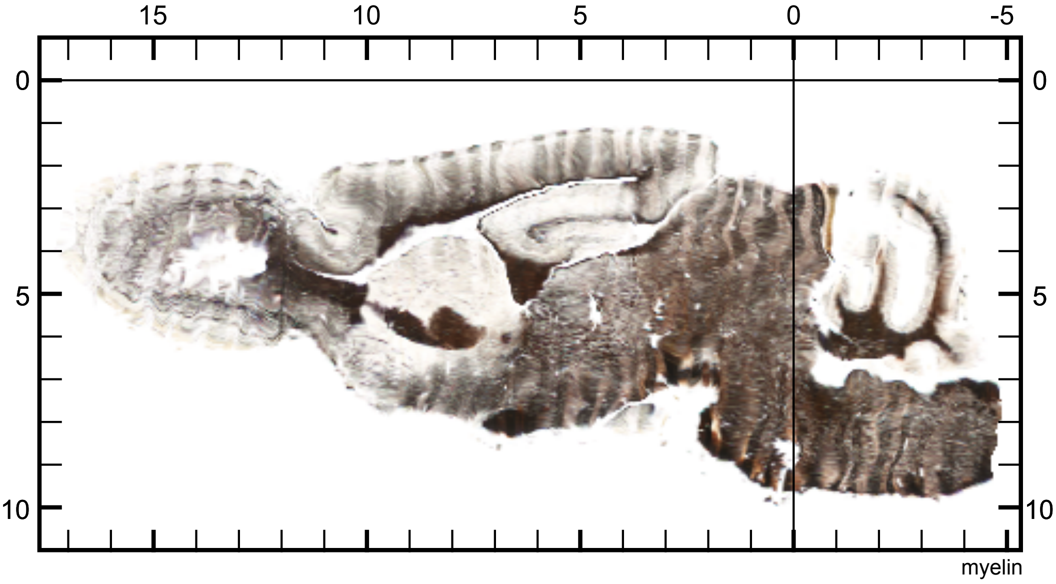

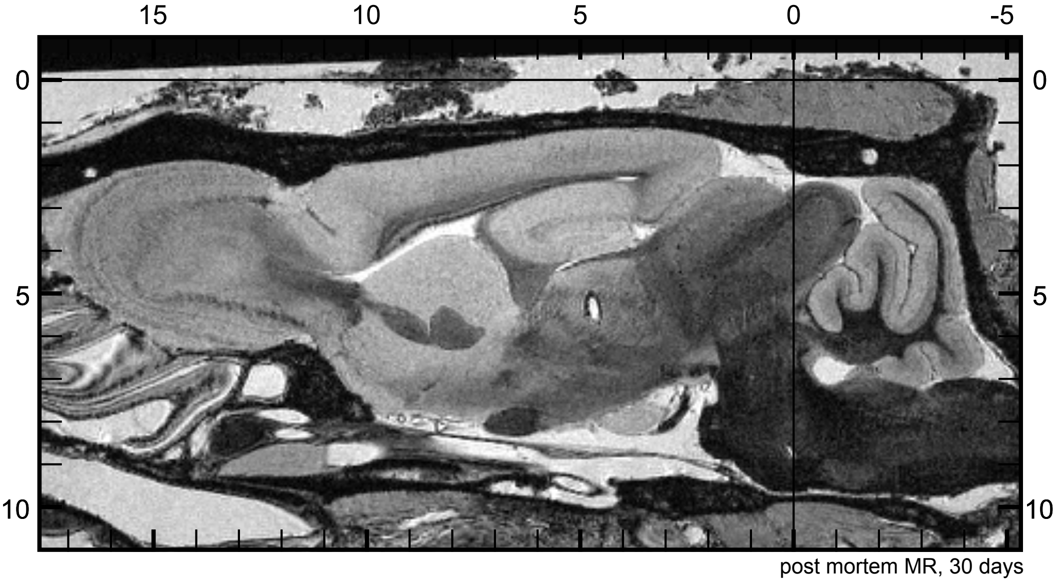

Sagittal cross-section 12 (continued)
1.5 mm right from midline

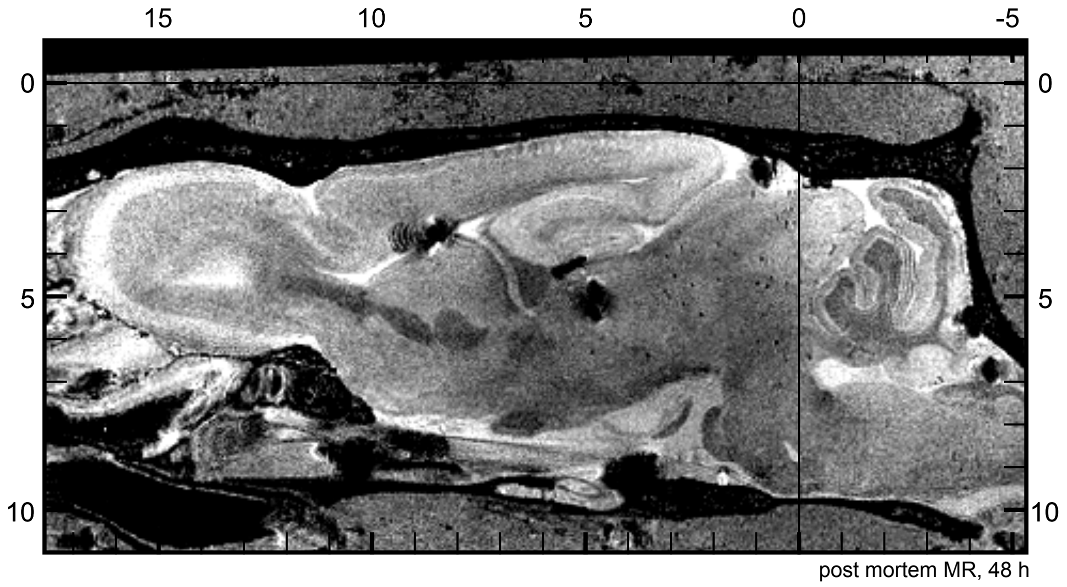

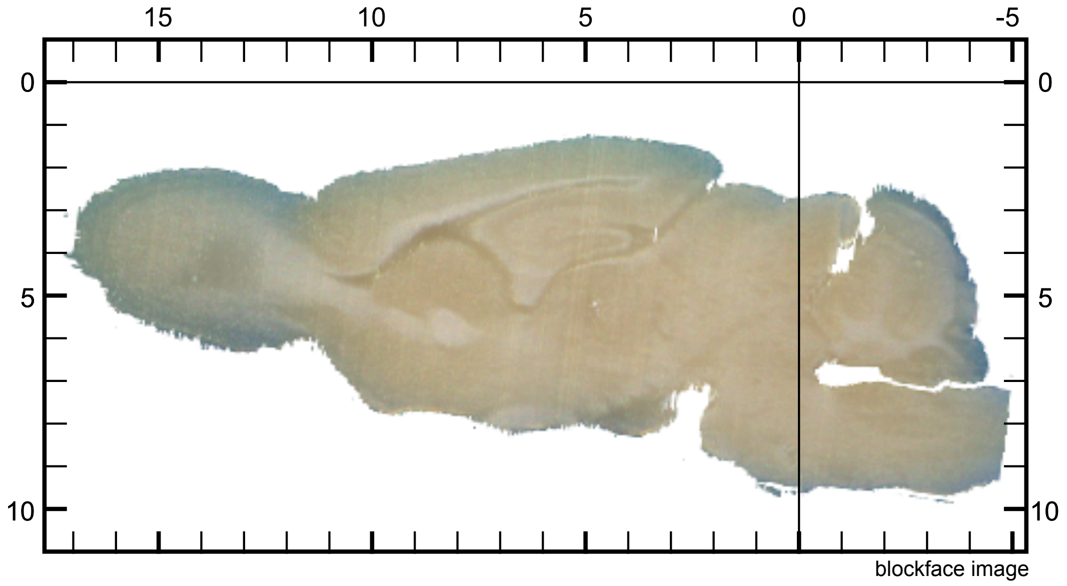

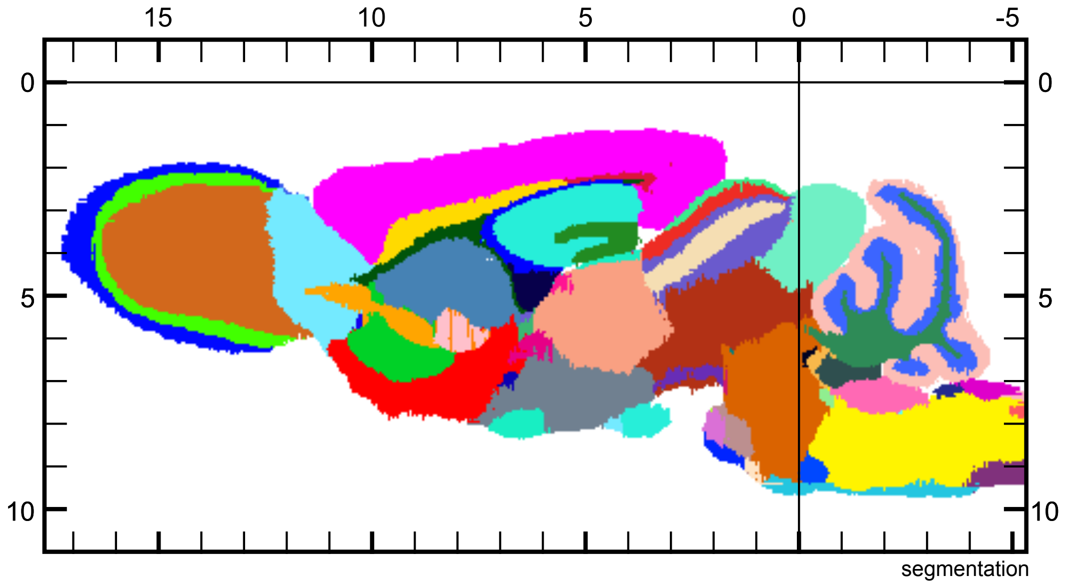

Sagittal cross-section 13
1.0 mm right from midline

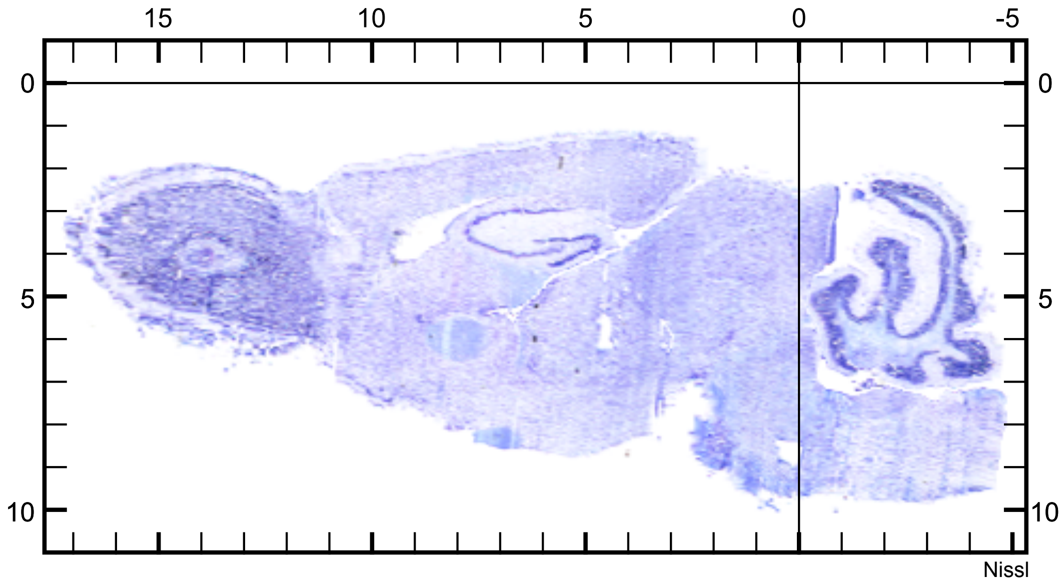

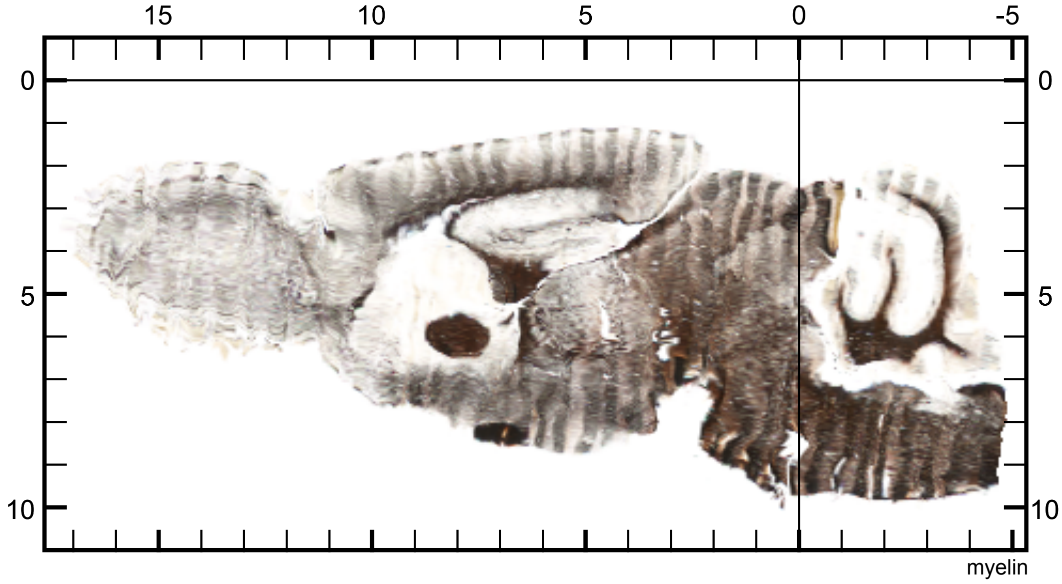

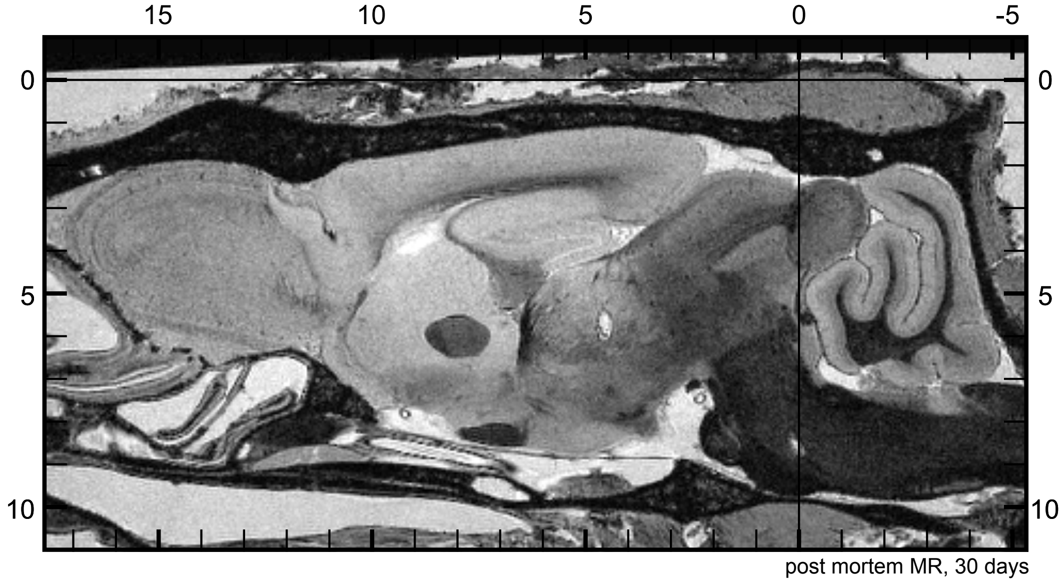

Sagittal cross-section 13 (continued)
1.0 mm right from midline

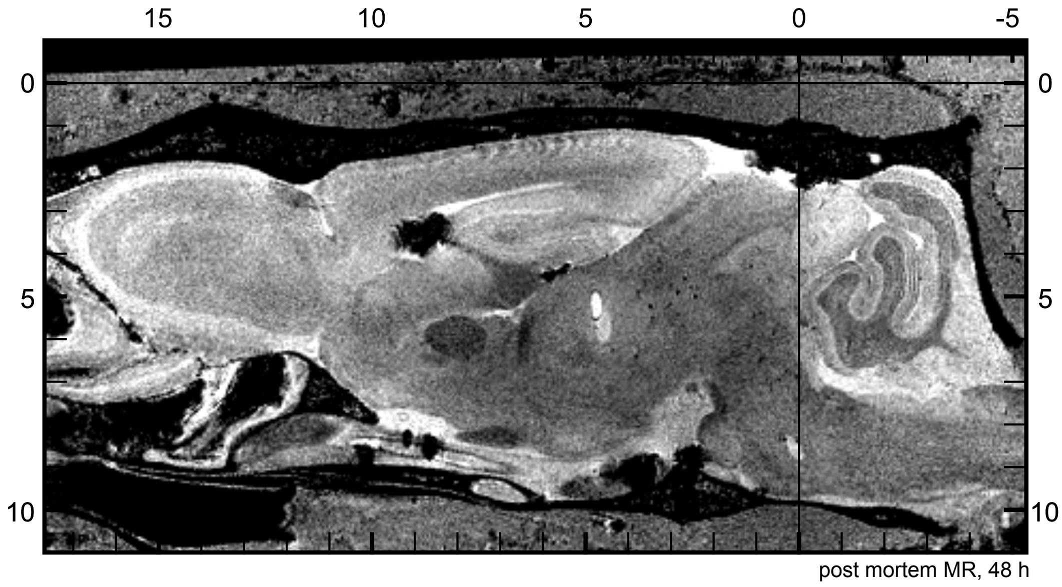

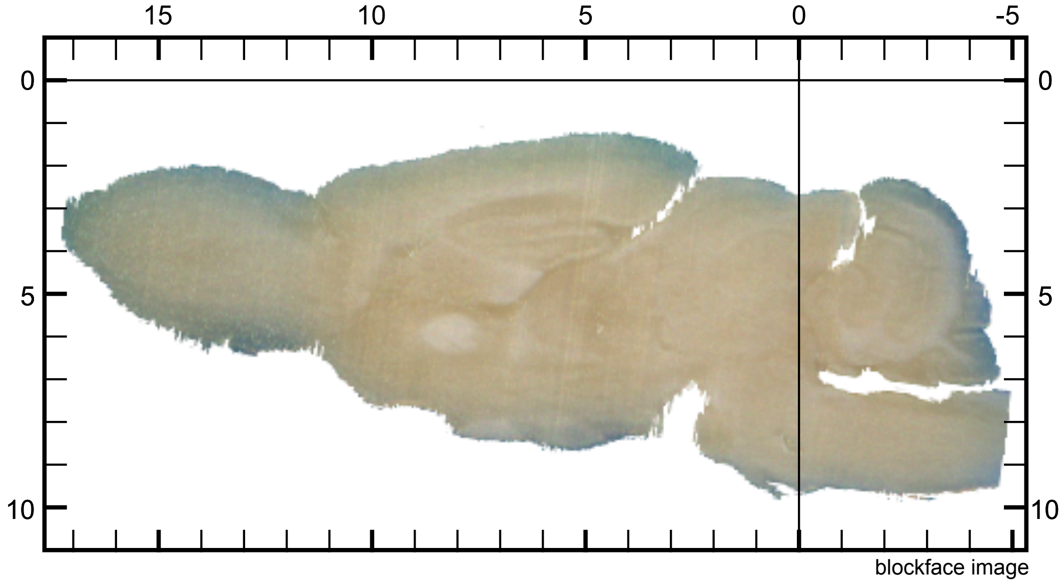

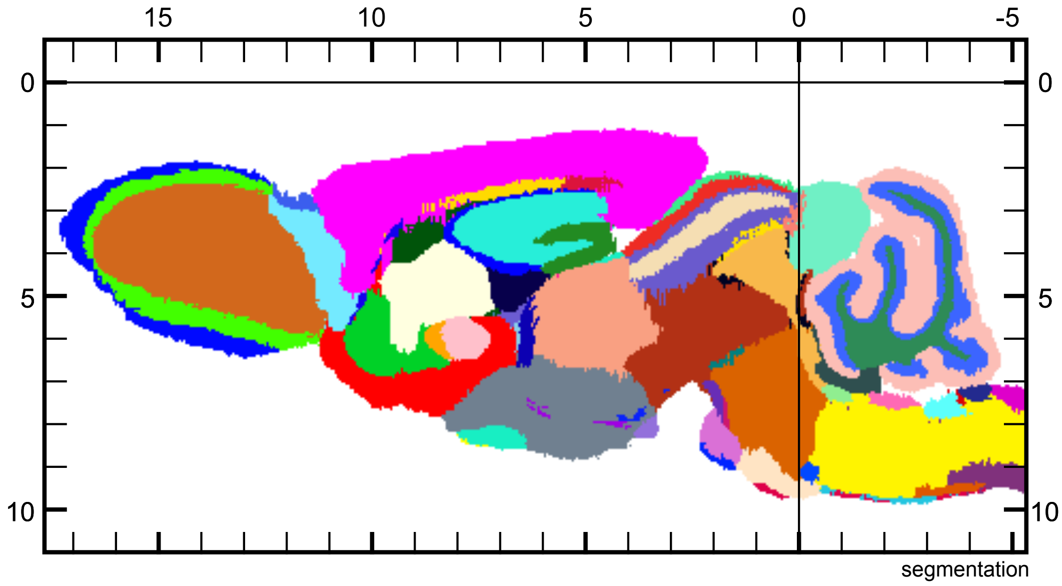

Sagittal cross-section 14
0.5 mm right from midline

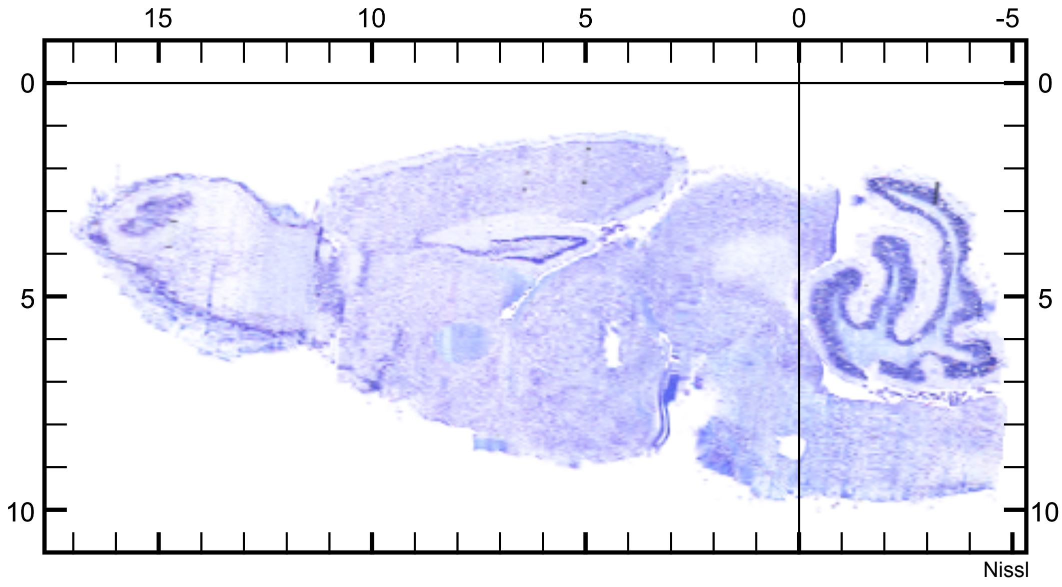

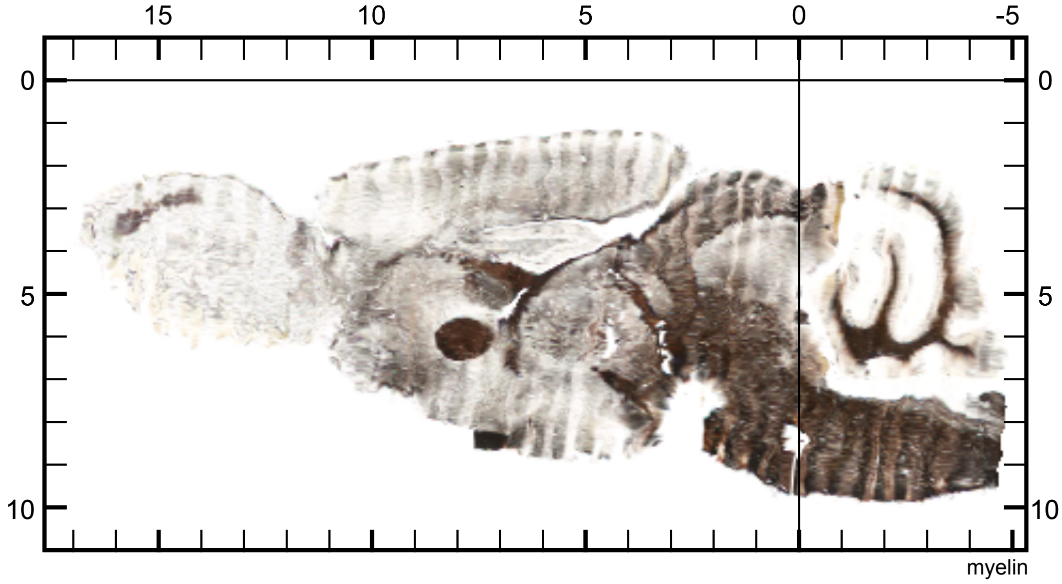

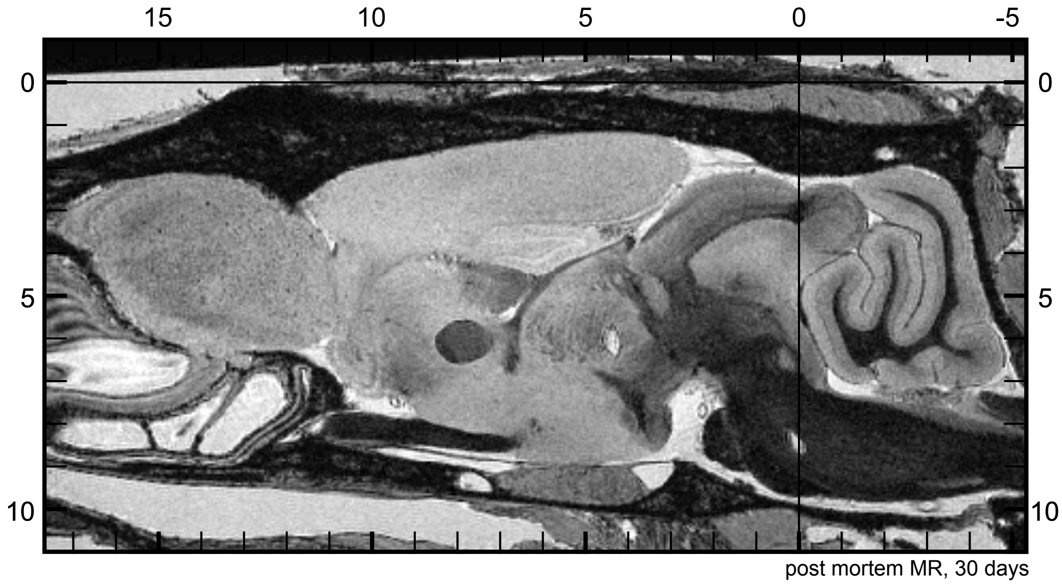

Sagittal cross-section 14 (continued)
0.5 mm right from midline

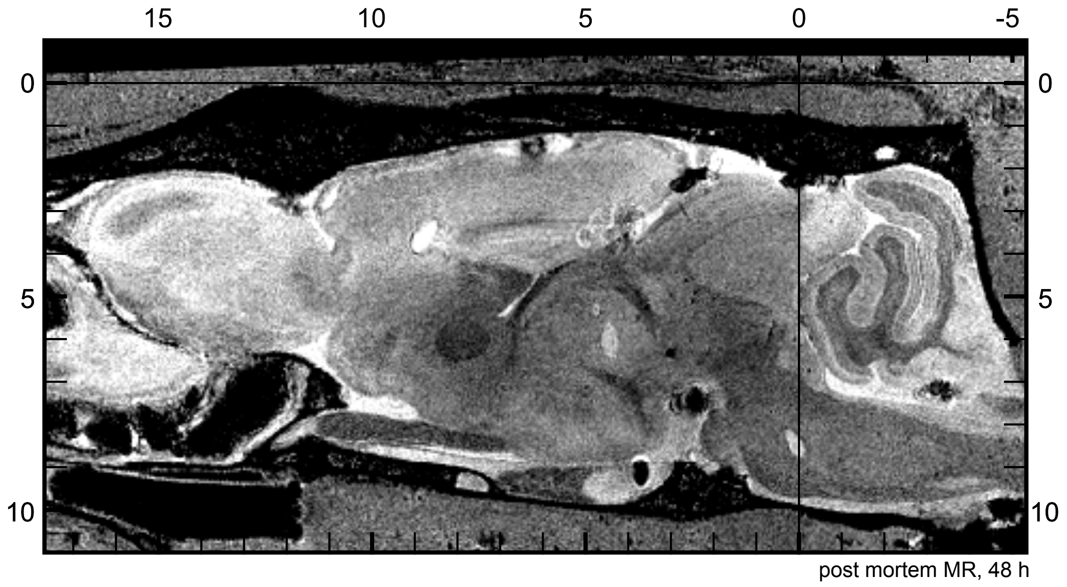

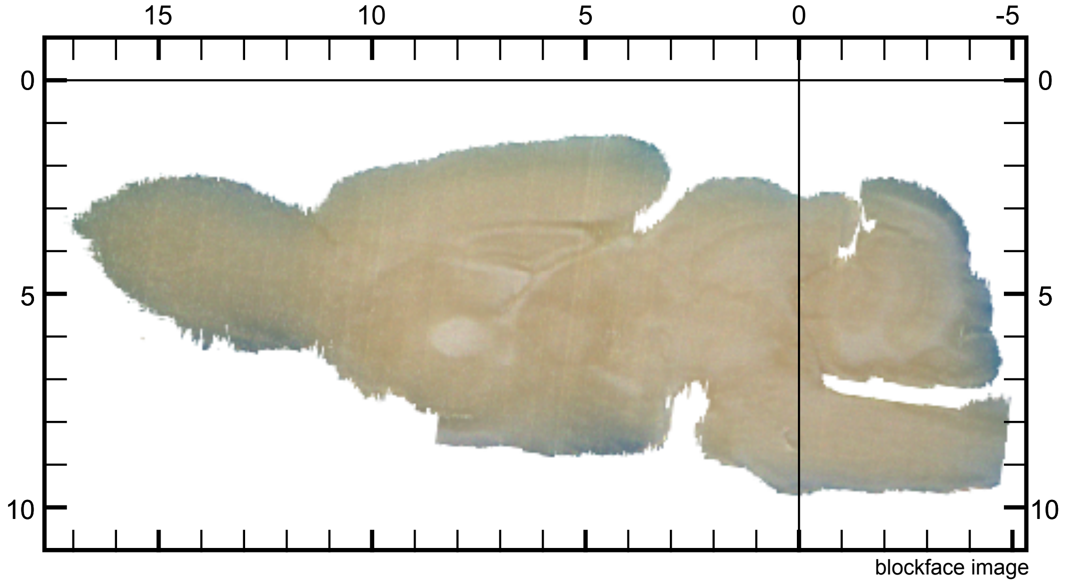

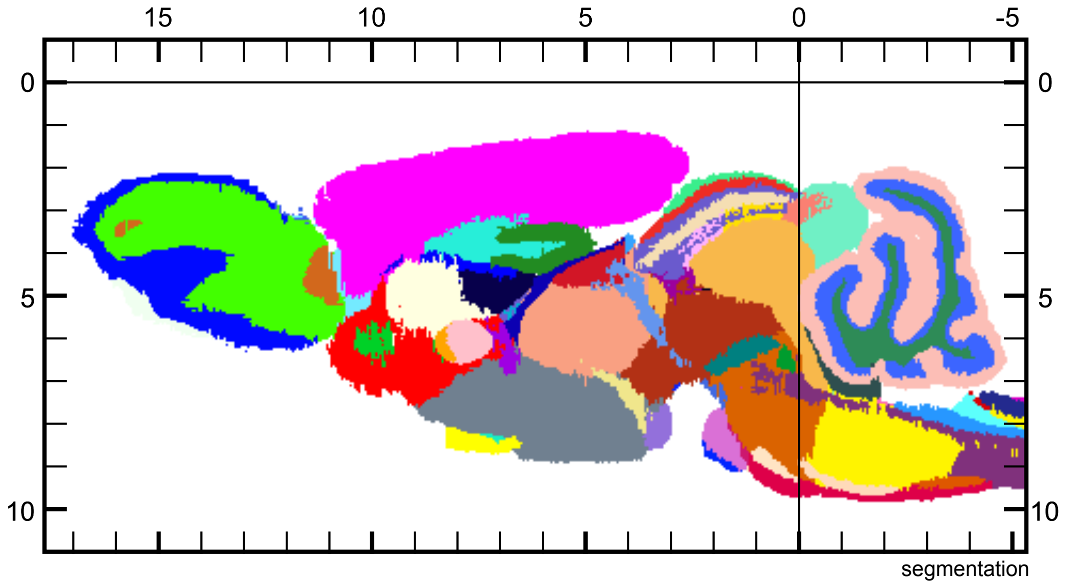

Sagittal cross-section 15
midline

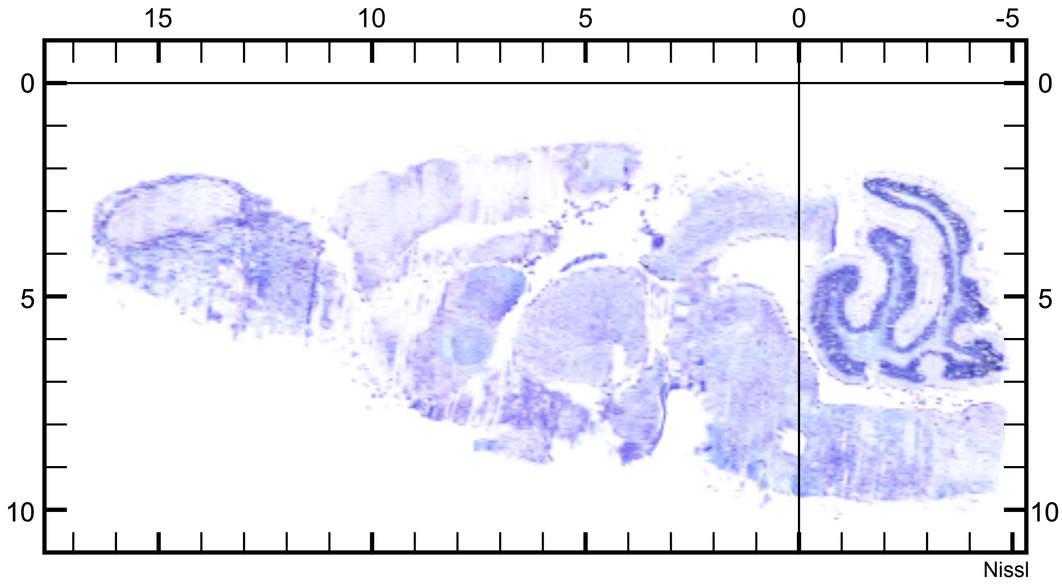

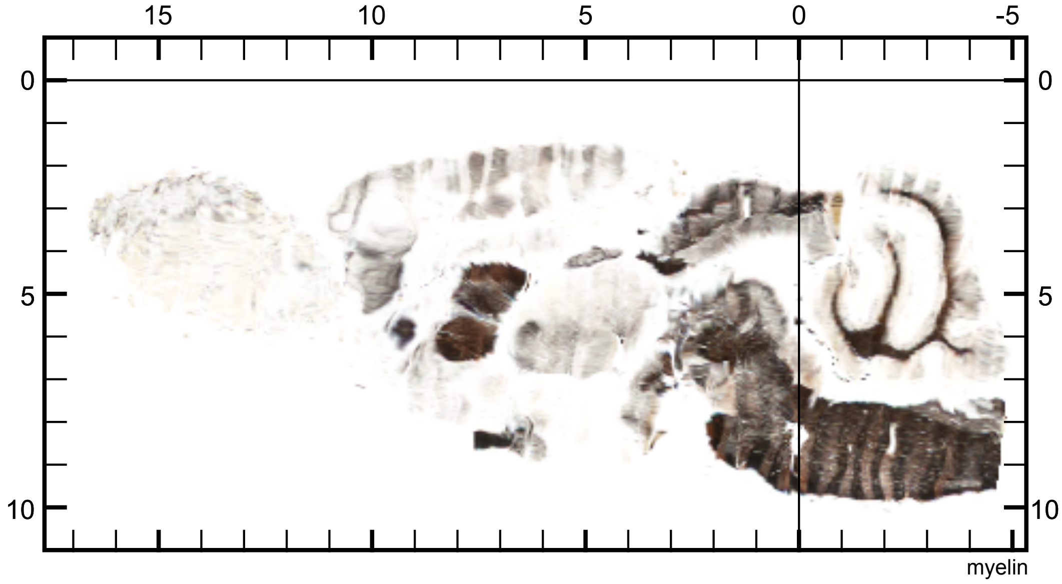

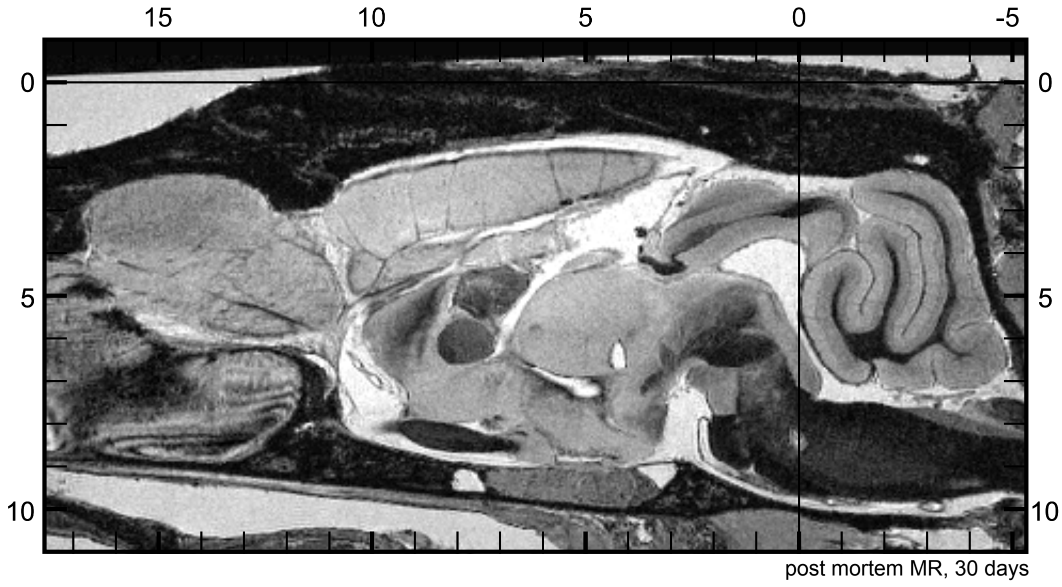

Sagittal cross-section 15 (continued)
midline

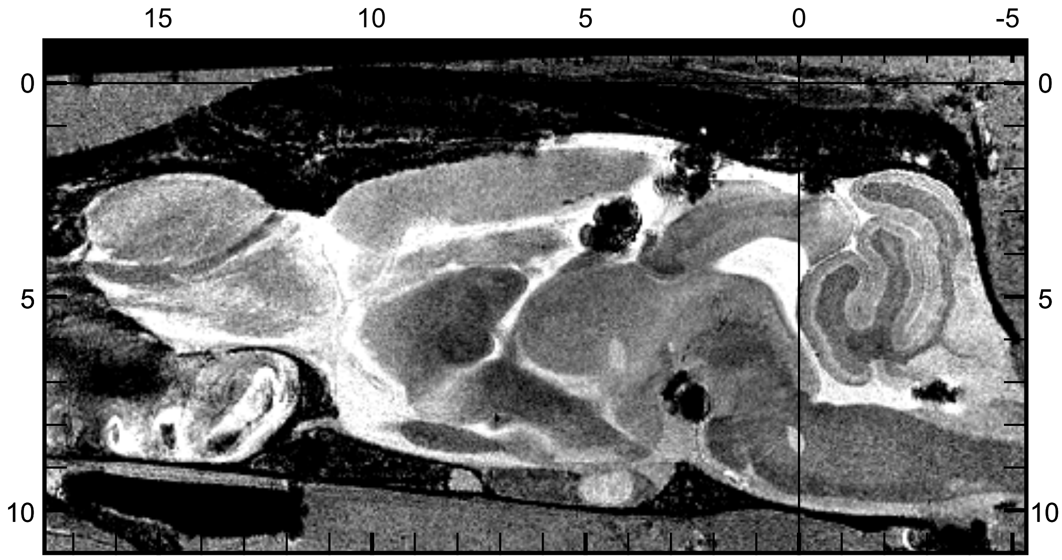

post mortem MR, 48 h

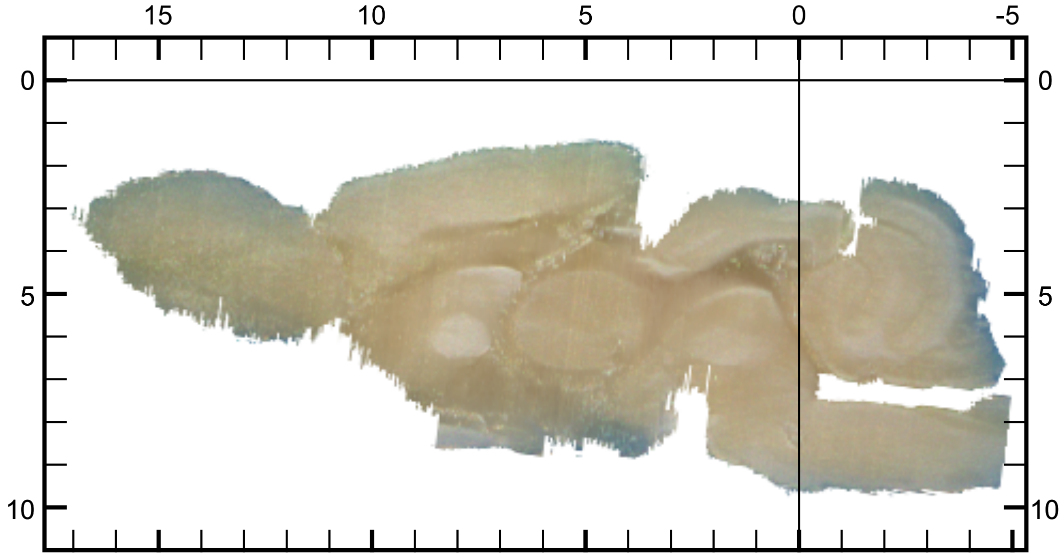

blockface image

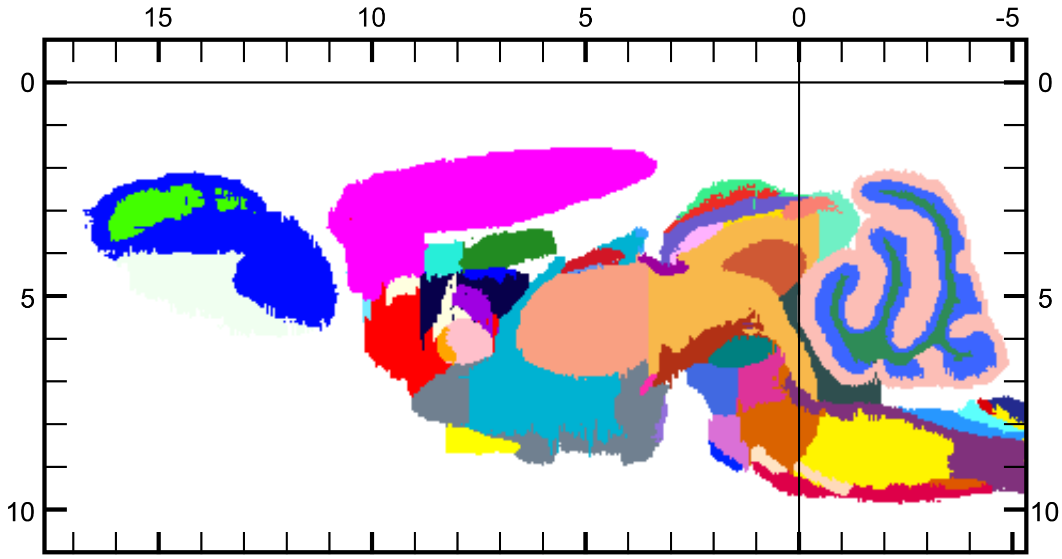

segmentation

Sagittal cross-section 16
0.5 mm left from midline

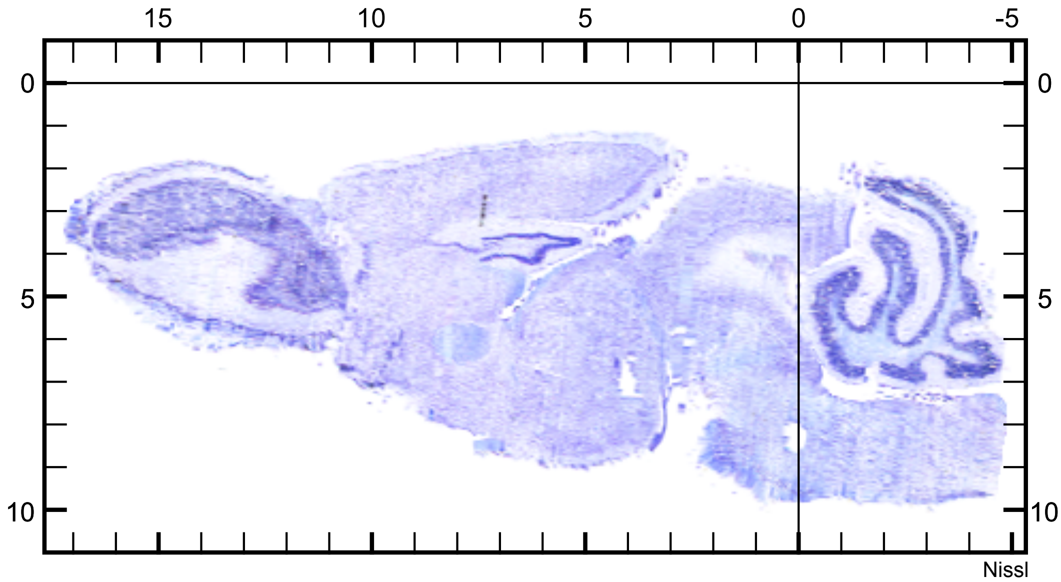

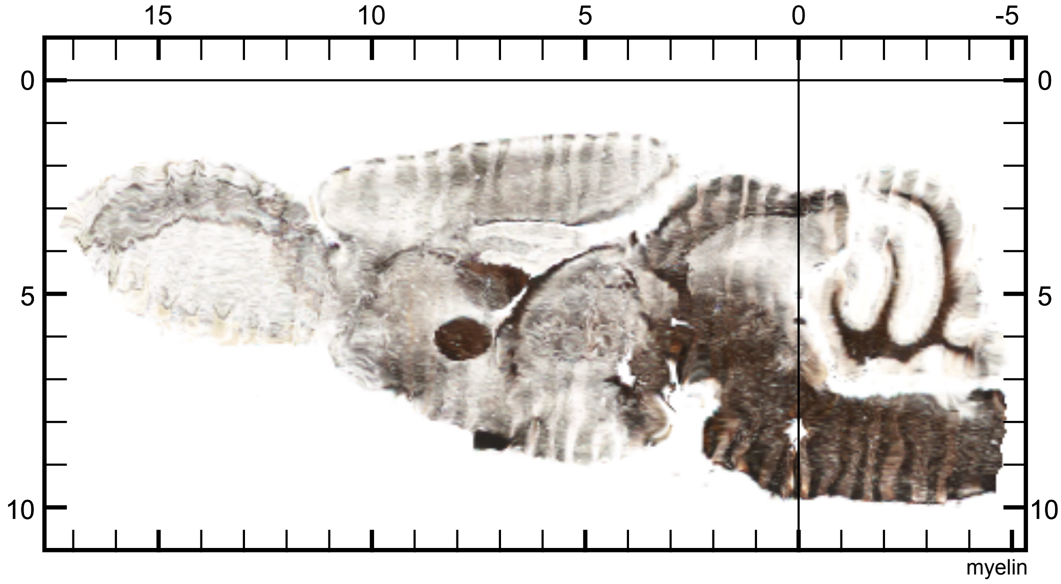

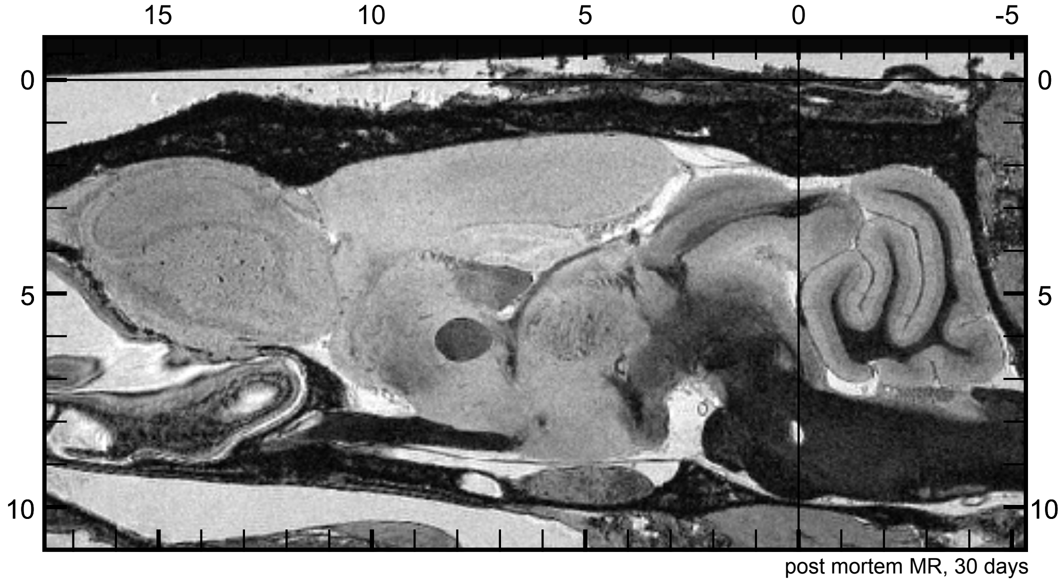

Sagittal cross-section 16 (continued)
0.5 mm left from midline

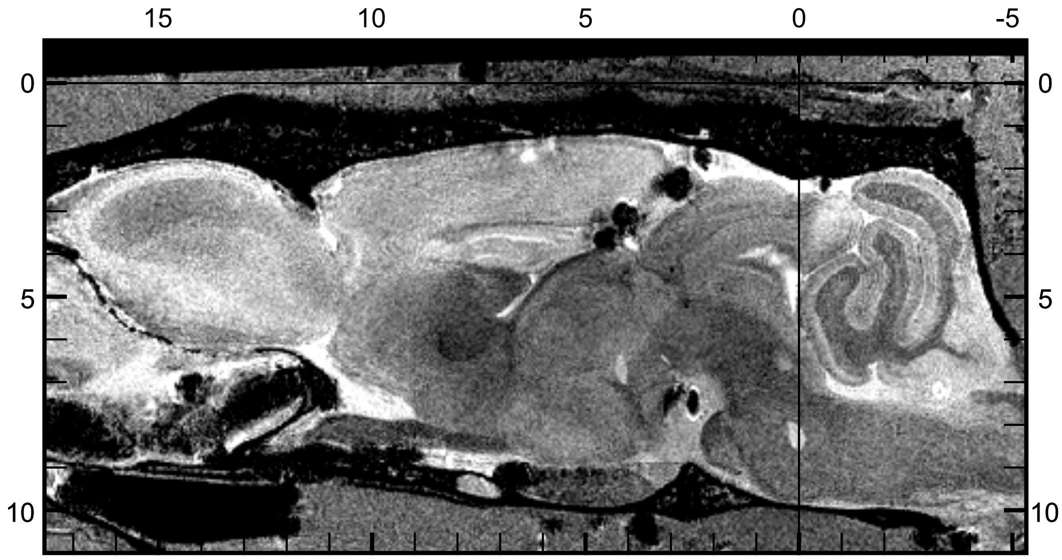

post mortem MR, 48 h

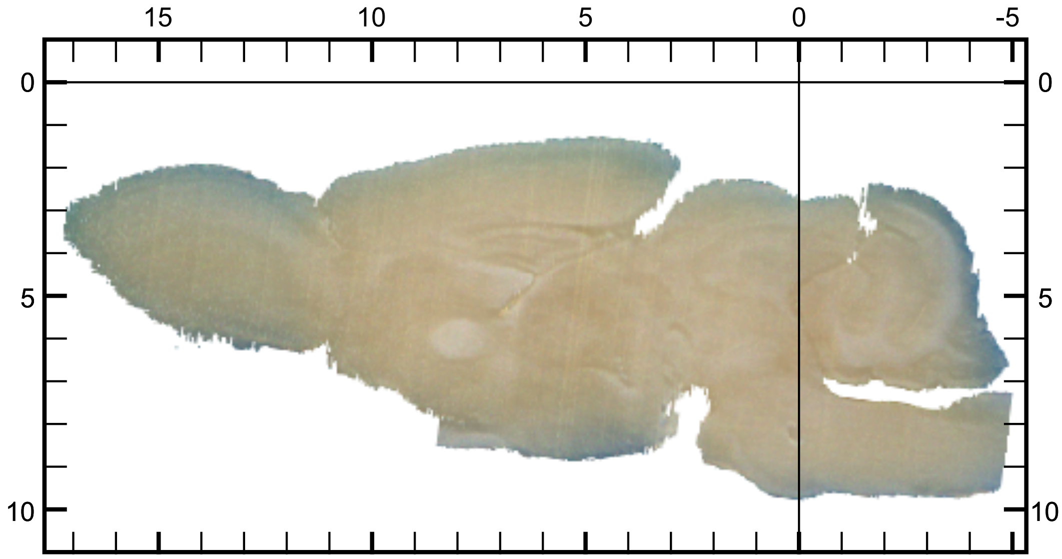

blockface image

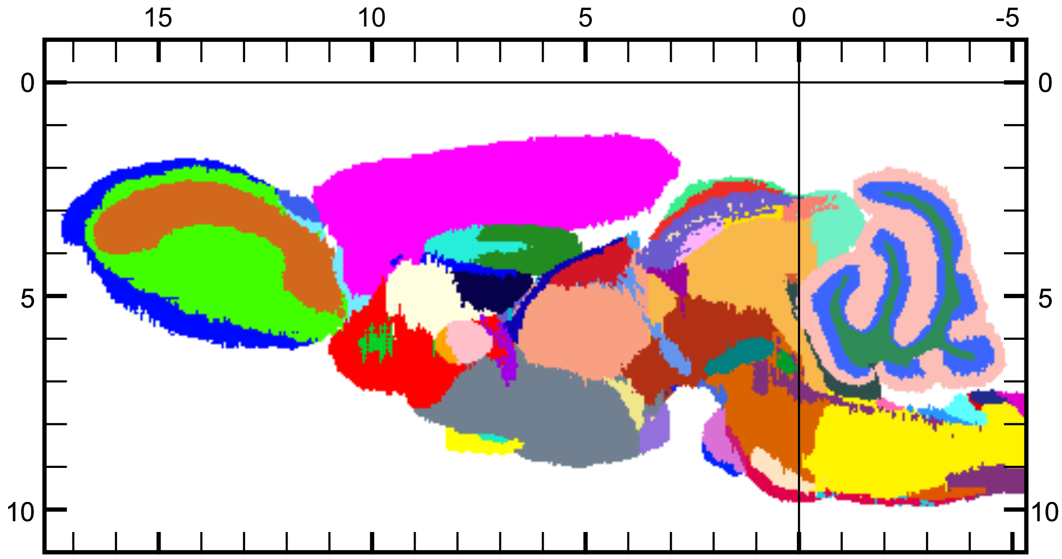

segmentation

Sagittal cross-section 17
1.0 mm left from midline

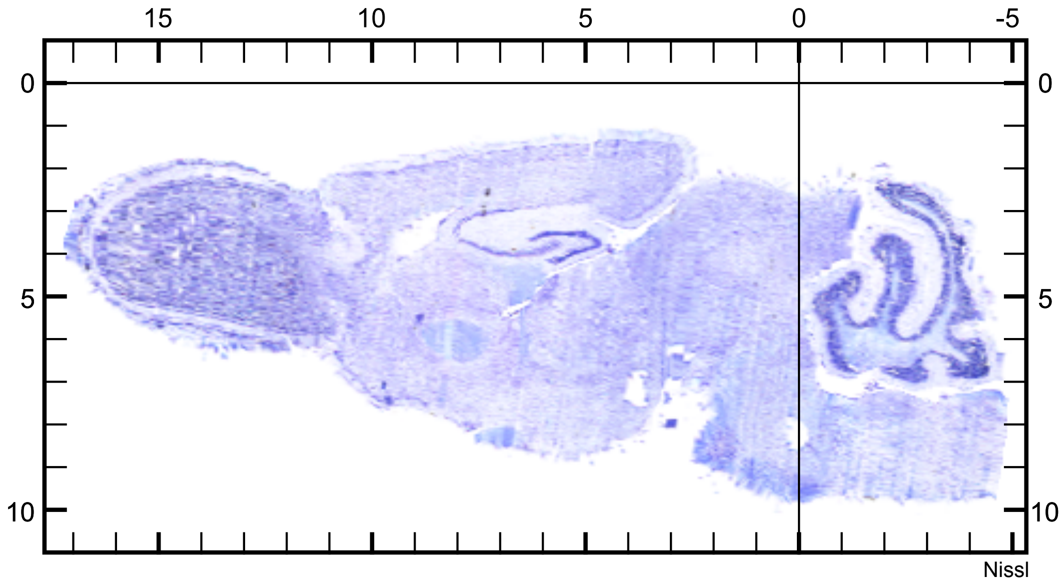

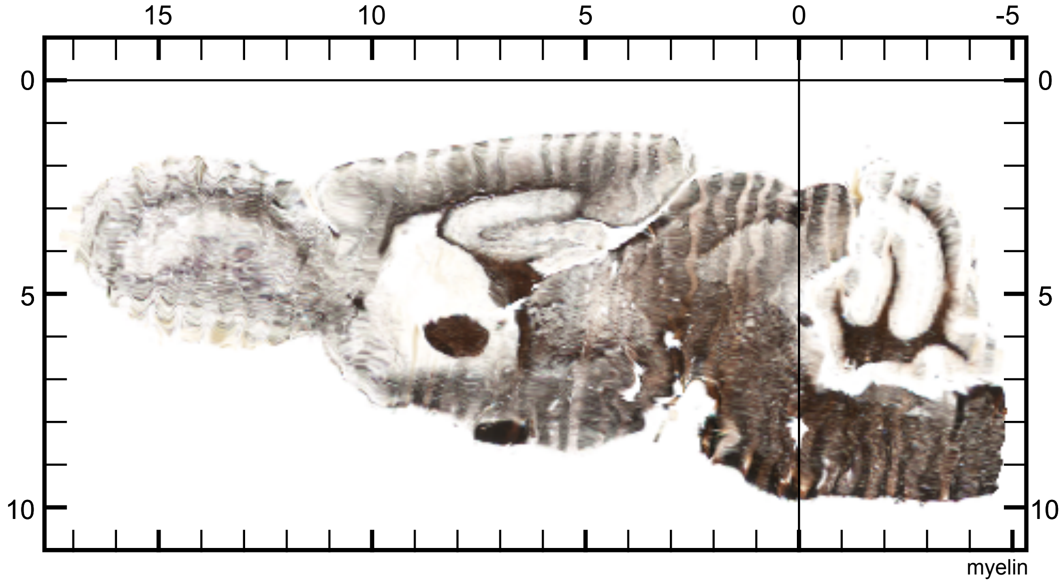

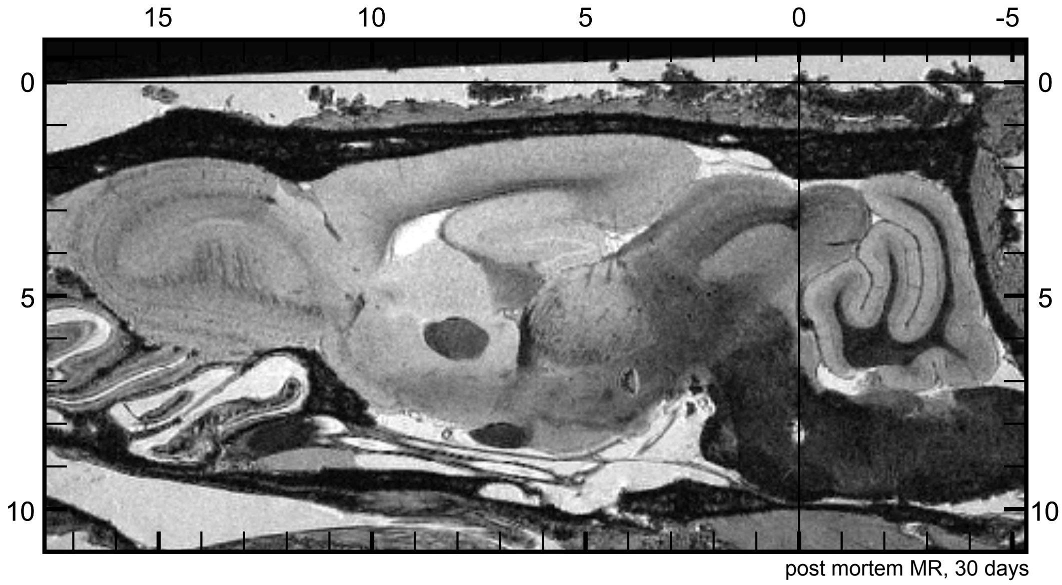

Sagittal cross-section 17 (continued)
1.0 mm left from midline

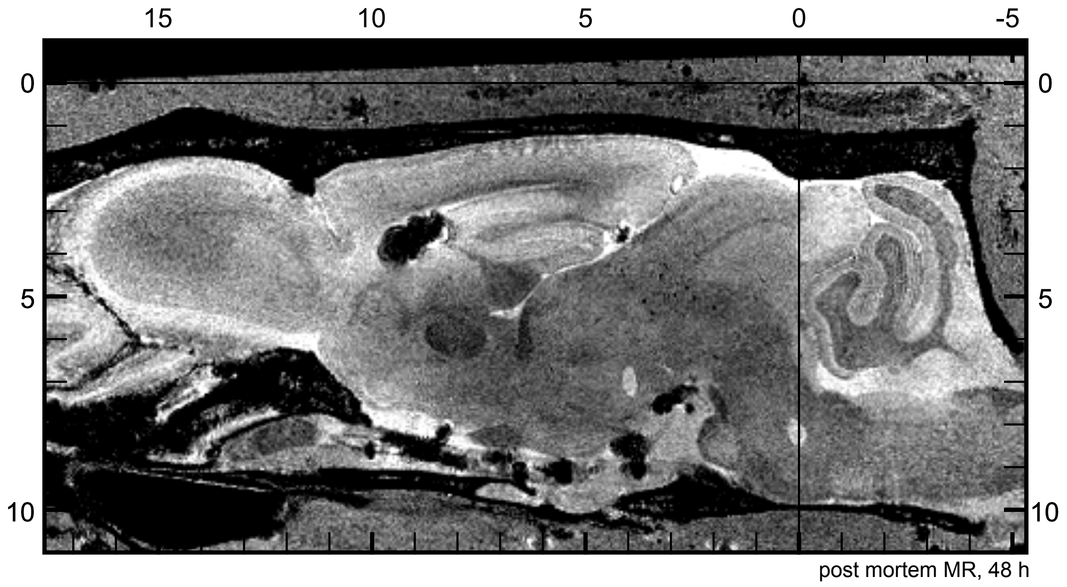

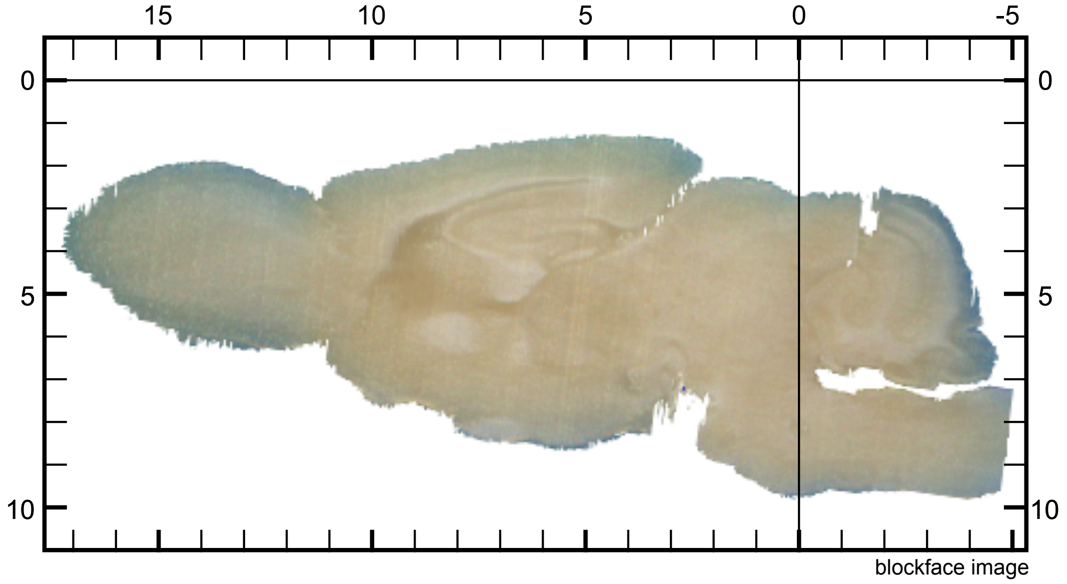

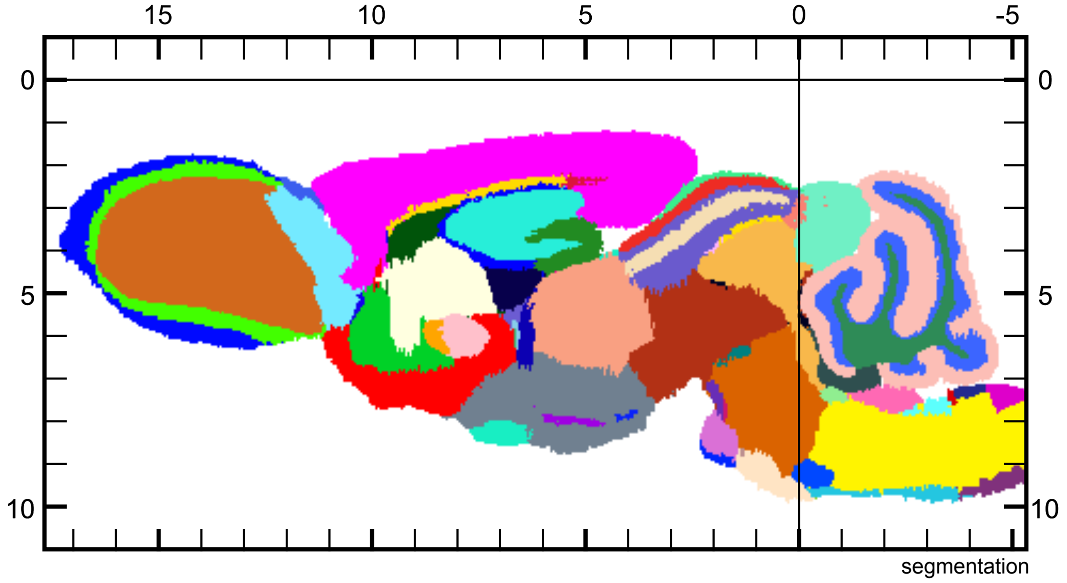

Sagittal cross-section 18
1.5 mm left from midline

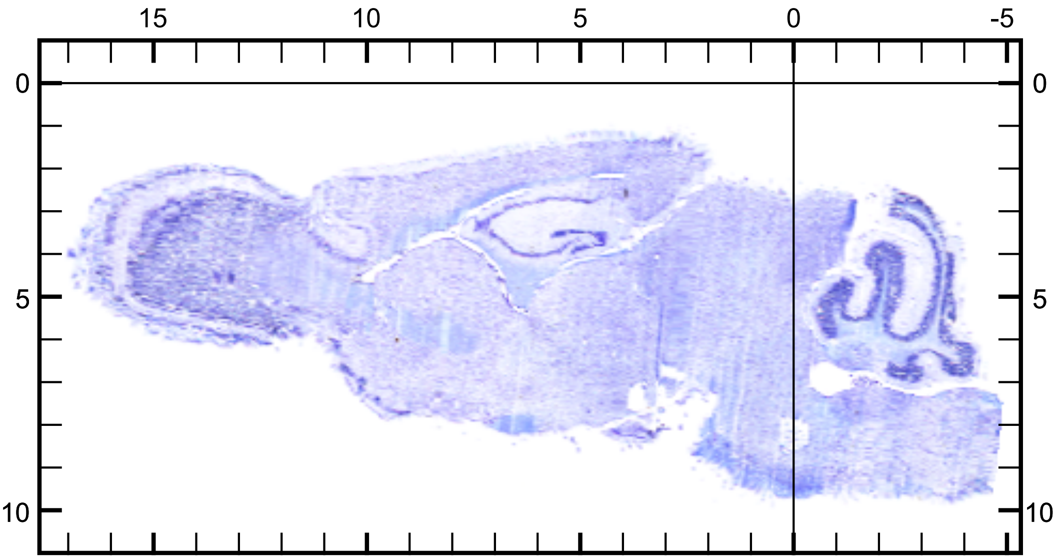

Nissl

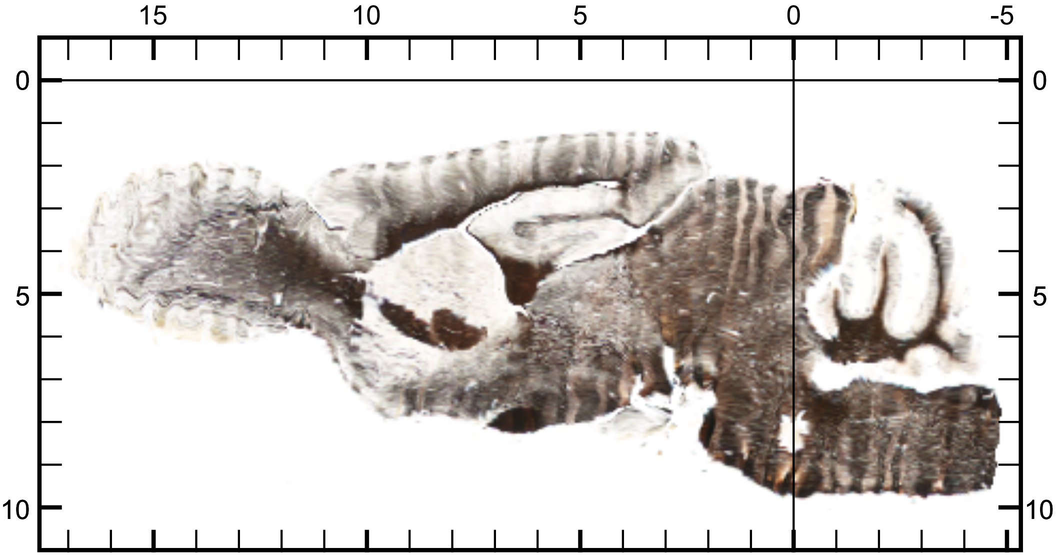

myelin

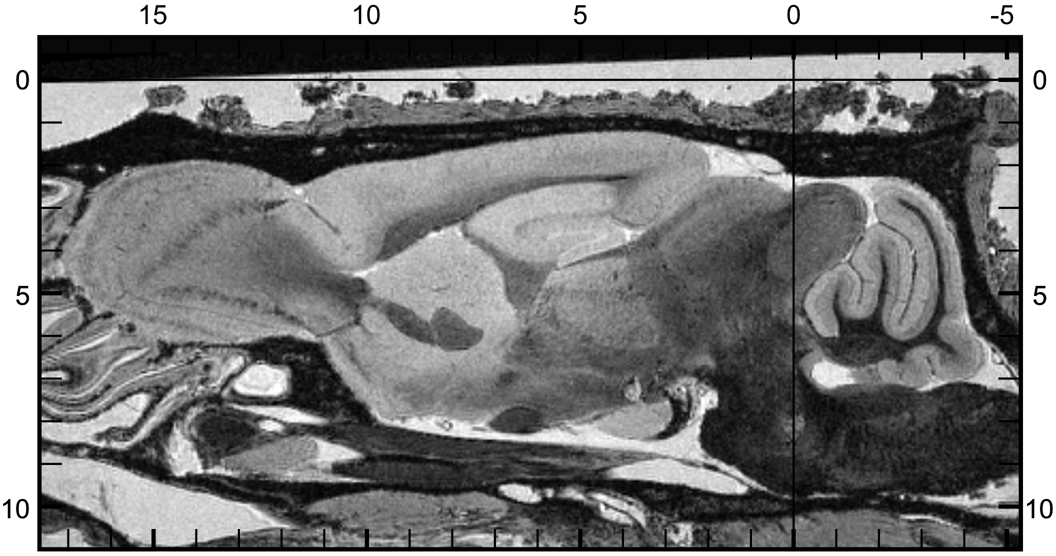

post mortem MR, 30 days

Sagittal cross-section 18 (continued)
1.5 mm left from midline

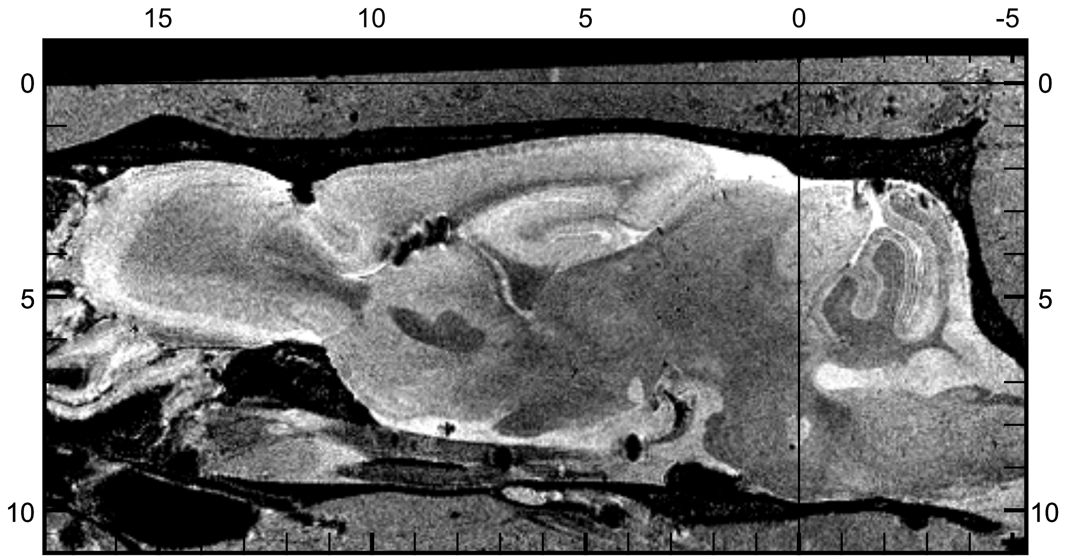

post mortem MR, 48 h

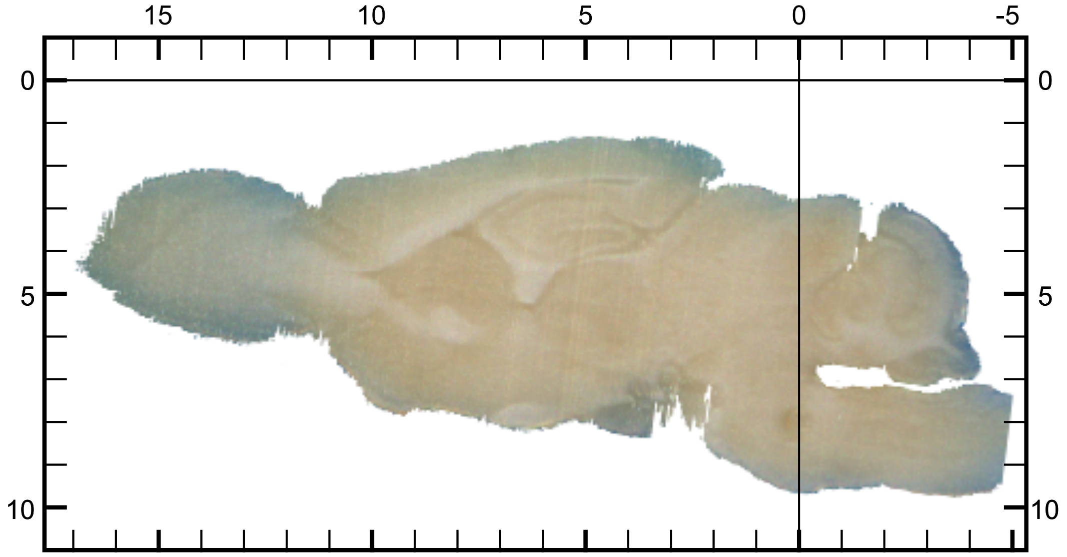

blockface image

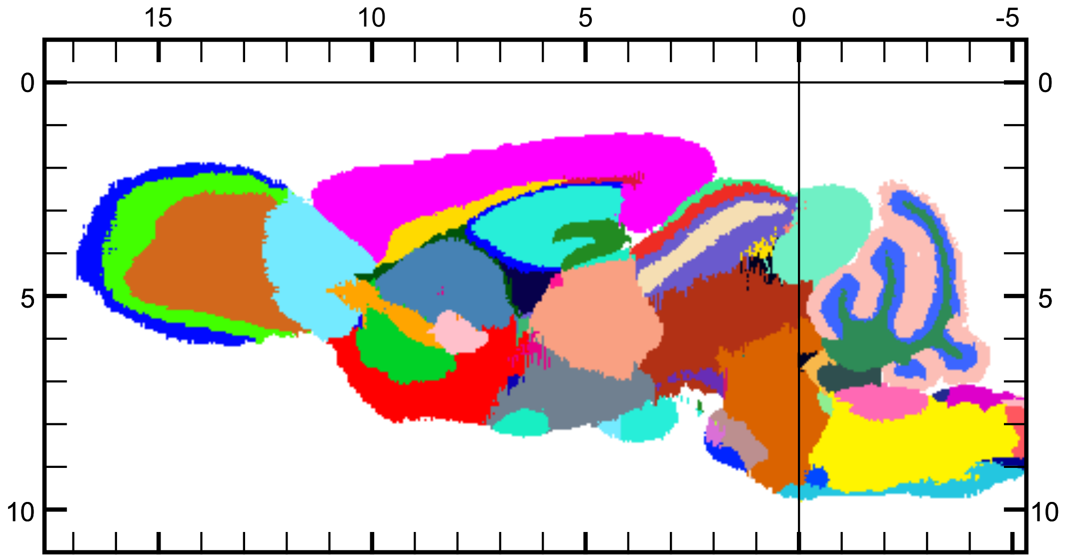

segmentation

Sagittal cross-section 19
2.0 mm left from midline

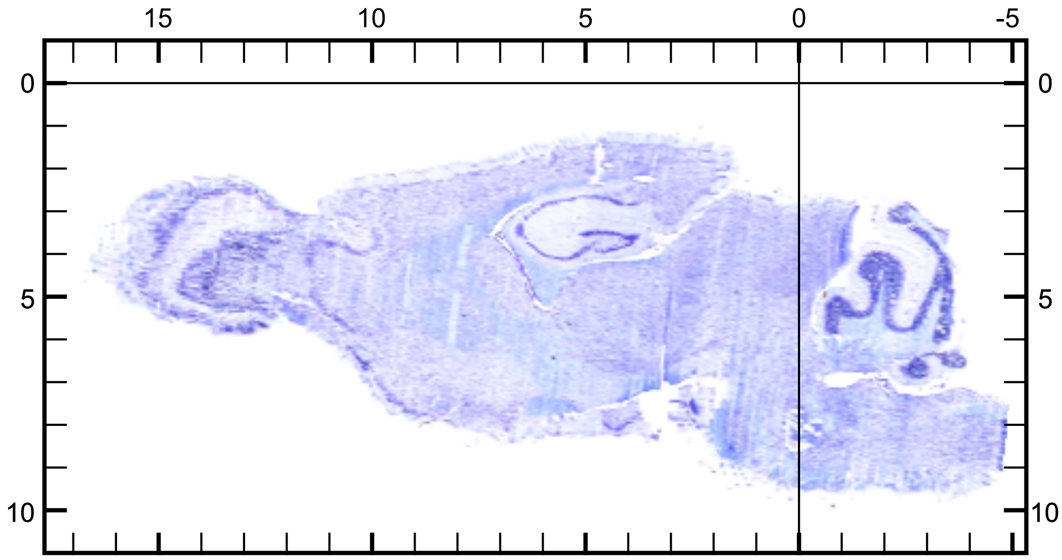

Nissl

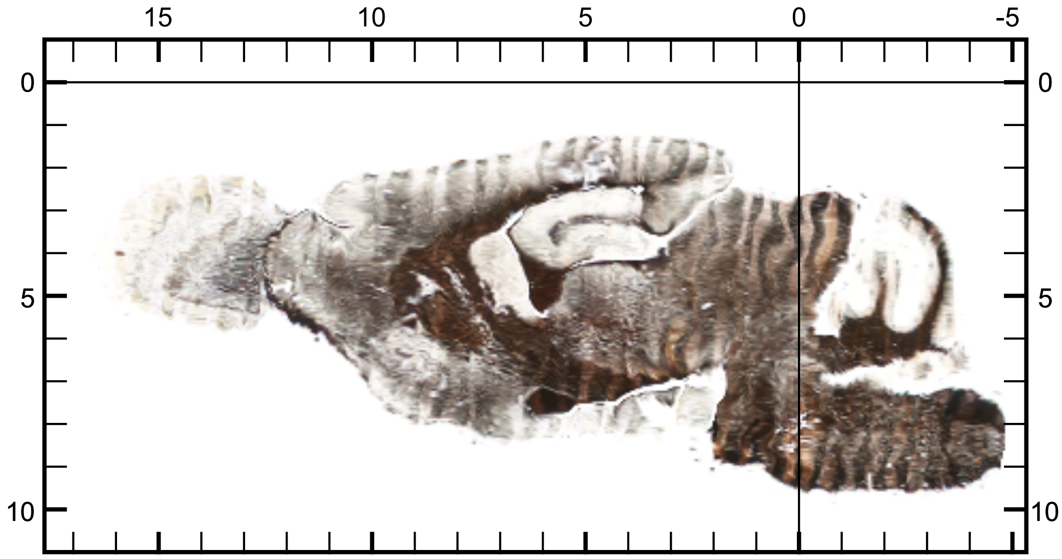

myelin

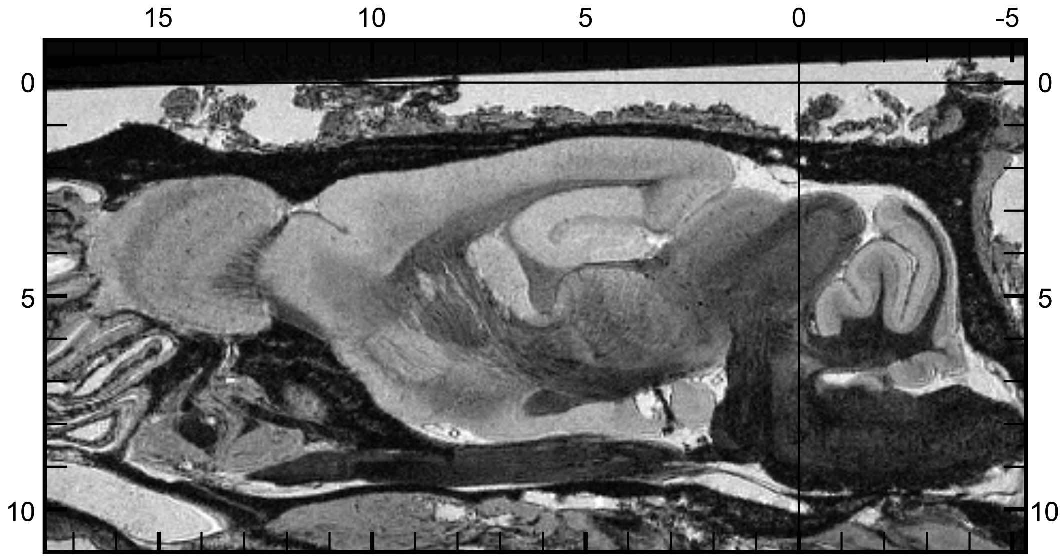

post mortem MR, 30 days

Sagittal cross-section 19 (continued)
2.0 mm left from midline

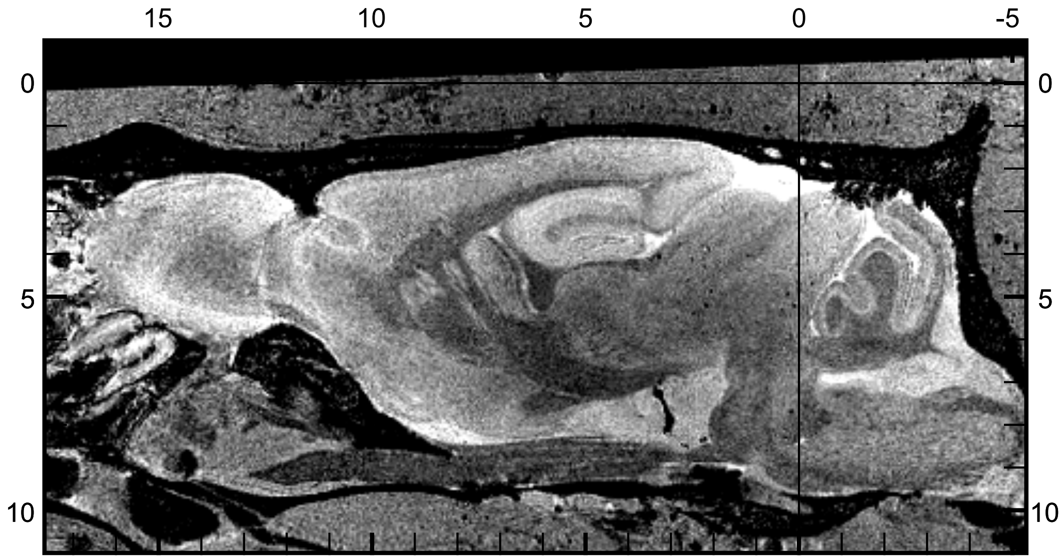

post mortem MR, 48 h

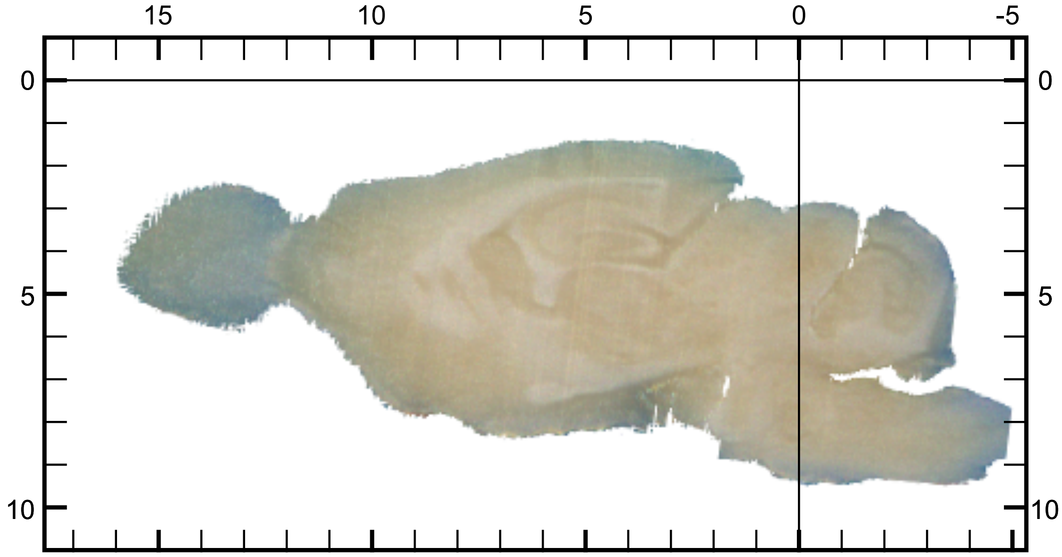

blockface image

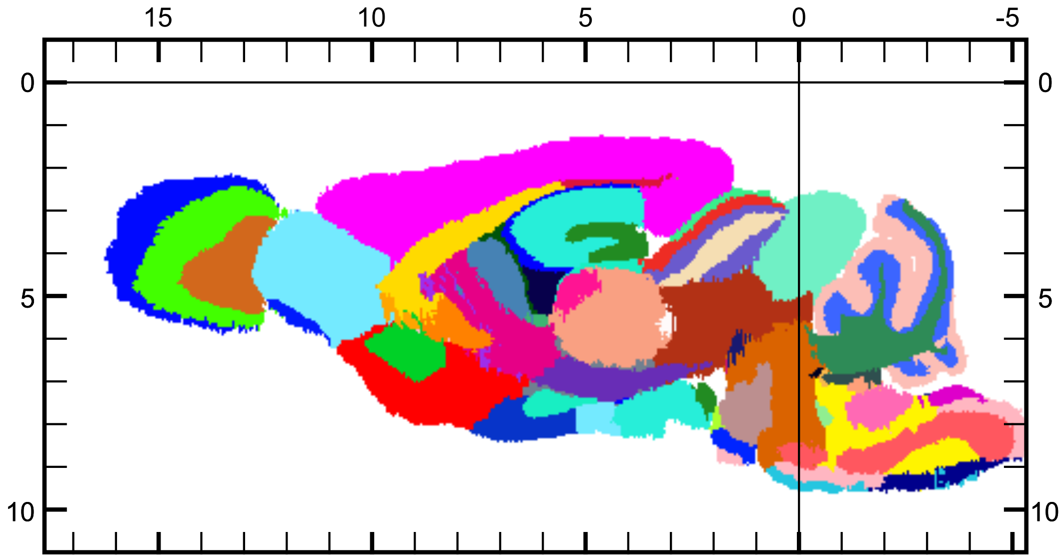

segmentation

Sagittal cross-section 20
2.5 mm left from midline

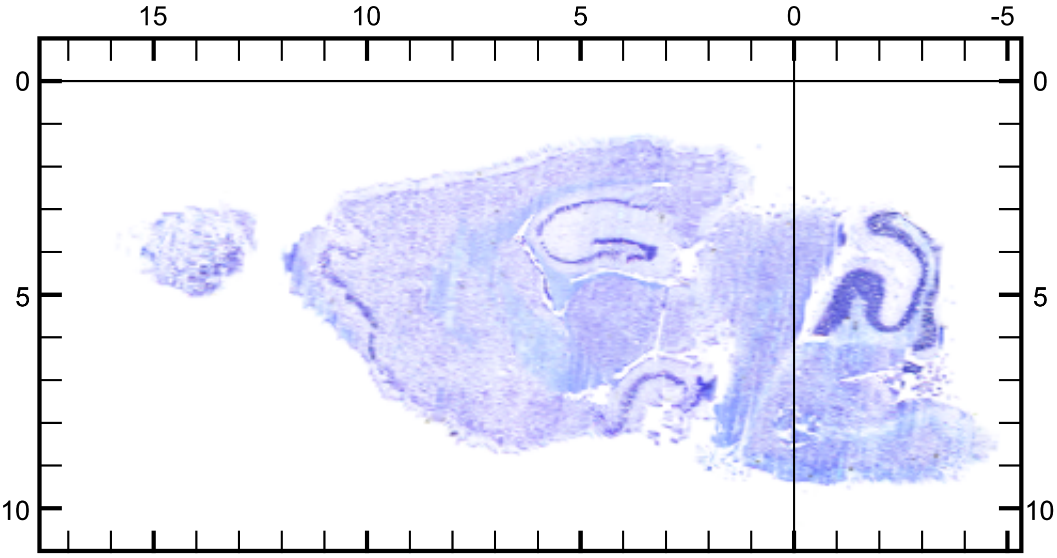

Nissl

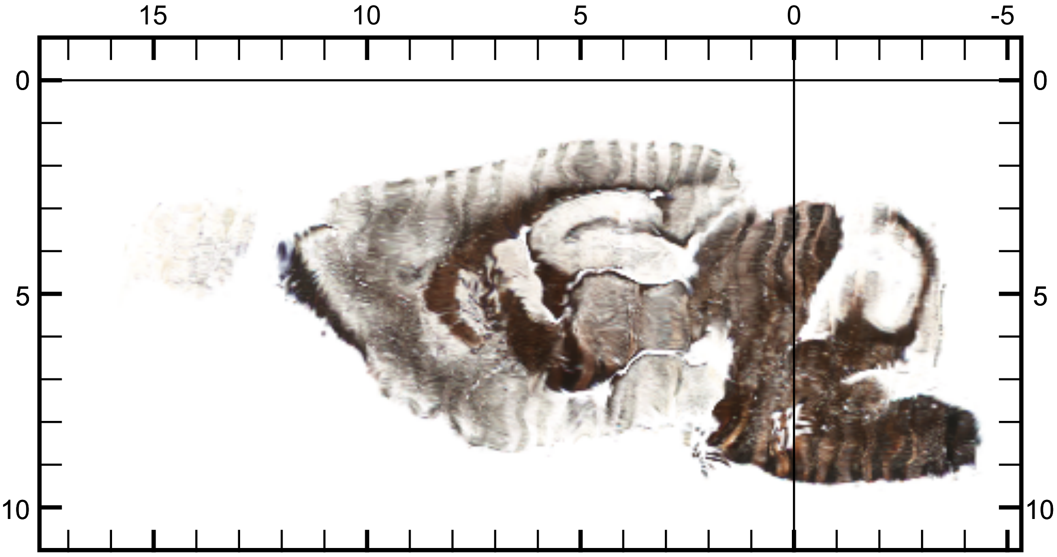

myelin

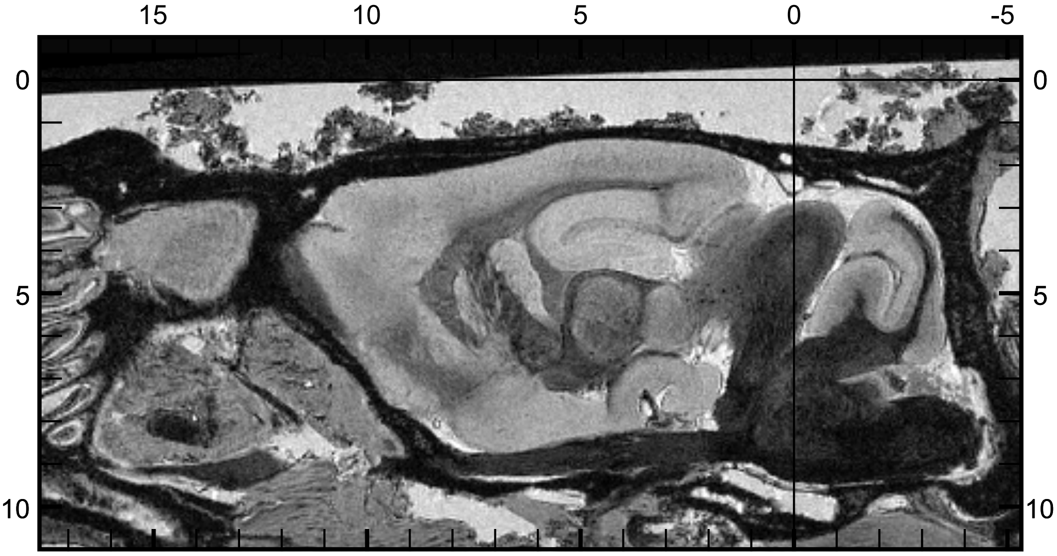

post mortem MR, 30 days

Sagittal cross-section 20 (continued)
2.5 mm left from midline

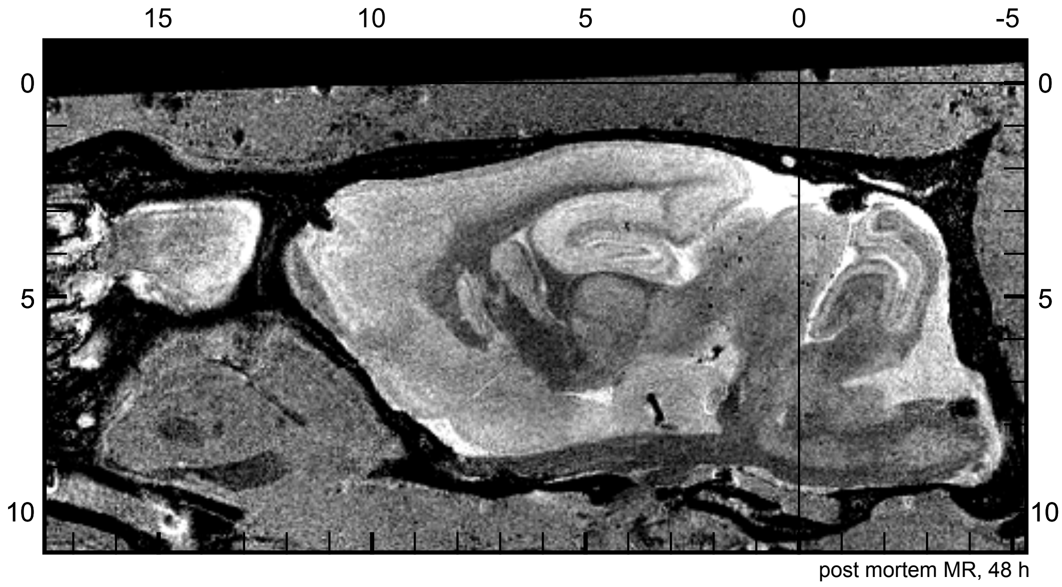

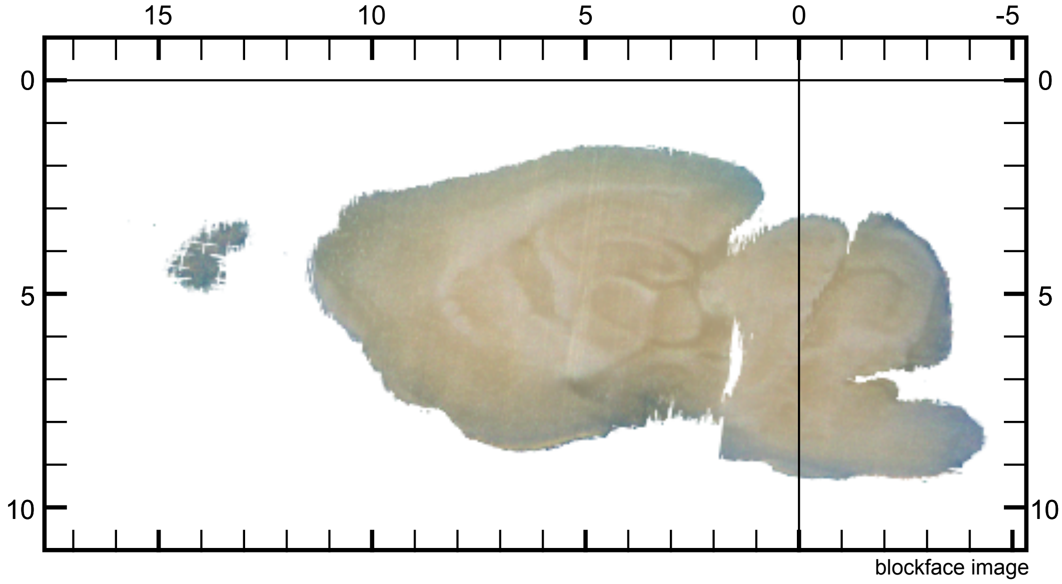

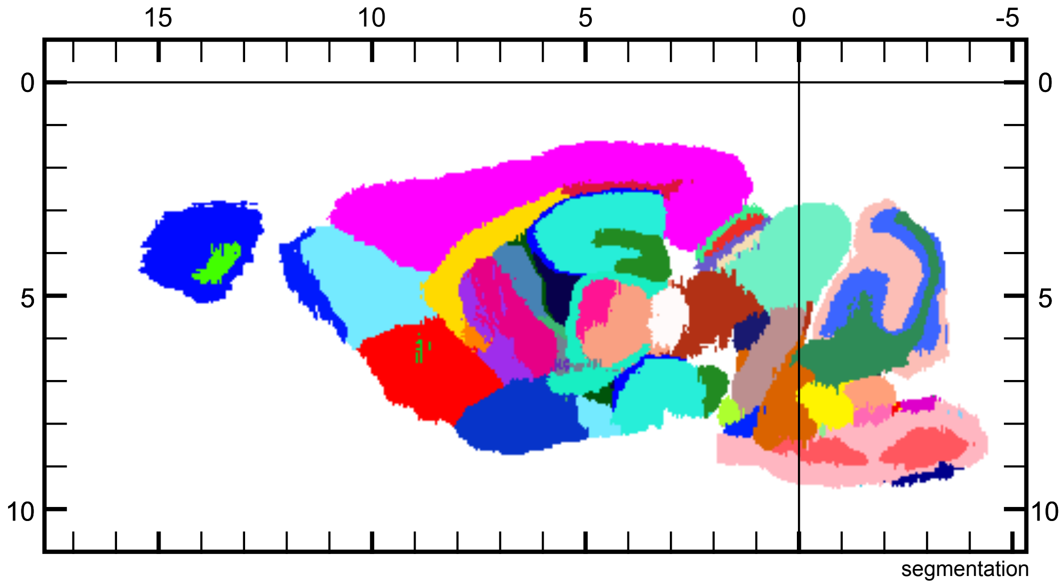

Sagittal cross-section 21
3.0 mm left from midline

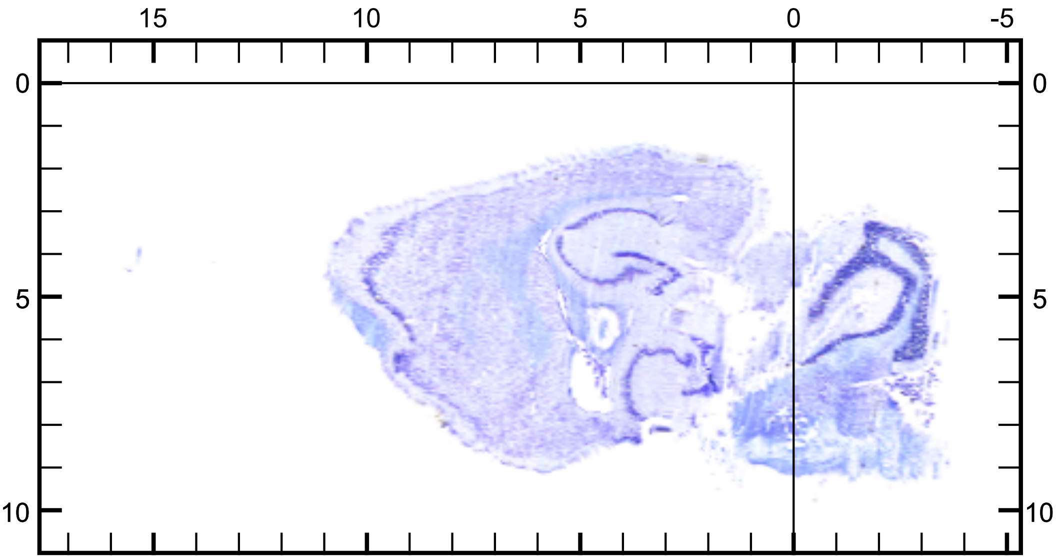

Nissl

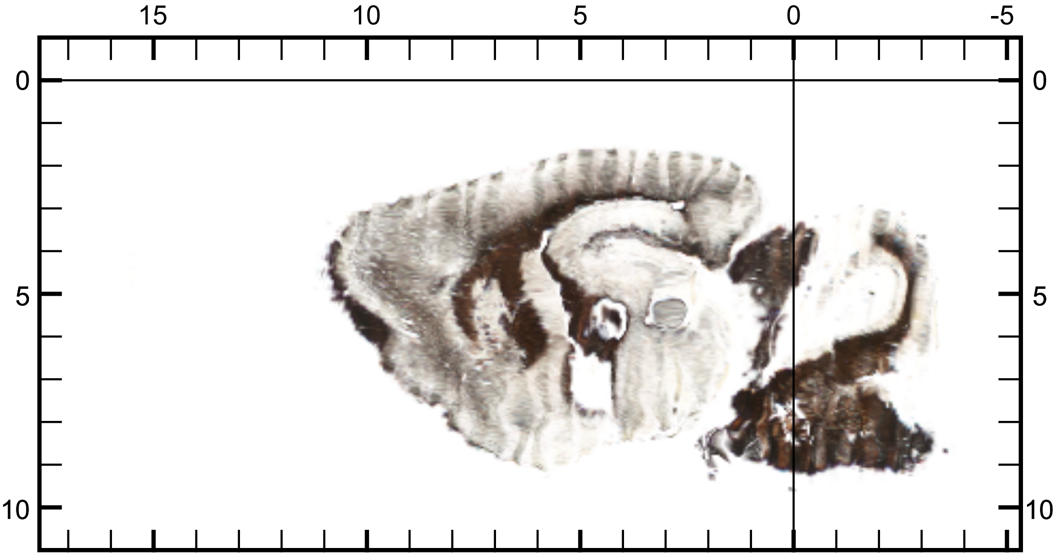

myelin

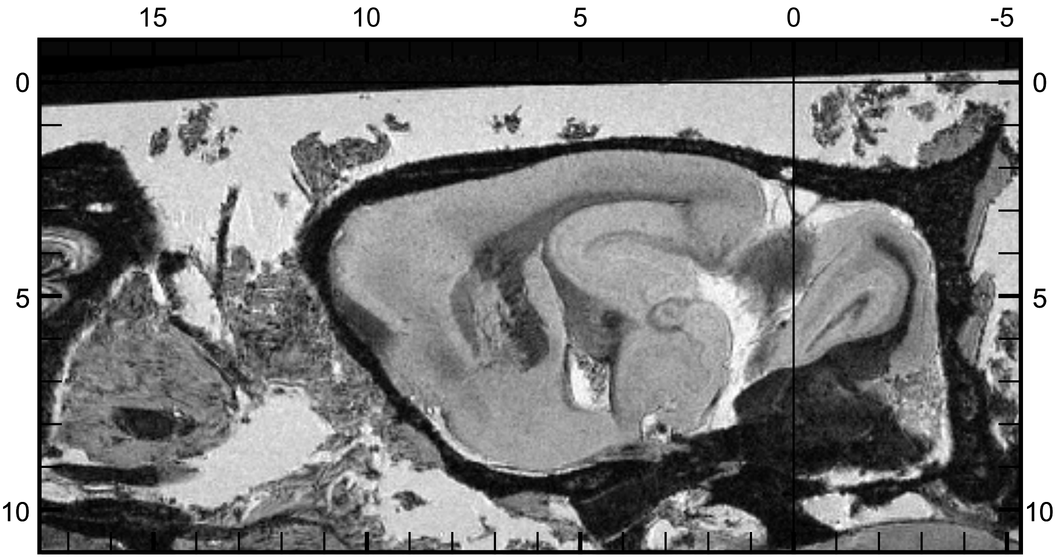

post mortem MR, 30 days

Sagittal cross-section 21 (continued)
3.0 mm left from midline

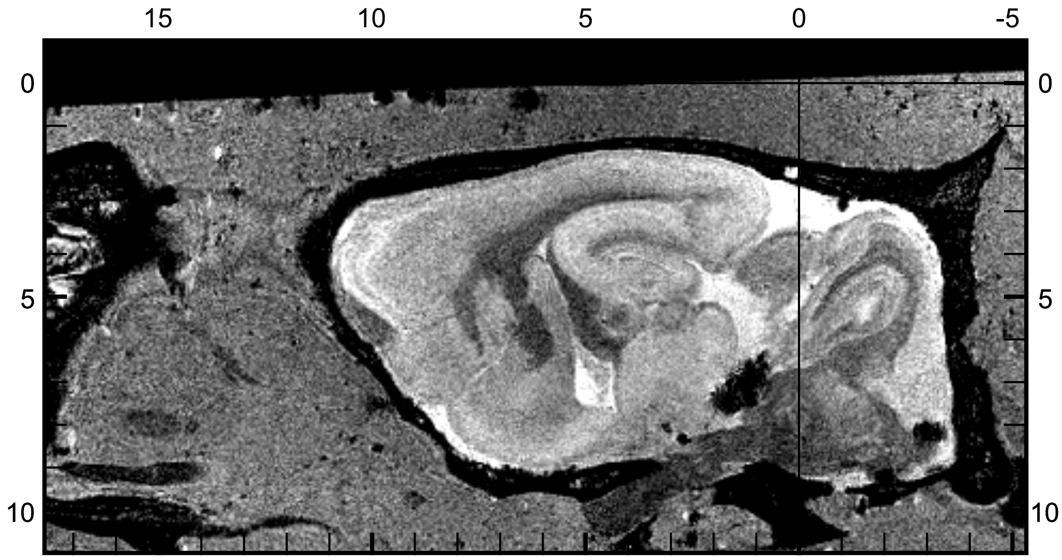

post mortem MR, 48 h

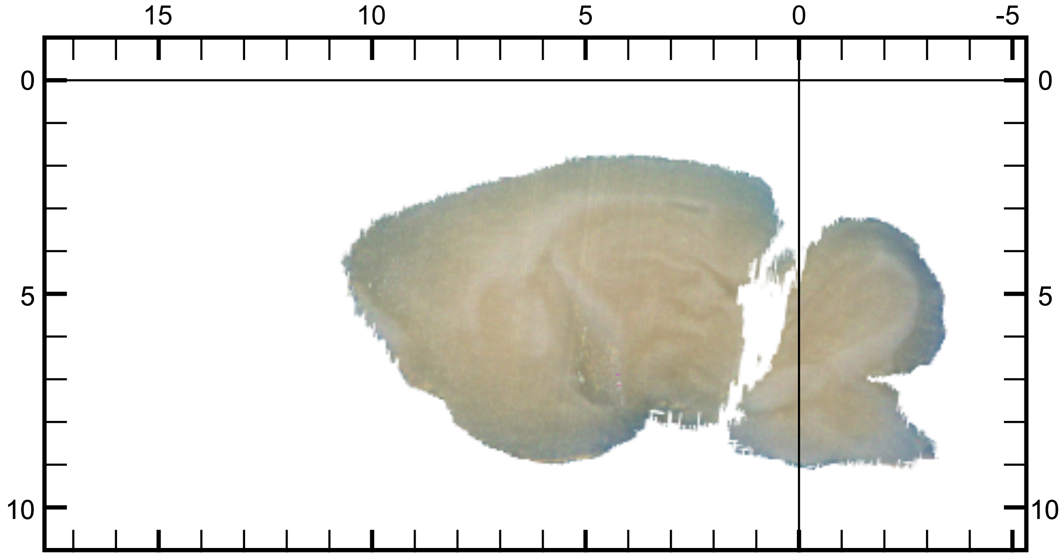

blockface image

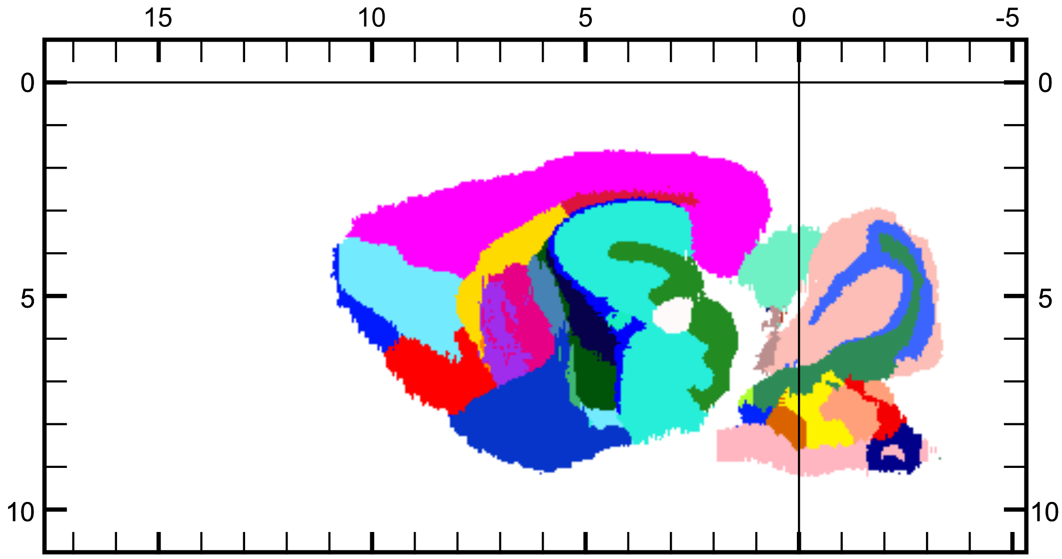

segmentation

Sagittal cross-section 22
3.5 mm left from midline

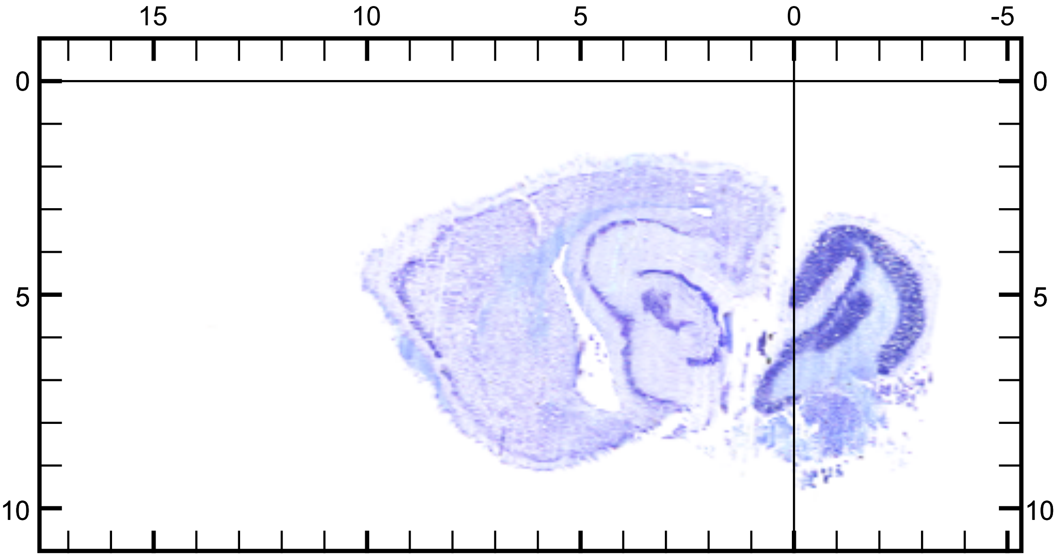

Nissl

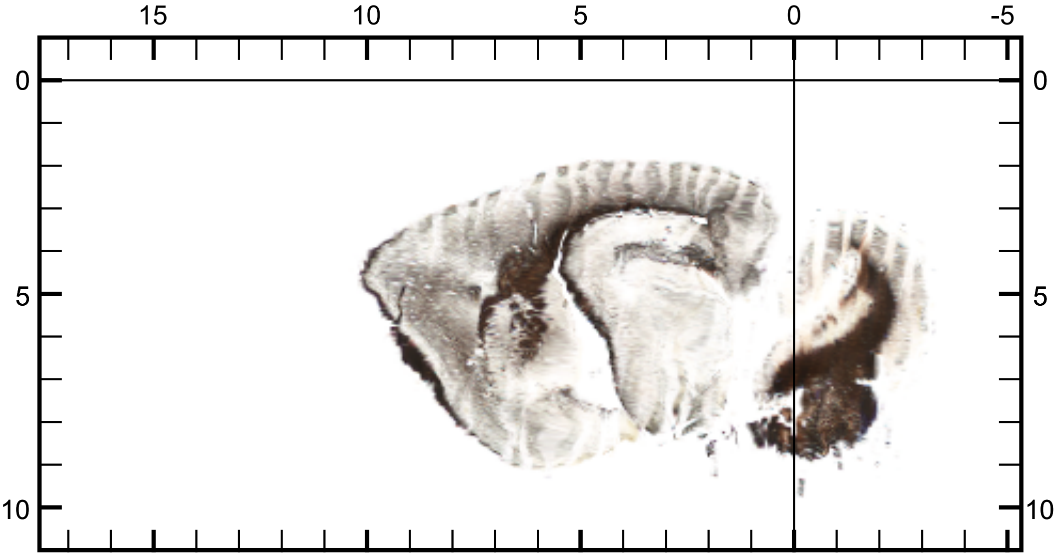

myelin

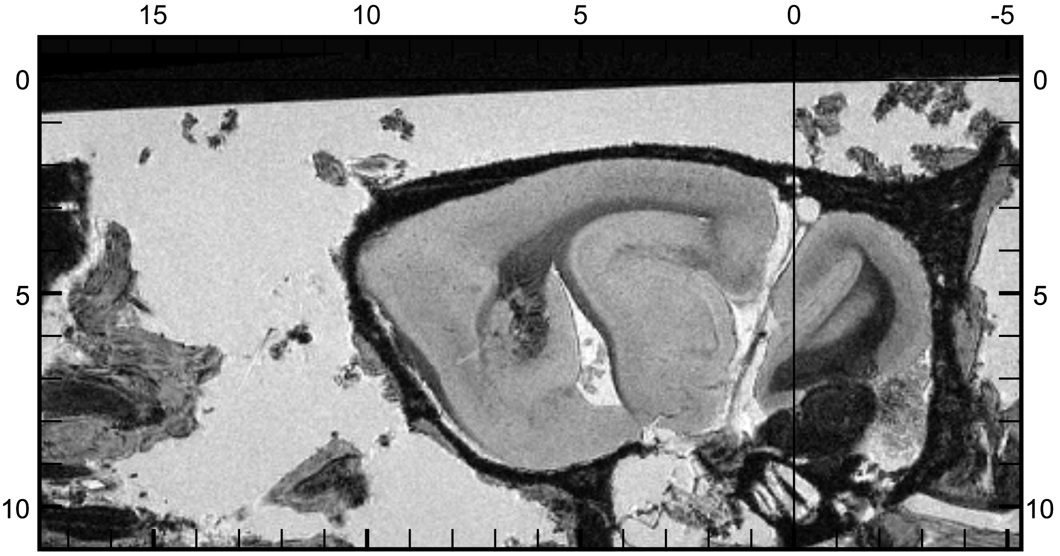

post mortem MR, 30 days

Sagittal cross-section 22 (continued)
3.5 mm left from midline

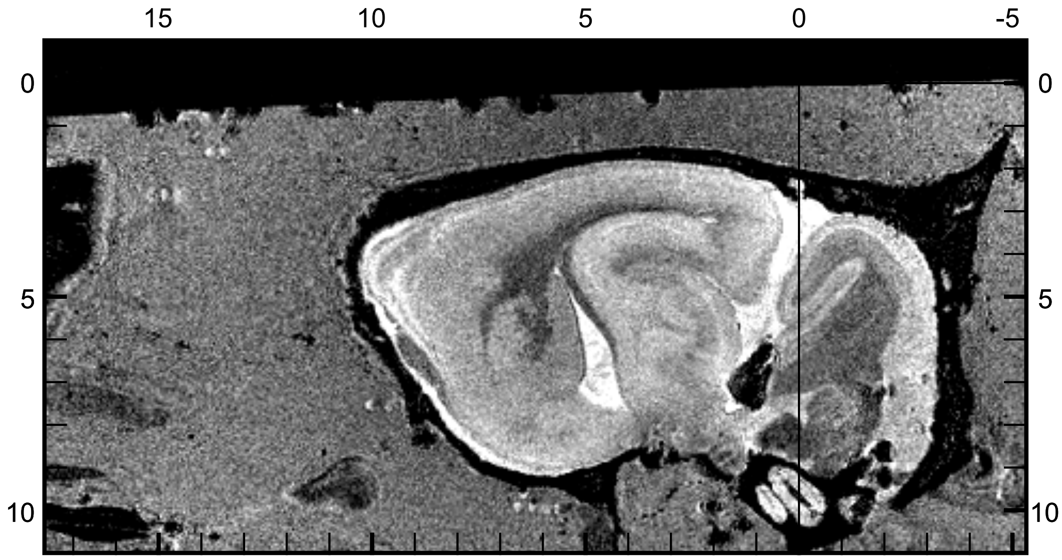

post mortem MR, 48 h

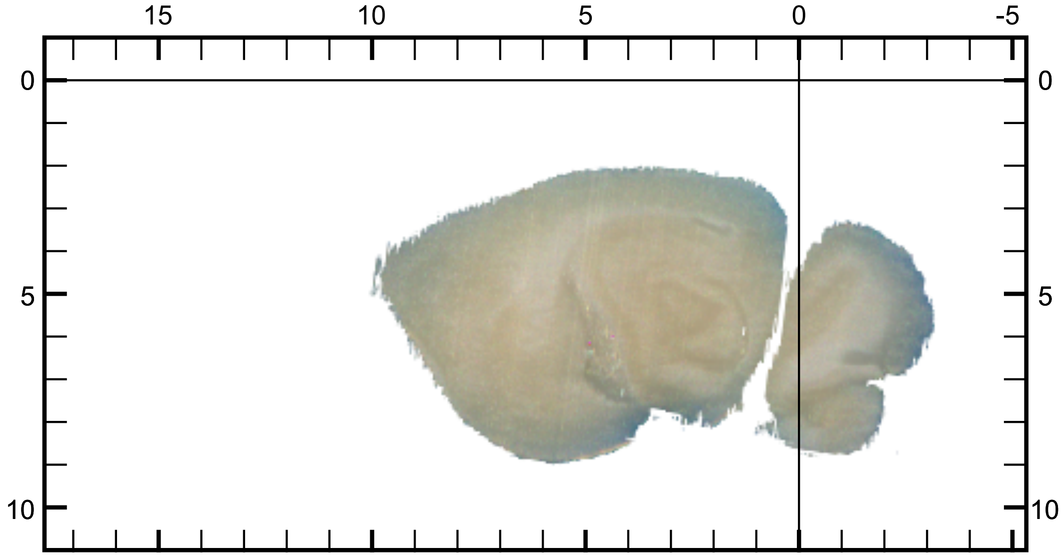

blockface image

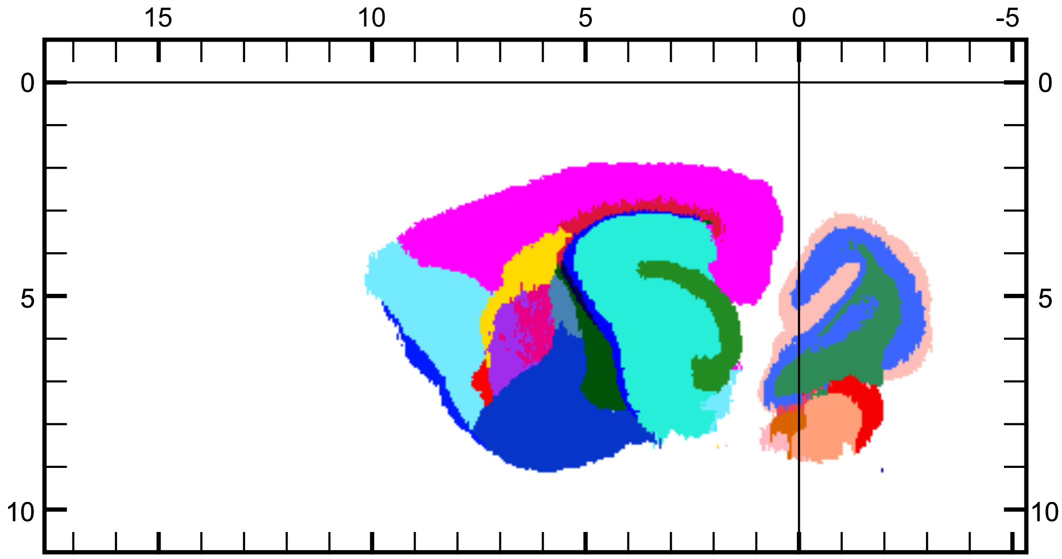

segmentation

Sagittal cross-section 23
4.0 mm left from midline

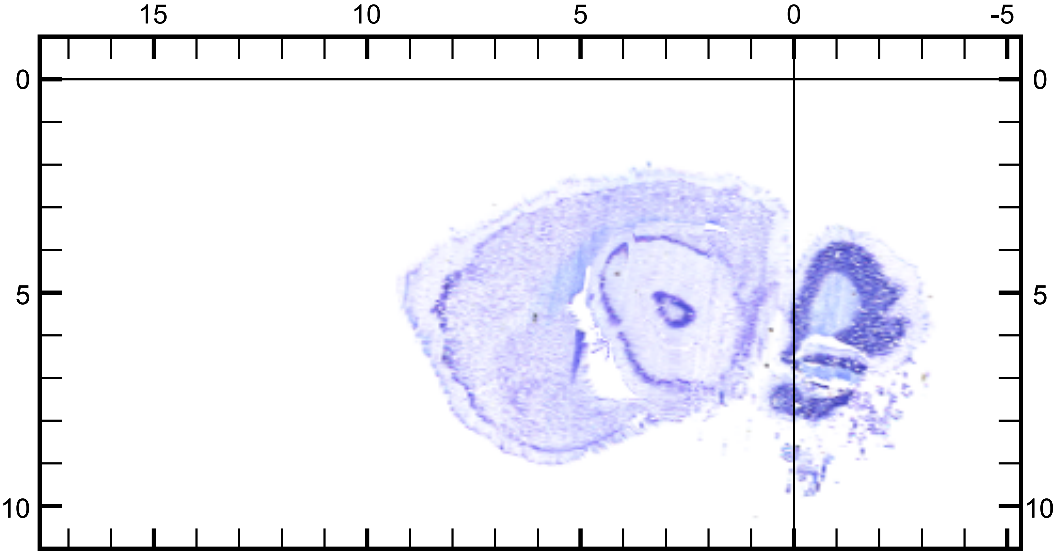

Nissl

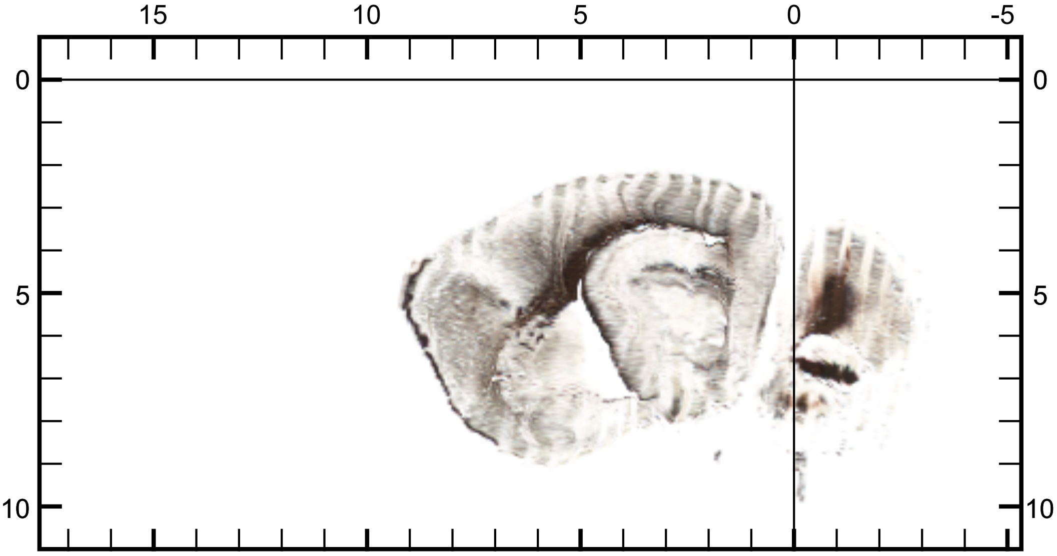

myelin

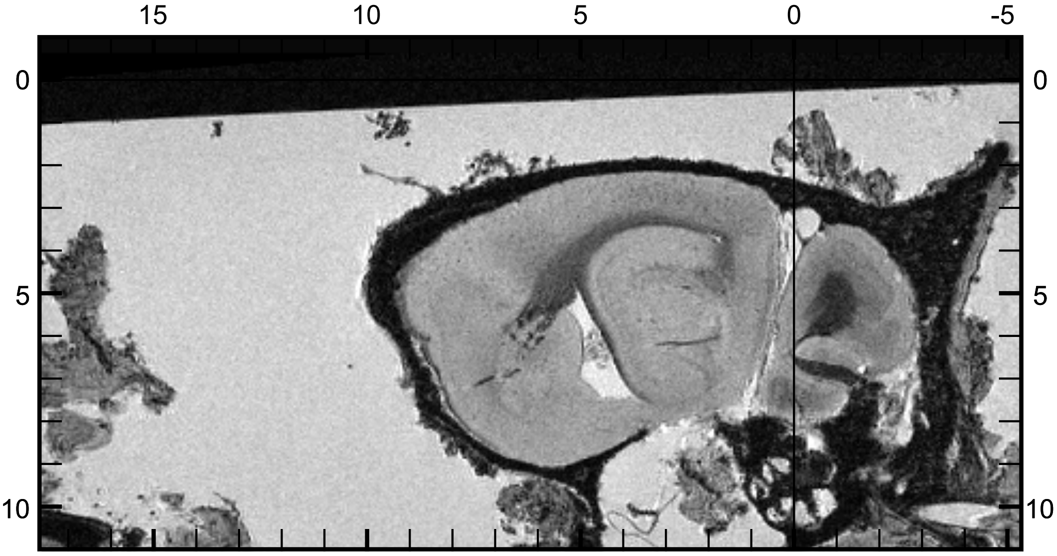

post mortem MR, 30 days

Sagittal cross-section 23 (continued)
4.0 mm left from midline

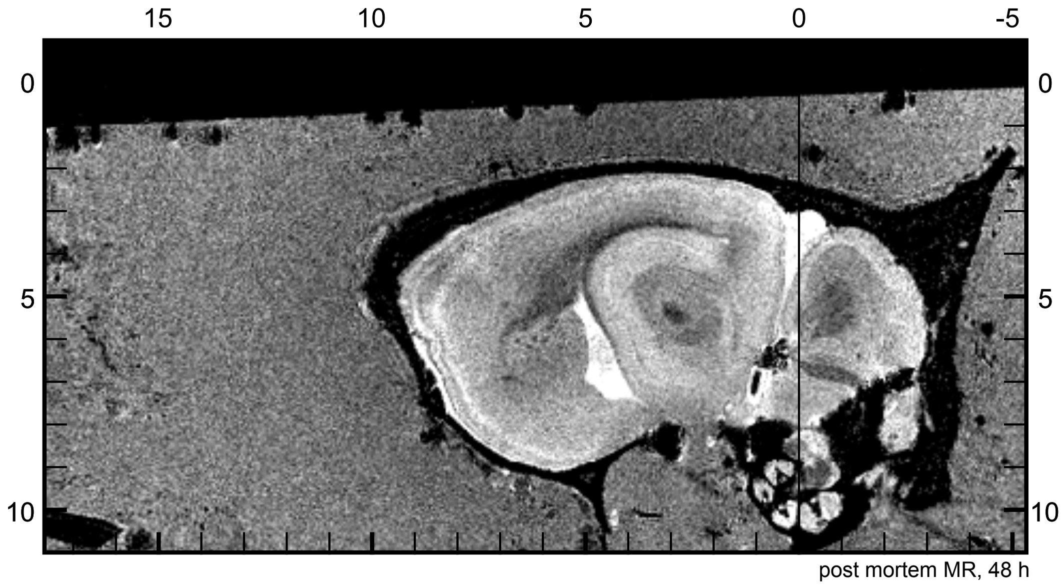

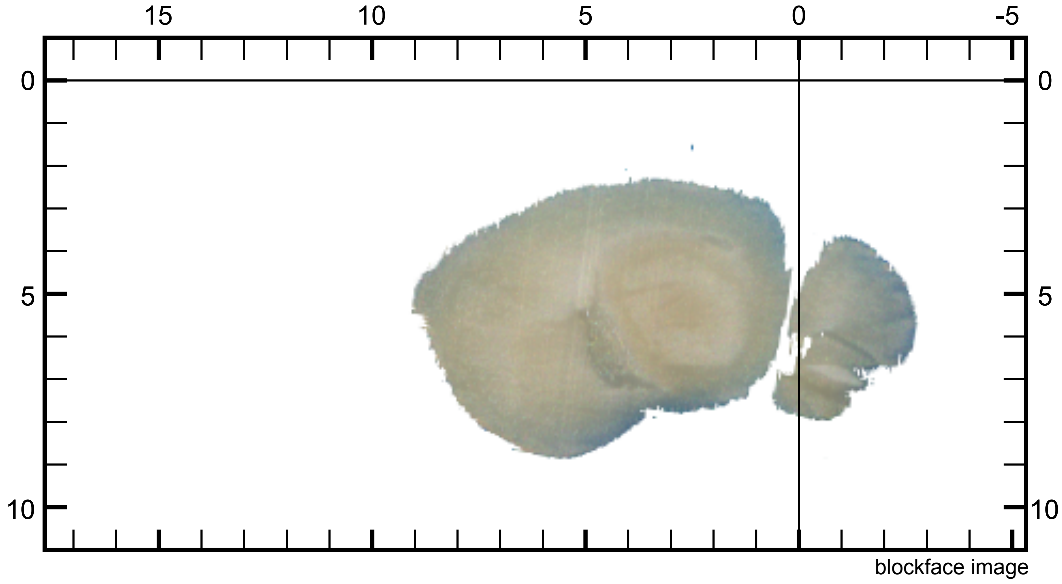

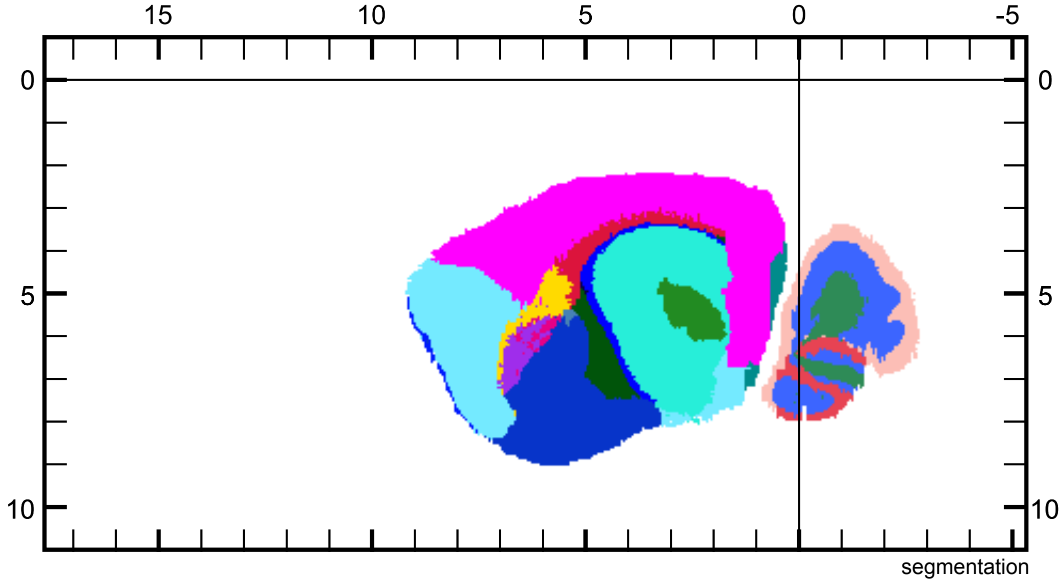

Sagittal cross-section 24
4.5 mm left from midline

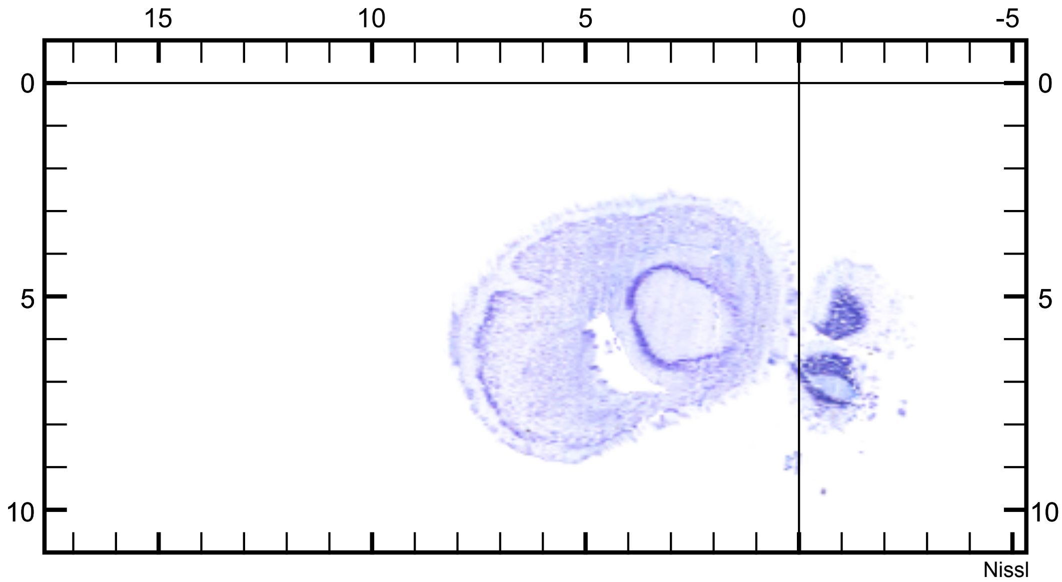

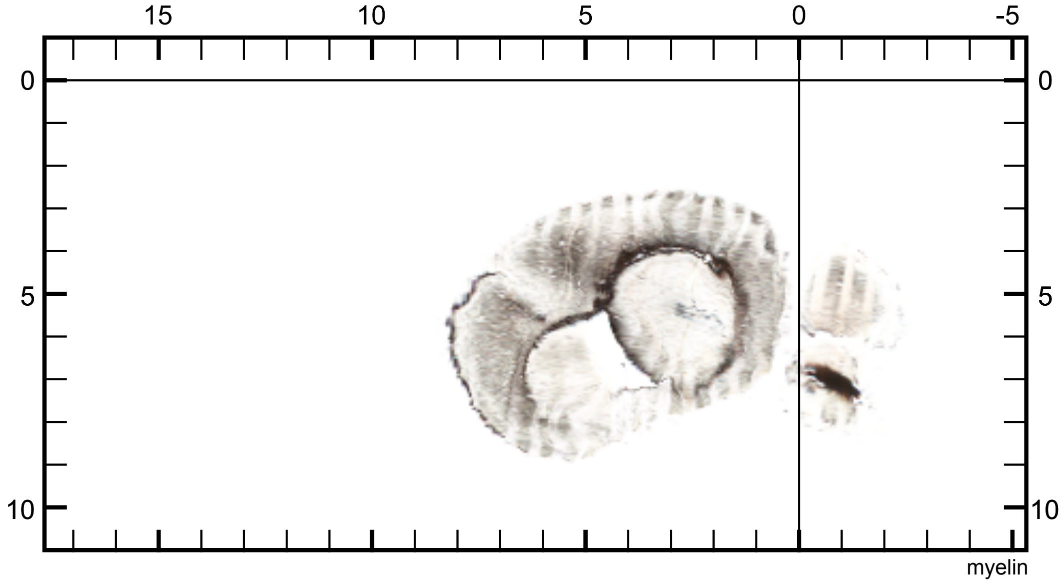

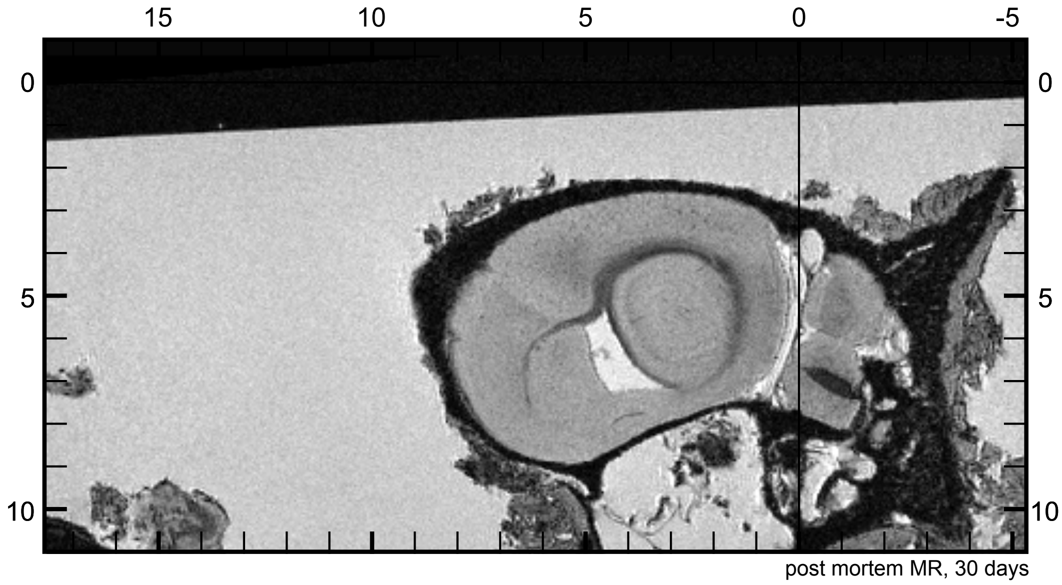

Sagittal cross-section 24 (continued)
4.5 mm left from midline

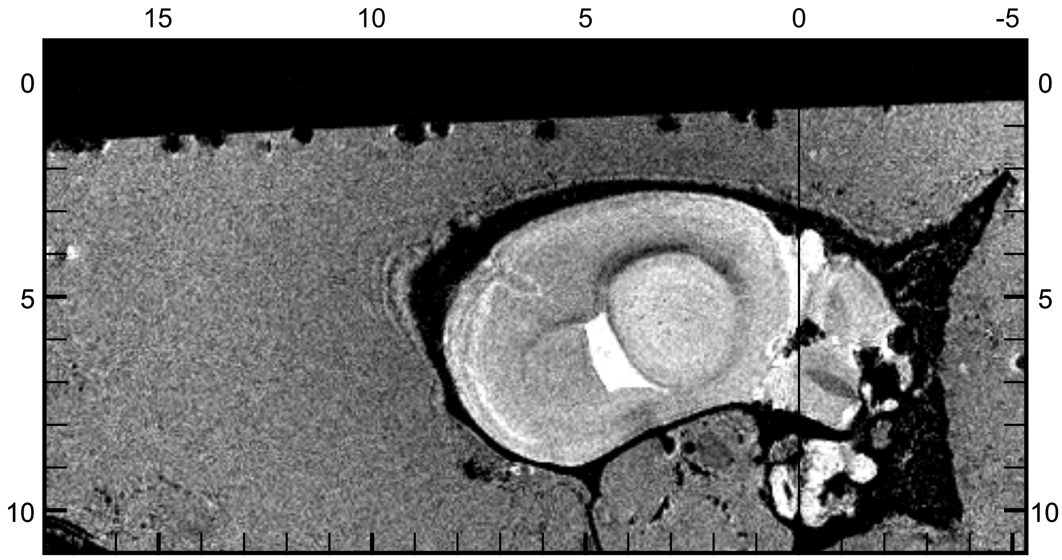

post mortem MR, 48 h

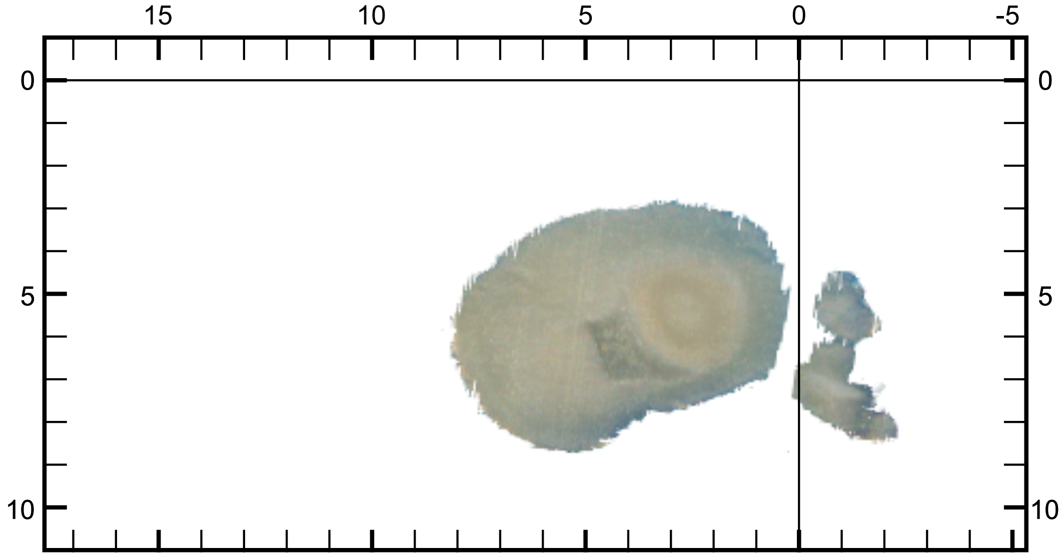

blockface image

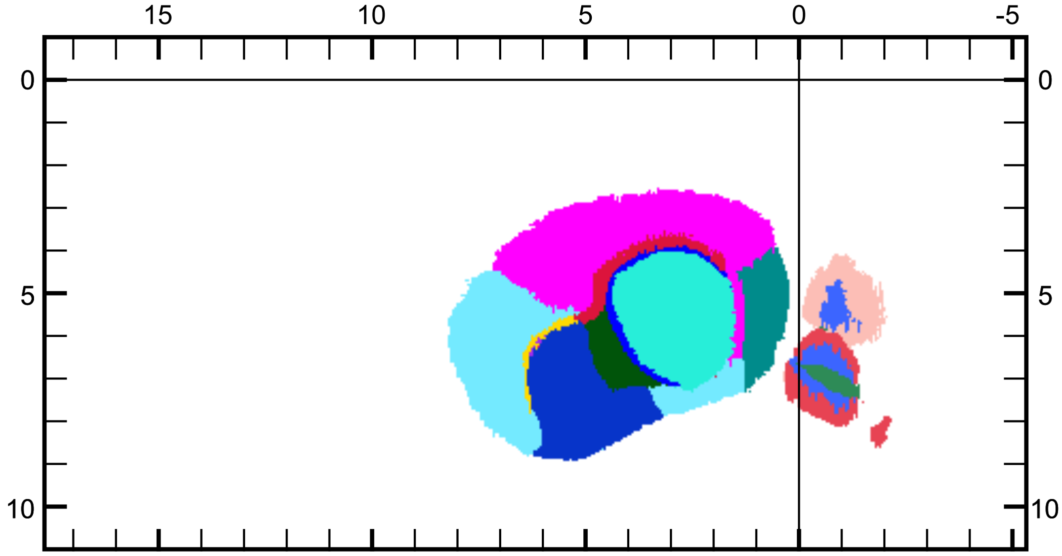

segmentation

Sagittal cross-section 25
5.0 mm left from midline

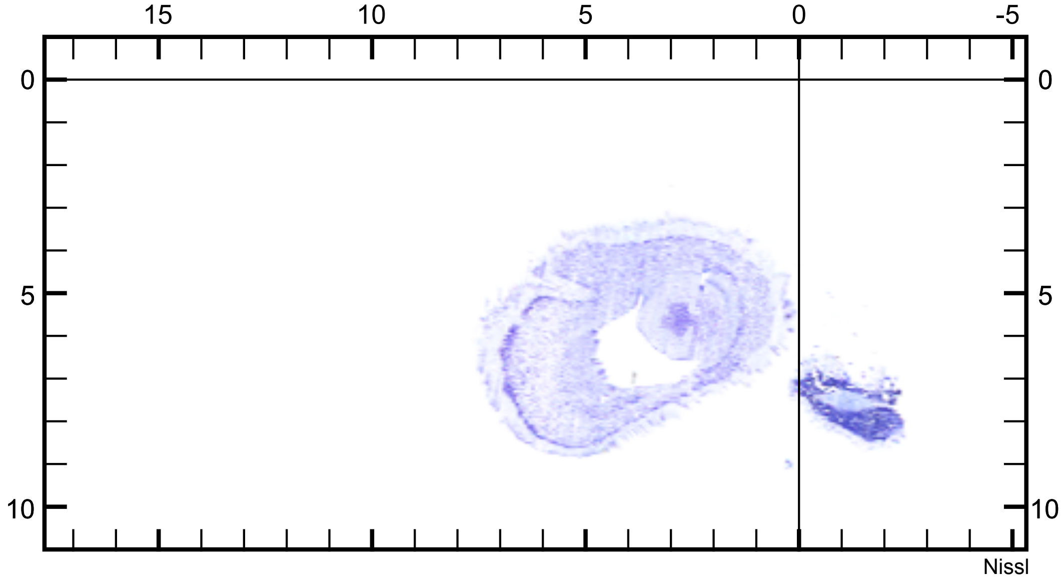

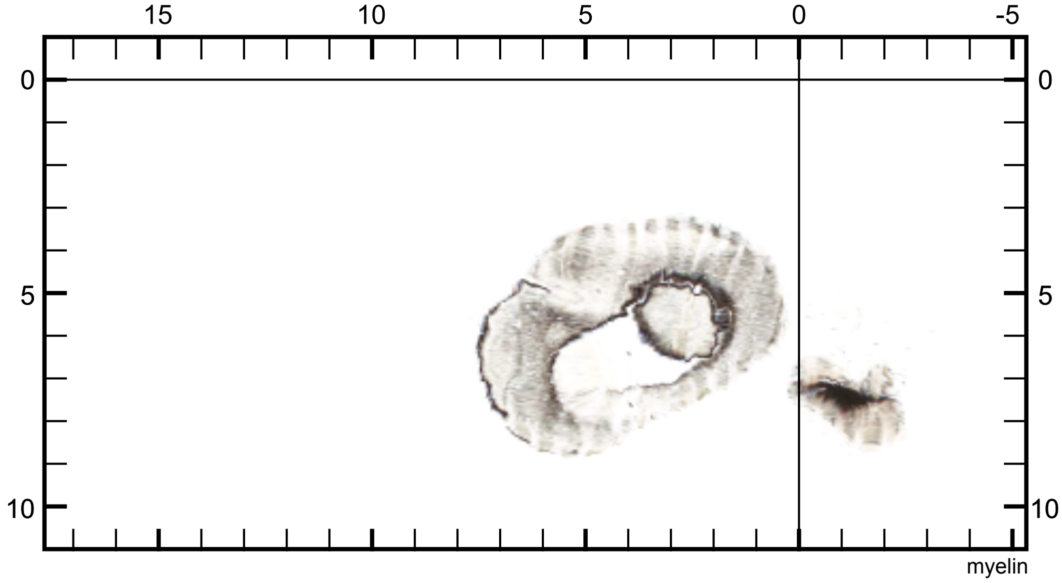

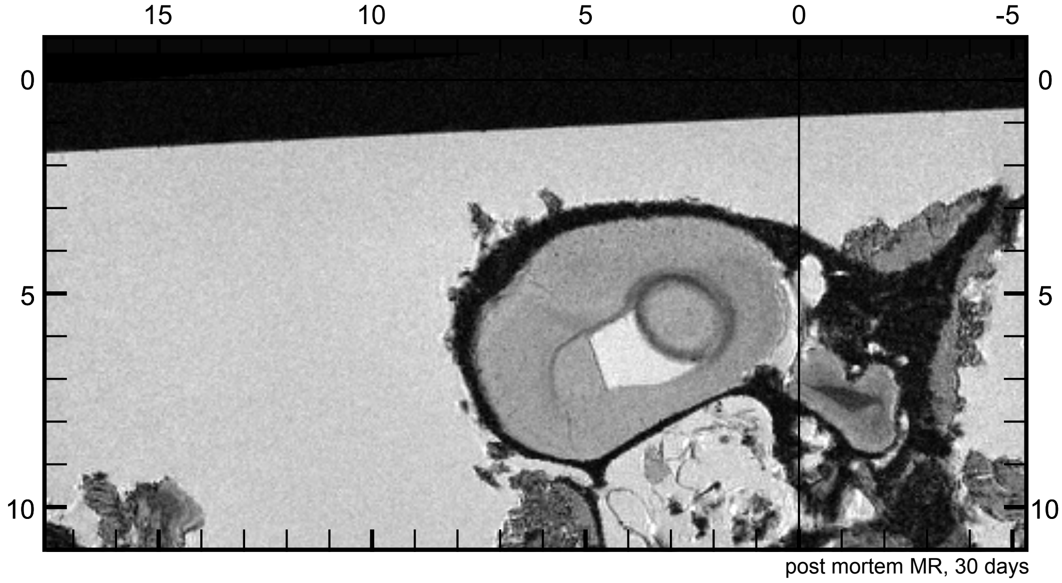

Sagittal cross-section 25 (continued)
5.0 mm left from midline

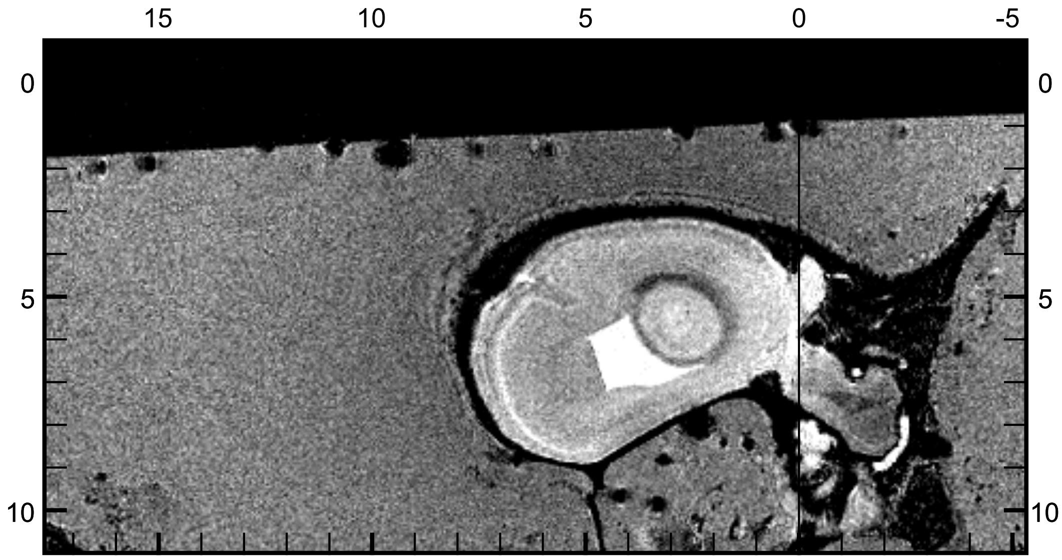

post mortem MR, 48 h

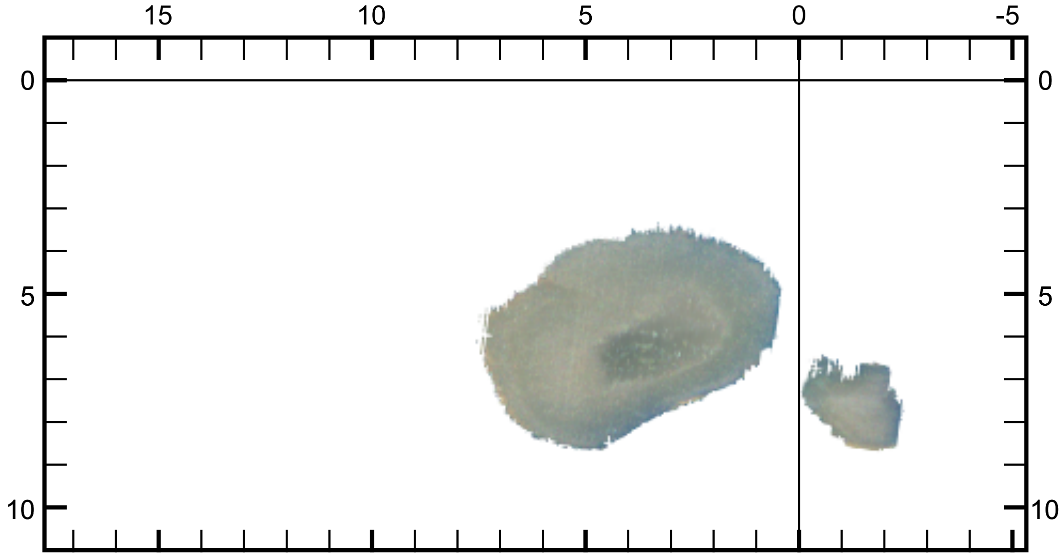

blockface image

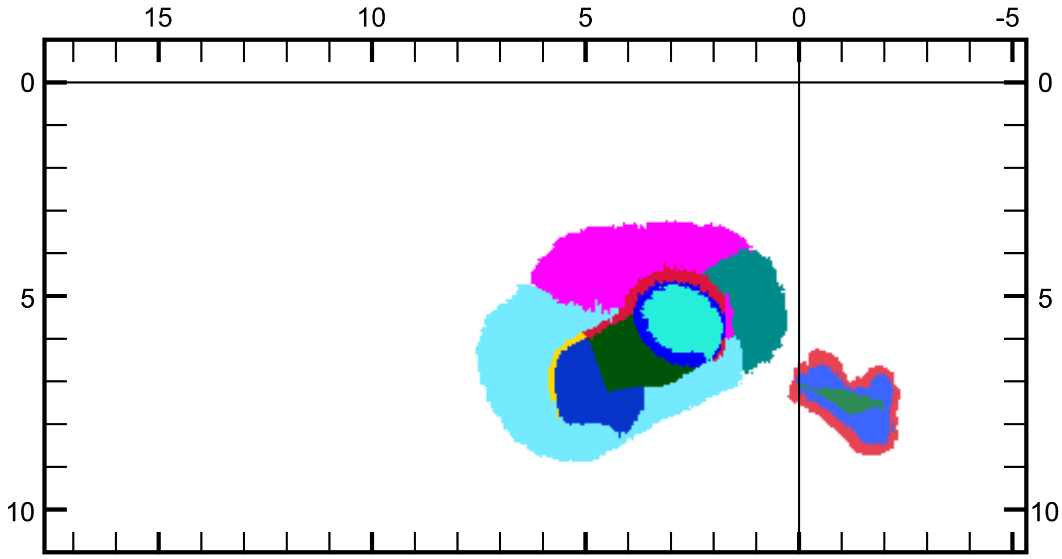

segmentation

Sagittal cross-section 26
5.5 mm left from midline

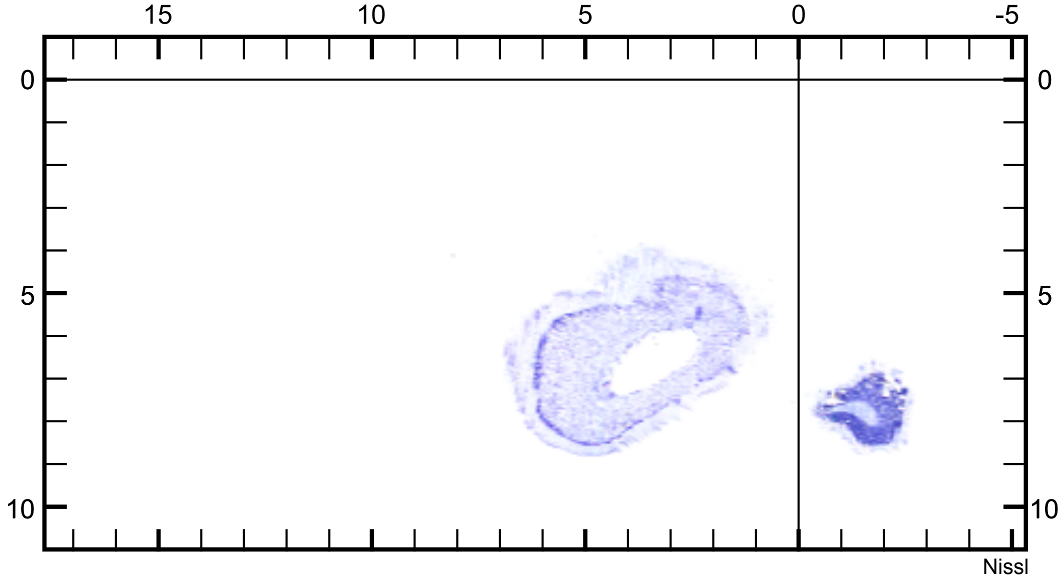

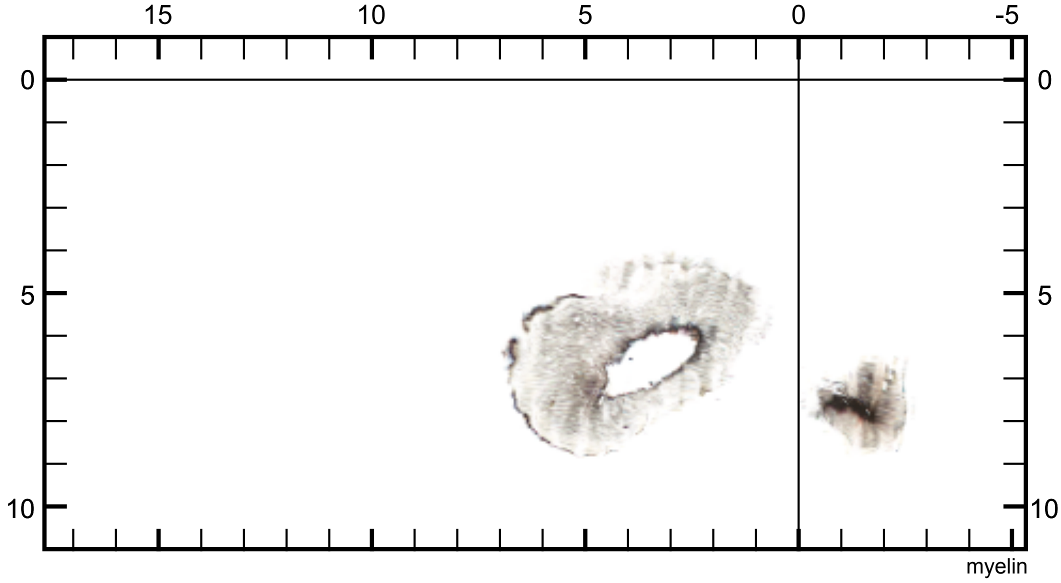

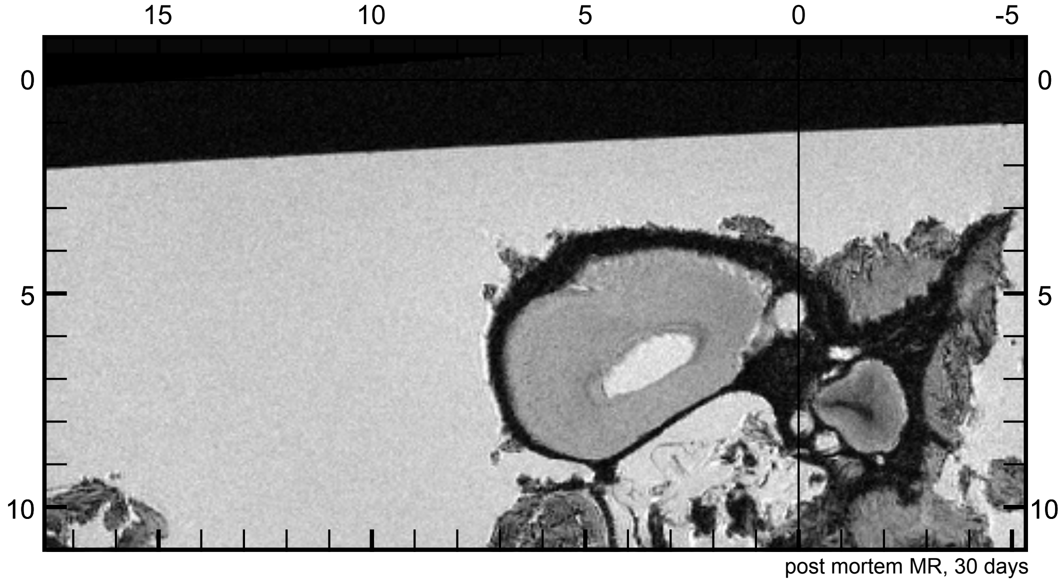

Sagittal cross-section 26 (continued)
5.5 mm left from midline

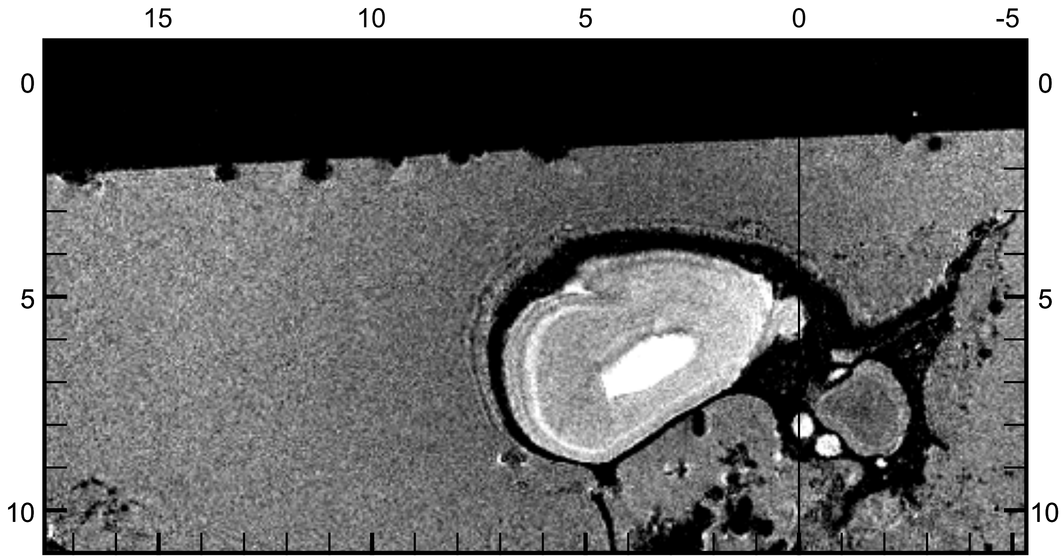

post mortem MR, 48 h

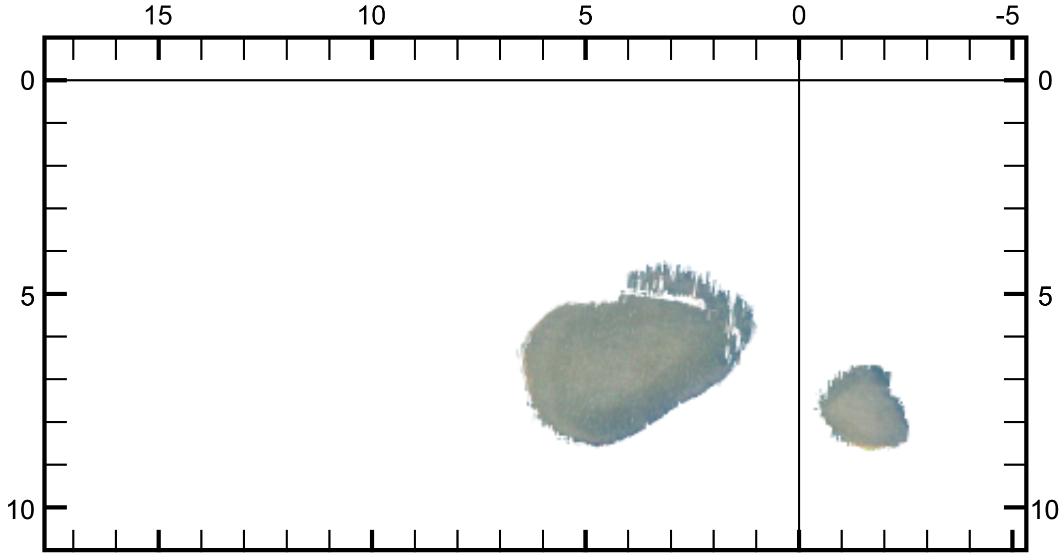

blockface image

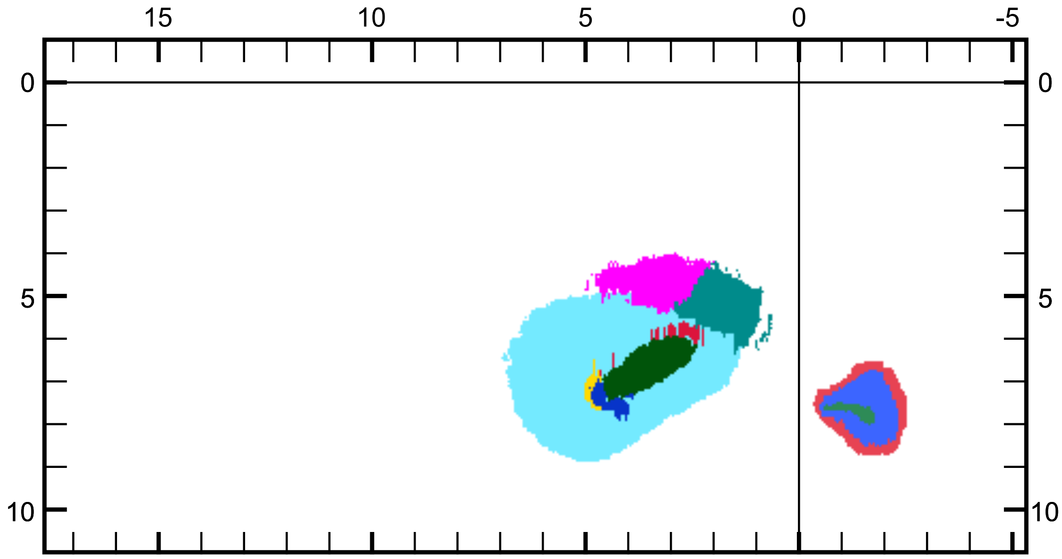

segmentation

Sagittal cross-section 27
6.0 mm left from midline

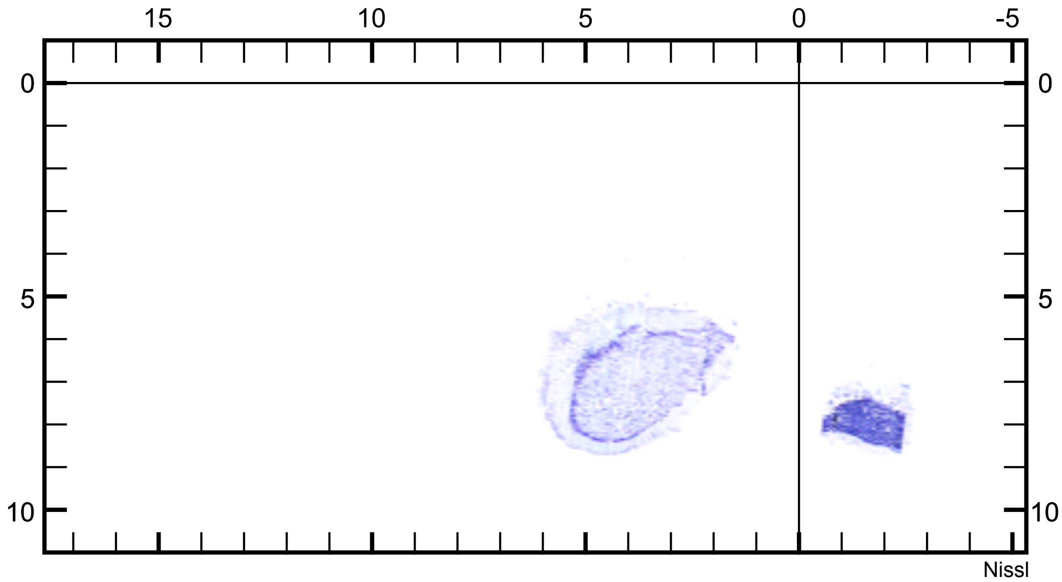

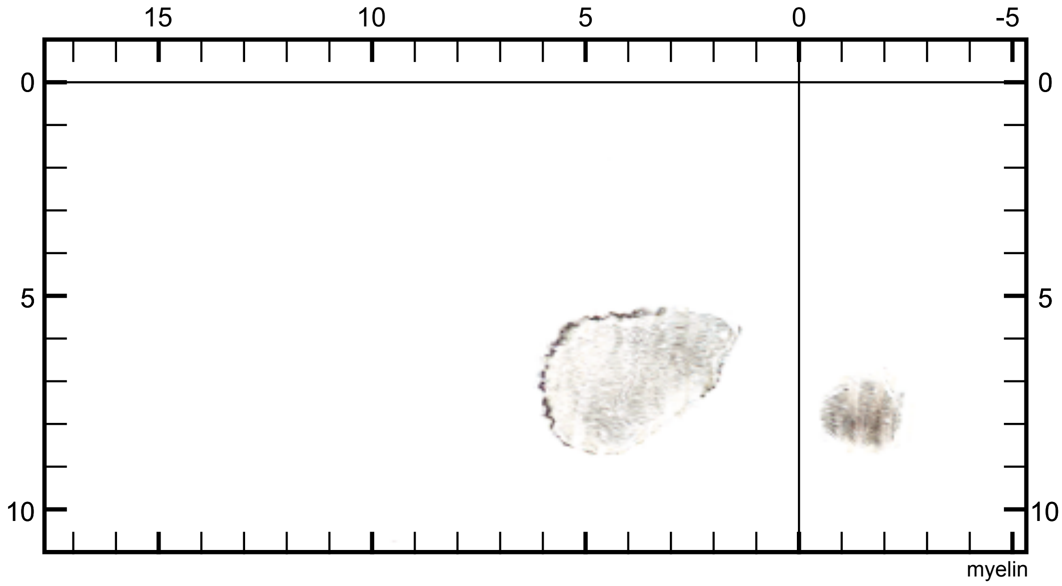

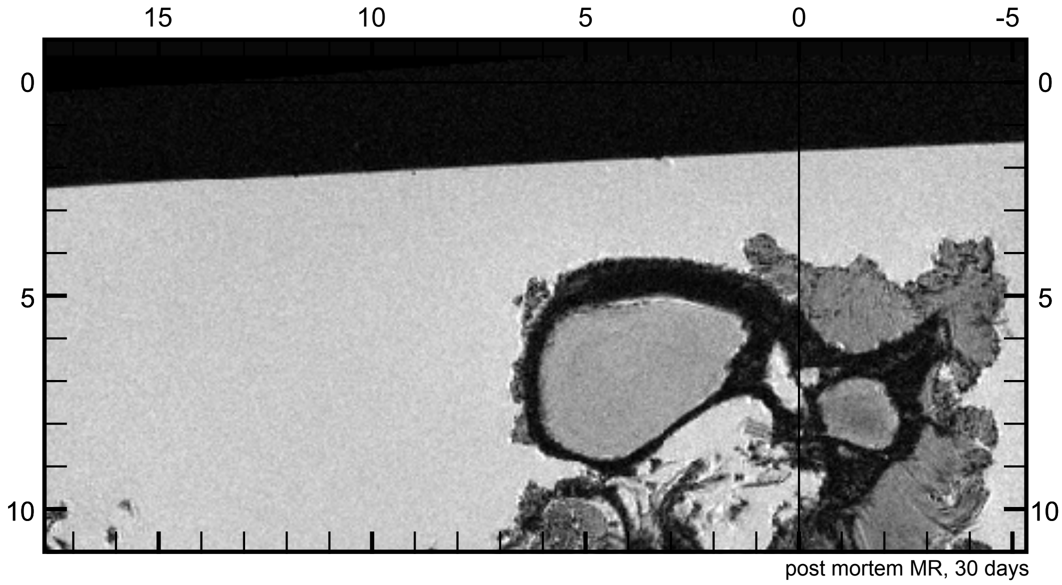

Sagittal cross-section 27 (continued)
6.0 mm left from midline

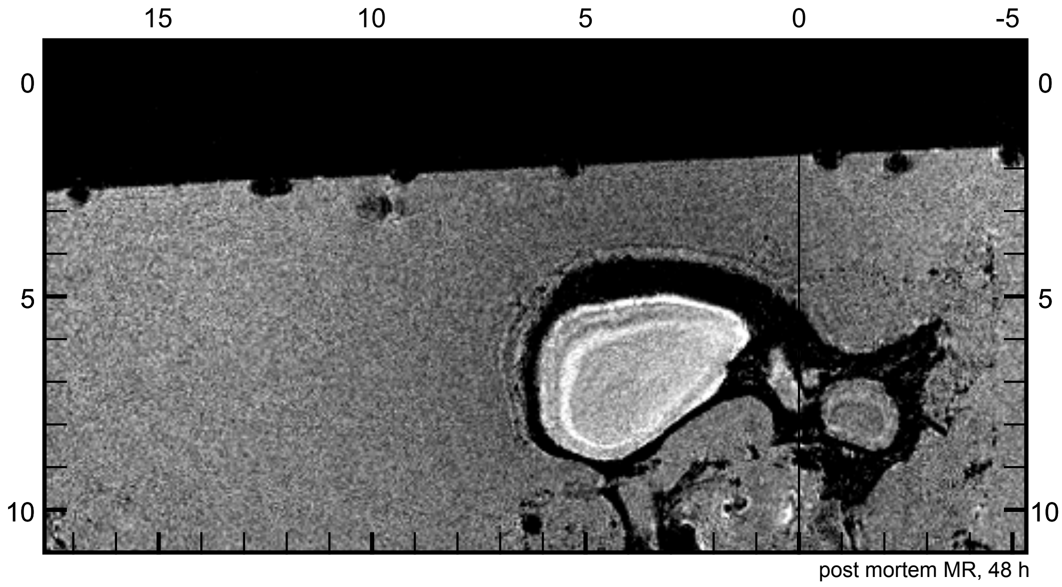

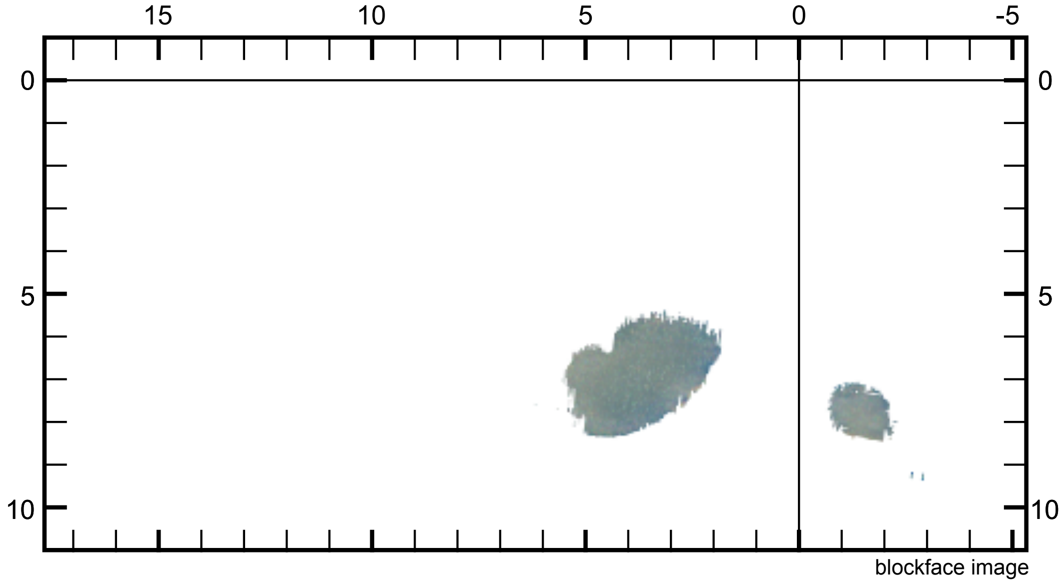

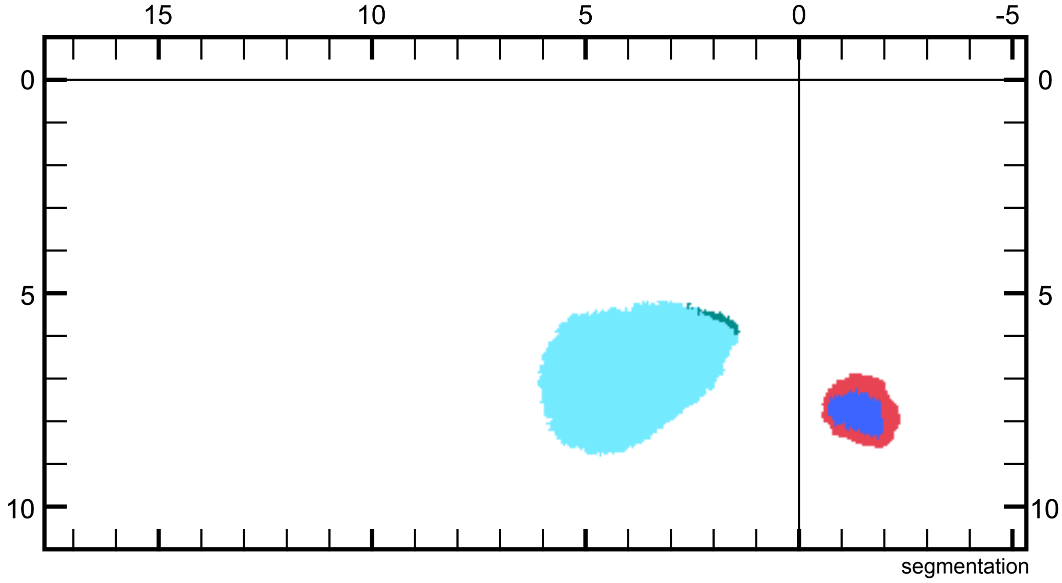

Sagittal cross-section 28
6.5 mm left from midline

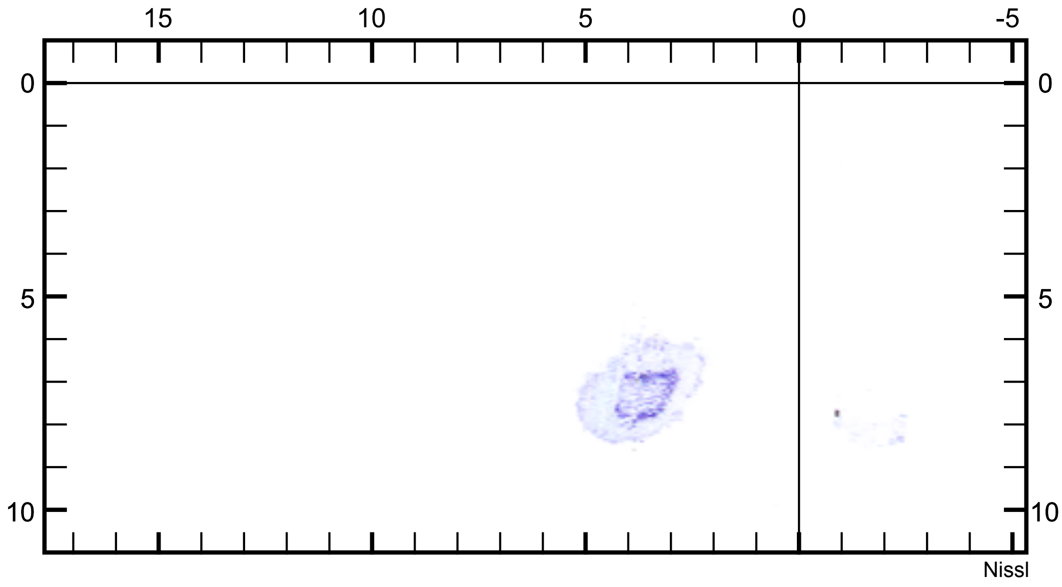

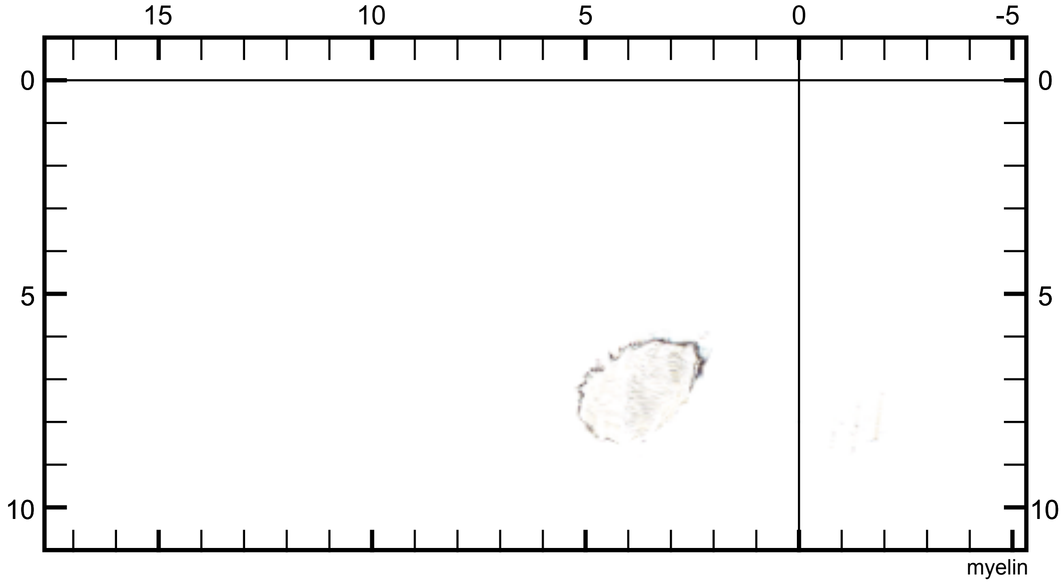

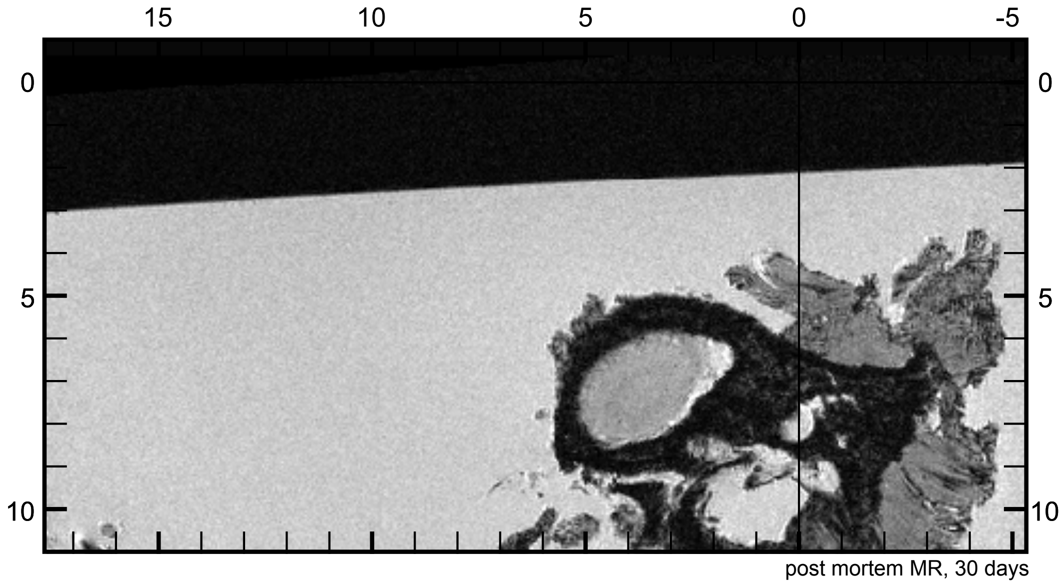

Sagittal cross-section 28 (continued)
6.5 mm left from midline

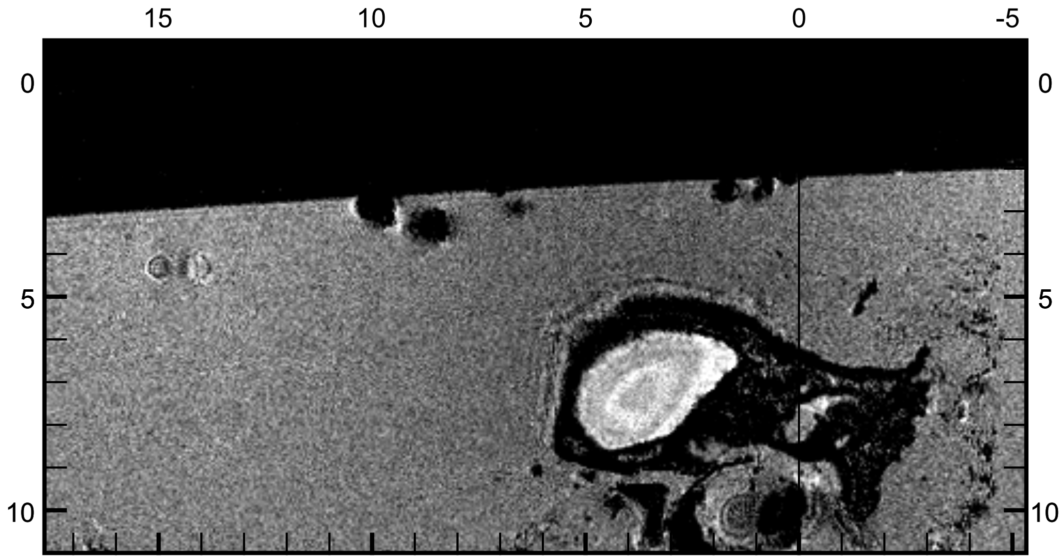

post mortem MR, 48 h

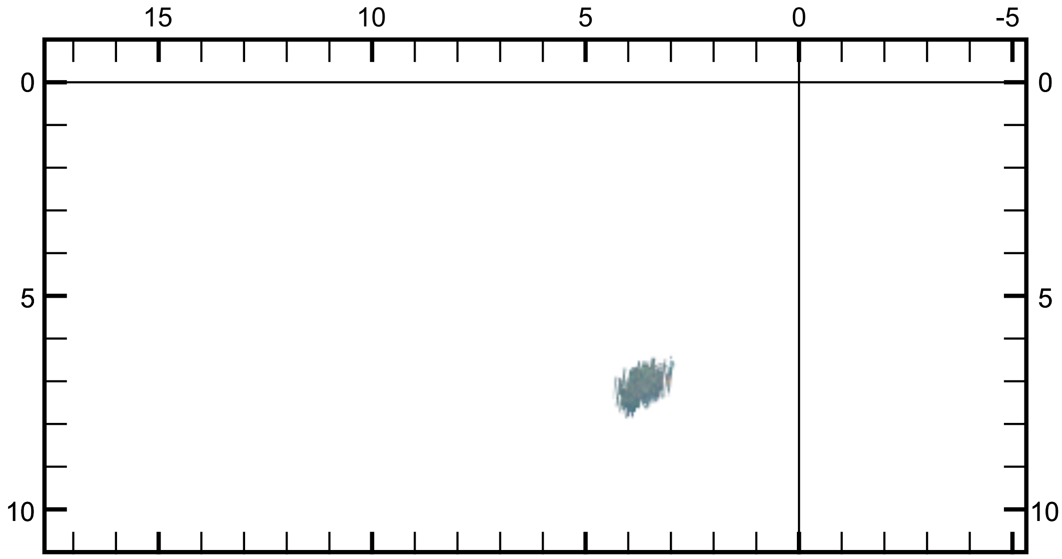

blockface image

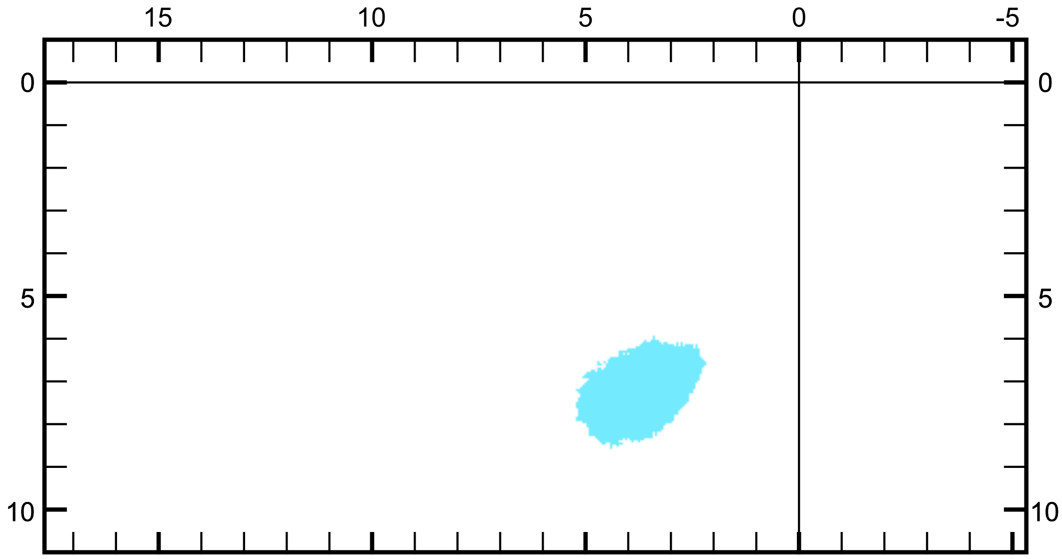

segmentation

Sagittal cross-section 29
7.0 mm left from midline

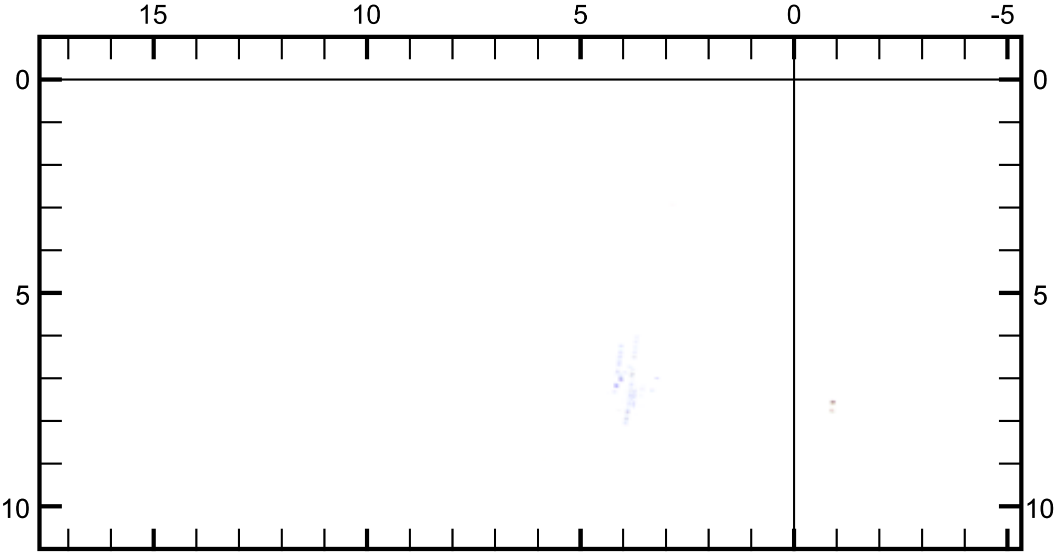

Nissl

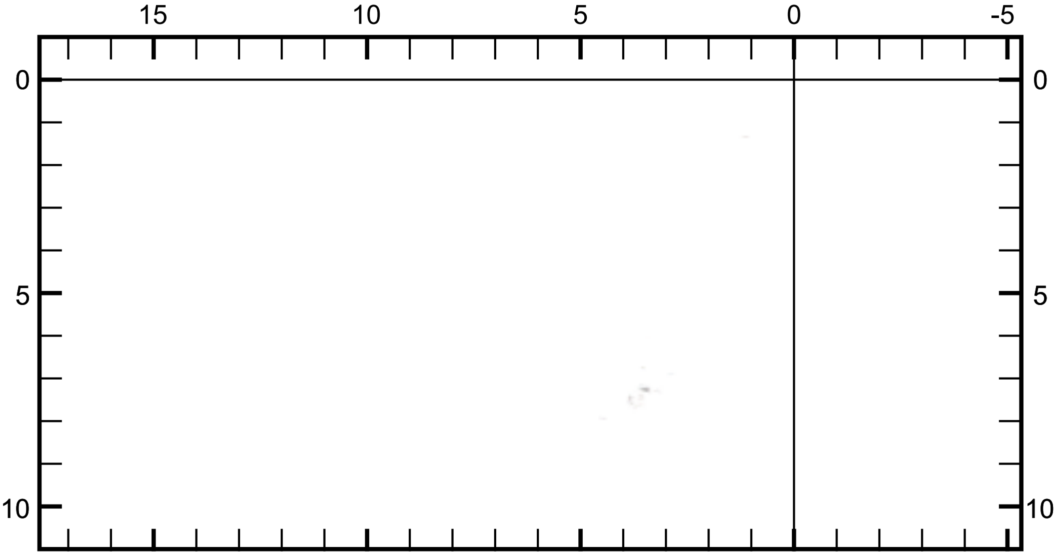

myelin

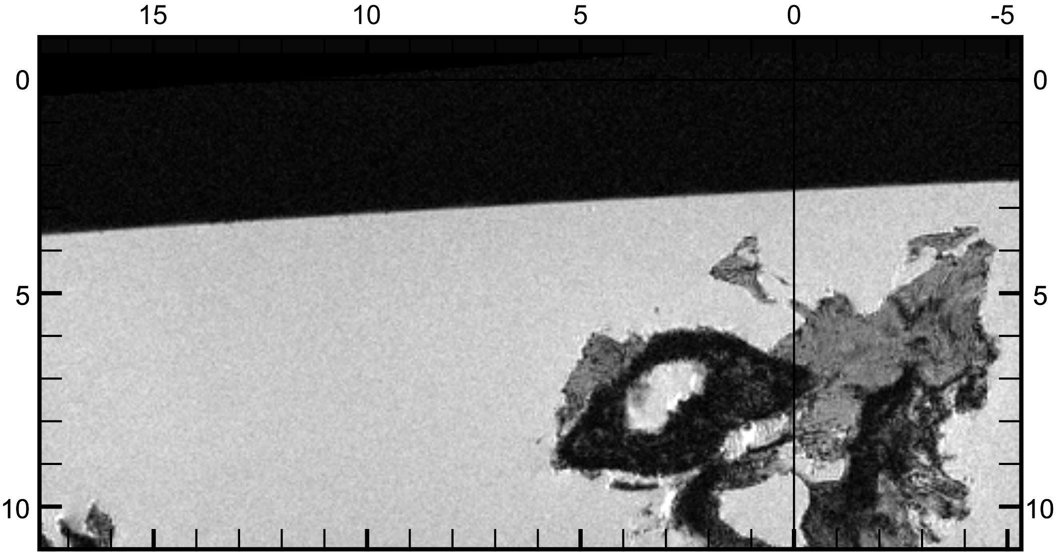

post mortem MR, 30 days

Sagittal cross-section 29 (continued)
7.0 mm left from midline

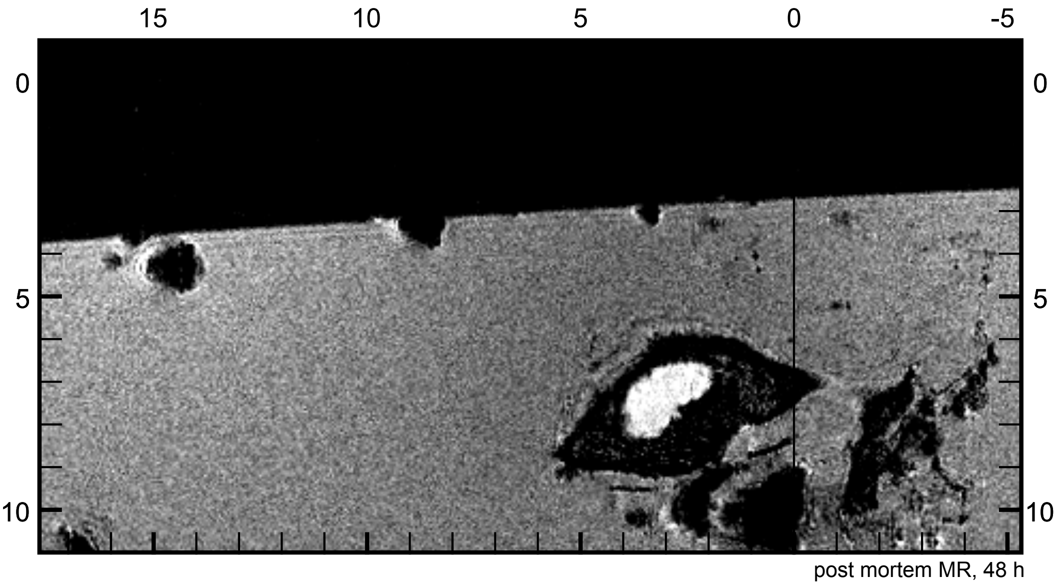

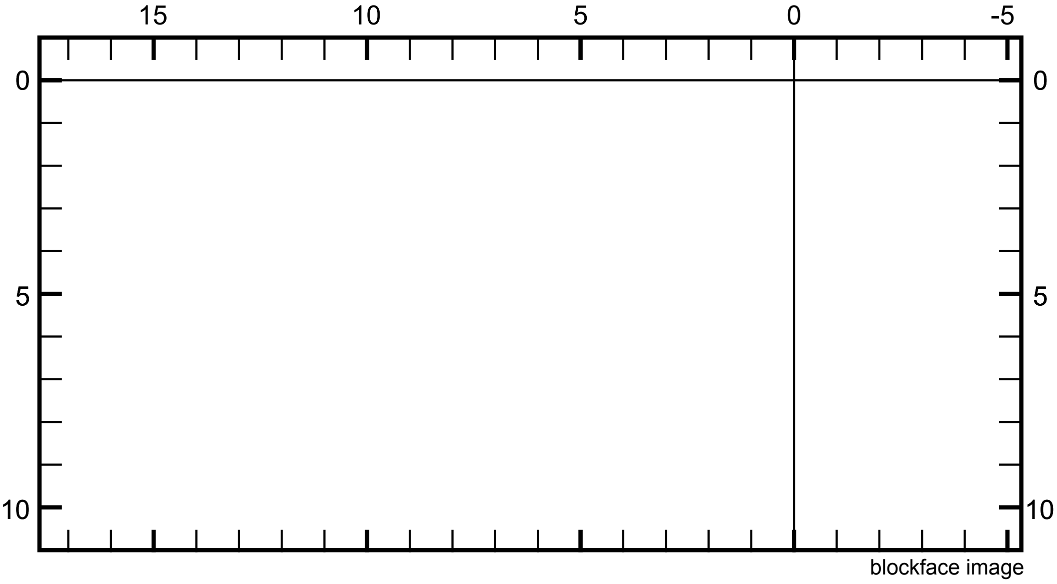

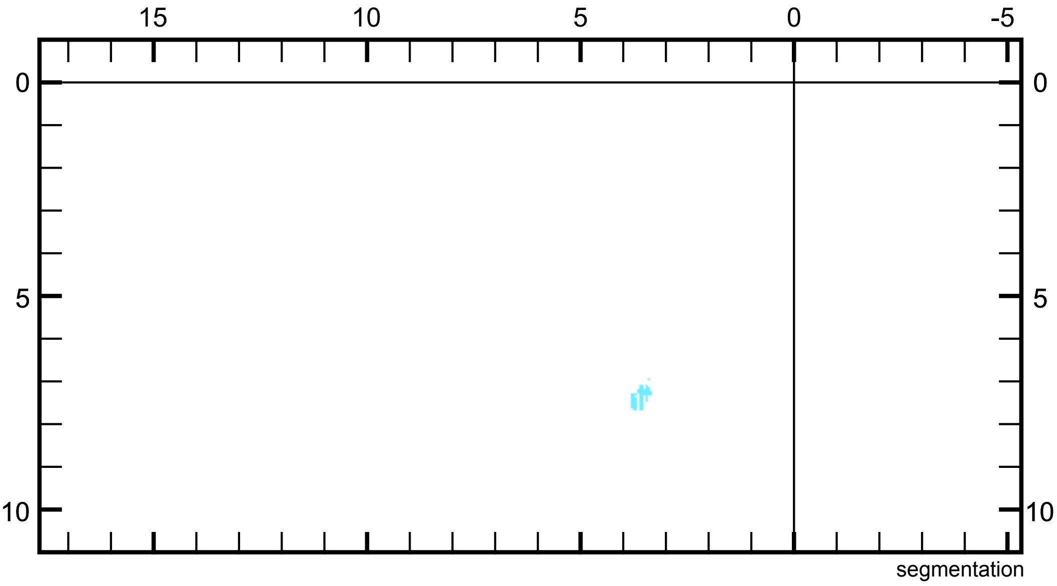

Structure	volume (mm³)	MRI 48h	MRI 30d	Nissl	Myelin
Brain *(Brain)*	993.0				
Forebrain *(FB)*	555.4				
Olfactory bulb *(OB)*	85.2				
Olfactory nerve layer *(ON)*	2.4	■	■	■	□
Glomerular layer *(Gl)*	26.0	■	■	■	□
External plexiform layer *(EPl)*	24.4	■	■	■	□
Granular cell layer *(GrO)*	32.4	■	■	■	□
Accessory olfactory bulb *(AOB)*	0.6	■	■	□	□
Neocortex *(NCtx)*	140.2	■	■	■	■
Piriform cortex *(Pir)*	91.3	■	■	■	□
Entorhinal cortex *(Ent)*	9.1	■	■	■	■
Hippocampus *(HIP)*	94.1				
Dentate gyrus *(DG)*	15.7	■	■	□	■
Oriens layer *(Or)*	8.9	□	■	□	□
Fimbria of the hippocampus *(fi)*	6.9	□	■	□	□
Hippocampus - unsegmented *(Hip-U)*	62.6	■	■	□	□
Basal Ganglia *(BG)*	28.7				
Nucleus accumbens *(Acb)*	7.2	□	■	■	□
Caudate nucleus *(Cd)*	9.4	■	■	■	■
Putamen *(Pu)*	5.7	■	■	■	□
Septum *(S)*	6.3	■	■	■	□
Bed nucleus of stria terminalis *(ST)*	0.1	□	■	□	□
Basal ganglia - unsegmented *(BG-U)*	28.8	■	■	■	□
Amygdala *(AMY)*	34.8	■	■	■	■
Anterior commissure *(ac)*	2.8	■	■	□	■
Anterior commissure, anterior limb *(aca)*	2.2	■	■	□	■
Anterior commissure, posterior limb *(acp)*	1.6	■	■	□	■
Lateral olfactory tract *(ol)*	3.8	■	■	□	□
Internal capsule *(ic)*	9.8	□	■	□	□
External capsule *(ec)*	14.2	■	■	□	□
Deep cerebral white matter *(dcw)*	8.2	■	■	□	□
Diencephalon *(DI)*	57.5				
Thalamus *(TH)*	31.5				
Habenular nuclei *(Hb)*	1.3	□	■	□	□
Habenular commissure *(hbc)*	0.1	□	■	□	□
Lateral geniculate nucleus *(LGN)*	1.5	□	■	□	■
Medial geniculate nucleus *(MGN)*	1.9	□	■	□	□
Thalamus - unsegmented *(TH-U)*	26.6	■	■	□	□
Hypothalamus *(HY)*	26.0				
Mammillary body *(M)*	0.8	□	■	□	□
Hypothalamus - unsegmented *(HY-U)*	25.2	■	■	□	□

Structure	volume (mm³)	MRI 48h	MRI 30d	Nissl	Myelin
Midbrain *(MB)*	82.8				
Superior colliculus *(SC)*	23.2				
Zonal and Superficial gray layer *(Zo-SuG)*	2.9	■	■	□	□
Optic nerve layer *(Op)*	4.2	■	■	□	□
Intermediate and Deep gray layer *(InG-DpG)*	9.7	■	■	□	□
Intermediate white layer *(InWh)*	5.5	□	■	□	□
Deep white matter *(DpWh)*	0.7	□	■	□	■
Commissure of the superior colliculus *(csc)*	0.5	□	■	□	■
Inferior colliculus *(IC)*	21.1				
Commissure of the inferior colliculus *(cic)*	0.9	□	■	□	■
Inferior colliculus - unsegmented *(IC-U)*	20.3	■	■	□	■
Dorsal nucleus of lateral lemniscus *(DLL)*	0.6	□	■	□	□
Posterior commissure *(pc)*	0.5	□	■	□	■
Periaqueductal gray *(PAG)*	13.4	■	■	□	□
Midbrain - unsegmented *(MB - U)*	24.0	□	■	□	■
Hindbrain *(HB)*	168.2				
Cerebellum *(Cb)*	128.8				
Molecular layer *(MlCb)*	50.6	■	■	□	□
Granule cell layer *(GlCb)*	39.9	■	■	■	□
Cerebellar white matter *(WhCb)*	29.4	□	■	□	□
Molecular layer of the paraflocculi *(MlPFl)*	8.9	■	■	□	□
Pons *(P)*	39.4				
Interpenduncular nucleus *(IP)*	0.7	■	■	□	□
Lateral lemniscus *(LL)*	4.9	■	■	□	□
Median raphe nuclei *(MR)*	0.6	□	■	□	□
Olivary nuclei *(O)*	2.0	■	■	□	□
Pontine nuclei *(Pn)*	2.1	■	■	□	□
Superior cerebellar peduncle *(SCP)*	1.3	□	■	□	□
Ventral tegmental nucleus *(VTg)*	0.1	■	■	□	■
Longitudinal fasciculus of the pons *(lfp)*	0.2	■	■	□	□
Motor root of trigeminal nerve *(m5)*	0.5	■	■	□	□
Middle cerebellar peduncle *(mcp)*	2.0	■	■	□	□
Pons - unsegmented *(P-U)*	24.9	■	■	□	■

Structure	volume (mm³)	MRI 48h	MRI 30d	Nissl	Myelin
Medulla oblongata *(MY)*	68.6				
Cochlear nucleus *(CN)*	3.9	■	■	□	□
Vestibular nucleus *(Ve)*	4.5	■	■	□	□
Facial nerve *(7n)*	0.5	■	■	□	□
Facial nucleus *(7N)*	0.6	□	■	□	□
Hypoglossal nucleus and central cervical nucleus *(12N)*	1.0	□	■	□	□
Cuneate nucleus *(Cu)*	2.9	■	■	□	□
Gracile nucleus *(Gr)*	1.1	□	■	□	□
Inferior olive *(IO)*	0.6	■	■	□	□
Area postrema *(AP)*	0.1	□	■	□	□
Nuclei of the solitary tract *(Sol)*	1.0	□	■	□	□
Paratrigeminal nucleus *(Pa5)*	0.1	□	■	□	□
Spinal trigeminal nucleus *(Sp5)*	7.1	■	■	□	□
Pyramidal tract *(py)*	2.0	■	■	□	□
Medial lemniscus *(ml)*	0.4	□	■	□	□
Dorsal spinocerebellar tract *(dsc)*	2.4	□	■	□	□
Ventral spinocerebellar tract *(vsc)*	3.9	□	■	□	□
Medulla oblongata - unsegmented *(MY - U)*	36.4	■	■	□	□
Fiber tracts *(FT)*	35.9	□	■	□	■
Cingulum *(cg)*	0.3	□	■	□	□
Fornix *(f)*	1.0	■	■	□	■
Optic chiasm *(och)*	1.1	□	■	□	■
Optic tract *(opt)*	5.4	□	■	□	■
Stria terminalis *(st)*	1.3	■	■	□	■
Mammillary peduncle *(mp)*	0.2	□	■	□	□
Mammillothalamic tract *(mt)*	0.4	□	■	□	□
Retromammillary decussation *(rmx)*	0.0	□	■	□	■
Cerebral peduncle *(cp)*	2.8	■	■	□	■
Fasciculus retroflexus *(fr)*	0.8	□	■	□	■
Mesencephalic trigeminal tract *(me5)*	0.6	□	■	□	■
Inferior cerebellar peduncle *(icp)*	1.5	■	■	□	□
Tectospinal tract *(ts)*	8.7	□	■	□	□
Sensory root of trigeminal nerve *(s5)*	11.6	■	■	□	□
Ventricles *(V)*	24.6	□	■	□	□
Lateral ventricle *(LV)*	19.2	■	■	□	■
Interventricular foramen *(IVF)*	0.3	□	■	□	□
3rd ventricle *(3V)*	1.2	□	■	□	□
Cerebral aqueduct *(Aq)*	0.9	□	■	□	□
4th ventricle *(4V)*	3.0	□	■	□	□
